# Factors Influencing the Uptake of Digital Health Interventions for Cardiovascular Disease Prevention Among Healthcare Providers: A Systematic Review

**DOI:** 10.5334/gh.1561

**Published:** 2026-06-04

**Authors:** Ariel Kraselnik, Hannah McGowan, Ines A. Amiali, Irene Gibson, Surabhi Joshi, Nasirumbi Magero, Corinne H. Miller, Sarah Oulousian, Abhinav Sharma, Mbiydzenyuy Ferdinant Sonyuy, Stefan Tino Kulnik, Nilay S. Shah

**Affiliations:** 1 Faculty of Chemistry, Universidad del Centro Educativo Latinoamericano (UCEL), Rosario, Argentina; 2 Ludwig Boltzmann Institute for Digital Health and Prevention, Salzburg, Austria; 3 Department of Health Promotion, Care and Public Health Research Institute (CAPHRI) Maastricht University Maastricht The Netherlands; 4 University of Montreal, Montreal, Canada; 5 Research Institute of the McGill University Health Centre, DREAM-CV Lab, McGill University, Montreal, Quebec, Canada; 6 National Institute for Prevention and Cardiovascular Health, Galway, Ireland; 7 Department of Noncommunicable Diseases, Disability and Rehabilitation, World Health Organization (WHO), Geneva, Switzerland; 8 Ministry of Health, Kenya; 9 Northwestern University Feinberg School of Medicine, Chicago, Illinois, USA; 10 Reconciliation and Development Association, RADA, Cameroon; 11 Africa NCDs Network (ANN), Cameroon; 12 Salzburg Research Forschungsgesellschaft m.b.H., Salzburg, Austria

**Keywords:** Barriers, cardiology, health personnel, facilitators, organization and administration, primary prevention, secondary prevention, telemedicine

## Abstract

**Background:**

Digital health interventions (DHIs) offer major potential for improving cardiovascular disease (CVD) primary and secondary prevention, but their adoption by healthcare providers (HCPs) remains inconsistent.

**Objective:**

To identify barriers and facilitators to DHI uptake in CVD primary and secondary prevention from HCPs’ perspectives.

**Methods:**

We conducted a systematic review of studies published between 2020 and 2024 that investigated HCPs’ perceptions of DHI implementation for CVD primary and secondary prevention. We appraised individual study quality using a validated tool. We performed a qualitative synthesis of reported barriers and facilitators, categorized according to country income level and according to the World Heart Federation Roadmap domains: HCPs, patients, technology, and health systems.

**Results:**

We included 125 primary studies (101 qualitative, 15 quantitative, 9 mixed methods). The most frequently cited barrier was excessive workload, both from existing responsibilities and additional tasks introduced by DHIs. The leading facilitator was the perceived positive clinical impact of DHIs—such as improved adherence, reduced hospital readmissions, and better outcomes. HCP motivation, adequate training, and system integration also facilitated adoption. Many factors—like effects on HCP-patient relationships and workflow—functioned as either barriers or facilitators, depending on the setting. Patient-related barriers included limited digital access and literacy; facilitators included perceived gains in patient-centered care. Health system factors such as organizational structure, financing, and policy support were commonly mentioned, with mixed views. Technology-related facilitators included usability, adaptability, and integration with electronic records; instability was a key barrier.

**Conclusions:**

This is the first systematic review to synthesize post-COVID-19 literature on HCPs’ perceptions of DHIs in CVD primary and secondary prevention. While offering a rich, global overview, limitations include a predominance of qualitative studies and lack of data from low-income countries. Effective implementation must address workload, align with workflows, and build trust through training and leadership.

**Lay Summary:**

This research analyzed 125 studies from 33 countries to understand the factors that influence healthcare professionals’ uptake of digital health tools, such as apps and wearable devices, for preventing cardiovascular disease.

The leading facilitator for adoption is the perceived positive clinical impact; doctors and nurses are highly motivated to use digital tools when they help patients follow treatments better, reduce hospital readmission rates, and improve overall heart health.

The most significant barrier is the perceived excessive workload. While some digital health tools can be time-saving, many providers feel that they add burdensome technical tasks to their already busy schedules, which undermines their acceptance.

Uptake is also influenced by patient-related factors, such as digital literacy and internet access, as well as technological factors like how easily a tool integrates into existing hospital computer systems.

To improve the future of cardiovascular care, digital tools should be co-designed with clinicians to ensure they fit seamlessly into daily work routines and are supported by proper training and strong institutional leadership.

## Graphical Abstract

**Figure:**
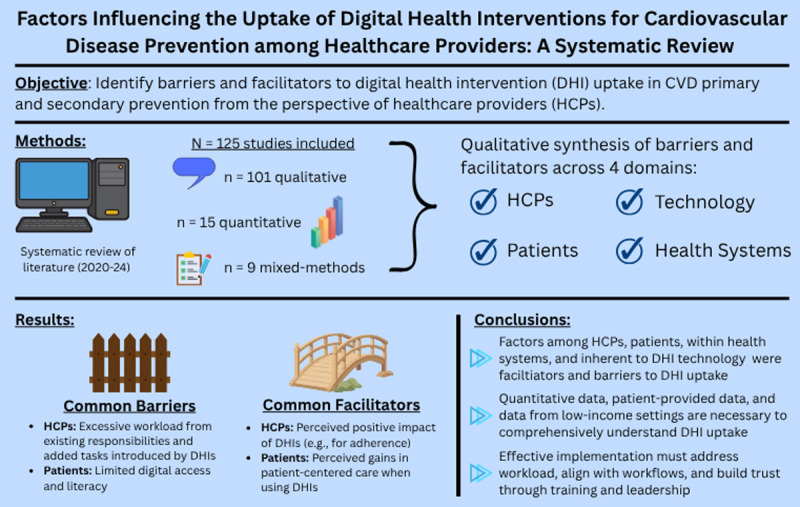

## Introduction

Cardiovascular diseases (CVDs) remain the leading cause of death globally, taking an estimated 17.9 million lives each year (1). A significant discrepancy persists between best practice (based on high-quality evidence) and the care provided in routine practice (2). There is increased recognition of the potential of digital health interventions (DHIs) and digital health tools to address this discrepancy and transform and improve CVD care.

DHIs include an array of tools that facilitate enhanced patient care. Examples include DHIs that enable remote monitoring for efficiency (e.g., ‘smart’ versions of blood pressure cuffs), remote monitoring and management for improved care (e.g., apps and devices for daily measurement of weight), clinical decision support (e.g., apps that integrate within electronic health records), patient engagement (e.g., solutions that promote patients’ active participation in their care), telehealth and virtual visits, point-of-care and workflow enhancement (e.g., communication and sharing of clinical data electronically), and patient access to clinical data (e.g., online portals showing results of lab or imaging tests) (3). Evidence of effectiveness is available for numerous DHIs that support CVD primary and secondary prevention, from physical activity promotion (3,4,5,6,7), healthy eating (8,9,10,11), weight management (7,12,13), and smoking cessation (14,15,16,17) to the management of hypertension (11,18,19,20,21,22), prediabetes (23,24), and type 2 diabetes (25,26).

Despite the availability of many evidence-based DHIs for the management and prevention of CVDs, low acceptability from healthcare providers (HCP), patients, and other key stakeholders remains a significant roadblock to the global uptake of these DHIs (27). Acceptability of clinical interventions has been defined as ‘the perception among implementation stakeholders that a given treatment, service, practice, or innovation is agreeable, palatable, or satisfactory’ or satisfaction with various aspects of the innovation (e.g., content, complexity, comfort, delivery, and credibility) (28). Specific to technology acceptance, the Unified Theory of Acceptance and Use of Technology describes 11 factors influencing use intention and use behavior, such as the expectation of performance, expectation of effort, social influence, and facilitating conditions. There is an urgent need to facilitate global adaptation and uptake of existing evidence-based DHIs to bridge the gap between best practice and usual care, as emphasized in the World Health Organization (WHO) Global Strategy on Digital Health 2020–2025 strategic objective 4 (advocate people-centered health systems that are enabled by digital health), which states:

‘The strategic objective places people at the center of digital health through the adoption and use of digital health technologies in scaling up and strengthening health service delivery. The individual is an essential component in the delivery of trust-based, people-centered care. This focus covers not only patients, families and communities but also the health workers who need to be prepared to deploy or use digital health technologies in their work’ (29).

Several investigations focus on patient-level facilitators and barriers to DHI uptake. However, HCPs represent another important stakeholder group for the implementation of DHIs in clinical practice because HCPs are often the group that introduces, initiates, or recommends DHIs to patients. We therefore performed a systematic review to identify factors influencing the uptake of DHIs for CVD primary and secondary prevention, with a focus on acceptability among HCPs.

Two recent systematic reviews, Whitelaw et al. (30) and Borges do Nascimento et al. (31), summarize prior evidence regarding facilitators and barriers to the uptake of DHIs among HCP. Whitelaw et al. (2021) conducted a systematic scoping review identifying key barriers such as increased workload, lack of integration with clinical workflows, insufficient institutional support, and limited reliability or usability of technologies, while facilitators included organizational endorsement, perceived usefulness, and enhanced communication and efficiency. More recently, Borges do Nascimento et al. (2023) performed an umbrella review encompassing over one hundred systematic reviews, reporting that infrastructural limitations, psychological barriers (e.g., lack of confidence or digital competence), and concerns about time burden were the most common obstacles, whereas education, training, and demonstration of clinical effectiveness were consistent facilitators of adoption. Together, these reviews highlight that both individual- and system-level factors must be addressed to optimize DHI implementation in healthcare settings. However, the review by Whitelaw et al. did not include evidence generated during the COVID-19 pandemic, a period in which the relevance and perceptions of DHIs were deeply modified. The review by Borges do Nascimento et al., while more recent, included only systematic reviews, did not analyze primary studies, and did not focus specifically on CVD.

The objective of this systematic review is to address the existing knowledge gap by synthesizing evidence on the use of digital health interventions for CVD primary and secondary prevention, focusing on studies published during and after the COVID-19 pandemic (2020–2024), and thereby extending prior evidence syntheses conducted in CVD populations.

## Methods

This systematic review followed the Preferred Reporting Items for Systematic Reviews and Meta-analyses (PRISMA) 2020 checklist (32) and was registered in the International Prospective Register of Systematic Reviews database (PROSPERO Number: CRD42024535062).

### Eligibility criteria

Primary studies that address factors (i.e., facilitators and barriers) that influence the uptake of DHIs by HCPs, published between 2020 and 2024, were eligible. For this review we only considered studies that involved HCPs as participants, including physicians, nurses, social workers, pharmacists, caregivers, psychologists, nutritionists/dietitians, physiotherapists, paramedics, rehabilitation professionals, podiatrists, and exercise physiologists. DHIs were considered broadly to include tools that facilitate communication with and engagement of patients (such as telehealth); collect and summarize patient-level clinical data (such as wearable devices); and measure quality of care (such as clinical decision support tools), guided by the American Medical Association’s classification of digital health tools (27) and the WHO 2.0 classification of digital interventions (33). CVD prevention was defined broadly, including both primary and secondary prevention, with no restrictions based on type of CVD (e.g., atherosclerotic CVD, heart failure, arrhythmia, etc.).

Qualitative, quantitative, and mixed-methods study designs were included in order to capture the breadth of the evidence, provided that the study obtained HCPs’ perspectives (e.g., interviews, focus groups, questionnaires). Excluded were studies without relevant extractable data, such as studies reporting only quantitative usability scores of a DHI, and studies informing the design of a DHI rather than evaluating HCPs’ perceptions of an existing DHI. Additionally, excluded were case studies, conference abstracts, posters, and non-peer-reviewed manuscripts. Studies that did not focus on CVD prevention, did not include perspectives from HCPs, or did not involve patient-facing DHIs (i.e., there was no direct or indirect interaction and engagement of patients with the digital tool) were also excluded.

### Information sources

We searched Ovid MEDLINE, CINAHL, Embase, Scopus, Web of Science, ACM Digital Library, IEEE Xplore, and Google Scholar in May 2024. We also performed a collateral search of reference lists from relevant systematic reviews.

### Search strategy and study selection

Records were retrieved using the search strategy developed and executed by a clinical librarian at the Galter Health Sciences Library at Northwestern University (Chicago, IL, USA). Full search strategies are provided in Supplemental File 1. Following the creation and execution of the search strategy, the search results were imported into the Rayyan systematic review management platform. A pilot screening of 100 titles and abstracts was conducted by all members of the research team. Any disagreements were resolved through discussion to ensure alignment in the screening process.

After the pilot screening, records were distributed among nine members of the research team for independent double-screening of titles and abstracts to identify studies that met the inclusion criteria or those in which the available information was insufficient for a decision. After compiling the list of studies deemed potentially appropriate based on the study aims, design, and overall findings, full-text versions of all selected articles were reviewed. This stage involved a detailed review of the studies to determine if the inclusion criteria were indeed met and if extractable (relevant) findings related to factors influencing uptake of DHIs could be obtained. Disagreements regarding inclusion were resolved through discussion and consensus among the study team members. These disagreements often revolved around decisions relating to the determination of whether the tool was patient-facing, whether the reported factors were relevant to the uptake of DHIs overall, and whether the clinical population aligned with our inclusion criteria. Excluded full-text articles were recorded in the PRISMA diagram, stating the reasons for exclusion according to the inclusion and exclusion criteria.

### Data collection

Data was extracted from all finally included full-text articles using a Microsoft Excel file. Four members of the research team divided up the full-text articles and extracted data for the following categories: title, DOI number, first author name, year published, country or countries where the study was conducted, classification of country income level, WHO classification of country region, study aims, study design, classification of HCPs, classification of DHIs (according to American Medical Association and WHO 2.0 classification), description of DHI, type of CVD prevention (primary versus secondary), sample characteristics (total sample size, sub-samples of different HCPs if applicable, age, sex), quantitative results (type of variable, measurement method, descriptive statistics), and qualitative results (type of analysis, facilitators, barriers). For an alignment of the data extraction approach, each team member first completed five to ten full-text data extractions separately. The team then met and discussed questions pertaining to the classification of data (e.g., categories of DHIs and types of CVD prevention) and best approaches for consistent data recording. Afterwards, each of the four team members independently completed the remaining full-text extractions with further regular meetings to discuss questions and align discrepancies. For a select number of studies, two members independently extracted the same data and met for discussion to reach a consensus.

### Quality of evidence appraisal

Six reviewers independently assessed the risk of each included study using the ‘Standard Quality Assessment Criteria for Evaluating Primary Research Papers from a Variety of Fields’ tool from the University of Alberta (34). This tool considers research design, study population, lack of endpoint data, potential for bias (including selection, measurement, and confounding), and internal and external validity and accommodates assessment of quantitative and qualitative studies. First, up to five studies were appraised by all six reviewers together for familiarization with the quality assessment tool and to align judgements among the reviewers. The remainder of the included studies was then divided among the six reviewers. Queries were resolved in consultation with a senior researcher on the study team.

### Data synthesis

We performed a qualitative synthesis of reported factors that influence HCPs’ uptake and acceptance of DHIs. Extracted data were coded and organized into overarching factors (e.g., attitudes, accessibility, usability, etc.) and subsequently categorized into one of four domains according to the World Heart Federation Roadmap for Digital Health in Cardiology: HCPs, patients, technology, and the health system (35). Coding was conducted by one researcher with constant comparison and peer review by other members of the research team. This continued progression of coding data and organization to factors led to an ongoing revisiting of factor terminology and collaborative decisions on whether to merge, separate, or develop new factors. Quantitative data (e.g., Likert scale responses, odds ratios) were also coded using this approach, based on their reported findings, such as statistically significant factors influencing DHI uptake or identified factors from descriptive analyses. The decision to analyze qualitative and quantitative data together was made 1) to allow for an overall synthesis of factors influencing uptake across study designs, 2) due to the large number of included qualitative studies, and 3) due to the overall focus of this review’s aim on perspectives of HCPs. The frequency of reported factors was visualized in bar charts, indicating the number of occurrences of any one factor across all included studies, how often a factor was reported or framed as a facilitator or barrier, and differentiating between country income levels. Supplemental File 2 contains the extracted data and coding.

## Results

**Figure 1:**
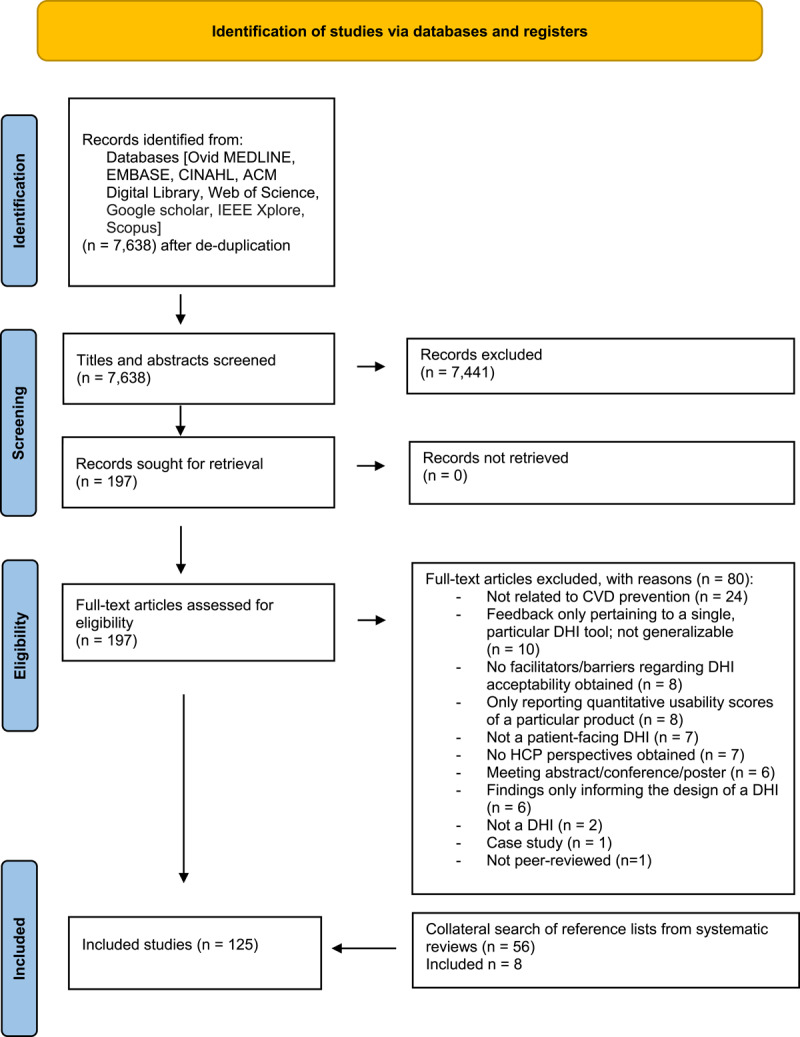
PRISMA flow diagram.

After removal of duplicates, 7,638 abstracts were eligible for screening, of which 117 studies were included in the analysis. Eight articles from the collateral search were added, resulting in the final number of 125 studies included in the analysis (Figure 1).

### Study characteristics

**Figure 2:**
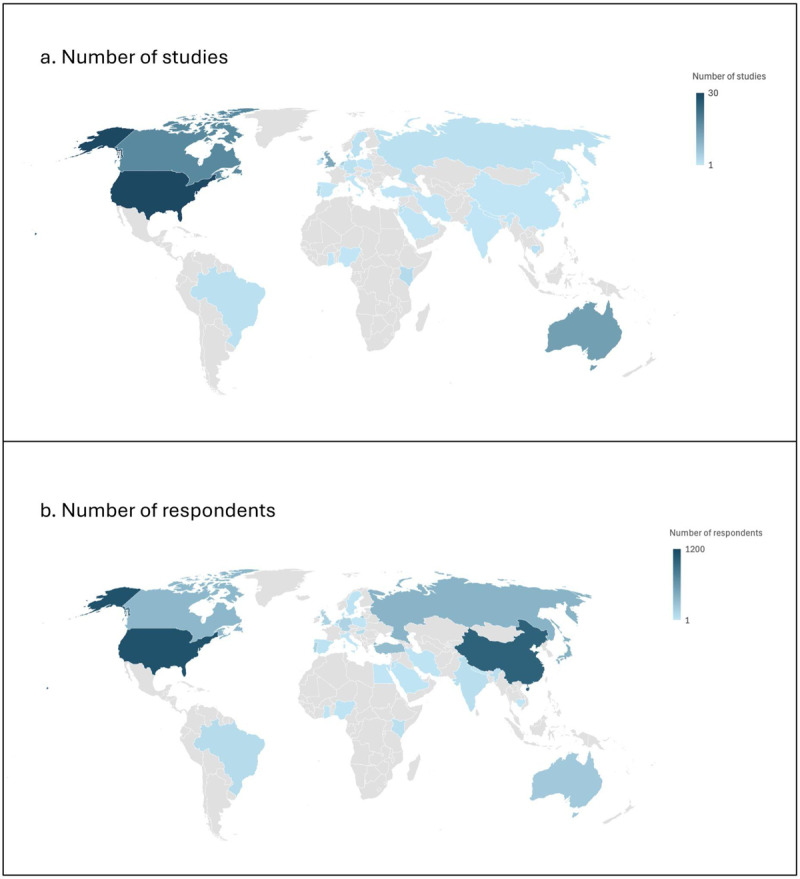
Global distribution of included studies **(a)** and number of respondents **(b)**.

One hundred and one studies were qualitative (predominantly interview and focus group studies) (36,37,38,39,40,41,42,43,44,45,46,47,48,49,50,51,52,53,54,55,56,57,58,59,60,61,62,63,64,65,66,67,68,69,70,71,72,73,74,75,76,77,78,79,80,81,82,83,84,85,86,87,88,89,90,91,92,93,94,95,96,97,98,99,100,101,102,103,104,105,106,107,108,109,110,111,112,113,114,115,116,117,118,119,120,121,122,123,124,125,126,127,128,129,130,131,132,133,134,135,136), 15 were quantitative (all surveys) (137,138,139,140,141,142,143,144,145,146,147,148,149,150,151), and nine were both qualitative and quantitative (152,153,154,155,156,157,158,159,160). The distribution of studies by regional income level included high-income countries (107) and middle-income countries (18), with no studies conducted in low-income countries. The included studies’ main characteristics are summarized in Supplemental File 3 (Table S3.1). The global distribution of included studies and the number of respondents are shown in Figure 2. The raw data for Figure 2 is presented in Supplemental File 3 (Table S3.2). Overall, 33 countries were represented in this review, presenting perspectives from 8,821 HCPs (mean 72 participants [range 1–1753] per study). The average percentage of males was 35 (range 0–87).

Clinical characteristics of study samples were described as follows: CVD and/or CVD risk factors in 41 studies (33%), hypertension in 23 studies (18%), heart failure in 19 studies (15%), general population samples in 12 studies (10%), coronary artery disease in eight studies (6%), pregnancy-related hypertension and/or diabetes in seven studies (6%), atrial fibrillation or risk of atrial fibrillation in six studies (5%), implantable cardiac device in two studies (2%), and one study (1%) each for cancer, cardiac surgery, children with CVD, hospitalized patients, heart transplantation, stroke, and transcatheter aortic valve implantation.

The respondent groups represented across all included studies were physicians (78%), nurses (11%), allied health professionals including physiotherapists and clinical physiologists (2%), pharmacists (2%), administrators/managers (2%), policymakers (<1%), healthcare assistant staff (<1%), psychologists (<1%), social workers (<1%), researchers (<1%), and other roles and professions (4%). The average age of respondents was not calculated because it was not reported in 90 out of 125 studies.

### Quality of primary studies

Overall, on a scale from zero (indicating lowest quality) to one (indicating highest quality), half of the qualitative studies had a quality score of 0.80 or higher, and three quarters of studies had a quality score of 0.65 or higher. For the quantitative studies, half had a quality score of 0.81 or higher and three-quarters a quality score of 0.69 or higher. Mixed-methods studies were assessed using both qualitative and quantitative criteria, such that qualitative components were assessed qualitatively and quantitative components quantitatively. Quality appraisals for all included studies are given in Supplemental File 4.

### Main Findings

Our data identify key factors reported by HCPs (i.e., factors representing HCPs’ perceptions) that influence the adoption of DHIs in routine practice. We categorized these factors as related to one of four domains: HCPs, patients, technology, and the health system. In the primary studies, factors could be reported or framed as either facilitators or barriers. For example, regarding the factor ‘HCP attitudes toward technology’, positive attitudes act as a facilitator, and negative attitudes act as a barrier. Here, we report on the most prominent factors and their respective framings as either facilitators or barriers.

### Factors influencing DHI uptake related to HCPs

The most frequently reported facilitator was the perceived positive clinical impact, which was mentioned 83 times. Moreover, HCPs’ motivation for using DHIs was cited as a facilitator 38 times, and HCP training/education on DHIs 36 times. The type of HCP-patient relationship (e.g., rapport, familiarity, trust), the expected impact of DHIs on the HCP-patient relationship dynamics, a perceived lower workload due to the DHI, and HCP technology attitudes towards DHIs were also reported as facilitators, 30, 25, 22, and 21 times, respectively. Following closely behind, HCP autonomy and trust in DHIs were reported as facilitators 19 times.

The most frequently reported barrier was the perceived added workload due to DHIs, identified as a negative factor 56 times. Additionally, HCPs’ current workload was cited as a barrier 38 times. Taking both the added and current workload together, the ‘workload’ factor emerges as the main barrier perceived by HCPs in adopting DHIs. Other factors mostly seen as barriers were HCP trust of DHIs (33 times), HCP negative attitudes towards technology (30 times), perceived negative clinical impact of the DHI (26 times), HCP improper use or misunderstanding of DHIs (23 times), concerns about privacy/security (23 times), and concerns of increased responsibilities and liability (17 times).

Due to the mixed groups of HCPs in many of the study samples, it was not possible to determine how factors of uptake differed between HCP types (e.g., physicians, nurses, physiotherapists).

Lastly, in terms of the top five factors influencing uptake of DHIs in high- and middle-income countries, similar overlapping factors were identified in both groups. They were perceived clinical impact, perceived added workload due to DHIs, and motivation to use DHIs. However, among high-income countries, trust in DHIs, HCPs’ attitudes, and HCP training emerged as additional key factors, while among middle-income countries, HCP views of DHI on (personal and patient) privacy and security and current workload were more prominent.

**Figure 3:**
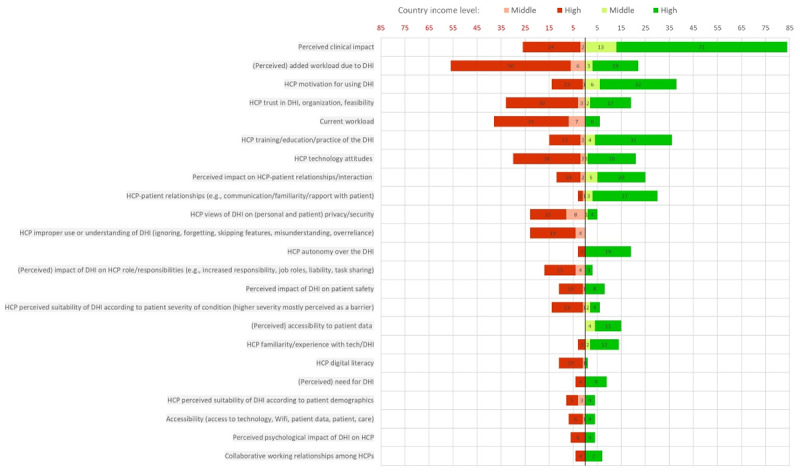
Most commonly cited (≥10 times) factors influencing digital health intervention (DHI) uptake by health care providers (HCPs). Numbers represent the number of times that the factor was cited, either as a facilitator (green) or as a barrier (red). Studies from high-income countries are represented in dark green and red, and studies from middle-income countries in light green and red.

Figure 3 presents the frequency of the most commonly cited (≥10 times) factors related to HCPs, their framing as either facilitators or barriers in the primary studies, and their distribution across middle- and high-income country levels. The full figure with all the factors identified is in Supplemental File 5.

### Factors influencing DHI uptake related to patients, technology and health systems

Patient-related factors influencing HCPs’ adoption of DHIs were the perceived impact of DHIs on patient-centered care (42 studies) and patients’ access to technology (i.e., mobile phones, internet) and care (38 studies), with the former mostly reported as a facilitator and the latter as a barrier for HCP adoption. Moreover, patient training/education on the DHI and perceived impact of the DHI on patient access to care were reported as factors in 33 studies, with training/education reported as a facilitator 25 times and perceived impact of the DHI on improved patient access to care reported 30 times.

Additionally, 31 studies frequently reported patient digital literacy and patient motivation for using DHIs as factors for HCP adoption. Patient digital literacy was primarily reported as a barrier for HCP adoption (29 times), whereas patient motivation for DHIs was reported as both a facilitator (18 times) and a barrier (17 times) for HCP adoption. Other factors mostly seen as barriers were patients’ improper use or misunderstanding of DHIs, patients’ older age, and patients’ attitudes towards technology, whereas a perceived impact of DHIs on improved patient empowerment was seen predominantly as a facilitator for HCP adoption.

HCPs’ perspectives of health system factors influencing adoption of DHIs centered on work structure (e.g., staffing, scheduling, workflow, work environment), cited in 77 studies, serving both as a facilitator (58 times) and as a barrier (43 times). Financing and funding aspects (e.g., sustainability, staff compensation) appeared in 34 studies, reported both as a barrier (23 times) and as a facilitator (17 times). Similarly, policies, laws, and regulations (e.g., insurance, reimbursement) were reported in 32 studies, 17 times as a facilitator and 21 times as a barrier. Other notable factors included information and communication technology (ICT) equipment and software, health system leadership influence, and physical space. ICT equipment and software were predominantly reported as a barrier due to mostly a lack thereof (19 compared to 6), whereas leadership influence in the health system was mostly reported as a facilitator, and physical space for DHIs showed mixed results as both a barrier and facilitator. Overall, across middle- and high-income countries, no substantial differences were observed among the top five health system factors influencing uptake of DHIs. The only variation was in their relative ranking of their reported frequencies, with factors of financing and funding of DHIs slightly more prominent than polices, laws, and regulation factors in high-income countries, whereas this was reversed in middle-income countries.

Lastly, HCPs’ perceptions of technology-related factors influencing DHI adoption emphasized DHI usability and user-friendliness (59 studies), more often reported as a facilitator (44 times) as compared to a barrier (28 times). Adaptability of the DHI (e.g., to patient needs, HCP and patient preferences, language) was also another key factor as reported in 54 studies, mostly seen as a facilitator (40 times) as compared to a barrier (24 times). Integration of the DHI with other systems (including electronic health records and clinical software) was discussed in 37 studies, with this factor reported as a facilitator 29 times and a barrier 11 times. Other relevant aspects included data visualization and presentation, technical stability of the DHI (often a barrier), integration of the DHI into the clinical workflow, accuracy/reliability of the DHI content, automation, and scientific evidence-based aspects of the DHI.

**Figure 4:**
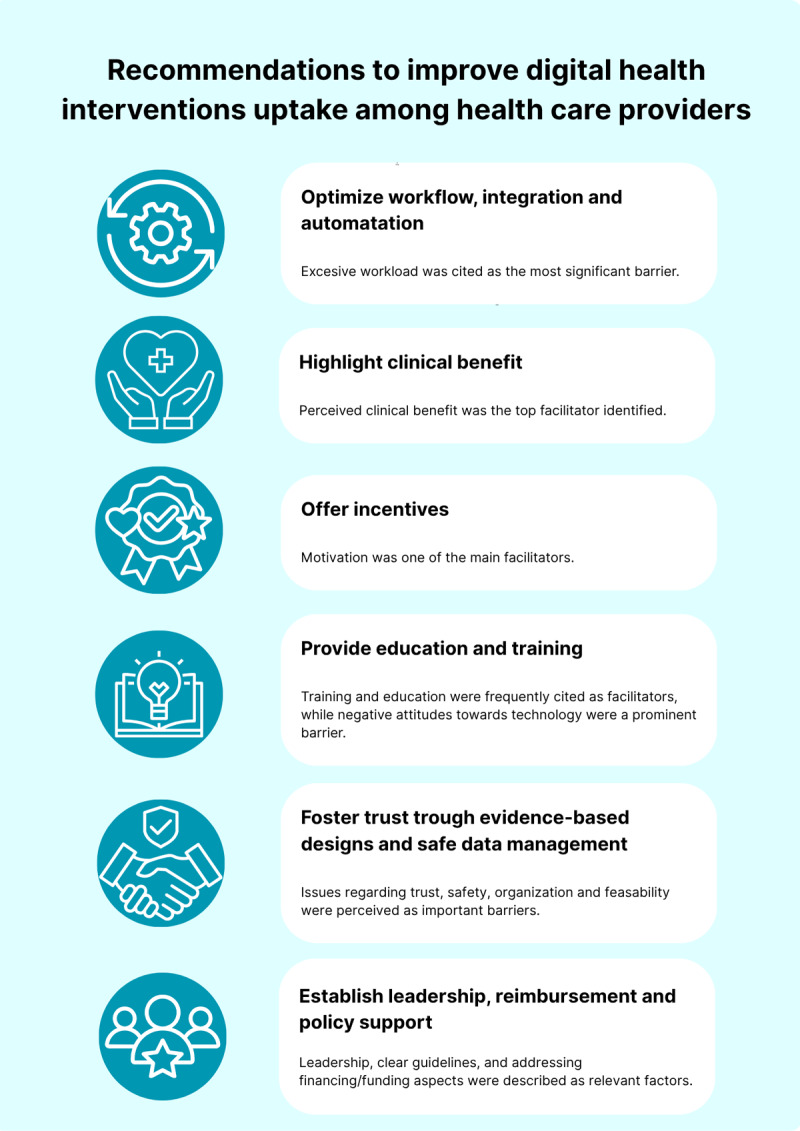
Key recommendations to improve health care providers’ uptake of digital health interventions for cardiovascular disease primary and secondary prevention.

Figures displaying all factors related to HCPs, patients, technology, and health systems are given in Supplemental File 5. Figure 4 summarizes key recommendations to improve DHI uptake among HCPs in CVD prevention, based on the findings of this review.

## Discussion

The successful adoption of DHIs among HCPs hinges on addressing critical barriers while leveraging facilitators. The most frequently reported barrier to DHI uptake among HCPs was excessive workload, including increased responsibilities such as data management and technical support and current workload, leading to time constraints and burnout. Nevertheless, perceived influence on workload was cited as a facilitating factor 22 times, reflecting HCPs’ perception that DHIs can also help alleviate some tasks and reduce their overall workload.

As seen across multiple other fields, skepticism about DHIs among HCPs, highlighted by concerns about technology attitudes and trustworthiness, underscores the need for transparency, robust evidence, adequate training, and integration into existing clinical processes. Structural factors and infrastructure limitations (e.g., availability of wireless internet access) were also relevant, particularly in middle-income settings.

It is encouraging that the perceived clinical impact was the most prominent facilitator, with HCPs recognizing the potential of DHIs to improve adherence, reduce readmissions, and enhance clinical outcomes. In the context of CVD prevention, these barriers and facilitators are especially relevant. For example, DHIs that support medication adherence or early detection of decompensation can help reduce readmissions for heart failure, aligning with HCPs’ perceptions of clinical benefit.

This highlights an opportunity to leverage HCPs’ positive perception to promote the wider adoption of DHIs among them, ultimately improving CVD prevention efforts. Motivation, driven by perceived value, interest, and financial incentives, also played a significant role as a facilitating factor. Adequate training and education and building trust through pilot testing and evidence-based practices are critical in fostering confidence and positive attitudes.

In the primary studies included in this review, most factors were framed as both facilitators and barriers. For example, the expected impact of DHIs on HCP-patient relationships was frequently reported as both a facilitator and a barrier, suggesting that while a DHI can enhance communication, it may also be perceived as disrupting established and valued HCP-patient interactions.

These findings highlight the importance of targeted efforts to optimize DHI adoption among HCPs, including enhanced training, education, workload management strategies, and organizational support.

HCPs also identified several patient-, health system-, and technology-related factors influencing the adoption of DHIs. Regarding patient-related aspects, the perceived impact on patient-centered care and the perceived impact on increased access to care were commonly seen as facilitators. However, patients’ existing access to technology and care, low digital literacy, improper use or misunderstanding of DHI features, and patients’ older age emerged as significant barriers, both in high- and middle-income countries. Such findings are aligned with prior studies evaluating DHIs in older adults.

Perceptions about health system factors influencing DHI adoption were mostly mixed, seen frequently as both facilitators and barriers. The main factor cited was work structure, which includes staffing, scheduling, workflow, guidelines, job roles, task delegation/sharing, and work environment. Financing and funding, as well as policies and regulations, were also perceived with this duality. This variability in perception reflects an opportunity to optimize these structural elements in ways that enhance DHI adoption, whether by improving workflow integration, ensuring sustainable financing models, or adapting policies to better support implementation.

In the technology domain, positive user experience and adaptability of the DHI to different scenarios (such as language preferences or diverse health conditions) were widely recognized as important, with mostly positive perceptions. Integration with electronic health records and existing clinical software was often seen as a relevant facilitator for DHI uptake, while technical stability was more frequently cited as a challenge. Other key aspects, such as integration of the DHI into the clinical workflow, accuracy of the DHI content, automation, and scientific evidence in support of the DHI, were seen as important factors.

Our findings identified similar barriers and facilitators as previous reviews, with several key differences. For example, a recent umbrella review by Borges do Nascimento et al. (31), which included 108 systematic reviews, evaluated barriers and facilitators for DHI uptake among HCPs, although only two of the reviews focused exclusively on CVD. In this review, infrastructure and technical barriers (such as lack of existing technologies/devices, issues with connectivity speed or access to electricity) and personal/psychological barriers (such as HCPs’ resistance to change, difficulty understanding the technology, or perception of less human interaction) were the two most frequent barriers described, while excessive workload was the third most reported barrier. This contrasts with the findings of our review, in which excessive workload was identified as the most frequent barrier, and only a few studies cited HCP accessibility as a barrier for DHI uptake.

This difference could be explained by several factors: For instance, more than half of the reviews included in the review by Borges do Nascimento et al. were from Africa, Latin America, and Caribbean regions, where infrastructure and technical barriers may be more prominent, while our review included mostly high-income countries. Additionally, Borges do Nascimento et al. included older studies where overcoming basic infrastructural and technical challenges could be significant barriers. Since we focused on studies published from 2020 onwards, this barrier may become less apparent as technology evolves, the prevalence of digital technology among the general population increases, and digital technology becomes more pervasive to the clinical domain. In this respect, the COVID-19 pandemic acted as a catalyst for the development and uptake of DHIs for both HCPs and patients but also amplified workload pressures on HCPs, increasing its salience as a barrier in more recent studies included in the current review. The heightened demands on HCPs during and after the COVID-19 pandemic may have shifted focus toward time constraints and stressors related to workload. Lastly, our review focused exclusively on CVD primary and secondary prevention, and HCPs in this clinical field may perceive different barriers and facilitators for DHI uptake compared to other clinical domains.

Developers should take note of our findings, addressing the main barriers to improving the uptake of DHIs by HCPs and integration into existing clinical processes. This could be achieved by optimizing workflows (i.e., by reducing duplicate or unnecessary tasks), integrating seamlessly into current systems without adding complexity, utilizing automation to handle repetitive activities (such as sending reminders or generating routine reports), and ensuring that tasks related to DHIs (e.g., data entry, follow-ups) fit naturally into HCPs’ daily routines rather than interrupt them. HCPs need to be involved in DHI development to ensure optimal workflow integration and user-friendly design.

Furthermore, policymakers should establish clear guidelines, support educational programs, provide financial incentives, and prioritize trust-building measures to ensure the sustainable adoption of DHIs among healthcare professionals in CVD prevention, as these were the most commonly identified facilitators of DHI uptake.

### Strengths and Limitations

A key strength of this study is its comprehensive analysis of HCPs’ perceptions of factors influencing DHI adoption, synthesizing findings from 125 studies published after 2020. Overall, the review incorporates data from 8,821 healthcare professionals across 33 countries, providing a broad and diverse international perspective. By focusing exclusively on recent literature, this review captures the post-COVID-19 pandemic healthcare landscape, making it the first review to examine HCP perspectives on DHI adoption in a world where digital health plays an increasingly central role. The study also covers a broad range of factors, spanning patient-related, technological, and health system domains and offering a nuanced understanding of both facilitators and barriers. The identification of factors that may constitute barriers but may also serve as facilitators highlights opportunities for optimization rather than fixed obstacles, providing valuable insights for future DHI implementation.

However, some limitations should be acknowledged. The variability in study methodologies and healthcare settings presents challenges, limiting generalizability across different contexts. Most studies were qualitative, which limits the ability to generalize the findings to broader populations or settings. While qualitative studies provide in-depth insights into the experiences and perceptions of HCPs, they are often context-specific and may not represent the views of all healthcare professionals across different regions or healthcare systems. Additionally, the lack of quantitative data makes it challenging to measure and compare the magnitude of the identified facilitators and barriers, as well as their relative impact on DHI adoption.

Another limitation of the evidence is that we did not find relevant studies from low-income countries, highlighting a persistent geographical imbalance in digital health literature. This gap likely reflects disparities in technological infrastructure, research funding, and access to digital health tools, which may limit the generalizability of current evidence and underscore the need for greater investment and capacity-building efforts in these settings. The absence of data from these settings means that the perceptions of HCPs in resource-limited environments, where DHI adoption may face unique challenges, are not represented.

### Future Research

Future research should address gaps identified in this review, specifically the lower proportion of quantitative assessments of DHI adoption and the lack of studies from low-income settings. This could offer a more comprehensive global picture and understanding of the factors influencing DHI adoption. In this review, the perspective of patients was incorporated through patient-related factors, but these were derived from HCPs’ reports and not from patients directly. There is therefore scope for replicating this review with a focus on primary studies that collected data from patients directly.

The key findings and recommendations from this review provide points of departure for local implementation studies informed by theory from the field of implementation science (162). For example, implementation studies may draw on the barriers and facilitators reported in this review as a starting point for identifying the most pertinent factors for DHI adoption at a local study setting. Moreover, implementation studies may develop concrete strategies that operationalize the key recommendations from this review for a specific setting, involving local HCPs and patients in the development of such strategies through co-design methods (163). The success of such a locally developed strategy or several alternative strategies may then be formally evaluated in implementation studies. This can provide valuable case examples and inspiration to others.

Despite the clear benefits of DHIs, their widespread adoption raises important sustainability considerations that current discussions rarely address. The growing reliance on cloud-based data storage and processing demands substantial energy use from data centers, contributing significantly to the sector’s carbon footprint (161). Future research should therefore address questions of environmental sustainability in digital health planning, promoting energy-efficient infrastructures and responsible data management practices.

## Conclusions

The most frequently reported barrier to HCPs’ uptake of DHIs for CVD primary and secondary prevention was the perception of an excessive workload, both in terms of current responsibilities and those anticipated with the implementation of DHIs. It is promising that the perceived clinical benefits emerged as the main facilitator identified, with HCPs acknowledging the potential of DHIs to enhance adherence, decrease readmissions, and improve clinical outcomes. This presents an opportunity to capitalize on HCPs’ favorable views to encourage broader adoption of DHIs, ultimately strengthening cardiovascular care.

To balance barriers and facilitators, DHIs must align with clinical workflows and address HCPs’ concerns through clear role delineation, effective training, and leadership support. Particular emphasis should be placed on avoiding additional workload, highlighting the clinical benefits of DHIs and offering incentives, since these were the most influential factors identified among HCPs.

## Additional File

The additional file for this article can be found as follows:

10.5334/gh.1561.s1 Supplemental Files. Supplemental file S1 to S5.

## Data Availability

All data generated or analyzed during this study are included in this published article and its supplemental files.
