# Supplementary material for: Factors Influencing the Uptake of Digital Health Interventions for Cardiovascular Disease Prevention Among Healthcare Providers: A Systematic Review
Source: Glob Heart. 2026 Jun 4;21(1):45. doi: 10.5334/gh.1561 (PMC13239346; doi:10.5334/gh.1561)

Supplemental file 1. Search strategies

Database: Ovid MEDLINE(R) ALL, 1946 to May 7, 2024
Date searched: May 9, 2024
Results: 6541

Line	Query
1	digital health/ or exp telemedicine/ or Internet-based intervention/ or "Therapy, Computer-Assisted"/ or wearable electronic device/ or cell phone/ or mobile applications/ or exp "Computers, Handheld"/ or internet/ or Software/ or "Decision Support Systems, Clinical"/ or exp electronic health records/ or "medical records systems, computerized"/ or digital technology/
2	(tele-cardiology or telecardiology or tele-care or telecare or tele-consult* or teleconsult* or tele-diagnos* or telediagnos* or tele-health* or telehealth* or tele-medic* or telemedic* or telemetr* or tele-monitor* or telemonitor* or tele-nurs* or telenurs* or tele-psych* or telepsych* or tele-rehab* or telerehab* or tele-surveillance or telesurveillance or tele-therap* or teletherap* or telephone-consult* or telephone-based-consult* or clinical-decision-support-system* or cds-system* or computer-assisted or computer-based or computerized-medical or computerised-medical or computerized-patient or computerised-patient or computerized-clinical or computerised-clinical or computerized-decision-support or computerised-decision-support or automated-health or automated-medical or cyberconsult* or cyber-consult* or digital* or e-care or ecare or electronic-consult* or e-consult* or econsult* or e-health* or ehealth* or e-rehab* or erehab* or electronic-device* or electronic-health* or EHR or EHRS or electronic-medical or EMR or EMRS or electronic-portal or e-portal or eportal or hand-held-computer* or handheld-computer* or health-application* or healthcare-application* or health-care-app* or health-technolog* or healthcare-technolog* or health-care-technolog* or internet or online or web or app or apps or mobile-application* or mobile-health or mhealth or m-health or mobile-phone* or mobile-telephone* or cell-phone* or cellphone* or cellular-phone* or cellular-telephone* or smart-phone* or smartphone* or smart-watch* or wristband* or fitness-tracker* or mobile-technolog* or personal-digital-assistant* or remote* or software or tablet* or virtual or wearable*).ti,ab.
3	1 or 2
4	exp cardiovascular diseases/ or exp dyslipidemias/
5	(cardiovascular or cvd or heart or cardiac or cardio* or telecardio* or coronary or cardiopath* or dyslipaemi* or dyslipemi* or dyslipidaemi* or dyslipidemi* or hyperlipaemi* or hyperlipemi* or hyperlipidaemi* or hyperlipidemi* or hypercholester* or htn or hypertens* or prehyperten* or high-blood-pressure or arrhythmia* or dysrhythmia* or atrial-fibrillation or atrium-fibrillation or afib or a-fib or peripheral-arterial-disease* or peripheral-artery-disease* or peripheral-arterial-occlusive-disease* or peripheral-artery-occlusive-disease* or peripheral-arterial-obstructive-disease* or peripheral-artery-obstructive-disease* or athero-sclero* or atherosclero* or arteriosclero* or myocardial-infarct* or ischaemi* or ischemi* or blood-circulation-disorder* or blood-flow-disorder* or circulation-disorder* or circulatory-disorder* or circulatory-disturbance*).ti,ab.
6	4 or 5
7	awareness/ or "attitude of health personnel"/
8	"attitude of health personnel"/ or exp health personnel/ or exp administrative personnel/ or hospital administration/ or multi-institutional systems/ or organizational policy/
9	7 and 8
10	((accept* or access* or attitude* or availability or aware* or barrier* or challeng* or constraint* or deter or deterren* or disseminat* or difficult* or facilitat* or enable* or hinder* or hindrance* or hurdle* or

	imped* or implement* or interfere* or issue* or limit* or obstacle* or perceived-value or perception* or perspective* or problem* or promot* or restrict* or roadblock* or uptake or usab* or use-of or utility or utiliz*) adj6 (Administrator* or hospital-administration or healthcare-administration or health-care-administration or Cardiologist* or Care-worker* or Clinician* or Doctor* or Employee* or Health-aide* or Healthcare-team* or Health-care-team* or Healthcare-worker* or Health-care-worker* or Medical-assistant* or Nurs* or Paramedic* or Personnel or Pharmacist* or Physician-assistant* or physicians-assistant* or physician-s-assistant* or Physician* or Physiotherapist* or Practitioner* or Professional or professionals or Provider* or Psychologist* or psychotherapist* or Social-worker* or Specialist* or Staff or Worker*)).ti,ab.
11	9 or 10
12	3 and 6 and 11
13	12 not (case reports or editorial).pt.

Database: CINAHL (EBSCO) 1937 - present
Date searched: May 9, 2024
Results: 3659

Line	Query
1	MH "Digital Health" OR MH "Telehealth+" OR MH "Internet-Based Intervention" OR MH "Decision Making, Computer Assisted+") OR MH "Wearable Sensors+" OR MH "Cellular Phone" OR MH "Smartphone" OR MH "Mobile applications" OR MH "Computers, Hand-Held" OR MH "Internet" OR MH "Software" OR MH "Decision Support Systems, Clinical" OR MH "Patient Record Systems" OR MH "Electronic Health Records+" OR MH "Digital Technology"
2	TI (tele-cardiology OR telecardiology OR tele-care OR telecare OR tele-consult* OR teleconsult* OR tele-diagnos* OR telediagnos* OR tele-health* OR telehealth* OR tele-medic* OR telemedic* OR telemetr* OR tele-monitor* OR telemonitor* OR tele-nurs* OR telenurs* OR tele-psych* OR telepsych* OR tele-rehab* OR telerehab* OR tele-surveillance OR telesurveillance OR tele-therap* OR teletherap* OR telephone-consult* OR telephone-based-consult* OR clinical-decision-support-system* OR cds-system* OR computer-assisted OR computer-based OR computerized-medical OR computerised-medical OR computerized-patient OR computerised-patient OR computerized-clinical OR computerised-clinical OR computerized-decision-support OR computerised-decision-support OR automated-health OR automated-medical OR cyberconsult* OR cyber-consult* OR digital* OR e-care OR ecare OR electronic-consult* OR e-consult* OR econsult* OR e-health* OR ehealth* OR e-rehab* OR erehab* OR electronic-device* OR electronic-health* OR EHR OR EHRS OR electronic-medical OR EMR OR EMRS OR electronic-portal OR e-portal OR eportal OR hand-held-computer* OR handheld-computer* OR health-application* OR healthcare-application* OR health-care-app* OR health-technolog* OR healthcare-technolog* OR health-care-technolog* OR internet OR online OR web OR app OR apps OR mobile-application* OR mobile-health OR mhealth OR m-health OR mobile-phone* OR mobile-telephone* OR cell-phone* OR cellphone* OR cellular-phone* OR cellular-telephone* OR smart-phone* OR smartphone* OR smart-watch* OR wristband* OR fitness-tracker* OR mobile-technolog* OR personal-digital-assistant* OR remote* OR software OR tablet* OR virtual OR wearable*) OR AB (tele-cardiology OR telecardiology OR tele-care OR telecare OR tele-consult* OR teleconsult* OR tele-diagnos* OR telediagnos* OR tele-health* OR telehealth* OR tele-medic* OR telemedic* OR telemetr* OR tele-monitor* OR telemonitor* OR tele-nurs* OR telenurs* OR tele-psych* OR telepsych* OR tele-rehab* OR telerehab* OR tele-surveillance OR telesurveillance OR tele-therap* OR teletherap* OR telephone-consult* OR telephone-based-consult* OR clinical-decision-support-system* OR cds-system* OR computer-assisted OR computer-based OR computerized-medical OR computerised-medical OR computerized-patient OR

	computerised-patient OR computerized-clinical OR computerised-clinical OR computerized-decision-support OR computerised-decision-support OR automated-health OR automated-medical OR cyberconsult* OR cyber-consult* OR digital* OR e-care OR ecare OR electronic-consult* OR e-consult* OR econsult* OR e-health* OR ehealth* OR e-rehab* OR erehab* OR electronic-device* OR electronic-health* OR EHR OR EHRS OR electronic-medical OR EMR OR EMRS OR electronic-portal OR e-portal OR eportal OR hand-held-computer* OR handheld-computer* OR health-application* OR healthcare-application* OR health-care-app* OR health-technolog* OR healthcare-technolog* OR health-care-technolog* OR internet OR online OR web OR app OR apps OR mobile-application* OR mobile-health OR mhealth OR m-health OR mobile-phone* OR mobile-telephone* OR cell-phone* OR cellphone* OR cellular-phone* OR cellular-telephone* OR smart-phone* OR smartphone* OR smart-watch* OR wristband* OR fitness-tracker* OR mobile-technolog* OR personal-digital-assistant* OR remote* OR software OR tablet* OR virtual OR wearable*)
3	S1 OR S2
4	(MH "Cardiovascular Diseases+") OR (MH "Hyperlipidemia+")
5	TI (cardiovascular OR cvd OR heart OR cardiac OR cardio* OR telecardio* OR coronary OR cardiopath* OR dyslipaemi* OR dyslipemi* OR dyslipidaemi* OR dyslipidemi* OR hyperlipaemi* OR hyperlipemi* OR hyperlipidaemi* OR hyperlipidemi* OR hypercholester* OR htn OR hypertens* OR prehyperten* OR high-blood-pressure OR arrhythmia* OR dysrhythmia* OR atrial-fibrillation OR atrium-fibrillation OR afib OR a-fib OR peripheral-arterial-disease* OR peripheral-artery-disease* OR peripheral-arterial-occlusive-disease* OR peripheral-artery-occlusive-disease* OR peripheral-arterial-obstructive-disease* OR peripheral-artery-obstructive-disease* OR athero-sclero* OR atherosclero* OR arteriosclero* OR myocardial-infarct* OR ischaemi* OR ischemi* OR blood-circulation-disorder* OR blood-flow-disorder* OR circulation-disorder* OR circulatory-disorder* OR circulatory-disturbance*) OR AB (cardiovascular OR cvd OR heart OR cardiac OR cardio* OR telecardio* OR coronary OR cardiopath* OR dyslipaemi* OR dyslipemi* OR dyslipidaemi* OR dyslipidemi* OR hyperlipaemi* OR hyperlipemi* OR hyperlipidaemi* OR hyperlipidemi* OR hypercholester* OR htn OR hypertens* OR prehyperten* OR high-blood-pressure OR arrhythmia* OR dysrhythmia* OR atrial-fibrillation OR atrium-fibrillation OR afib OR a-fib OR peripheral-arterial-disease* OR peripheral-artery-disease* OR peripheral-arterial-occlusive-disease* OR peripheral-artery-occlusive-disease* OR peripheral-arterial-obstructive-disease* OR peripheral-artery-obstructive-disease* OR athero-sclero* OR atherosclero* OR arteriosclero* OR myocardial-infarct* OR ischaemi* OR ischemi* OR blood-circulation-disorder* OR blood-flow-disorder* OR circulation-disorder* OR circulatory-disorder* OR circulatory-disturbance*)
6	S4 OR S5
7	(MH "Attitude of Health Personnel+")
8	(MH "Attitude of Health Personnel+") OR (MH "Health Personnel+") OR (MH "Paramedics") OR (MH "Administrative Personnel") OR (MH "Health Facility Administrators") OR (MH "Nurse Administrators") OR (MH "Physician Executives")
9	S7 AND S8
10	TI ((accept* OR access* OR attitude* OR availability OR aware* OR barrier* OR challeng* OR constraint* OR deter OR deterren* OR disseminat* OR difficult* OR facilitat* OR enable* OR hinder* OR hindrance* OR hurdle* OR imped* OR implement* OR interfere* OR issue* OR limit* OR obstacle* OR perceived-value OR perception* OR perspective* OR problem* OR promot* OR restrict* OR roadblock* OR uptake OR usab* OR use-of OR utility OR utiliz*) N6 (Administrator* OR hospital-administration OR healthcare-administration OR health-care-administration OR Cardiologist* OR Care-worker* OR Clinician* OR Doctor* OR Employee* OR Health-aide* OR Healthcare-team* OR Health-care-team* OR Healthcare-worker* OR Health-care-worker* OR Medical-assistant* OR Nurs* OR Paramedic* OR Personnel OR Pharmacist* OR Physician-assistant* OR physicians-assistant* OR physician-s-assistant* OR Physician* OR Physiotherapist* OR Practitioner* OR Professional OR professionals OR Provider* OR Psychologist*

	OR psychotherapist* OR Social-worker* OR Specialist* OR Staff OR Worker*)) OR AB ((accept* OR access* OR attitude* OR availability OR aware* OR barrier* OR challeng* OR constraint* OR deter OR deterren* OR disseminat* OR difficult* OR facilitat* OR enable* OR hinder* OR hindrance* OR hurdle* OR imped* OR implement* OR interfere* OR issue* OR limit* OR obstacle* OR perceived-value OR perception* OR perspective* OR problem* OR promot* OR restrict* OR roadblock* OR uptake OR usab* OR use-of OR utility OR utiliz*) N6 (Administrator* OR hospital-administration OR healthcare-administration OR health-care-administration OR Cardiologist* OR Care-worker* OR Clinician* OR Doctor* OR Employee* OR Health-aide* OR Healthcare-team* OR Health-care-team* OR Healthcare-worker* OR Health-care-worker* OR Medical-assistant* OR Nurs* OR Paramedic* OR Personnel OR Pharmacist* OR Physician-assistant* OR physicians-assistant* OR physician-s-assistant* OR Physician* OR Physiotherapist* OR Practitioner* OR Professional OR professionals OR Provider* OR Psychologist* OR psychotherapist* OR Social-worker* OR Specialist* OR Staff OR Worker*))
11	S9 OR S10
12	S3 AND S6 AND S11
13	S12 NOT ((ZT "abstract") or (ZT "editorial") or (ZT "case study"))

Database: Embase (Elsevier) 1947 - present
Date searched: May 9, 2024
Results: 7550

Line	Query
1	'telehealth'/exp OR 'web-based intervention'/exp OR 'computer assisted therapy'/de OR 'wearable device'/de OR 'wearable sensor'/exp OR 'wearable computer'/exp OR 'mobile phone'/exp OR 'mobile application'/exp OR 'personal digital assistant'/exp OR 'internet'/de OR 'software'/de OR 'healthcare software'/exp OR 'clinical decision support system'/exp OR 'electronic health record'/de OR 'electronic medical record'/exp OR 'electronic medical record system'/exp OR 'digital technology'/exp
2	(tele-cardiology OR telecardiology OR tele-care OR telecare OR tele-consult* OR teleconsult* OR tele-diagnos* OR telediagnos* OR tele-health* OR telehealth* OR tele-medic* OR telemedic* OR telemetr* OR tele-monitor* OR telemonitor* OR tele-nurs* OR telenurs* OR tele-psych* OR telepsych* OR tele-rehab* OR telerehab* OR tele-surveillance OR telesurveillance OR tele-therap* OR teletherap* OR telephone-consult* OR telephone-based-consult* OR clinical-decision-support-system* OR cds-system* OR computer-assisted OR computer-based OR computerized-medical OR computerised-medical OR computerized-patient OR computerised-patient OR computerized-clinical OR computerised-clinical OR computerized-decision-support OR computerised-decision-support OR automated-health OR automated-medical OR cyberconsult* OR cyber-consult* OR digital* OR e-care OR ecare OR electronic-consult* OR e-consult* OR econsult* OR e-health* OR ehealth* OR e-rehab* OR erehab* OR electronic-device* OR electronic-health* OR EHR OR EHRS OR electronic-medical OR EMR OR EMRS OR electronic-portal OR e-portal OR eportal OR hand-held-computer* OR handheld-computer* OR health-application* OR healthcare-application* OR health-care-app* OR health-technolog* OR healthcare-technolog* OR health-care-technolog* OR internet OR online OR web OR app OR apps OR mobile-application* OR mobile-health OR mhealth OR m-health OR mobile-phone* OR mobile-telephone* OR cell-phone* OR cellphone* OR cellular-phone* OR cellular-telephone* OR smart-phone* OR smartphone* OR smart-watch* OR wristband* OR fitness-tracker* OR mobile-technolog* OR personal-digital-assistant* OR remote* OR software OR tablet* OR virtual OR wearable*):ti,ab
3	#1 OR #2

4	'cardiovascular disease'/exp OR 'dyslipidemia'/exp OR 'hyperlipidemia'/exp OR 'hypercholesterolemia'/exp
5	(cardiovascular OR cvd OR heart OR cardiac OR cardio* OR telecardio* OR coronary OR cardiopath* OR dyslipaemi* OR dyslipemi* OR dyslipidaemi* OR dyslipidemi* OR hyperlipaemi* OR hyperlipemi* OR hyperlipidaemi* OR hyperlipidemi* OR hypercholester* OR htn OR hypertens* OR prehyperten* OR high-blood-pressure OR arrhythmia* OR dysrhythmia* OR atrial-fibrillation OR atrium-fibrillation OR afib OR a-fib OR peripheral-arterial-disease* OR peripheral-artery-disease* OR peripheral-arterial-occlusive-disease* OR peripheral-artery-occlusive-disease* OR peripheral-arterial-obstructive-disease* OR peripheral-artery-obstructive-disease* OR athero-sclero* OR atherosclero* OR arteriosclero* OR myocardial-infarct* OR ischaemi* OR ischemi* OR blood-circulation-disorder* OR blood-flow-disorder* OR circulation-disorder* OR circulatory-disorder* OR circulatory-disturbance*):ti,ab
6	#4 OR #5
7	'awareness'/exp OR 'health personnel attitude'/de
8	'health personnel attitude'/de OR 'healthcare personnel'/exp OR 'administrative personnel'/de OR 'hospital administrator'/exp OR 'medical director'/exp OR 'medical receptionist'/exp OR 'medical secretary'/exp OR 'health care management'/de OR 'hospital management'/de OR 'hospital organization'/de OR 'multihospital system'/exp OR 'hospital policy'/exp
9	#7 AND #8
10	((accept* OR access* OR attitude* OR availability OR aware* OR barrier* OR challeng* OR constraint* OR deter OR deterren* OR disseminat* OR difficult* OR facilitat* OR enable* OR hinder* OR hindrance* OR hurdle* OR imped* OR implement* OR interfere* OR issue* OR limit* OR obstacle* OR perceived-value OR perception* OR perspective* OR problem* OR promot* OR restrict* OR roadblock* OR uptake OR usab* OR use-of OR utility OR utiliz*) NEAR/6 (Administrator* OR hospital-administration OR healthcare-administration OR health-care-administration OR Cardiologist* OR Care-worker* OR Clinician* OR Doctor* OR Employee* OR Health-aide* OR Healthcare-team* OR Health-care-team* OR Healthcare-worker* OR Health-care-worker* OR Medical-assistant* OR Nurs* OR Paramedic* OR Personnel OR Pharmacist* OR Physician-assistant* OR physicians-assistant* OR physician-s-assistant* OR Physician* OR Physiotherapist* OR Practitioner* OR Professional OR professionals OR Provider* OR Psychologist* OR psychotherapist* OR Social-worker* OR Specialist* OR Staff OR Worker*)):ti,ab
11	#9 OR #10
12	#3 AND #6 AND #11
13	#12 NOT ('case report'/de OR 'conference abstract'/it OR 'editorial'/it)

Database: Scopus (Elsevier) 1788 - present
Date searched: May 9, 2024
Results: 10685

Line	Query
1	TITLE-ABS-KEY ({tele cardiology} OR {telecardiology} OR {tele care} OR {telecare} OR {tele consult} OR {tele consultation} OR {tele consults} OR {tele consultations} OR {teleconsult} OR {teleconsultation} OR {teleconsults} OR {teleconsultations} OR {tele diagnosis} OR {telediagnosis} OR {telediagnostic} OR {tele diagnostic} OR {tele health} OR {telehealth} OR {tele medicine} OR {telemedicine} OR {telemetry} OR {tele monitoring} OR {telemonitoring} OR {tele nursing} OR {telenursing} OR {tele psychology} OR {telepsychology} OR {telepsychiatry} OR {tele psychiatry} OR {tele rehab} OR {telerehab} OR {tele

	rehabilitation} OR {telerehabilitation} OR {tele surveillance} OR {telesurveillance} OR {tele therapy} OR {teletherapy} OR {telephone consult} OR {telephone consults} OR {telephone consultation} OR {telephone consultations} OR {telephone based consult} OR {telephone based consults} OR {telephone based consultation} OR {telephone based consultations} OR {clinical decision support system} OR {clinical decision support systems} OR {cds system} OR {cds systems} OR {computer assisted} OR {computer based} OR {computerized medical} OR {computerised medical} OR {computerized patient} OR {computerised patient} OR {computerized clinical} OR {computerised clinical} OR {computerized decision support} OR {computerised decision support} OR {automated health} OR {automated medical} OR {cyberconsult} OR {cyberconsults} OR {cyberconsultation} OR {cyberconsultations} OR {cyber consult} OR {cyber consults} OR {cyber consultation} OR {cyber consultations} OR {digital} OR {digitally} OR {e care} OR {ecare} OR {electronic consult} OR {electronic consults} OR {electronic consultation} OR {electronic consultations} OR {e consult} OR {e consults} OR {e consultation} OR {e consultations} OR {econsult} OR {econsults} OR {econsultation} OR {econsultations} OR {e health} OR {ehealth} OR {e rehab} OR {e rehabilitation} OR {erehab} OR {erehabilitation} OR {electronic device} OR {electronic devices} OR {electronic health} OR {EHR} OR {EHRS} OR {electronic medical} OR {EMR} OR {EMRS} OR {electronic portal} OR {e portal} OR {eportal} OR {hand held computer} OR {hand held computers} OR {handheld computer} OR {handheld computers} OR {health application} OR {health applications} OR {healthcare application} OR {healthcare applications} OR {health care app} OR {healthcare app} OR {health care apps} OR {healthcare apps} OR {health technology} OR {health technologies} OR {healthcare technology} OR {healthcare technologies} OR {health care technology} OR {health care technologies} OR {internet} OR {online} OR {web} OR {app} OR {apps} OR {mobile application} OR {mobile applications} OR {mobile health} OR {mhealth} OR {m health} OR {mobile phone} OR {mobile phones} OR {mobile telephone} OR {mobile telephones} OR {cell phone} OR {cell phones} OR {cellphone} OR {cellphones} OR {cellular phone} OR {cellular phones} OR {cellular telephone} OR {cellular telephones} OR {smart phone} OR {smart phones} OR {smartphone} OR {smartphones} OR {smart watch} OR {smart watches} OR {wristband} OR {wristbands} OR {fitness tracker} OR {fitness trackers} OR {mobile technology} OR {mobile technologies} OR {personal digital assistant} OR {personal digital assistants} OR {remote} OR {remotely} OR {software} OR {tablet} OR {tablets} OR {virtual} OR {wearable} OR {wearables})
2	TITLE-ABS-KEY({cardiovascular} OR {cvd} OR {heart} OR {cardiac} OR "cardio*" OR {telecardiology} OR {coronary} OR {cardiopathy} OR {cardiopathies} OR {dyslipaemia} OR {dyslipaemic} OR {dyslipemia} OR {dyslipemic} OR {dyslipidaemia} OR {dyslipidaemic} OR {dyslipidemia} OR {dyslipidemic} OR {hyperlipaemia} OR {hyperlipaemic} OR {hyperlipemia} OR {hyperlipidemic} OR {hyperlipidaemia} OR {hyperlipidaemic} OR {hyperlipidemia} OR {hyperlipidemic} OR "hypercholester*" OR {htn} OR {hypertension} OR {hypertensive} OR {prehypertenion} OR {prehypertenive} OR {high blood pressure} OR {arrhythmia} OR {arrhythmias} OR {dysrhythmia} OR {dysrhythmias} OR {atrial fibrillation} OR {atrium fibrillation} OR {afib} OR {a fib} OR {peripheral arterial disease} OR {peripheral arterial diseases} OR {peripheral artery disease} OR {peripheral artery diseases} OR {peripheral arterial occlusive disease} OR {peripheral arterial occlusive diseases} OR {peripheral artery occlusive disease} OR {peripheral artery occlusive diseases} OR {peripheral arterial obstructive disease} OR {peripheral arterial obstructive diseases} OR {peripheral artery obstructive disease} OR {peripheral artery obstructive diseases} OR {athero sclerosis} OR {athero sclerotic} OR {atherosclerosis} OR {atherosclerotic} OR {arteriosclerosis} OR {arteriosclerotic} OR {myocardial infarct} OR {myocardial infarcts} OR {myocardial infarction} OR {myocardial infarctions} OR {ischaemia} OR {ischaemic} OR {ischemia} OR {ischemic} OR {blood circulation disorder} OR {blood circulation disorders} OR {blood flow disorder} OR {blood flow disorders} OR {circulation disorder} OR {circulation disorders} OR {circulatory disorder} OR {circulatory disorders} OR {circulatory disturbance} OR {circulatory disturbances})
3	TITLE-ABS-KEY((accept* OR access* OR attitude* OR availability OR aware* OR barrier* OR challeng* OR constraint* OR deter OR deterren* OR disseminat* OR difficult* OR facilitat* OR enable* OR hinder* OR hindrance* OR hurdle* OR imped* OR implement* OR interfere* OR issue* OR limit* OR obstacle* OR perceived-value OR perception* OR perspective* OR problem* OR promot* OR restrict* OR roadblock* OR uptake OR usab* OR use-of OR utility OR utiliz*) W/6 (Administrator* OR hospital-administration OR

	healthcare-administration OR health-care-administration OR Cardiologist* OR Care-worker* OR Clinician* OR Doctor* OR Employee* OR Health-aide* OR Healthcare-team* OR Health-care-team* OR Healthcare-worker* OR Health-care-worker* OR Medical-assistant* OR Nurs* OR Paramedic* OR Personnel OR Pharmacist* OR Physician-assistant* OR physicians-assistant* OR physician-s-assistant* OR Physician* OR Physiotherapist* OR Practitioner* OR Professional OR professionals OR Provider* OR Psychologist* OR psychotherapist* OR Social-worker* OR Specialist* OR Staff OR Worker*))
4	DOCTYPE(ed) OR KEY("Case Report")
5	#1 AND #2 AND #3 AND NOT #4

Database: Web of Science (Clarivate)
Editions: Science Citation Index Expanded (SCI-EXPANDED)--1900-present,
Social Sciences Citation Index (SSCI)--1900-present,
Arts & Humanities Citation Index (AHCI)--1975-present,
Conference Proceedings Citation Index – Science (CPCI-S)--1990-present,
Conference Proceedings Citation Index – Social Science & Humanities (CPCI-SSH)--1990-present,
Book Citation Index – Science (BKCI-S)--2005-present,
Book Citation Index – Social Sciences & Humanities (BKCI-SSH)--2005-present,
Emerging Sources Citation Index (ESCI)--2005-present,
Current Chemical Reactions (CCR-EXPANDED)--1985-present,
Index Chemicus (IC)--1993-present
Date searched: May 9, 2024
Results: 5960

Line	Query
1	TS = (tele-cardiology OR telecardiology OR tele-care OR telecare OR tele-consult* OR teleconsult* OR tele-diagnos* OR telediagnos* OR tele-health* OR telehealth* OR tele-medic* OR telemedic* OR telemetr* OR tele-monitor* OR telemonitor* OR tele-nurs* OR telenurs* OR tele-psych* OR telepsych* OR tele-rehab* OR telerehab* OR tele-surveillance OR telesurveillance OR tele-therap* OR teletherap*)
2	TS = (cardiovascular OR cvd OR heart OR cardiac OR cardio* OR telecardio* OR coronary OR cardiopath* OR dyslipaemi* OR dyslipemi* OR dyslipidaemi* OR dyslipidemi* OR hyperlipaemi* OR hyperlipemi* OR hyperlipidaemi* OR hyperlipidemi* OR hypercholester* OR htn OR hypertens* OR prehyperten* OR high-blood-pressure OR arrhythmia* OR dysrhythmia* OR atrial-fibrillation OR atrium-fibrillation OR afib OR a-fib OR peripheral-arterial-disease* OR peripheral-artery-disease* OR peripheral-arterial-occlusive-disease* OR peripheral-artery-occlusive-disease* OR peripheral-arterial-obstructive-disease* OR peripheral-artery-obstructive-disease* OR athero-sclero* OR atherosclero* OR arteriosclero* OR myocardial-infarct* OR ischaemi* OR ischemi* OR blood-circulation-disorder* OR blood-flow-disorder* OR circulation-disorder* OR circulatory-disorder* OR circulatory-disturbance*)
3	TS = ((accept* OR access* OR attitude* OR availability OR aware* OR barrier* OR challeng* OR constraint* OR deter OR deterren* OR disseminat* OR difficult* OR facilitat* OR enable* OR hinder* OR hindrance* OR hurdle* OR imped* OR implement* OR interfere* OR issue* OR limit* OR obstacle* OR perceived-value OR perception* OR perspective* OR problem* OR promot* OR restrict* OR roadblock* OR uptake OR usab* OR use-of OR utility OR utiliz*) NEAR/6 (Administrator* OR hospital-administration OR healthcare-administration OR health-care-administration OR Cardiologist* OR Care-worker* OR Clinician* OR Doctor* OR Employee* OR Health-aide* OR Healthcare-team* OR Health-care-team* OR Healthcare-worker* OR Health-care-worker* OR Medical-assistant* OR Nurs* OR Paramedic* OR Personnel OR Pharmacist* OR Physician-assistant* OR physicians-assistant* OR physician-s-assistant* OR

	Physician* OR Physiotherapist* OR Practitioner* OR Professional OR professionals OR Provider* OR Psychologist* OR psychotherapist* OR Social-worker* OR Specialist* OR Staff OR Worker*))
4	#1 AND #2 AND #3
5	#4 NOT DT=(Meeting Abstract OR Editorial Material)

Database: ACM Digital Library, 1908 – present
Date searched: May 9, 2024
Results: 484

Query
(Title:(cardiovascular OR cvd OR heart OR cardiac OR cardiology OR coronary OR cardiopathy) AND (telehealth OR telemedicine OR digital OR technology OR computer OR electronic OR internet OR online OR software OR app OR "mobile application") OR Abstract:(cardiovascular OR cvd OR heart OR cardiac OR cardiology OR coronary OR cardiopathy)) AND (Title:(telehealth OR telemedicine OR digital OR technology OR computer OR electronic OR internet OR online OR software OR app OR "mobile application") OR Abstract:(telehealth OR telemedicine OR digital OR technology OR computer OR electronic OR internet OR online OR software OR app OR "mobile application")) AND (Title:(barrier OR challenge OR constraint OR deterrent OR facilitator OR obstacle OR uptake) OR Abstract:(barrier OR challenge OR constraint OR deterrant OR facilitator OR obstacle OR uptake)) AND (Title:(clinician OR doctor OR physician OR staff OR employee OR healthcare OR "health care" OR worker OR practitioner OR provider OR nurse OR therapist OR professional) OR Abstract:(clinician OR doctor OR physician OR staff OR employee OR healthcare OR "health care" OR worker OR practitioner OR provider OR nurse OR therapist OR professional))

Database: IEEE Xplore, 1893 – present
Date searched: May 9, 2024
Results: 644
Note: Used command line search

Query
("Document Title":cardiovascular OR "Document Title":cvd OR "Document Title":heart OR "Document Title":cardiac OR "Document Title":cardio* OR "Document Title":coronary OR "Document Title":telecardio* OR "Abstract":cardiovascular OR "Abstract":cvd OR "Abstract":heart OR "Abstract":cardiac OR "Abstract":cardio* OR "Abstract":coronary OR "Abstract":telecardio*) AND ("Document Title":telehealth OR "Document Title":telemedicine OR "Document Title":telecardio* OR "Document Title":digital OR "Document Title":technology OR "Document Title":computer OR "Document Title":electronic OR "Document Title":internet OR "Document Title":online OR "Document Title":software OR "Document Title":app OR "Document Title":"mobile app*" OR "Abstract":telehealth OR "Abstract":telemedicine OR "Abstract":telecardio* OR "Abstract":digital OR "Abstract":technology OR "Abstract":computer OR "Abstract":electronic OR "Abstract":internet OR "Abstract":online OR "Abstract":software OR "Abstract":app OR "Abstract":"mobile app*") AND ("Document Title":barrier OR "Document Title":challenge OR "Document Title":constraint OR "Document Title":deterrent OR "Document Title":deter OR "Document Title":facilitator OR "Document Title":facilitate OR "Document Title":obstacle OR "Document Title":uptake OR "Abstract":barrier OR "Abstract":challenge OR "Abstract":constraint OR "Abstract":deterrent OR "Abstract":deter OR "Abstract":facilitator OR

"Abstract":facilitate OR "Abstract":obstacle OR "Abstract":uptake) AND ("Document Title":clinician OR "Document Title":doctor OR "Document Title":physician OR "Document Title":staff OR "Document Title":employee OR "Document Title":healthcare OR "Document Title":"health care" OR "Document Title":worker OR "Document Title":practitioner OR "Document Title":provider OR "Document Title":nurse OR "Document Title":therapist OR "Document Title":professional OR "Abstract":clinician OR "Abstract":doctor OR "Abstract":physician OR "Abstract":staff OR "Abstract":employee OR "Abstract":healthcare OR "Abstract":"health care" OR "Abstract":worker OR "Abstract":practitioner OR "Abstract":provider OR "Abstract":nurse OR "Abstract":therapist OR "Abstract":professional)

Source: Google Scholar, via Publish or Perish software
Date searched: May 9, 2024
Results note: Exported first 200 records

Query
(telehealth|telemedicine|digital|technology|computer|electronic|internet|online|software|app|application)(cardiovascular|cvd|heart|cardiac|cardiology|coronary|cardiopathy)(barrier|challenge|constraint|deterrent|facilitator|obstacle|uptake)

S2

**Article ID**	**Title of Manuscript**
107 - Collateral search	Barriers and facilitators to the implementatio
17	Healthcare Providers' Experience Using Telehealth During the Onset of the COVID-19
100	Clinicians' Perspectives in Using Patient-Gene

64	Perceived barriers to effective use of teleheal

30	Identification of the Most Suitable App to Sup

31	Patients and Professionals as Partners in Hype
1	Patients' and providers' perspectives on and n

32	Experiences and Perceptions of Patients and P
65	Smartphone-Based Remote Monitoring for Ch

108- Collateral search	Critical appraisal of a mHealth-assisted comm
2	Patient and Health Professional Perceptions o

50	Optimizing Telehealth for Diabetes Managem[illegible]

51	Evaluation of a Clinical Decision Support Tool [illegible]
92	Clinical managers' identity at the crossroad of

33	Acceptability of a mHealth strategy for hypert
25- Collateral search	Consumer-led screening for atrial fibrillation u
52	Cardiology professionals’ views of social robot

1	Comparing the Acceptance of Mobile Hyperte

53	What works and why in the implementation o
110- Collateral search	Description, utilisation and results from a tele

34	Implementation outcomes of Humanwide:inte
22	Understanding the Feasibility, Acceptability, a

22	Perspectives on the Use of Digital Health Tech

66	A mobile health application for patients eligib

3	Telehealth is here to stay but not without cha

93	Understanding the Attitudes of Clinicians and
67	Trust and Uncertainty in the Implementation o

68	Implementing patient decision aids into gener

11	Pragmatic Study of Clinican Use of a Personali
69	Healthcare professionals’ perceptions of using

35	Nurses’ and Physicians’ Perspectives on Text-B
8	Survey of current perspectives on consumer-a

70	Implementation and evaluation of an electron

71	Automated Messaging Program to Facilitate S

47	Clinician Perspectives on the Design and Appli
54	Virtual care during COVID-19: The perspective

36	Changes in patient activation following cardia[illegible]
55	“Hypertension is such a difficult disease to ma[illegible]

4	Use of mobile apps in heart failure self-manag

48	Interdisciplinary Mobile Health Model to Impr

96	Physician perceived barriers and facilitators fo

2	Normalizing Telemonitoring in Nurse-led Care

72	Exploring the Incorporation of a Novel Cardiot
73	Patient–clinician dynamics in remote consulta

74	Implementing a home-based virtual hyperten[illegible]
26	Telemonitoring at scale for hypertension in pr[illegible]

6	Development and user-testing of a digital pati

7	Challenges in managing a remote monitoring

75	Screening of Atrial Fibrillation in Dental Practic

76	Adoption of shared decision-making and clinic

56	Determinants of Bluetooth-Enabled Self-Meas

9	Users’ acceptance of electronic patient portal
94	Acceptability of a computer-tailored and pedo

8	Process Evaluation of a Randomised Controlle

9	Healthcare providers' perspectives on using sr

97	How digital health affects the patient-physicia

24	The attitude of primary care physicians to the

7	On the topic of demand and usage of the tele
20	Moblie health in cardiac patients: an overview

10	Changing to remote management of a commu
95	Increasing cardiovascular medication adheren
77	Qualitative analysis of a remote monitoring in

4	Implementation of a personal health planning
10	Implementation of an intensive telehealth int[illegible]
37	Informing telehealth service delivery for cardi[illegible]
98	Cardiological teleconsultation in the coronavi[illegible]
11	Reaching Frail Elderly Patients to Optimize Di[illegible]

78	Informing Use of Telehealth for Managing Chr

19	Nurses’ willingness to resort to telemonitoring
89	Clinical Perspectives on the Development of a

79	Adoption of Electronic Medical Records for Ch
109- Collateral search	Facilitators and barriers to optimal home bloo

12	Teleconsultations and their implications for he

99	Cardiac Rehabilitation in Canada During COVID
38	Clinician Preimplementation Perspectives of a

90	Implementing a Clinical Decision Support Tool

27	Postoperative Remote Automated Monitoring

57	Self-Measured Blood Pressure Monitoring Du
29	Cardiologists' Perception of Wearable Device
12	Implementing a digital intervention for manag

15	Acceptance of Telemedicine by Specialists and
40	Engaging primary care providers in a mobile h
5	Quality of virtual care for ambulatory care ser

41	Lessons learned from Integrated Managemen

80	Perceived health system facilitators and barri[illegible]
103	Digital transformation in the healthcare secto[illegible]

81	Digital Health Secondary Prevention Using Co-

6	Blood pressure telemonioring and telemedicir

49	“I Like the Idea of It…But Probably Wouldn’t U

16	Digital health and cardiovascular healthcare p
58	Tailoring remote patient management in card

59	Investigating barriers & facilitators for the suc

42	Facilitators of and Barriers to Lifestyle Suppor

14	Utilizing telehealth intervention to support pa

13	An Evaluation of Cardiology Virtual Care Durin
25	Patients, clinicians, and researchers working t

82	Feasibility of a Virtual Educational Prograr
104 - Collateral search	Pharmacists detecting atrial fibrillation in gene

20	Artificial intelligence–enabled tools in cardiov[illegible]

91	Physiotherapists' Adoption and Perceptions of
44	Personalized, Electronic Health Record–Integr

60	Artificial intelligence predictive analytics in he
15	Collaborative Quality Improvement Strategy i

84	Benefits and challenges of telemedicine for he

16	Evaluating Health Care Provider Perspectives [illegible]

24	The importance of ICT and Wearable Devices

106 - Collateral search	Facilitators and Barriers to Chronic Self-Manag
28	Can mHealth and eHealth improve manageme

61	Implementing a Sodium-Glucose Cotransporte

85	Implementation and use of technology-enable

86	Sustaining telehealth among cardiac and pulm

17	Perspectives on the participation in a feasibilit
18	Qualitative study of the views of people living

62	Feasibility of early digital health rehabilitation

45	Status of Medical Care and Management Requ
19	Patient and practitioner views on a combined

21	Leveraging User Experience to Improve Video
105 - Collateral search	Mother, an mHealth System to Support Wom
102	Clinican Attitudes Towards Telemonitoring for

101	COVID-19 telehealth preparedness: a cross-se

46	Expanding Telemonitoring in a Virtual World:A

87	Exploring the use of a digital therapeutic inter

63	Managing Medications During “Sick Days” in P

21	Factors affecting wearable ECG device adoptic
88	Translating and piloting a cardiovascular risk a

DOI Number	First author last name	Publishing year
10.1186/s12913-019-4774-x	Adler	2020
10.7759/cureus.36260	Al Hemyari	2023
rated Health Data to Improve Ischer	Alaboud	2022

10.1186/s12913-023-09368-w.	Alarabyat	2023

10.2196/29207	Alessa	2021

10.2196/26143	Andersson	2021
10.2196/32545	Aquino	2022

10.2196/28259	Artanian	2021
10.2196/44630	Auton	2023

10.1080/20477724.2020.1816286	Aw	2020
/10.2196/32874	Baratta	2022

10.2196/43583	Bazzano	2024

10.1161/JAHA.123.031523	Bergstedt	2024
10.1111/isj.12267	Bernardi	2020

10.1136/bmjopen-2021-052986	Bhandari	2021
10.1016/j.ejim.2020.09.005	Boriani	2020
10.1093/ehjdh/ztad067	Bouchard	2024

10.2196/31617	Breil	2022

10.1080/17483107.2022.2088867	Brouns	2024
10.1177/1357633X18789562	Brown	2020

10.1186/s12875-021-01373-4	Brown-Johnson	2021
http://dx.doi.org/10.2196/19882	Buis	2020

10.5543/tkda.2023.13660	Burunkaya	2024

10.1186/s12911-023-02221-4	Cao	2023

10.1093/eurjcn/zvab118	Cartledge	2022

10.2196/15492	Cher	2020
10.2196/36072	Chew	2023

10.1016/j.pecinn.2023.100140	Cornell	2023

10.1089/tmj.2019.0112	Coylewright	2020
10.1186/s12913-023-09997-1	Danielsbacka	2023

https://doi.org/10.1016/j.nurpra.2	Demers	2021
10.1016/j.cvdhj.2020.06.002	Ding	2020

10.1093/jamiaopen/ooad098.	Dullabh	2023

10.2196/51316	Einhorn	2023

10.2196/17299	Ferguson	2020
10.1017/S0714980824000102	Flores-Sandoval	2024

https://doi.org/10.1186/s12913-02	Frith	2021
10.1186/s43058-024-00587-8	Gago	2024

10.2196/33992	Giordan	2022

http://cardio.jmir.org/2020/1/e190	Gomis-Pastor	2020

10.1371/journal.pone.0255578	Gondi	2021

10.2196/36346	Gordon	2022

10.2196/46481.	Gregory	2023
10.1136/bmjopen-2022-070923	Grey	2023

10.1093/fampra/cmac084.	Gupta	2023
10.1371/journal.pmed.1003124	Hammersley	2020

10.1186/s12911-022-01882-x	Harris	2022

10.1016/j.hroo.2021.12.002	Harvey	2022

10.1080/14779072.2023.22546	Hassan	2023

10.1093/jamiaopen/ooad012.	Hauschildt	2023

10.1101/2023.08.17.23294249	Hellem	2024

10.1186/s12911-020-1047-x	Honein-AbouHaidar	2020
10.1177/2055207619899840	Houle	2020

10.3389/fmed.2021.780882	Indraratna	2022

doi.org/10.1016/j.ijmedinf.2022.10	Islam	2022

https://doi.org/10.1016/j.preghy.2	Jongsma	2021

10.17116/profmed2020230628	Kalinina 2020	2020

10.17116/profmed20212406128	Kalinina 2021	2021
10.1136/bmjinnov-2019-000418	Kauw	2020

10.1016/j.ijcha.2020.100665	Kerr	2020
10.1111/jan.14465	Khonsari	2020
10.1186/s12872-023-03456-9.	Klaiman	2023

10.1016/j.heliyon.2022.e09900	Kleiner	2022
10.1007/s11606-021-07281-8	Kobe	2022
10.1071/AH19231	Kocanda	2021
10.33963/KP.15737	Kołtowski	2021
10.3390/ijerph19127383	Ladegaard	2022

10.1161/JAHA.122.027362	Leonard	2023

10.17665/1676-4285.20236648	Lino	2023
10.1097/CIN.0000000000000983	Lukey	2023

10.2196/43878.	Lyles	2023
10.1186/s12913-023-09976-6	Mahmoud	2023

10.1016/j.ijmedinf.2022.104751	Maria	2022

https://doi.org/10.1016/j.cjco.2020	Marzolini	2021
10.2196/26964	Matthiesen	2021

10.1097/NNR.0000000000000714	McCarthy	2024

http://dx.doi.org/10.2196/15548	McGillion	2020

10.1097/JHQ.0000000000000417	Meador	2024
10.1007/s11606-019-05390-z	Mehta	2020
10.1186/s13012-021-01123-1	Morton	2021

10.2196/49526	Muehlensiepen	2024
10.1177/20552076211066746	Nau	2021
10.2196/35599	Nayyar	2022

https://doi.org/10.1186/s13063-02	Nemis-White	2021

10.1136/bmjopen-2023-074274	Otieno	2023
10.1007/978-3-030-30911-4_31	Palozzi	2020

10.2196/49892.	Pelly	2023

10.1080/08037051.2022.2123781.	Piskorz	2022

10.2196/18101	Portz	2020

10.1016/j.repc.2023.10.014	Queiroz	2024
10.1186/s12875-024-02355-y	Rakers	2024

10.1371/journal.pone.0298898	Riboli-Sasco	2024

10.2196/25646	Rodrigues	2021

10.1016/j.apnr.2022.151641	Saifan	2022

https://doi.org/10.1016/j.cjco.202	Sanderson	2021
10.1136/bmjopen-2019-031187	Santana	2020

10.3390/ijerph20115934.	Santos	2023
10.3399/bjgpopen20X101042	Savickas	2020

10.1016/j.cvdhj.2023.04.003.	Schepart	2023

https://doi.org/10.3138/ptc-2021-(	Schertzer	2024
10.1161/CIRCOUTCOMES.120.007:	Schott	2021

10.1093/jamia/ocae017	Sideris	2024
10.5334/gh.1161	Singh	2022

10.1186/s12913-023-09872-z.	Singhal	2023

10.2196/40546	Sivakumar	2022

https://doi.org/10.1007/978-3-030	Sobreiro	2020

2020/4/e13536/	Steinman	2020
10.21037/mhealth-19-249	Steinman2	2020

10.2196/50737	Suen	2024

10.1186/s12875-023-02014-8.	Teo	2023

10.1093/eurjcn/zvac111	Thomas	2023

10.2196/35365	Thorup	2022
10.1136/bmjopen-2021-056768	Tighe	2022

10.1186/s12913-024-10601-3	Toft	2024

10.1253/circrep.CR-20-0132	Tsukada	2021
10.1136/bmjopen-2021-053183	Van Emmenis	2022

10.2196/19771	Vandekerckhove	2020
10.1089/dia.2020.0509	Varnfield	2021
10.1145/3544549.3585723	Vugs	2023

10.2217/pme-2021-0179	Waldman	2022

http://www.jmir.org/2021/1/e261	Wali 2021	2021

10.1371/journal.pdig.0000371	Wali 2023	2023

10.1016/j.jcjd.2024.02.003	Watson	2024

10.3389/fpubh.2023.1128127	Yao	2023
10.1016/j.jsps.2023.02.001.	Zolezzi	2023

Description of study
**Country(ies) where the study was conducted**
Ghana
10 southern Kentucky counties (where approximately 61% of this populatior
United States

Jordan

Saudi Arabia

Sweden
Canada

Canada
London, United Kingdom

Kenya
United States

United States

United States
England

Nepal
(482 HCPs provided their geographical region) Europe (77.4%), Asia/Oceania
Canada

Germany

Netherlands
United States

United States of America
United States

Turkey

Ohio, US

Australia

Singapore
central Singapore

Australia (general practice consultations)

United States
Region Västra Götaland, Sweden

United States of America
N/A 77 countries (but 50.3% responses from the United States)

Bridgeport, Connecticut

urban and suburban practices in the Pittsburgh metropolitan area

Australia
Canada

United Kingdom
United States

Australia

Spain

United States

Canada

US
rheumatology and cardiology outpatient departments of a general hospital i

University of Kansas Medical Center, Kansas City, United States
United Kingdom

United Kingdom

United States (n=40), United Kingdom (n=3), Australia (n=1), Egypt (n=1)

North-West of England

US

United States

Lebanon
Canada

Australia

Australia

Netherlands

Russian federation

Russian Federation
The Netherlands

Ireland
Iran
Philadelphia hospitals in the University of Pennsylvania Health System

Hungary
United States
Australia
Poland
Denmark

US (6 Veterans Health Administration Medical Centers with high telehealth [illegible]

municipalities from the 15th Health Region of Paraná
Canada

Kenya’s Hagadera refugee camp
Nigeria

Portugal

Canada
Denmark

United States

Canada and the United Kingdom

United States
United States
United Kingdom

Germany
Australia
Canada

Canada

Kenya (12 counties)
Italy

Victoria, Australia

[illegible]<br>Brazil (10.9%, n=191), Venezuela (8.7%, n=153), Guatemala (8.2%,n =144),

United States of America

Portugal
Netherlands

England

Netherlands

Jordan

Canada
Canada

south of Brazil (Cardio- Oncology and Exercise Medicine Center, Florianopoli
United Kingdom

Kennett Square, PA (US)

Canada
United States of America

United States
India

South of England (UK)

Canada

Portugal

Cambodia
Cambodia

Australia

Singapore

Queensland, Australia

Denmark
Australia

Denmark

Japan
United Kingdom

Belgium
Australia
Netherlands

United States

Canada

Toronto, Canada

Canada

Sichuan, China
Qatar

**Classification of country income (https://blogs.worldbank**
lower-middle
high
high

lower-middle

high

high
high

high
high

lower-middle
high

high

high
high

lower-middle

(13.7%), North or South America (8.3%), and 3 (0.6%) in oth

high

high

high
high

high
high

upper-middle

high

high

high
high

high

high
high

high

high

high

high
high

high
high

high

high

high

high

high
high

high
high

high

high

high

high

high

lower-middle
high

high

high

high

high

high
high

high
upper-middle
high

high
high
high
high
high

high

upper-middle
high

lower-middle
lower-middle

high

high
high

high

high

high
high
high

high
high
high

high

lower-middle
high

high

upper-middle

high

high
high

high

high

lower-middle

high
high

upper-middle
high

high

high
high

high
lower-middle

high

high

high

lower-middle
lower-middle

high

high

high

high
high

high

high
high

high
high
high

high

high

high

high

upper-middle
high

WHO classification of regions (https://www.who.int/	Study aim(s)	Study design
African region (AFR)	This paper presents results of an assessment of the six	Qualitative - interview stu
Region of the Americas (AMR)	The goal of this article was to	Quantitative - observation
Region of the Americas (AMR)	The main objective of the st	Qualitative - interview stu

Eastern Mediterranean region (EMR)	The study aims to explore th	Qualitative - interview stu

Eastern Mediterranean region (EMR)	The aim of this study is thus	Qualitative - interview stu

European region (EUR)	The objectives of this study	Qualitative - focus group s
Region of the Americas (AMR)	(1) What are the needs and	Qualitative - interview stu

Region of the Americas (AMR)	The purpose of this paper is	Qualitative - interview stu
European region (EUR)	This study aims to evalua	Other

African region (AFR)	In this operational research	Qualitative - interview stu
Region of the Americas (AMR)	To formally assess the imple	Qualitative - interview stu

Region of the Americas (AMR)	To explore barriers, facilitat	Qualitative - interview stu

Region of the Americas (AMR)	To evaluate physician telem	Mixed methods
European region (EUR)	To investigate how clinical [illegible]	Mixed methods

South-East Asia region (SEAR)	This formative study aimed	Qualitative - interview stu
ner regions	A variety of consumer-facin	Quantitative - observation
Region of the Americas (AMR)	To elicit the views of health	Qualitative - interview stu

European region (EUR)	The present study aims to investigate the	Quantitative - observation

European region (EUR)	To understand what worked	Mixed methods
Region of the Americas (AMR)	[illegible] weight management	Qualitative- survey

Region of the Americas (AMR)	We assessed implementatio	Qualitative - interview stu
Region of the Americas (AMR)	The goal of this study was to investigate the	Qualitative - interview stu

European region (EUR)	In this study, they aimed to	Quantitative - observation

Region of the Americas (AMR)	to explore healthcare provic	Qualitative - interview stu

Western pacific region (WPR)	We aimed to explore (i) clinicians’ experiences and	Qualitative - focus group s

South-East Asia region (SEAR)	To explore the perceptions	Qualitative - interview stu
Western pacific region (WPR)	This study examined the pe	Other

Western pacific region (WPR)	aimed to test the feasibilit	Other

Region of the Americas (AMR)	This study examines use of a	Qualitative- survey
European region (EUR)	the aim of the study was t	Qualitative - interview stu

Region of the Americas (AMR)	Thus, this study was conduc	Qualitative - interview stu
	[illegible] (healthcare practitioners)	Quantitative - observation

Region of the Americas (AMR)	We had 3 evaluation goals:	Qualitative - interview stu

Region of the Americas (AMR)	goal of the current study wa	Qualitative - interview stu

Western pacific region (WPR)	This study aimed to explore	Qualitative - focus group s
Region of the Americas (AMR)	To explore the perceptions	Qualitative - interview stu

European region (EUR)	he primary aim of this coho	Qualitative - interview stu
Region of the Americas (AMR)	To identify facilitators and b	Mixed methods

Western pacific region (WPR)	This study aims to explore the perspectives of	Qualitative - interview stu

European region (EUR)	The main objectives of this s	Qualitative- survey (open-

Region of the Americas (AMR)	The purpose of this study was to describe the	Qualitative - interview stu

Region of the Americas (AMR)	The objective of this study i	Qualitative - interview stu

Region of the Americas (AMR)	sought to determine consid	Qualitative - interview stu
European region (EUR)	explore the experiences of [illegible]	Qualitative - interview stu

Region of the Americas (AMR)	assess feasibility, acceptabil	Mixed methods
European region (EUR)	To explore the feasibility an	Qualitative - interview stu

European region (EUR)	This study aimed to develop	Qualitative - interview stu

Region of the Americas (AMR)	The purpose of this research was to survey	Mixed methods

European region (EUR)	explore the perceptions of c	Qualitative - interview stu

Region of the Americas (AMR)	This study explored the circ	Mixed methods

Region of the Americas (AMR)	To understand the experien	Qualitative - interview stu

Eastern Mediterranean region (EMR)	This study aims to use the T	Quantitative - observation
Region of the Americas (AMR)	To identify factors influenci	Qualitative - focus group s

Western pacific region (WPR)	[illegible]<br>this paper aims to identify	Qualitative - interview stu

Western pacific region (WPR)	Therefore, this study aimed	Qualitative - interview stu

European region (EUR)	[illegible] qualitative interview study	Qualitative - interview stu

European region (EUR)	The aim of this study is to e:	Quantitative - observation

European region (EUR)	To conduct a survey of prim	Quantitative - observation
European region (EUR)	We aimed to explore the experiences and	Qualitative - interview stu

European region (EUR)	To address the impact of th	Quantitative - observation
Eastern Mediterranean region (EMR)	To explore Iranian cardiac n	Qualitative - focus group s
Region of the Americas (AMR)	The goal was to explore pat	Qualitative - interview stu

European region (EUR)	The aim was the detailed as	Qualitative- survey
Region of the Americas (AMR)	To examine the<br>implementation of	Qualitative - interview stu
Western pacific region (WPR)	Given the importance of clir	Qualitative - interview stu
European region (EUR)	The aim of this study was<br>to assess how	Qualitative - interview stu
European region (EUR)	The specific questions that<br>this feasibility study aims	Qualitative - interview stu

Region of the Americas (AMR)	The goal of this study was t[illegible]	Qualitative - interview stu[illegible]

Region of the Americas (AMR)	To verify Primary Care nurse	Qualitative- survey (open-
Region of the Americas (AMR)	This paper describes the design and development	Other

African region (AFR)	evaluated a specialized mHe	Qualitative - interview stu
African region (AFR)	This study evaluated facilita	Other

European region (EUR)	This study aims to explore p	Qualitative - interview stu

Region of the Americas (AMR)	They examined the effect o	Quantitative - observation
European region (EUR)	The aim of this study was to	Qualitative - interview stu

Region of the Americas (AMR)	Given the need to formally a	Qualitative - interview stu

Region of the Americas (AMR)	[illegible] randomized controlled trial	Qualitative - interview stu

Region of the Americas (AMR)	To understand perceptions	Mixed methods
Region of the Americas (AMR)	Cardiologists' perceptions o	Qualitative- survey
European region (EUR)	This mixed-methods proces	Qualitative - interview stu

European region (EUR)	To assess the acceptance of	Quantitative - observation
Western pacific region (WPR)	This pilot study aimed to ev	Qualitative - interview stu
Region of the Americas (AMR)	To characterize patients’ an	Mixed methods

Region of the Americas (AMR)	The purpose of this paper is	Other

African region (AFR)	explored the perceived facil	Other
European region (EUR)	The aim of this study was to understand the benefits	Qualitative - interview stu

Western pacific region (WPR)	aimed to gauge the opinion	Other

Region of the Americas (AMR)	The aim of this study was to assess the opinion of	Quantitative - observation

Region of the Americas (AMR)	The objective of this paper i	Qualitative - interview stu

European region (EUR)	To provide a snapshot of th[illegible]	Quantitative - observation[illegible]
European region (EUR)	To assess the perception of	Mixed methods

European region (EUR)	To identify barriers and faci	Mixed methods

European region (EUR)	This study aims to gain insig	Qualitative - interview stu

Eastern Mediterranean region (EMR)	[illegible] is to explore the	Qualitative - interview stu

Region of the Americas (AMR)	A survey evaluation was cor	Qualitative- survey (open-
Region of the Americas (AMR)	The overall goal of this study is to identify	Other

Region of the Americas (AMR)	This study addressed the fe	Other
European region (EUR)	To explore the perspectives of three	Qualitative - focus group s

Region of the Americas (AMR)	To evaluate current awaren	Other

Region of the Americas (AMR)	The aim of this study was to	Qualitative - interview stu
Region of the Americas (AMR)	This study aims to investiga	Qualitative - interview stu

Region of the Americas (AMR)	To characterize the workflo	Mixed methods
South-East Asia region (SEAR)	[illegible] describe the challenges	Qualitative - interview stu

European region (EUR)	aimed to evaluate the expe	Qualitative - interview stu

Region of the Americas (AMR)	the objective of this qualitative descriptive	Qualitative - interview stu

European region (EUR)	To understand healthcare p	Qualitative - focus group s

Western pacific region (WPR)	[illegible] to the research and	Qualitative - interview stu
Western pacific region (WPR)	The aim of this paper is to s	Mixed methods

Western pacific region (WPR)	to explore the barriers and [illegible]	Qualitative - interview stu[illegible]

Western pacific region (WPR)	This study explored the exp	Qualitative - interview stu

Western pacific region (WPR)	aimed to describe how telel	Other

European region (EUR)	The aim was to explore pati	Qualitative - interview stu
Western pacific region (WPR)	This study aimed to explore the needs of	Qualitative - interview stu

European region (EUR)	To explore the usability, acc	Qualitative - interview stu

Western pacific region (WPR)	Thus, in the present study, v	Quantitative - observation
European region (EUR)	This study aimed to explore	Qualitative - interview stu

European region (EUR)	They employed a design thinking tool called a	Qualitative - interview stu
Western pacific region (WPR)	The aim of this work was to	Qualitative- survey
European region (EUR)	The aim of this research is 1	Qualitative - interview stu

Region of the Americas (AMR)	In order to address these gaps, the American College	Qualitative- survey

Region of the Americas (AMR)	The objectives of this paper	Qualitative - interview stu

Region of the Americas (AMR)	This study explored clinician	Qualitative - interview stu

Region of the Americas (AMR)	To explore barriers and ena	Mixed methods

Western pacific region (WPR)	To identify factors that influ	Qualitative- survey (open-
Eastern Mediterranean region (EMR)	To pilot test an Arabic versi	Qualitative - interview stu

If 'other' please specify
Focus groups were also done but this was [illegible]
online survey: Likert scale and Likert-style questions

dy

semi-structured interviews continued until

dy

free-text written patient feedback provide

dy

dy

dy

al - cross-sectional
dy

nal - cross-sectional

Open-ended questions

It was part of a larger mixed methods pilot

al - cross-sectional

semi-structured online interviews were

dy
interventional, quasi-experimental (qualita

qualitative observational longitudinal

Although this survey is cross-sectional, it e[illegible]
dy

al - cross-sectional

dy

dy

dy

dy

[illegible] measured blood pressure)

It was part of a multimethod single-case st

dy
dy

quantitative experimental/RCT + qualitativ
The qualitative process was embedded into a larger quasi-experimental

[illegible]
(alpha testing) which also included

dy

quantitative experimental + qualitative sur

dy

al - cross-sectional
tudy

It was part of a larger RCT

dy

dy

A medical and sociological study was condu

al - cross-sectional
dy

study

dy

Also included some quantitative data colle
Part of a larger mixed-methods implement
Plus acceptance scale
It was part of a larger mixed-methods stud

dy

ended)

dy
Interviews and focus groups

dy

[illegible] practical, robust implementation and

This was part of a larger study which incluc

al - cross-sectional

Collection of a research team's "lessons lea

qualitative: 44 key informant interviews an
They adopted a case study method, an "en

qualitative: focus group + interview

al - cross-sectional

al - cross-sectional

dy

Qualitative descriptive study

complete an online survey and attend one
study

qualitative focus group sessions and semi-s

dy

dy

dy

included short quantitative questionnaires

study

This study also included focus groups, but [illegible]
It was part of a clustered randomized cont

dy

dy

interviews + focus group

dy
The semis-structured interviews were part

dy

this study also included focus groups along

Interviews were used to inform a custome

dy

cross-sectional survey
dy

**Please specify study design details (if needed) (e.g., semi-structured interv**
in-depth interviews (semi-structured interviews)
physicians of the Med Center Health Physician group (most respondents were primary care
semi-structured interviews

selected because they have experience in telehealth with a minimum of 11 [illegible]

Qualitative sub-study within a randomised controlled trial
semistructured interviews

patients with chronic HFrEF being treated in regional heart failure serv

At the end of the study, the CHW (community health worker) was interview
semistructured interview guide to assess the implementation outcomes of f

Semi-structured interviews

Quantitative longitudinal evaluation of telemetry use (to answer aim 1) and
Qualitative case-study approach involving semi-structured interviews (to [illegible]

[illegible]
AF-SCREEN International Collaboration. Then, it was distributed in two

Individual structured interviews

a cross-sectional web-based questionnaire study

Surveys and in-depth interviews
[illegible] openended questions to assess the perceived utility and benefits of the

Qualitative data collection within a mixed-methods design
Semi-structured interviews

An online survey consisting of 43 multiple-choice questions was emailed to

5 healthcare professionals (pharmacists currently associated with the

exploratory qualitative online consultation

Semi-structured interviews
face-to-face interviews with 13 patients with chronic hypertension and 8 sta[illegible]

this study is part of the CHAT-GP project, a national partnership that a

Multiple choice questions (quantitative) and open-ended (qual)

individual semi-structured interviews over zoom (open questions) were perf

Qualitative interviews alongside a quantitative survey

The HRS (Heart Rhythm Society) Digital Health Committee independently de

semistructured interviews with YNHH clinical care team members, the app c

semi-structured interviews conducted either in-person or via telephone

Semi-structured interviews

Qualitative data collection within a service evaluation with quantitative obs
Surveys, training analytics, semi-structured interviews

A qualiatative phenomenological study involving face-to-face semistructure

A survey with pre-specified statements for stakeholders to rate, plus option

semi-structured interviews

Semi-structured interview based on the 4 overarching constructs outlined ir

semistructured web-based interviews of >50% the cardiotoxicity provi
25 semistructured (open-ended questions) interviews were conducted with

semi-structured interviews
Semi-structured face-to-face interviews and observation notes

[illegible]

Quanitative - satisfaction level questionnaire for remote device clinic issues

semi-structured interviews conducted face-to-face

9-question provider survey

Semi-structured interviews

It included a cross-sectional survey design and the vignette approach to explore the determinants of users' acceptance of EPP. (electronic patient
Six focus groups (two for each participant category: patients, health care pr

Semi-structured interviews

Semi-structured interviews

semi-structured interviews

Survey of doctors using a specially developed questionnaire aimed at assess

Medical-sociological study was conducted, including anonymous questionna
Semi-structured interviews

One focus group for phase 1 of the study, which focused on the interventior
semi-structured interviews via telephone with 43 patients + 16 clinicians

Two opinion surveys with only close-ended questions to assess the feasibilit
Semi-structured interviews that were obtained over years. Each year 3-5 VH
After the teleconsultation, both the physician and patient were interviewed
Semi-structured interviews (face-to-face)

semistructured interviews with 31 patients and 17 cardiology provider

online questionnaire
Qualitative - a focus group and an individual interview

6 informant remote interviews

They conducted [illegible] in-depth in-person interviews and [illegible] focus group
discussions with 3-6 participants each comprised of physicians, nurses,

Semi-structured interviews

A web-based questionnaire was administred to Canadian CRPs (cardiac reha
Qualitative interviews as part of staged intervention development and simu

semi-structured interviews

Semi-structured debrief interview

Online questionnaire included multiple choice (quantitative), Likert scale typ
[illegible] data was valid. On a self developed non-validated questionnaire, they
Qualitative interviews as part of a mixed-methods process evaluation of a ra

Questionnaire with multiple choice questions
Qualitative interviews as part of a pilot study including pre-post questionnai
Included a survey with both quantiative and qualitaitve options (sent aroun

"Lessons learned" reported by the research team - not systematically collec

[illegible]d 8 focus group discussions

Semi-structure interviews

3 rounds of semi-structured focus groups and qualitative interviews using ar

A cross-sectional online survey consisting of 40 questions was developed us

Preliminary findings from an ongoing larger mixed-methods research study

Survey with multiple choice questions
Semi-structured interviews and focus group

Semi-structured focus groups and individual interviews

Semi-structured interviews

Survey (electronic) collecting quantitative and qualitative data, but only qua
Workshops that used participatory health research

8 healthcare providers
Focus groups with HCPs and support staff from GP practices will also be use

small sample of cardiologists and health IT administrators

Semi-structured interviews
Qualitative interviews conducted with clinicians as part of a randomised co[illegible]

Norandomized pilot phase (qualitative (semi-structured interviews and obse
Semi-structured interviews

semi-structured interviews were conducted using Microsoft Teams with 8 c[illegible]

Semi-structured interviews

[illegible]
1.) explore the importance and uses given by healthcare professionals to

Interviews
Interviews

Semi-structured interviews

semi-structured interviews were conducted in English or Mandarin with thir

Semi-structured interviews (n = 8) and focus groups (n = 7) were conducted

Semi-structued interviews where the focus of the interviews was on their ex
Semi-structued interviews

Semi-structured dyadic interviews

Survey with one section about ICT for home-based care for elderly patients
Semi-structured interviews

telephone interviews
[illegible] the Likert scale and open-ended survey questions.
semi-structured video interviews

An online, 31-question, multiple-choice and qualitative survey was sent to A

Qualitative interviews within a qualitative case study

semi-structured interviews with providers from the Hospital for Sick Childre

Focus groups and individual interviews

valid questionnaires from GPs (web-based questionnaire survey answered u
semi-structured interviews were conducted with 10 community pharmacist:

**Classification of health-care providers**
Mix of HCPs
Clinicians/Doctors/Physicians
Mix of HCPs

Mix of HCPs

Clinicians/Doctors/Physicians

Mix of HCPs
Mix of HCPs

Mix of HCPs
Nurse practioner

Other
Mix of HCPs

Mix of HCPs

Clinicians/Doctors/Physicians
Mix of HCPs

Mix of HCPs
Mix of HCPs
Mix of HCPs

Clinicians/Doctors/Physicians

Mix of HCPs
Other

Other
Mix of HCPs

Clinicians/Doctors/Physicians

Pharamacists

Mix of HCPs

Mix of HCPs
Mix of HCPs

Mix of HCPs

Clinicians/Doctors/Physicians
Mix of HCPs

Clinicians/Doctors/Physicians
Mix of HCPs

Clinicians/Doctors/Physicians

Other

Mix of HCPs
Mix of HCPs

Other
Mix of HCPs

Mix of HCPs

Mix of HCPs

Clinicians/Doctors/Physicians

Mix of HCPs

Clinicians/Doctors/Physicians
Mix of HCPs

Mix of HCPs
Mix of HCPs

Mix of HCPs

Other

Mix of HCPs

Mix of HCPs

Mix of HCPs

Mix of HCPs
Mix of HCPs

Mix of HCPs

Mix of HCPs

Mix of HCPs

Clinicians/Doctors/Physicians

Clinicians/Doctors/Physicians
Mix of HCPs

Clinicians/Doctors/Physicians
Nurse
Mix of HCPs

Pharamacists
Other
Mix of HCPs
Clinicians/Doctors/Physicians
Mix of HCPs

Mix of HCPs

Nurse
Nurse practioner

Other
Mix of HCPs

Clinicians/Doctors/Physicians

Other
Clinicians/Doctors/Physicians

Mix of HCPs

Nurse

Mix of HCPs
Clinicians/Doctors/Physicians
Mix of HCPs

Clinicians/Doctors/Physicians
Clinicians/Doctors/Physicians
Mix of HCPs

Other

Mix of HCPs
Clinicians/Doctors/Physicians

Mix of HCPs

Clinicians/Doctors/Physicians

Mix of HCPs

Mix of HCPs
Mix of HCPs

Mix of HCPs

Mix of HCPs

Mix of HCPs

Mix of HCPs
Mix of HCPs

Physiotherapists
Mix of HCPs

Other

Physiotherapists
Mix of HCPs

Mix of HCPs
Mix of HCPs

Mix of HCPs

Mix of HCPs

Mix of HCPs

Other
Other

Mix of HCPs

Mix of HCPs

Mix of HCPs

Mix of HCPs
Mix of HCPs

Physiotherapists

Clinicians/Doctors/Physicians
Mix of HCPs

Mix of HCPs
Mix of HCPs
Mix of HCPs

Mix of HCPs

Mix of HCPs

Mix of HCPs

Mix of HCPs

Clinicians/Doctors/Physicians
Pharamacists

Methods
**If other, please specify, or if mix of HCPs, please specify which HCPs(e.g. clinicia**
HCPs (nurses, clinician, physician's assistant and a pharmacist), and policy maker
Physicians, Registered Nurses, Clinical Researcher

7 cardiologists, 3 internists, and 14 nurses from two major hospitals in Amman

Doctors (1 resident physician, 5 general practitioners), 9 nurses (3 district nurses,
physicians (4) and nurse practitioner (1)

Cardiologists (4) and nurse (1)
6 HFSNs

Community health worker
Primary care physician, specialist medical doctor, pharamacist, nurse

Primary care physicians, endocrinologists, and advanced practice nurses

Primary care doctor, Public health practitioner, Social care worker, Physician, Bus

HCPs (11) and key informants (4)
Electrophysiologist, general cardiologist, neurologist/stroke physician, primary ca
Cardiologists, nurses, mental health specialists, residents in cardiology, health se

Physical therapists, occupational therapists, rehabilitation physicians, speech the
Primary care providers

9 providers and 2 staff employed for programme intake and orientation
Pharmacist and primary care physician

Cardiologists (either fellows in training or experts)

[illegible]
Association (ACRA) were invited :

Doctors, nurses, pharmacists
2 physicians, 3 care managers, 1 senior nurse clinician and 2 care coordin

12 individual practice staff including GPs and nurses using the app an ave

This included physicians, advanced practice providers, and other clinicians. Only [illegible]

nurses (5) and physiotherapists (7) with experience of using the DPE as part of a [illegible]

Cardiac electrophysiologist, Cardiologist, Electrophysiology fellow, Cardiology fell

11 physicians and 6 nurses/medical assistants from primary care settings

50 HCPs working in cardiology, rehabilitation, cardiac rehabilitation, and aged car
Physicians, dietitians, physiotherapists, nurses and mental health professionals

3 "cardiac rehabilitation exercise professionals"
Primary care providers (physicians, physicians assistants, nurse practitioners, and

General practitioners, clincial pharmacist, dietitian, exercise physiologist, counse

26 participants: 6 physicians, 3 nurses, 5 pharmacists, 2 psychologists, 2 technolo

Family medicine and internal medicine PCPs (primary care physicians) from a larg

Frontline providers and management (administrators) direclty involved (ie using

2 board-certified general cardiologists, 2 oncologists, 3 hematologists, 3 cardio-c
6 cardiology and 6 rheumatology (5 nurses and 7 doctors)

6 physicians (one from a community-based clinic, three from academic Family Me
General practitioners, practice nurses, and healthcare assistants

Cardiologists, cardiology specialist nurses, interventional radiology matron

[illegible]
Group included: Registered nurses, nurse practioners, physician's assistant,

11 staff members including dentists, dental therapists, dental nurses, and manag

physician, advanced practitioner,nurse/MA/other

Administrative workers and care providers (community health worker, health edu

Physicians and nurses
Nurses, nurse practioners, kinesiologists, physicians, cardiac and/or respiratory s[illegible]

Team members, cardiologists and general practitioners (GPs)

Nurses, cardiologists, general practitioner, pharmacists

nurses, residents and obstetricians working in the digital monitoring team as par

Doctors from 6 Bryansk polyclinics - they were general practitioners whose tasks

Primary health care physicians
Cardiologists, cardiac nurses, and professors in medicine AND managers

9 physicians, 2 nurse practitioners, 5 nurses

Veterans Health Administration staff and administration
2 doctors, 3 nurses, 4 allied health professionals, 1 hospital administrator
Cardiologists and cardiology residents
General practitioners and nurses

physician, clinical pharmacist, nurse

Here in paper described as nurse clinicians

IRC health facility staff involved in providing NCD care who had experience using

Physicians, Nurses/midwives, Administrators

General practitioners and cardiologists

CRP (cardiac rehabiliaton program) managers

cardiologists and nurse practitioner

Physicians, nurse practitioners, physician assistants, pharmacists, and executive c
Cardiologists
27 practitioners (13 doctors/GPs, 2 healthcare assistants, 1 GP practice manager,

Allied health, Physician, Nurse practitioner, Nurse, Cardiologist, Endocrinologist, [illegible]

Profession not reported, described as "primary care providers" and "clinical expe

frontline health workers such as medical doctors, clinical officers, nurses, pharma
Physicians were recruited from the head of the cardiology department, The Coor

38 participants across 3 rounds- round 1 included 8 participants (4 patients and 4

Cardiology, Internal medicine, General practitioner, and other speciality

20 HCPs: advanced practice nurses (n=7), art/music therapists (n=2), chaplains/sp

Medical specialists (Cardiologists, internists, cardiothoracic surgeons, pediatric ca
General practionners, practical nurses, other stakeholders

Primary care workers (PCWs) such as (GPs, clinical pharmacists, nurses) and non-

16 HCPs (4 doctors, 7 nurses, 1 physician assistant, 2 physiotherapists, 1 psycholo

Cardiologists, Internist, and nurses

26 HCPs working in cardiology (13 staff physicians, 8 physician trainees, 2 nurse p
Physicians and nurses with specialties in CV care

Clinical pharmacists and general practice staff (e.g., office support staff, receptio

interviews with 12 cardiologists and 8 health information technology (IT) adminis

Physiotherapists in pulmonary rehabilitation, and cardiac rehabilitation
9 HCPS (2 attending cardiologists, 2 cardiology fellows, 2 advance practice provid

Medical doctors, nurse practioners, registered nurses, pharmacists
Physicians, non-physician health workers, health administrator or policy maker

Royal Brompton Hospital - Four consultant cardiologists in the HF service, three s

cardiologists, nurses, nurse practitioners

Nurses, physiotherapists, physicians, cardiac physiologists.

[illegible]
Peer educators (from both rural and urban districts)

General practitioners, practice nurses, practice managers and practice nurses, cli

two family physicians, one nurse clinician, three care managers and two care coo

Clinical nurse (14), physiotherapist (11), exercise physiologist (2)

Physiotherapists and nurses
Cardiac rehabilitation nurses, medical doctors, cardiologists, physiotherapists, se

392 General Practitioners
General practitioner, healthcare assistant, practice nurse, practice pharamacist

Lead hospital information technology (IT) manager, a research nurse, the treating
clinicians and diabetes educators
Cardiologist, heart failure nurses, general pracitioners

All CV (cardiovascular)-professionals. Made up of cardiologists, advanced practice

13 HCPs (9 clinicians - cardiologists, nurses - and 4 operational staff)

4 cardiologists, 3 nurse practitioners and 1 cardiology fellow

Pharmacists, primary care physicians, and nurse practioners

**Classification of DHIs (https://www.ama-assn.org/sy**
Patient engagement (Solutions to promote patient we
Other/More than one
Patient engagement (Solutions to promote patient we

Other/More than one

Patient engagement (e.g., solutions to promote patier

Remote monitoring and management for improved ca
Other/More than one

Remote monitoring and management for improved ca
Other/More than one

Clinical decision support
Other/More than one

Tele-vists/ virtual visits

Clinical decision support
Other/More than one

Patient engagement (e.g., solutions to promote patier
Other/More than one
Other/More than one

Patient engagement (e.g., solutions to promote patier

Patient engagement (e.g., solutions to promote patier
Tele-vists/ virtual visits (An audio/video connection us

Remote monitoring for efficiency (e.g., smart versison
Remote monitoring and management for improved ca

Other/More than one

Other/More than one

Other/More than one

Other/More than one
Other/More than one

Clinical decision support

Consumer access to clinical data (e.g.,Secure access al
Other/More than one

Remote monitoring and management for improved ca
Other/More than one

Other/More than one

Other/More than one

Remote monitoring and management for improved ca[illegible]
Tele-vists/ virtual visits

Remote monitoring and management for improved ca
Other/More than one

Other/More than one

Patient engagement (e.g., solutions to promote patier

Remote monitoring and management for improved ca

Remote monitoring and management for improved ca

Remote monitoring and management for improved ca
Tele-vists/ virtual visits

Remote monitoring and management for improved ca
Other/More than one

Patient engagement (Solutions to promote patient we

Remote monitoring and management for improved ca

Other/More than one

Clinical decision support

Remote monitoring and management for improved ca

Consumer access to clinical data (e.g.,Secure access al
Other/More than one

Other/More than one

Other/More than one

Remote monitoring and management for improved ca

Remote monitoring and management for improved ca

Other/More than one
Other/More than one

Tele-vists/ virtual visits (An audio/video connection us
Patient engagement (e.g., solutions to promote patier
Remote monitoring and management for improved ca

Patient engagement (Solutions to promote patient we
Other/More than one
Tele-vists/ virtual visits
Tele-vists/ virtual visits (An audio/video connection us
Point of care/ workflow enhancement (Communicatio

Tele-vists/ virtual visits

Tele-vists/ virtual visits
Patient engagement (Solutions to promote patient wel

Point of care/ workflow enhancement (communicatio
Remote monitoring and management for improved ca

Tele-vists/ virtual visits

Patient engagement (Solutions to promote patient wel
Clinical decision support

Other/More than one

Other/More than one

Remote monitoring and management for improved ca
Patient engagement (Solutions to promote patient we
Remote monitoring and management for improved ca

Tele-vists/ virtual visits
Patient engagement (e.g., solutions to promote patier
Tele-vists/ virtual visits

Clinical decision support

Point of care/ workflow enhancement (communicatio
Remote monitoring and management for improved ca

Patient engagement (e.g., solutions to promote patier

Remote monitoring and management for improved ca

Remote monitoring and management for improved ca

Other/More than one
Remote monitoring and management for improved ca

Remote monitoring and management for improved ca

Other/More than one

Other/More than one

Tele-vists/ virtual visits
Other/More than one

Other/More than one
Remote monitoring for efficiency (e.g.,Smart versions

Other/More than one

Other/More than one
Clinical decision support

Remote monitoring and management for improved ca
Other/More than one

Tele-vists/ virtual visits

Other/More than one

Patient engagement (Solutions to promote patient we

Patient engagement (Solutions to promote patient wel
Patient engagement (Solutions to promote patient wel

Clinical decision support

Other/More than one

Other/More than one

Other/More than one
Other/More than one

Remote monitoring and management for improved ca

Tele-vists/ virtual visits
Patient engagement (e.g., solutions to promote patier

Tele-vists/ virtual visits (An audio/video connection us
Other/More than one
Remote monitoring and management for improved ca

Tele-vists/ virtual visits (An audio/video connection us

Remote monitoring and management for improved ca

Other/More than one

Patient engagement (e.g., solutions to promote patier

Remote monitoring and management for improved ca
Other/More than one

**If other or more than one, please specify**
The two parts of the intervention that we extracted analysis from
interactions using a computer, tablet, and/or smartphone, phone
PGHD

telehealth programs

Remote monitoring and management for improved care (e.g., App

recommend that RM should be viewed as a supplement to, r

uses algorithmic CVD risk stratification
Remote monitoring and management for improved care (e.g., App

Tele-vists/ virtual visits; Remote monitoring for efficiency (e.g., sm

Patient engagement; Remote monitoring and management for im

nt wellness and active participation in their care for chronic disease

nt wellness and active participation in their care for chronic disease

two-way videoconferencing technology. Some aspects of remote

Also included a bit of patient engagement (e.g., solutions to prom

General digital health technologies

smartphone app (Statinterface) included symptom logging, v

Range of tools to conduct CR; mainly using tele-visits (videoconfer

Patient engagement (e.g., solutions to promote patient wellness a
remote blood pressure monitoring program to measure and uploa

An integrated patient decision aid into the electronic health recor
use the DPE as a pedagogic tool

clinicians enrolled patients with the SMART on FHIR app, pr

core feature is an automated SMS text messaging program system

Patient engagement; Tele-vists/ virtual visits; Remote monitoring

Remote monitoring and management for improved care (e.g., App

re (e.g., Apps and devices for use by chronic disease patients for d

[illegible] promote patient wellness and active participation in their care

re (e.g., Apps and devices for daily measurement of vital signs and

re (e.g., Apps and devices for daily measurement of vital signs and
[illegible]<br>common clinical devices such as thermometers, blood pressure

Also included aspects of 'consumer access to clinical data' as the i

re (e.g., Apps and devices for use by chronic disease patients for d

screening tool

Electronic health record (EHR)-based shared decision-making (SDM

[illegible]re (e.g., Apps and devices for daily measurement of vital signs and

Patient engagement (e.g., solutions to promote patient wellness a

Remote monitoring and management for improved care (e.g., App

[illegible]
Apps and devices for use by chronic disease patients for daily

re (e.g., Apps and devices for use by chronic disease patients for d

General questions about mobile health (mHealth) in cardiac patients. BUT it appears to focus on remote diagnosis and

did not mention if video was used
nt wellness and active participation in their care for chronic disease
re (e.g., Apps and devices for daily measurement of vital signs and

also included aspects of point of care/workflow enhancement *it

[illegible]
common clinical devices such as thermometers, blood pressure

also included the possibility to conduct a video conference betwe

llness and active participation in their care for chronic diseases (e.g

n and sharing of electronic clinical data to consult with specialists,

llness and active participation in their care for chronic diseases (e.g

[illegible] promote patient wellness and active participation in their care

[illegible] common clinical devices such as thermometers, blood pressure

re (e.g., Apps and devices for daily measurement of vital signs and
Included some aspects of patient management but there wasn't a

n and sharing of electronic clinical data to consult with specialists,

nt wellness and active participation in their care for chronic disease

re (e.g., Apps and devices for use by chronic disease patients for d

Remote monitoring and management for improved care (e.g., App
re (e.g., Apps and devices for daily measurement of vital signs and

re (e.g., Apps and devices for daily measurement of vital signs and

The interviewes asked about "eHealth" in general, specifying "We

General telehealth

[illegible]
Questions were:

CR is an outpatient model of secondary preventive care proven to
Although it does not seem to automatically upload into medical re

AI-enabled digital health tools

re (e.g., Apps and devices for daily measurement of vital signs and

Clinical decision support AND Patient engagement (e.g., solutions

Remote monitoring and management for improved care (e.g., App

[illegible]
for improved care (e.g., Apps and devices for use by chronic

llness and active participation in their care for chronic diseases (e.g

llness and active participation in their care for chronic diseases (e.g

hypertension management = BP telemonitoring (allows BP reading

provision of exercise sessions and education in lifestyle change (i.e

Patient engagement (e.g., solutions to promote patient wellness a
General telehealth with a major focus on a self-management platf

re (e.g., Apps and devices for daily measurement of vital signs and

nt wellness and active participation in their care for chronic disease

Remote monitoring and patient engagement
including aspects of audio, and other telecommunications

[illegible]<br>televisits/consulatations

mobile phone-based digital therapeutic, known as the Medly prog

nt wellness and active participation in their care for chronic disease

re (e.g., Apps and devices for daily measurement of vital signs and
CVDRAM (CVD risk assessment and management)-related mobile

Description of DHI: please provide a short description of the DHI (if applicable)
This was part of a larger community initiative called [illegible] that included community-based education, community-based screening, management of hypertensive patients, etc. For our
rapid implementation of telemedicine during the COVID-19 pandemic in a southern United States rural population
General perceptions of patient-generated health data (PGHD) : defined as health-related data creat

immature implementation in telehealth programs in Jordan

Several different apps for patient self-management of hypertension were considered for use in Sau

Interactive web-based communication system: (a) blood pressure device; (b) self-reports, reminder
Participants dicussed how they envisioned a telemonitoring program could be used in the care of pa

Medly, a telemonitoring program for patients with HF launched at UHN in 2016, was chosen to facil
(Luscii app) a smartphone-based RM strategy combining self-measurement of vital signs (blood pres

[illegible]
stratification based on SMS text input. Users (CHWs) enter patient data as a single SMS text

A blood pressure cuff for intermittent remote BP readings via the Withings device and app

Teleheath visits for diabetes care by telephone and video (10/18 participants) and by telephone onl

CDS tool embedded in EHR to promote adherence to AHA telemetry monitoring practice standards
Digitally enabled electrocardiograms available in primary care (ECGs) and two telehealth projects, one for heart failure and the other obstructive sleep apnoea.

Mobile phone text messaging strategy targeting patients with hypertension
Focused on general perceptions of AF screening by consumer-facing wearables, devices and apps
Social robots that can be equipped with AI and embedded with remote monitoring technology to as

This is looking at general acceptance of medical apps for self-management of hypertension

Fast@home is a web-based eRehabilitation intervention developed to support stroke patients and h

[illegible]
practices per cohort) via interactive two-way videoconferencing technology; the patient groups of

Humanwide, an approach to provide personalized care at all points of the care continuum: wellness

[illegible]
pharmacist). They allow for real-time electronic home blood pressure monitoring and medication

Includes DHTs broadly: wearables, cardiac implantable electronic device telemonitorization, mobile

mobile app helps physicians manage patients at CVD risk level with statin intolerance (app was deve

General exploration into the experience of shifiting from in-person to remote CR

E-tool known as "Self-management and Education Tool for AF patients" (SETAF) that can be used to
Bluetooth-enabled home BP monitor (TaiDoc Technology FORA P20b Blood Pressure Monitoring Sys

developed a web-based CHAT-GP DA to provide a tool for clinicians to use with patients to

[illegible]
personalized risk estimates by connecting data existing within a patient’s EHR (electronic health

The DPE consists of 13 modules covering texts, illustrations, and short video clips with inte

Text-based BP monitoring is one strategy that has been shown to increase adherence to American [illegible]
Remote monitoring for diagnosis - consumer-available digital health devices for atrial fibrillation de[illegible]

smartphone application remote monitoring of women experiencing postpartum hypertensive disor[illegible]

MyBP program deploys automated bidirectional text messaging for HBPM (home BP monitoring) an

The study did not investigate one specific DHI, but asked HCPs about devices for cardiac monitoring
Virtual visits by phone or video call to replace all in-person visits for the majority (as it was done dur

Active+me is a medically certified (CE marked Class I) telemetry device (ISO 13485:2016) and has a [illegible]

ALTA is a multilevel technology-facilitated, team care approach in which clinical staff work collabora[illegible]

Exploring the perceptions of mobile apps to support self-management of heart failure

The "mHeart system" ("an internet- and theory-based holistic intervention model was implemented

General perceptions on self-measured blood pressure (SMBP recommendation and management) potential intervention strategies that might address known barriers).

[illegible]
physiological metrics including blood pressure, weight, heart rate, blood sugar and symptoms with

mHealth (app) symptom-tracking tool for early recognition of cardiotoxicity in patients with cancer
most remote consultations were via telephone although a few cardiology clinicians and two

home-based virtual hypertension programme (virtual collaborative care clinic (vCCC) to remotely m

[illegible]
sphygmomanometer and shown how to submit BP readings via a low-Financing/cost third-party

The aim of the patient decision aid [illegible] was to provide decisional support for patients
experiencing symptoms of stable angina and deciding whether to continue with cardiac medicines

Focused on managing a remote monitoring device clinic with patients having some form of cardiac i

for screening AF using small hand-held (portable)device

CV Wizard is a web-based shared decision-making (SDM) and clinical decision support (CDS) system

Bluetooth-enabled Self Measured Blood Pressure monitoring (SMBP)

Electronic patient portals (EPPs) are a secure online platform where patients enter a password to a[illegible]
Web-based computer-tailored intervention, which allows action planning and personalized messagi[illegible]

[illegible]
management, and to provide clinicians with daily home-based readings blood pressure (BP), heart

[illegible]
tablet, a personal computer or smartphone application, and a Google Home interface. The sensors

Pregnant women with a high risk of HPD (hypertensive disorders related to pregnancy), use a mobile app to measure their blood pressure at home, the

General questions on remote monitoing for chronic patients - with a focus on arterial hypertension

[illegible] communication with patients, attitude and willingess of doctors to remotely monitor the health

[illegible]
Reminders via a text-messaging technology to improve acute coronary syndrome patients’ adheren
remote monitoring technology to track patients’ weight and diuretic adherence - a digital scale, an [illegible]

locates risks. For this study, the personal health planning could be made individually at home or

Administration) HT (home telehealth) telemonitoring with telehealth-based self-management

Telehealth in genral: "Telehealth in the region is largely delivered within the public health system a

all consultations that did not require direct contact or specialized examination were required to be

Cardio-Share model is based on the use of a compact heart sensor that registers the heart rhythm.

virtual visits

remotely monitoring individuals via telephone calls

[illegible]
of the game design elements in a non-game context.

the Sana.NCD mobile health (mHealth) app, initially developed in Lebanon, is the only known mHea
General perceptions of a home blood pressure monitoring program in the management of hyperten

[illegible]
appointments using videoconferencing equipment to involve live interaction among

General perceptions of remote cardiac rehabilitation programs - telerehabilitation during the COVID-19 pandemic
A prediction tool for improving the support for clinical decision-making in ICD remote monitoring ba

[illegible]
<50% of recommended PA will prompt an alert to an HCP > cardiac rehabiliation referral and/or PA

There were two digital health applications tested and evaluated.
Guardian user testing (in-hospital) remote monitoring

Self-measured blood pressure monitoring
A fitness tracker wristband (Microsoft Band), instructed to use and asked to wear for 2 weeks. The
HOME BP was an online intervention for patients and practitioners which aimed to reduce uncontro

Telemedicine in cardiology care
Participants were sent SMS messages over 6 months, each with a URL link to a webpage containing
Focused on experiences with the quality and sustainibililty of synchronous telephone and video visi

Clinical decision support (CDS) tool: A fully integrated application that computerized national clinica

National health management information system (HMIS) = helps connecting all levels of integration

[illegible]
of cardiac chronicity.

digital health technology (supporting self-management app)

Focused on general perception of telehealth, but with a focus on remote blood pressure monitorin[illegible]

The study informed the later design/develompent of an mHealth intervention for heart failure patie

Digital health broadly: Smartwatches; implantable technologies with sensors or devices for decision
A digital health panel that facilitates the assessment of patients outside their usual clinical setting

BP@home is an NHS-funded remote blood pressure monitoring programme

The interviewes asked about "eHealth" in general, specifying "We asked about both the use of eHea

"Virtual care" by telephone and video platform ("The development of Web-based platforms and tec

cardiac patients from a CR programme received a 12-week virtual educational intervention (via Wh[illegible]
[illegible] Mobile single-led handheld ECG device.

N/A

[illegible]<br>Here tele-rehabilitation is defined as the remote delivery of rehabilitation services, inclusive of
Decision aid for atrial fibrillation, desigend to help clinicians lead shared decision-making conversati

Continuous wearable AI monitoring analytics, with notifications to alert clinicians of potential heart

[illegible]
electronic health records, task-sharing via trained NPHW (non-physician health worker), and

Telephone appointments or video consultations were conducted using the AttendAnywhere web-b

Questions were focused on an perceptions of an app for heart failure management

[illegible]
obtain accurate information about cardiovascular diseases, enabling access to preventive services,

[illegible] disease management in Cambodia. The mHealth mobile app is focused on disease management
[illegible] (electronic data capture by tablet) to improve hypertension and diabetes

Future Health Today (FHT) is software that facilitates clinical decision support and quality improvem

each participant in the intervention group was loaned a Bluetooth-enabled home BP monitor and m

telehealth programmes for cardiac and pulmonary rehabilitation provide long- term self-manageme

cardiac telerehabilitation program: [illegible] is a 12-week digital cardiac telerehabilitation program that includes supervised home-based video training, a web-based session with a cardiac
Questions were related about general perceptions on a digital health platform to support CVD disea

A mHealth app designed to support remote exercise-based CR of elderly patients early after cardiac

ICT for home-based care for elderly patients with heart failure ("In addition, information and comm
Views were collected on a potential SMS text message or smartphone app intervention that could support hypertension medication adherence

A video consultation that included collaboration between a patient, a nurse and a cardiologist. A nu

The MOTHer app is used for data input, such as blood glucose levels, weight, exercise, and sympton

[illegible]
physiological measurements with the aid of health-care devices such as weight scales, blood

Survey questions were related to overeall telehealth (it asked the HCPs to define what telehealth m

Mobile phone-based telemonitoring system (" "Medly" program, a mobile phone–based telemonito

digital therapeutics used as an evidence-based tool to support heart disease management

An eHealth tool for diabetes, kidney and cardiac patients to self-manage sick days (with guidance)

wearable electrocardiograph devices (WEDs) = worn on the body as an accessory to continuously c[illegible]

web and mobile application

**Type of CVD prevention**
Primary
Secondary
Secondary

Secondary

Primary

Primary
Primary

Secondary
Secondary

Primary
Primary

Primary

Secondary
Secondary

Primary
Primary
Secondary

Primary

Secondary
Primary

Primary
Primary

Secondary

Secondary

Secondary

Secondary
Secondary

Primary

Primary
Secondary

Primary
Primary

Secondary

Primary

Secondary
Secondary

Secondary
Primary

Secondary

Secondary

Primary

Both

Primary
Secondary

Primary
Primary

Secondary

Both

Primary

Primary

Primary

Primary
Secondary

Secondary

Secondary

Primary

Both

Primary
Secondary

Secondary
Secondary
Secondary

Primary
Primary
Primary
Both
Primary

Secondary

Primary
Secondary

Primary
Primary

Secondary

Secondary
Secondary

Primary

Secondary

Primary
Secondary
Primary

Both
Primary
Both

Primary

Primary
Secondary

Secondary

Primary

Secondary

Both
Primary

Primary

Secondary

Secondary

Secondary
Secondary

Secondary
Primary

Secondary

Secondary
Primary

Secondary
Secondary

Secondary

Secondary

Secondary

Primary
Primary

Both

Primary

Secondary

Secondary
Secondary

Secondary

Secondary
Primary

Secondary
Primary
Secondary

Secondary

Secondary

Secondary

Both

Primary
Primary

**Sample size (number of HCP participants) - If mix of HCPs, please provide total and number per HCP (e.g. 100: clinic**
17 (10 HCPs- 7 nurses, 1 clinician, 1 physician's assistant and a pharmacist), and 7 policy makers
58 providers
7 HCPs (physician n=4, registered nurses n=1, clinical researcher n=2)

24 health professionals at two hospitals in different clinical areas with experience helping patients with CVDs

12

15 (6 doctors/physicians, 9 nurses)
5 (physicians n=4 and nurse practitioner n=1)

5 (4 cardiologists, 1 nurse)
6 heart failure specialist nurse users of the remote monitoring platform

1 CHW (community health worker)
15 (primary care physicians n=4, specialist medical doctor n=4, pharamacist n=1, nurse n=1) * only 10 reported their d

6 (number per type of HCPs not provided)

16
11 - Primary care doctor (6), Public health practitioner (1), Social care worker (1), Physician (2), Business consultant fo

11 HCPS (2 medical officers, 4 doctors, 1 nurse, 4 health assistants/auxilliary nurses), 4 key informants (3 policy/gover
588 HCPS but only 475 responded about their profession (electrophysiologist = 37.1%, general cardiologist = 17.7%, n
22 - cardiologists (7), nurses (4), mental health specialists (3), cardiology residents (2), health services management w

62 - occupational therapists (17), physical therapists (20), rehabilitation physicians (7), psychologists (4) , speech thera
N/A

11 HCPSs (9 providers and 2 staff employed for programme intake and orientation)
2 (pharmacist n=1 and primary care physician n=1)

308 cardiologists

5 healthcare professionals (pharmacists currently associated with the medical center of The Ohio State Univ

30 (registered nurse n = 13, exercise physiologist n = 3, and physiotherapist n = 1)*only 17 members provided their de

12 - Doctors (4), Nurses (4), Pharmacists (4)

8

12 providers

109
12 providers

2
1601 (Cardiac electrophysiologist [43.6%], Cardiologist [9.5%], Electrophysiology fellow [6.2%], Cardiology fellow [6.6%

4 providers

17 providers

50 HCPs working in cardiology, rehabilitation, cardiac rehabilitation, and aged care services, at 3 major tertiary-level h
6 (number per type of HCPs not provided)

3 "cardiac rehabilitation exercise professionals"
67 were interviewed, but number per type of HCPs not provided for those interviewed

6 (general practitioners n=2, clincial pharmacist n=1, dietitian n=1, exercise physiologist n=1, counselor n=1).

26 participants: 6 physicians, 3 nurses, 5 pharmacists, 2 psychologists, 2 technology analysts, 3 key representatives of

17 physicians

7 (frontline providers n=4, management administrators n=3) *but only n=6 completed the questionnaires

11 providers
12 clinicians

11 providers
8 (GPs, practice nurses, and healthcare assistants) *n value per specality is not known

9 healthcare professionals (cardiologists n=4, cardiology specialist nurses n=4, Interventional radiology matron n=1)

45 (Registered nurses n=19 , nurse practioners n=14, physician's assistant n=2, technicians n=5 and others n=5)

44 clinic providers

10 - care providers (4) and administrative workers (6) (number per type of HCPs not provided)

17 *not possible to determine 'n' by speciality
29 (16 HCPs and 13 managers) - Nurses (6), nurse practioners (2), kinesiologists (6), physicians (2), cardiac and/or resp

10 (teleclinical care team members = 4, cardiologists = 3, GPs = 3)

9 (nurses n=4, cardiologists n=3, general practitioner n=1, pharmacists n=1)

14 HCPs (nurse n=6, resident n=2, obstetrician n=6)

93

284 primary health care physicians
13; 8 (cardiologists n=3, cardiac nurses n=2 and professors in medicine n=3) AND 5 managers (hospital managers n=1,

N/A
23
16 clinicians

75 resident pharmacists
15
10 (2 doctors, 3 nurses, 4 allied health professionals, 1 hospital administrator)
8 (cardiologists n=5, cardiology residents n=3)
[illegible] (general practitioner n=[illegible] and nurses n=[illegible])<br>[a group of six GPs and two nurses, all employed at the same shared clinic in Copenhagen, participated in the

17 providers

65 primary care nurses
8 clinic nurses

6 clinical staff
22 (physicians n=11 [cardiology, obstetrics and gynaecology, maternal-fœtal medicine, resident doctors], Nurses/midv

11 physicians (general practitioners n=7 and cardiologists n=4)

114 (10 CRP managers represented 3 to 12 separate CRPs)
7 cardiologists

4 (cardiologists n=3, nurse practitioner n=1)

26 (Canada n=15 and UK n=11)

65 - physicians (36), nurse practitioners (19), physician assistants, pharmacists, and executive directors (10)
6
27 (13 doctors/GPs, 2 healthcare assistants, 1 GP practice manager, 11 nurses)

97
7 General Practitioners
54 total: For the survey, 40 (Allied health n=8, Physician n=7 , Nurse practitioner n=3, Nurse n=2, Cardiologist n=6, Enc

Not reported how many HCPs feedback the "lessons learned" are based on (203 primary care providers participated ir

44
N/A (based on quotations likely 5)

4-13-8 (round 1-2-3)

1753 physicians (Cardiology n=714, Internal medicine n=245, General practitioner n=197, other=523)

20 HCPs: advanced practice nurses (n=7), art/music therapists (n=2), chaplains/spiritual care (n=2), physicians (n=4), re

117 - medical specialists (74), cardiopulmonary technicians/clinical physiology technicians (28), medical residents (9),
19 HCPs - general practionners (9) and practical nurses (10) and 5 other stakeholders

20 - general practionner (6), clinical pharmacist (3), practice nurse manager/lead and nursing associate (3), program/p

16 HCPs (4 doctors, 7 nurses, 1 physician assistant, 2 physiotherapists, 1 psychologist, 1 life coach)

12 (cardiologists n=4, internist = 1, nurses=7)

26 HCPs working in cardiology (13 staff physicians, 8 physician trainees, 2 nurse practitioners, 2 allied health professio
7 (phyisicians and nurses with specialties in CV care, endocrinology, nephrology, and internal medicine) n value not de

8 HCPs
13 (CPs [clinical pharmacists] n=4 and GPS [general practice staff] n=9)

20 (Cardiologists (12) and health information technology administrators (8))

7 physiotherapists (six practising solely in pulmonary rehabilitation and 1 practising in both pulmonary and cardiac reh
9 HCPS (2 attending cardiologists, 2 cardiology fellows, 2 advance practice providers (APPs), and 3 primary care attenc

14 (number per type of HCPs not provided)
56 (physicians n=38, non-physician health workers n=14, health administrator or policy maker n=4)

8 clinicians

21 (cardiologists n=8, nurses n=6, nurse practitioners n=7)

19 (nurses n=11 , physiotherapists n=2 , physicians n=3, cardiac physiologists n=3)

11 peer educators
6

16 - general practioners (8), practice nurses (6), practice manager and practice nurse (1), clinical health assistant (1)

8 HCPs

27 (22 clinicians and 5 director/team leader)

4 (nurses=2, physiotherapist=2)
8 *cannot determine make-up of HCP (number per HCP per category)

392 General Practitioners
11 (General practitioner n=1, healthcare assistant n=2, practice nurse n=6, practice pharamacist n=2)

4 (Lead hospital information technology (IT) manager n=1 , a research nurse n=1, the treating cardiologist n=1 and the
N/A (clinicians and diabetes educators)
8 HCPs (cardiologist n=1, heart failure nurses n=5, general practitioner n=2)

342 CV (cardiovascular)-professionals (cardiologists n=315, Advanced practice nurse n=12, physician assistant n=4, nu

13 HCPs (9 clinicians - cardiologists, nurses - and 4 operational staff)

8 providers

28 - pharmacists (13), primary care physician (12), nurse practitioners (3)

1,004 providers
10 community pharmacists

Results

Sample Size	Enter mean (SD) age of HCPs	Mean age
17	N/A	
58	N/A	
7	N/A	

24 ese professionals were between 34 and 58 years

12	Mean 40 years (range 28-57)	

15	Mean age 55 years (range 29-71)	
5	N/A	

5	Not reported	
6	N/A	

1	N/A	
15	N/A	3 (30%) * only 1

6	50 (5.1)	

16	NA	
11	NA	

15	Not reported	
588	N/A	
22	49.5	

46	34.28 (8.6)	

62	NA	
22	N/A	
878		

11	Not reported	
2	N/A	

308	N/A	

5	N/A	

30	N/A	3 (17%) * only 1

12	NA	
8	N/A	

12	N/A	

109	N/A	
12	49.5	

2	Not reported	
1601	N/A	

4	N/A	

17	31	

50	Not reported	
6	39 (R = 30-62)	

3	Not reported	
67	NA	

6	38 (5.2)	

26	Mean age 46 (SD 10) years	

17	47.3±12.2	

7	N/A	

11	N/A	
12	N/A	

11	N/A	
8	N/A	

9	42 (6)	

45	49 (11.69)	

11	N/A	

44	N/A	

10	43 (16.47)	

	39.5 (9.5)	
29	NA	

10	N/A	

9	N/A	

14	39.01 (10.69)	

93	N/A	

284	47.01 (15.8)	
13	N/A	

N/A	N/A	
23	36.64 (6.69)	
16	N/A	

75	N/A	
15	N/A	
10	Not reported	
8	N/A	
2	N/A	

17	N/A	

65	36	
8	N/A	

6	N/A	
22	N/A (only have the value including with patients)	N/A ([illegible]

11	N/A	

114	N/A	
7	Mean 52 years, range 43-62	

4	N/A	

26	N/A	

65	NA	
6	N/A	
27	Not reported	

97	R = 20-29 to > 80 (56.7% were aged between 40 and 59)	
7	Not reported	
54	N/A	Interview : [illegible]

	Not reported	

44	N/A	
5	N/A	

16	N/A	

1753	50.8 (12.2)	

20	Not reported	

117	R = <30 to ≥70 (60% of the respondents were aged >50)	
24	41 (R = 31–65) for HCPs only	9 (26%) for HCPs only; [illegible]

20	NA	

16	Not reported	

12	42.2 (N/A)	

26	Not reported	
7	N/A	

8	N/A	
13	N/A	

20	N/A	

7	N/A	
9	Not reported	

14	R = 29-63	
56	49.5 (SD not reported)	

8	N/A	

21	42.9 (8.6)	

19	N/A	

11	52.8 (6.5)	
6	N/A	

16	NA	

8	N/A	

27	47.5	

4	atients, they were all older between 74-90 years)	
8	N/A	

2	NA	

392	[illegible]3%), 50s: 157 (40%), 60s: 99 (25%), 70+: 26 (7%)	
11	N/A	

4	N/A	
	N/A	
8	N/A	

342	N/A	

13	Not reported	

8	N/A	

28	R = <35 to 55–65	

1004	N/A	
10	majority between 25 and 39 years of age	

Enter male (n/%) of HCPs	Male
N/A	
35/58 (60%)	
N/A	

18/24 (7/7 cardiologists, 2/3 internists)	

4 male (33%)	

5 male (33%)	
1 (20%)	1

1 male (20%)	
N/A	

N/A	
0 participants reported their demographics	3

2/6 (33%)	

NA	
7/11 (64%)	

Not reported	
N/A	
5/22 (23%)	

18 (39.1%)	

NA	
N/A	

Not reported	
N/A	

223 (72.4%)	

N/A	

7 participants reported their demographics	3

NA	
1 male = family physician	

N/A	

N/A	
2	

0 male (0%)	
N/A	

N/A	

N/A	

28 male (56%)	
2/6 (33%)	

Not reported	
NA	

1 (17%)	1

9 male (35%)	

2 (12%)	

0 (0%)	

N/A	
7	

N/A	
N/A	

5 (56%)	

N/A	

6 male /11	

N/A	

NA	

1 (5.88%)	
3/16 HCPs (19%) and 2/13 managers (15%)	

N/A	

2 (22%)	

1 (7.2%)	

14 (15.1% men)	

234 (82.4%)	
5 (63%) and for managers 2 (40%)	

N/A	
4/23 (17%)	
N/A	

N/A	
N/A	
1 male (10%)	
N/A	
N/A	

N/A	

9 (13.8%)	
N/A	

N/A	
only have the value including with patients)	

3 (27%)	

N/A	
6 male (86%)	

N/A	

4 (15)	

NA	
N/A	
9 male (33%)	

54/97 (56%)	
Not reported	
0 (0%) and focus group : 3 (12%) were male	

Not reported	

27/44	
N/A	

1/4 - 4/13 - 1/8	

1084 (62.4%)	

4 male (20%)	

55/117 (47%)	
11/19 (46%) in total, including stakeholders	

9/20 (45%)	

6 male (38%)	

9 (75%)	

Not reported	
4 (57%)	

N/A	
2 (15%)	

N/A	

0 (0%)	
4 male (44%)	

NA	
41 (73%)	

50%	

4 (19%)	

10 (57.6%)	

6 (55)	
N/A	

5/16 (31%)	

1/8 (12.5%)	

8/27 (30%)	

2 (50%)	
2 (25%)	

0/2 (0%)	

male 352 (87%)	
1 (9%)	

N/A	
N/A	
N/A	

255 (74.6%)	

Not reported	

N/A	

10/28 (36%)	

37.6% (378/1004)	
6/10 (60%)	

**Which variable was measured? (e.g., acceptability)**

(Question: At the level of individual people, are there any important disadvantages

(Question: In view of the large number of apps available that can be downloaded f

security concerns, trustworthiness of content, technical reliabilty of software, hyg
UTAUT = performance expectance (expected benefits), effort expectancy (expecte

Belief that wearables and mobile health applications are beneficial for both physic
Belief that wearables and mobile health applications are beneficial for both physic
Engaging with social media platforms to share medical information

Using social media platforms to share medical information with patients
Barriers to the utilization of DHT in Cardiology
Barriers to the utilization of DHT in Cardiology
Barriers to the utilization of DHT in Cardiology
Barriers to the utilization of DHT in Cardiology
Barriers to the utilization of DHT in Cardiology
Barriers to the utilization of DHT in Cardiology
Barriers to the utilization of DHT in Cardiology
Barriers to the utilization of DHT in Cardiology
Utilizing smart devices to monitor their own health
Using social media platforms to share medical information with patients
Barriers to the utilization of DHT in Cardiology
Barriers to the utilization of DHT in Cardiology
Barriers to the utilization of DHT in Cardiology
Barriers to the utilization of DHT in Cardiology
Barriers to the utilization of DHT in Cardiology
Barriers to the utilization of DHT in Cardiology
Barriers to the utilization of DHT in Cardiology
Barriers to the utilization of DHT in Cardiology
Barriers to the utilization of DHT in Cardiology
Facilitators of the utilization of DHT
Barriers to the utilization of DHT in Cardiology
Barriers to the utilization of DHT in Cardiology
Barriers to the utilization of DHT in Cardiology

Perception that the iPDAs helped patients understand the benefits and harms of t
Clinicians felt the iPDAs improved decisional quality by leading to greater congrue
Clinicians felt the iPDAs improved decisional quality by leading to greater congrue
Perception of patient satisfaction as perceived by clinicans to be higher when an i
Perception of patient satisfaction as perceived by clinicans to be higher when an i
Feeling that visits with an iPDA took a similar amount of time, or were more efficie
Feeling that visits with an iPDA took a similar amount of time, or were more efficie

Private practice vs working in an academic or university setting
Cardiac electrophysiologists vs other types of HCPS
lack of confidence in the accuracy of AF detection, detection of 30 seconds of AF

lack of confidence in the accuracy of AF detection, detection of 30 seconds of AF
Who would initiate OAC (oral anticoagulant) for a patient at an elevated risk for st
[illegible] diagnosed using a digital device in a patient with prior AF but not on OAC, such
In addition to a consensus document from your scientific society, which further do
In addition to a consensus document from your scientific society, which further do
3 most commonly cited challenges
“What additional steps are needed to encourage you to prescribe mobile or digita
Steps widely cited by survey respondents as influencing their decision to recomme
lack of confidence in the accuracy of AF detection, detection of 30 seconds of AF
3 most commonly cited challenges
Awareness of the existence of health insurance plans and subsidizing
“What additional steps are needed to encourage you to prescribe mobile or digita
Steps widely cited by survey respondents as influencing their decision to recomme
lack of confidence in the accuracy of AF detection, detection of 30 seconds of AF
HCP confidence by type of digital device used for AF detection
Steps widely cited by survey respondents as influencing their decision to recomme
3 most commonly cited challenges
Steps widely cited by survey respondents as influencing their decision to recomme
3 most commonly cited challenges
3 most commonly cited challenges
lack of confidence in the accuracy of AF detection, detection of 30 seconds of AF
lack of confidence in the accuracy of AF detection, detection of 30 seconds of AF

TM as familiar, TM value in their work, support TM in the future, TM can be easily

Satisfaction with current patient-to-staff ratio; connectivity, satisifcation with the

Behavioral intention of use, perceived usefulness, perceived ease of use, social inf
Behavioral intenion of use: "I intend to encourage patients to use the appointmen
Perceived Ease of Use: "It will be easy for me to post information on EPP.", "I will f
Perceived Usefulness: "Using EPP will give me greater control over my patient’s ch
Social influence: If my colleagues are using EPP and find it worth it, I would use it t
I would be concerned about the information privacy of my patients
Perceived Usefulness: "Using EPP will give me greater control over my patient’s ch
Behavioral intention of use, perceived usefulness, perceived ease of use, social inf
Behavioral intention of use, perceived usefulness, perceived ease of use, social inf
Behavioral intention of use, perceived usefulness, perceived ease of use, social inf
Perceived Usefulness: "Using EPP will give me greater control over my patient’s ch
Perceived Usefulness: "Using EPP will give me greater control over my patient’s ch

Physicians' attitudes towards the effectiveness of remote monitoring within the fr
Physicians' opinions on the most appropriate patient groups for inclusion in remot

Barriers and obstacles that, in the opinion of doctors, may arise if the issue of wid
Barriers: Lack of time for remote consultation, lack of confidence in collection and
Using the internet and other forms of remote communication with patients
Patient characteristics of who would benefit from telemedicine
Awareness of telemedicine
Readiness to transfer patients to a remote monitoring
Proportion of doctors from different age groups who have personal experience of
Perception that Amount of reference to ambulatory (out-patient) will be less; Rela
Importance of centralizing data
Perception of usefulness of remote monitoring
Perception that Amount of reference to ambulatory (out-patient) will be less; Rela
Perception that Amount of reference to ambulatory (out-patient) will be less; Rela
Perception that Amount of reference to ambulatory (out-patient) will be less; Rela
Perception that Amount of reference to ambulatory (out-patient) will be less; Rela
Using the internet and other forms of remote communication with patients
Patient characteristics of who would benefit from telemedicine
Preferences of digital technologies
Patient characteristics of who would benefit from telemedicine
Patient characteristics of who would benefit from telemedicine
Barriers: Lack of time for remote consultation, lack of confidence in collection and
Perception of usefulness of remote monitoring
Barriers: Lack of time for remote consultation, lack of confidence in collection and
Readiness to transfer patients to a remote monitoring
Barriers: Lack of time for remote consultation, lack of confidence in collection and
Barriers: Lack of time for remote consultation, lack of confidence in collection and
Barriers: Lack of time for remote consultation, lack of confidence in collection and
Barriers: Lack of time for remote consultation, lack of confidence in collection and
Barriers: Lack of time for remote consultation, lack of confidence in collection and

Reasons for clinical dissatisfaction

Cooperation with other workers in the pharmacy - whether it was easy or difficult
Time that pharmacists could spend on the assessment of a citizen in the pharmacy
Usefulness of the project "All in all, do you think, the project is useful?"
Cooperation with the other workers in the pharamacy
Acceptance evaluation

User satsification

How the health center initiated or expanded patients’ use of home blood pressure
Self-efficacy; outcome expectacies; perceived acceptability of the intervention to

What prevents you from using telemedicine?
quality of examination virtually; allowed HCPs to sufficiently address patients' clin

Background (experience, interest and conducting) telemonitoring
Younger age and higher probability on telemonitoring
Years of clinical practice and higher probability on telemonitoring
Country of residence and higher probability on telemonitoring
Multidisciplinary team working and and higher probability on telemonitoring
Practicing in big cities and higher probability on telemonitoring
Academic activities

Read scientific manuscript in last 30 days
Knowledge telemonitoring platform
Availability telemonitoring hardware
Judgment positive relevance of telemonitoring on own-practice compared to 1–5
How helpful could be telemonitoring to facilitate adherence to face-to-face recom
How many recommendations can be done by telemonitoring
Better way to connect to patients in telemedicine
Paid telemonitoring by insurance companies in your country
Telemedicine laws available in your country

Main barriers to the implementation of digital health applications/tools in clinical

Likelihood of using virtual visits as an alternative to in-person visit for most first vis

[illegible]
perception (β = 0.585) had significant positive effects on usage intention (p <

How was it measured? Please specify the exact measure (e.g., name of the questionnaire or tool used)
the Qualitrics© survey questionnaire was developed following the design method of Dillman et al. and was optimized for smartphone browsers; the answers were analyzed by the Mann-Whitney U test

s you see in mass community screening using wearable devices/apps?) Anxiety in people who test positive for AF, risk [illegible]

from Apple or Google play stores what is most important to guide the consumers?): randomized controlled trials of spe[illegible]

VII) Skala für Ärzte analog zu Albrecht 2017
UTAUT-2 based on Venkatesh 2012; e-health literacy according to Norman and Skinner 2006; Perceived privacy risk ac

online survey
online survey
online survey

online survey
online survey
online survey
online survey
online survey
online survey
online survey
online survey
online survey
online survey
online survey
online survey
online survey
online survey
online survey
online survey
online survey
online survey
online survey
online survey
online survey
online survey
online survey
online survey

survey
survey
survey
survey
survey
survey
survey

odds ratio (logisitic regression to evaluate the univariate association between having recommended a mobile device to
odds ratio (logisitic regression to evaluate the univariate association between having recommended a mobile device to
descriptive

descriptive
descriptive
descriptive
descriptive
descriptive
descriptive
descriptive
descriptive
descriptive
descriptive
descriptive
descriptive
descriptive
descriptive
descriptive
descriptive
descriptive
descriptive
descriptive
descriptive
descriptive
descriptive

Normalization Measure Development (NoMAD) questionnaire

Satisfaction level for various aspects of managing remote monitoring device clinics

using constructs of the technology acceptance model
using constructs of the technology acceptance model
using constructs of the technology acceptance model
using constructs of the technology acceptance model
using constructs of the technology acceptance model
using constructs of the technology acceptance model
using constructs of the technology acceptance model
using constructs of the technology acceptance model
using constructs of the technology acceptance model
using constructs of the technology acceptance model
using constructs of the technology acceptance model
using constructs of the technology acceptance model

online survey
online survey
online survey

online survey
self-constructed questionnaire
self-constructed questionnaire
self-constructed questionnaire
self-constructed questionnaire
self-constructed questionnaire
self-constructed questionnaire
self-constructed questionnaire
self-constructed questionnaire
self-constructed questionnaire
self-constructed questionnaire
self-constructed questionnaire
self-constructed questionnaire
self-constructed questionnaire
self-constructed questionnaire
self-constructed questionnaire
self-constructed questionnaire
self-constructed questionnaire
self-constructed questionnaire
self-constructed questionnaire
self-constructed questionnaire
self-constructed questionnaire
self-constructed questionnaire
self-constructed questionnaire
self-constructed questionnaire
self-constructed questionnaire
self-constructed questionnaire
self-constructed questionnaire

Early cooperative index

' (question E1) 'Did you usually have enough time to complete the project within the opening hours of the pharmacy?"

Final cooperative index

11-point, single-item Net Promoter Scale (NPS)

Frequency and percentage were used to summarize multiple choice question findings
self-report questionnaires, internally designed

Frequency and percentage were used to summarize multiple choice question findings

Patient and provider surveys were developed using questions from existing validated tools [24–27], as well as novel qu[illegible]

cross-sectional online survey
cross-sectional online survey
cross-sectional online survey
cross-sectional online survey
cross-sectional online survey
cross-sectional online survey
cross-sectional online survey

cross-sectional online survey
cross-sectional online survey
cross-sectional online survey
cross-sectional online survey
cross-sectional online survey
cross-sectional online survey
cross-sectional online survey
cross-sectional online survey
cross-sectional online survey

Weighted mean of each of the nine factors' contribution to difficulty in the implementation of digital health applicatio

Multiple-choice response questions

web-based questionnaire survey via Sojump

Quantitative Data
**Scale range (e.g., 0 - 100 on a visual analogue scale)**
2 scales: 1-3 and 1-5

of bleeding due to OAC (oral anticoagulation) against potential but unproven ben
ecific apps or devices, validation by scientific associations, more rigorous regulatio

nominal scale (1-11)
5-point Likert scale from 'strongly disagree' to 'strongly agree' for all 3 (UTAUT, [illegible]

n, %
n, %
chi-square test

chi-square test
n, %
n, %
n, %
n, %
n, %
n, %
chi-square test
chi-square test
n, %
n, %
n, %
n, %
n, %
n, %
n, %
n, %
n, %
n, %

5-point Likert scale (strongly agree to strongly disagree)
5-point Likert scale (strongly agree to strongly disagree)
5-point Likert scale (strongly agree to strongly disagree)
5-point Likert scale (strongly agree to strongly disagree)
5-point Likert scale (strongly agree to strongly disagree)
5-point Likert scale (strongly agree to strongly disagree)
5-point Likert scale (strongly agree to strongly disagree)

o patients for AF detection)
o patients for AF detection)

5 point likert scale

5-point Likert scale (strongly agree to strongly disagree)

Yes/No
Yes/No
5-point Likert Scale ranging from 'strongly disagree' to 'strongly agree'
5-point Likert Scale ranging from 'strongly disagree' to 'strongly agree'
Yes/No
5-point Likert Scale ranging from 'strongly disagree' to 'strongly agree'
5-point Likert Scale ranging from 'strongly disagree' to 'strongly agree'
Yes/No
Yes/No
Yes/No
5-point Likert Scale ranging from 'strongly disagree' to 'strongly agree'
5-point Likert Scale ranging from 'strongly disagree' to 'strongly agree'

n, %
n, %
n, %

n, %
n,%
n,% (choice between 'no I cannot do it', 'yes' and 'hard to tell')
n,%
n,%
n,%
n,%
n,%
n,%
n,%
n,%
n,%
n,%
n,%
n,% (choice between 'no I cannot do it', 'yes' and 'hard to tell')
n,%
n,%
n,%
n,%
n,%
n,%
n,%
n,%
n,%
n,%
n,%
n,%
n,%

0 to 5, higher values meant better cooperation with coworkers
0 to 4
scale from 1 to 10, where 1 point means no acceptance and 10 full acceptance

11-point “How likely is it that you would recommend the system to a friend or co[illegible]

NA
1-5 or 1-10

NA
5 likert scale (strongy agree to strongly disagree)

n, %
bivariate logistic regression
bivariate logistic regression
bivariate logistic regression
bivariate logistic regression
bivariate logistic regression
bivariate logistic regression

bivariate logistic regression
bivariate logistic regression
bivariate logistic regression
bivariate logistic regression
bivariate logistic regression
bivariate logistic regression
bivariate logistic regression
bivariate logistic regression
bivariate logistic regression

1=not at all important, 2=not very important, 3=neutral, 4=very important, 5=ex

Yes/No-type questions

items were measured with a 5-point Likert-type scale ranging from 1 (strongly di

**Mean (SD) of the score**
cardiologists found that patients were able to communicate effectively

65%, 39%, 41%, 20%
45%, 41%, 25%, 26%, 32%, 4%, 8%

The main concerns were about the secu
[illegible]<br>performance expectancy had a

[illegible] believed that wearables and mobile
[illegible] believed that wearables and mobile
[illegible] with social media platforms to share

Similarly, those who reported using soc
Increased workload and
Increased workload and
adaptability (200/245, 81.6%), low
Increased workload and
Increased workload and
devices to monitor their own health
Similarly, those who reported using soc
Increased workload and
Additionally, the cost of technologies
Increased workload and
Increased workload and
Increased workload and
health activities showed a greater
Increased workload and
Increased workload and

98.1% agree or strongly agree
93.50%
93.50%
82.6% agree or strongly agree
82.6% agree or strongly agree
73.80%
73.80%

[illegible] (95% confidence interval: 1.22–2.54; P
[illegible] more likely to recommend a digital
lack of confidence in the accuracy of AF

lack of confidence in the accuracy of AI
Less than half of those surveyed (42.7%
(88.5%) of respondents
More than half of respondents reporte
More than half of respondents reporte
“potentially unclear or noisy results” (5
(27.3%) of participants had no reservat
[illegible]<br>(36.7%), legislation clarifying medical
lack of confidence in the accuracy of AI
“potentially unclear or noisy results” (5
Only 15.9% of participants were aware
(27.3%) of participants had no reservat
[illegible]<br>(36.7%), legislation clarifying medical
lack of confidence in the accuracy of AI
Whereas only 26.8% of respondents re
[illegible]<br>(36.7%), legislation clarifying medical
“potentially unclear or noisy results” (5
[illegible]<br>(36.7%), legislation clarifying medical
“potentially unclear or noisy results” (5
“potentially unclear or noisy results” (5
lack of confidence in the accuracy of AI
lack of confidence in the accuracy of AI

[illegible] their work (5/6, 83%) , they would

[illegible] satisfaction with current patient-<br>to-staff ratio; 88% perceived

Behavioral intention of use (73%), perc
"I intend to encourage patients to use t
Mean (SD): 3.5(0.6) and 3.2 (0.9)
Mean (SD): 4.0 (0.6) and 3.3 (1.1) and 3
n = 12 (70.6%)
Mean (SD): 3.6(1.1)
Mean (SD): 4.0 (0.6) and 3.3 (1.1) and 3
Behavioral intention of use (73%), perc
Behavioral intention of use (73%), perc
Behavioral intention of use (73%), perc
Mean (SD): 4.0 (0.6) and 3.3 (1.1) and 3
Mean (SD): 4.0 (0.6) and 3.3 (1.1) and 3

reduce the number of visits (64.5% agr
Young age (55.9% advisable, 41.9% not
[illegible]<br>took part in the survey were

the majority of respondents (80.6%) na
Lack of time for remote consultation (7
More than 2/3 respondents used in the
The majority of physicians considered i
The survey showed that 20.9% of the d
answered affirmatively to the
increasing age, the proportion of
Thus, in general, 60.8% of respondents
Doctors of the youngest age (37.1%
Every 5th respondent (22.5%; 9 doc
Thus, in general, 60.8% of respondents
Thus, in general, 60.8% of respondents
Thus, in general, 60.8% of respondents
Thus, in general, 60.8% of respondents
More than 2/3 respondents used in the
The majority of physicians considered i
The most preferred means of communi
The majority of physicians considered i
The majority of physicians considered i
Lack of time for remote consultation (7
Every 5th respondent (22.5%; 9 doc
Lack of time for remote consultation (7
answered affirmatively to the
Lack of time for remote consultation (7
Lack of time for remote consultation (7
Lack of time for remote consultation (7
Lack of time for remote consultation (7
Lack of time for remote consultation (7

Lack of data resulting in 'barrier to titra

4.34 (indicating that supporting factors<br>[illegible]
cooperation with other healthcare
Negative in 34%
[illegible]<br>believed that it was useless, whereas
3.02 (*here only 51 pharmacists filled c
Median (IQR) 10 (8-10)

[illegible] likelihood that each user group would

"My health center began distributing h
Prescriber data, after training: self-effic

"Administration" (26 (27%)); "Present i
[agree and strongly agree added togeth

[illegible] telemonitoring; almost half of the
[illegible] 0.99-1.59) and p=0.055
[illegible] 0.93-1.48), p=0.17 (not significant!)
[illegible] 1.87), p=0.005
[illegible] (95% CI = 1.42-2.35), p<0.001
[illegible] 1.27-2.17), p<0.001
[illegible] CI = 1.23-1.89), p<0.001

[illegible] (95% CI = 1.17-2.22), p=0.003
[illegible] OR = 2.62 (95% CI = 2.03-3.39),
[illegible] OR = 5.52 (95% CI = 2.78-10.9),
[illegible] 3.33-5.96), p<0.001
[illegible] 3.40-7.10), p<0.001
[illegible] 2.57-4.22), p<0.001
[illegible] = 0.72-1.42, p=0.98 (not significant!)
[illegible] p=0.006
[illegible] p=0.005

Lack of motivation among health profes

[illegible] virtual visits for most patient follow-

Attitudes about AI in cardiovascular car

isagree) to 5 (strongly agree)

**Where in the article is this result reported? (e.g., table 2, page 7)**
table 4 and results text

results
results

page 8

table 4

pages 46-48
pages 46-48
pages 46-48

pages 46-48
pages 46-48
pages 46-48
pages 46-48
pages 46-48
pages 46-48
pages 46-48
pages 46-48
pages 46-48
pages 46-48
pages 46-48
pages 46-48
pages 46-48
pages 46-48
pages 46-48
pages 46-48
pages 46-48
pages 46-48
pages 46-48
pages 46-48
pages 46-48
pages 46-48
pages 46-48
pages 46-48

page 600
page 600
page 600
page 600
page 600
page 600
page 600

pages 23-24
pages 23-24
pages 23-24

pages 23-24
pages 23-24
pages 23-24
pages 23-24
pages 23-24
pages 23-24
pages 23-24
pages 23-24
pages 23-24
pages 23-24
pages 23-24
pages 23-24
pages 23-24
pages 23-24
pages 23-24
pages 23-24
pages 23-24
pages 23-24
pages 23-24
pages 23-24
pages 23-24
pages 23-24

page 4

page 4

used Figure 4 multivariate regression results

pages 3-5
pages 3-5
pages 3-5
pages 3-5
pages 3-5
pages 3-5
pages 3-5
pages 3-5
pages 3-5
pages 3-5
pages 3-5
pages 3-5

page 12

page 12

page 11

page 12
table 3
Figure 1
table 6
page 31
Figure 3
figure 2
Table 4
figure 5
page 33
Table 4
Table 4
Table 4
Table 4
Figure 1
table 7
table 2
table 6
table 6
table 3
page 34
table 3
Figure 3
table 3
table 3
table 3
table 3
table 3

page 2

pages 4-5
pages 4-5
pages 4-5
pages 4-5
page 77

table 2 and 3

page 7

Table 3

Page 10, table 6

Table 2

figure 2

page 240
page 241
page 241
page 241
page 241
page 241
page 241

page 241
page 241
page 241
page 241
page 241
page 241
page 241
page 241
page 241

Table 3, Table 4

page 1296 and Fig 3

Figure 3 and 4

Table 5 (p.8)

**What data analysis was conducted (e.g., thematic analysis)**
Thematic content analysis
Thematic analysis

thematic analysis (enhanced with a method used by Lincoln &Guba)

Framework analysis

Thematic analysis according to Braun and Clarke
Thematic analysis (Braun and Clark).

Conventional content analysis mapped onto the theoretical framework by Chaudoir (innc
thematic analysis using the method described by Braun and Clarke

informal feedback
A hybrid qualitative approach integrating a priori and emergent themes. A priori subjects

Thematic analysis

Thematic analysis
Inductive thematic analysis

Thematic analysis according to Braun and Clarke, theoretically informed by the Technolog
Thematic analysis

Thematic analysis
Most likely descriptive

Consensus qualitative analysis according to Richards and Hemphill
Qualitative thematic analysis [using concepts of feasibility, acceptability and effectivenes

thematic framework analysis was employed to extract themes (involving steps such as fa

Inductive thematic analysis (Braun and Clarke's six-step process)

Thematic analysis
initial round of inductive thematic analysis to elicit main themes from the interview data

meta-analysis

Thematic analysis of written responses
inductive content analysis according to Graneheim and Lundman

Thematic analysis

thematic analysis of the interview summaries related to the 3 evaluation goals

transcripts were thematically analyzed using an inductive coding approach

Thematic analysis according to Braun and Clarke
Thematic analysis

Thematic analysis
Thematic analysis

Thematic data analysis. Themes were identified using an inductive data-driven approach

Deskriptive statistical analysis (content analysis)

[illegible]
based on the behaviour change wheel (BCW) 1.) Motivation 2.) Opportunity and 3.)

Interpretative description approach, which was then used to dedutively analyze a detaile

thematic analysis
analysed thematically using NVivo V.12 software to help organise, code and explore the [illegible]

combination of inductive coding and template analysis
Thematic analysis using an interpretive descriptive approach from interviews and observe

Deductive content analysis

Thematic analysis

thematic framework analysis

thematic analysis was performed on the qualitative survey responses; CV Wizard use and

Thematic analysis

Thematic analysis

Analyzed according to the Framework Method for analysis of qualitative data

inductive thematic analysis

Thematically analyzed by means of a constant comparative method using deductive and i

Thematic analysis using inductive coding and evaluated through a process of 'axial coding

Thematic analysis
thematic analysis on patient transcripts and clinician transcripts separately

Thematic analysis and the matrix method
Thematic analysis according to Braun and Clarke
Descriptive?
Manifest qualitative content analysis

interviews were analyzed using team-based rapid content analysis

Thematic analysis using a deductive, semantic approach

thematic analysis
Combination of inductive and deductive approaches was used for the analysis. They used

Conventional content analysis (inspired by Graneheim and Lundman)

not explained - most likely descriptive?
Inductive qualitative approach based on grounded theory

Content analysis - as inductive analytic method

(inductive) thematic content analysis

Thematic analysis
N/A (descriptive?)
Reflexive thematic analysis

Thematic analysis

Interpretive Description approach . An initial codebook was generated deductively on
the basis of the National Academy of Medicine Six Domains of Health Care Quality

Not reported / not applicable

Deductive and inductive thematic analysis
Qualitative content method using a framework for the analysis by deriving the interview

descriptive data analysis

Phenomenological thematic analysis

Thematic analysis

Thematic analysis

Content analysis

Thematic analysis (Braun and Clarke)

"basic content analysis approach"
Thematic analysis using a deductive process

All the open-ended responses were coded using an interpretive-descriptive approach.
Deductive TDF (theoretical domains framework) approach

grounded theory approach for the analysis

Conventional content analysis
"iterative, inductive thematic approach"

Thematic analysis (with an initial i-PARIHS theoretical constructs (coding))
An iterative approach was used for data analysis based on the framework method for qua

thematic analysis

Content analysis

Content textual analysis - Use of a conceptual map to quantify and display participants' p

Deductive thematic analysis,
Deductive thematic analysis (using the [illegible] framework)

Thematic analysis

inductive analysis

Hybrid inductive/deductive framework analysis

inductive content analysis
thematic analysis (Braun and Clarke) (inductive)

Thematic analysis

Descriptive analysis (n, %)

Analysis was informed methodologically by the constant comparative approach and theo

N/A
Descriptive reporting of a mixture of Likert scale and open-ended questions
reflexive thematic analysis

N,% and X2, Mann–Whitney and one-way analyses of variance as required.

"inductive thematic analysis at the semantic level according to the iterative 6-phase appr

inductive thematic analysis using Braun and Clarke’s iterative six-phase approach

Inductive analysis creating codes and then overall categories

deductive content analysis

Qualitative Data
**What are the facilitators to DHI implementation (please list them, separate them by semicolon, if necessary specify**
[illegible] many found it be one of the greatest strengths of the project. Nurses believed it particularly helped the patients
Perception that home blood pressure gives a more accurate reflection of one's true blood than clincial blood pressure

findings also showed a growing acceptance rate among patients experiencing telehealth solutions for the first time an

[Doctors] felt that they would be more willing to recommend apps that had been scientifically tested, were based on [illegible]

DHI functions/features perceived as helpful by HCPs; helpful visualisations of data: "The professionals considered the
[illegible] appropriate interventions for abnormal BP readings: providers indicated that paients at risk for preeclampsia require

HCPs perceive the DHI as clinically useful ("Perhaps most importantly, the usefulness of the intervention had a crucial
involve patient and nurse (end users) in platform development and testing stage of smart RM technologies; including

[illegible]
was doing an important thing and most people too liked me when I shared with them the test results.'

[illegible]
you in three months, you're not going to think about doing your blood pressure. Maintaining, pushing a little more

Convenience of telehealth visits (patients living in rural areas, having issues with transportation or mobility challenges

The anticipated benefits (e.g., reduction in inappropriate telemetry monitoring use, improvement of quality of care an

Telehealth for HF: A health care professional (e.g., a nurse) trained in telehealth should be designated to pick up
[illegible]

Availability/accessibility of required technology to patients ("HCWs also confirmed that mobile phones are widely acce
Potential for educating and prompting patients and family members on strategies for self-monitoring and self-manage

used signficant variables of the multiple hierarchical regression mode

Despite the challenges of implementing the tool, support by clinical champion (who sent regular emails and made sur[illegible]
Greater access to evidence-based programs: one provider noted that the programme provided access to an evidence-[illegible]

HCP perception of clinical benefits of the DHI; HCP perception of benefits to the patient-provider relationship through
[illegible]<br>for other stuff I mean, we spend a lot of time…

to promote app engagement-interactive app features (symptom tracking, patient tracking, health tips, patient

Working with insurers to accept new models of care; Incorporating family members to help out with patients' lack of t

HCPs prefer an e-health tool integrated with eHealth record as they perceive it more accurate and more useful; HCPs
patients feel reassured that someone is monitoring (gives sense of security, support and feeling of tailored g

patient decision aids are information sources (such as pamphlets, videos, or web-based tools) designed to support act

[illegible]
(shared decision making). One clinician wrote that the iPDA ‘‘(b)rings in the best available evidence to support shared

no need to commute long distance to hospital/transportation issues; increased accessibility for patients with

Institutional and cross-sectoral buy-in ("...implementation would need to be a team effort and would require “buy-in”

display of patient-contributed data alongside clinician-recorded (this data approach enhanced data interpretation and

ease of use = more likely to engage patient; habit/structure formation in the convenience and comfort of the

Devices that measure accurately; devices that collect data continuously (as opposed to intermittently); devices that h
The virtual care model offers flexibility, which enhances patient attendance and engagement in their rehabilitation act

Possibility to implement a personalised approach according to patient needs ("A ‘one size fits all approach’ was not co
Presence of social support within a patients’ life, in the form of children or relatives who were available to support nav

communication ability in the form that clinicians considered helpful to send motivational messages to patients, reminding them to maintain self-monitoring, or asking them about signs and symptoms ("I think that if we have

HCPs perceive a clinical benefit of the DHI ("Facilitates innovation in health and documentation of evidence that trans

[illegible]
office visits currently is not reimbursed, so having some reimbursement for that kind of work would be helpful.—I

[illegible]
used a TM system based on health coaching, allowing them to compare the new TM platform to their previous

reduce delays in diagnosis (early recognition, enhanced symptom capture/education) and lead to better patie
patients with stable/well-managed conditions (and who had an established relationship with the clinicians), remote co

limited time during clinic visits; competing health conditions, long intervals between consecutive appointments; logist
Perception of better integration ("'Well, I already do home BP (blood pressure) monitoring with my patients, but the system doesn't work quite so well, so I thought this looked like a more efficient system' (GP)") ; Automatic workflow

[illegible]
them isn’t it and it’s giving them, well it’s giving thema voice, it’s allowing them to actually make a choice without

Optimizing alert transmissions, increasing remote staffing, and utilizing third-party platforms. Alert transmissions wer

non-clinicians were all mainly positive about screening for AF because it gave them an opportunity to develo

likelihood of system use was significantly higher in encounters with patients with higher CVD risk compared to those w

Anticipated clinician benefit motivated use of the DHI (controlled BP, reduced emergency department visits, greater m

[illegible]
computerized tool could be also used for assessing and monitoring patients’ progress more easily; Provides a sense

[illegible]
All members praised their orientation; all benefited from 1-on-1 approach to observing and then undertaking

[illegible]
Patient education is fundamental to enable successful self-management. However, people with HF may only get a

Perception that patients bear the responsibility to collect reliable data, meaning that they should measure every
weekday, at the sametime with the same medical device. A few argued that it is also the patients'responsibility to

healthcare professionals.

Remote care is one of most important benefits of mHealth. The ability to monitor patients between hospital visits

Need for surveying patients and conducting a pilot study to have a better understanding of feasibility and acceptabilit
support for patient; many appreciated the lottery intervention =may have motivated/increased adherence during the

Staff enjoyed the team dynamic ACDC (Advanced Comprehensive Diabetes Care) fostered, particularly supporting coll
HCPs perceiving the benefit of DHI for increasing access to services (e.g., in rural areas): ("Although some participants
According to physicians, in 99 consultations (99%) communication with the patient was effective. It was deemed unsa
[illegible] perception from the GPs was that patients generally had great confidence in the Holter monitor and the

infrastructural support was the primary facilitator for providers; improve access to care for veterans who live remotely

Another theme of the focus group was that education scenarios could support patients in practicing decision-making related to HF self-care. Clinicians described at length the complexity of self-care decision-making and acknowledged

providers indicated that access to historic patient information was easier, benefiting NCD control and follow-up; stanc

[Digital aspects of home blood pressure monitoring] The availiablity of phones: Participants noted that nearly all

[illegible]
especially for patients who live further away or have limited mobility (cardiologist, "at selected cases, it can obviously

Technology – Program financial reimbursement, tech support, training for staff & patients, increased patient access in rural areas, easy to use for patients and staff, secure and private

Good "task-technology fit", i.e., HPCs perceive the DHI as valuable in practice ("Acceptability of the prediction tool wa

[illegible]
and a reason if they do not engage in PA (e.g., boredom, pain, dizziness); Perception that alerts help prompt

Ease of use, speaking of the simplicity of the Guardian interface and feeling confident about navigating the system after a short while. Participants also commented on their perception of Guardian as an aide to day-to-day nursing

Combining telehealth with SMBP increases their use reciprocally (patients do check in more frequently regarding their
[illegible] cardiologists ([illegible]) stated a preference to have the data available at the time of the patient visit rather than 2 weeks after the visit or in between visits.
Dedicated time for HCPs to deliver the DHI ("Having designated time helped supporters manage this task."); HCPs rece

Quick and easy for the HCP to deliver the DHI ("It was emphasised that the practicality of using mHealth in primary ca[illegible]
[illegible]<br>follow-up appointements, patients and providers perceived virtual visits to be as safe and clinically effective as in-

HCPs' ability to access DHI data remotely ("However, having the ability to access data remotely offered several benefit

availability of an HMIS = helps efficiency; all public health facilities are mandated to submit healthcare utilisation data

Data management as well as job quality seems to be the most relevant factor considered as the IoHT driver. When we asked doctors about implementation drivers of IoHT, we found as main topics knowledge management issues

digital health in addressing the unmet needs of post-MI care; incorporating simple visuals and games with a

used significant variables of the mulitvariate analysis from table 4

Interoperability of the DHI with electronic health record and other clinical systems; Interoperability of the DHI with ot

[illegible]
agreements with external partners in the region are essential for establishing the digital infrastructure;

Good communication with NHSE & within integrated care systems (frequent opportunities to share information, discu

HCP perceive the DHI to address a clinical need, e.g., the need for patient education ("HCPs indicated a need for educa

[illegible]
and by making decisive decisions regarding others and booking them for surgery. In addition, the healthcare

easy-to-use and unser-friendly digital platform; provision of a physical setup with digital technology ready to use; adap
[focus was on identifying research priorities in CV] The use of e-health technology was raised as a research priority, especially in the facilitation of communication between patients and healthcare providers ; Other priorities for CVD

bi-weekly phone calls and educational content aligned with the needs of their patients; maintain or improve health th

[illegible]
much about AF-related risks prior to the screening initiative; Clinical pharamacists perceived the intervention

faster and more reliable risk stratification, diagnosis of rare or commonly missed conditions; increases patient engage

[illegible]
significantly affected the quality of care that they delivered. A few participants reported that patient adherence to

Useful features of the DHI based on electronic health record integration; time-saving features of the DHI; ("Specifically

The DHI would be more trustworthy and more actionable by RNs and MDs if clinic leadership explicitly embedded it in

Physicians thought that implementing the C-QI (collaborative quality improvement) strategy would standardize
treatment protocols and reduce medication errors as they will receive alerts or clinical decision support prompts for

telemedicine changed how they spent their time in a clinic = consultations were overall shorter, and time was saved b

Participants also identified that adoption of apps required a certain level of motivation from patients and that those who are highly engaged in HF self-management would likely benefit from using such tools ; Participants felt that apps

Perceptions of ICT (information and communication technologies) in daily practice as "important" and "essential".
[In a concept map that reflects the future relationship and opportunities that healthcare professionals consider

[illegible]
intervention to support chronic disease management ; mHealth messages are seen not just as ways to educate,

[illegible]
governmental organization) may have been more likely to receive messages by answering the phone when

HCPs had confidence that the recommendations from the CDS tool were accurate knowing they were based on the lat

convenience (helps with travelling, waiting time, especially for working individuals) ; improvement in patient-provider

avoids inconsistent participation (much more flexible, less issues with geographical distance because it extends the ge

[illegible]
despite individual capabilities. (barrier - For the physiotherapists, the individualization seemed double-sided as it was

[illegible]
programmes were unavailable.("‘there is, for certain groups of people, a benefit for this sort of an approach… a lot of

User-friendly device; A structured and personalized programme; Sharing and discussion of experiences; Prompt and a

HCPs perceive patient benefit ("Patients can receive better care at home through daily monitoring", 39%; "Patients ca

[illegible]
("I guess they need a more educational approach...that’s the kind of need to explore why they are choosing not to

Value of fewer clicks for patients: The cardiologist was given remote training on Zoom by the researchers while they si
All clinicians agreed (or strongly agreed) that the app improved their efficiency in caring for their patients; Incorporati
All the clinicians who were interviewed expressed the need to have the TM system integrated with the electronic heal

Insurance companies offering support (“Major insurance companies and Medicare have relaxed regulations and are n[illegible]

HCP perception about patients' digital health literacy / suitability for DHI ("With the limitations created by COVID-19, c

availability of real-time data and remote support = allow clinicians to modify patient care regiments on a more preven

Intervention was facilitated by pharmacists in the community (pharmacists should be primary responsible and should

quality indicators of the EPI RxISKTM calculator that facilitate end- users’ interaction with the application and motiva

What are the barriers to DHI implementation (please list them, separate them by semicolon, if necessary specify wh
[illegible] Other nurses reported that there were signal (connection) problems"<br>Electricity was also a concern, and one nurse recounted that when a community member would go to the market,
No standardized way to collect and manage PGHD ; The health care team identified data accessibility at the point of ca

Additional data and further evidence is needed to support the benefits of telehealth (many are mainly unregulated an

Older age, low education level: "Among doctors, users' ages and educational levels were considered the most influent

Professinals judgement that DHI is time-consuming and not worthwhile: "The professionals’ opinions about how feasil

[illegible]
financial barriers (eg, Financing/cost of a home BP monitor) to enhance patient BP self-monitoring. ("Some people

Negative impact on HCPs' workload/time resources ("Provider-level factors, namely the intervention’s impact on the c
increased workload/responsibility for HFSNs (difficulty to manage additional alerts and time-consuming in addition to

[illegible]
deterred from participating in the study because of the requirement to sign an informed consent form: ‘[they] did

[illegible]
trackers, tracking notices, now every single day. The patients have those tracking information back for me. But if I’m

Teleheath visits cannot substitute all in-person visits (meeting with a new patient, patients with multiple chronic cond

Disagreement among clinicians on whether they should reduce telemetry monitoring ordering or duration (for some, [illegible]

Telehealth for HF: Increased workload of primary care providers; Uncertainty around clinical safety and whether the
[illegible]

Older age; illiteracy; digital illiteracy ("HCWs added that such a programme could be more effective in urban areas and
Perceived lack of evidence of safety for patients and of value added beyond current remote patient monitoring that w

Lack of evidence of the added value supporting its use; Few-patient suitable and applicable; Some HCPs would like mo
[illegible] sufficient clinic space for the group

Additional workload through DHI-generated data ("Data review and patient follow-up through digital health, however
The stakeholders also indicated a few notable reservations, including the accuracy of readings, patient adherence, and a desire for data to transfer directly from

concerns that some users might be less motivated to use the app, especially for an extended period of time

[illegible] comprehensive Financing/cost-effectiveness (low Financing/cost of telehealth meaning that it's priortized just

Some HCPs have concerns over confidentiality and privacy with the DHI being linked to EHR; HCPs worry that it would
telemedicine intervention depends heavily on human factors such as trust (interpersonal, institutional, human-techno

communication difficulties as a barrier to implementing absolute CVD risk assessment guidelines; lack of tim

NA
lack of infrastructure, incentives, clarity in regulations, knowledge, and training in digital tools; most providers

HCPs' perception that DHI reduces important face-toface interaction time ("A common theme regarding disadvantage

workflows for interpreting large volume of patient-contributed data (could overwhelm data that requires immediate a

Financing/cost of BP devices/medication remains an encumbrance (and insurance policies were considered to be a po

Poor accuracy of measurements taken with the device/DHI; older age of patients ("Difficulty in use due to user not be
Challenges in communicating through the virtual model with healthcare professionals in other settings and those acro

Lack of time; Other work commitments; Poor integration of the DHI into the usual clinical processes ("Health care prof
Patients’ limitations in digital access and literacy (e.g., lack of access to a smartphone or computer, lack of familiarity,

According to professionals, patients with a stable or long-time condition would be less prone to engage in a new strategy with mobile apps to manage their condition, especially if they are not used to technology ("They [older

[illegible]
of mobile health apps; Poor interoperability/connectedness with other digital systems ("Risk of not sharing the

[illegible]
problems of SMBP, "So, I don’t think physician incentives really play a role. Patient incentives— now, that’s a

Some staff members felt that their complex patients may be apprehensive about TM technology. Clinicians wondered if their patients would use it and what kinds of benefits might be realized ("I think because we’ve had a

challenges of determining who would receive regularly symptom reports and ensuring all members of the patient’s ca
loss of visual cues (including from subtleties of patient’s demeanour) meant some patients felt they could no

inadequate patient engagement (adherence to medication and HBPM); inability to monitor home BPs or adherence w

Perception that [illegible] is complex (‘ I was in a really busy period and it was...it is a lot of information you get, and even
though somebody came out to show me, it was still a bit of a hurdle to actually get going.’(GP)) ; and that telehealth

[illegible]
was I had to put the slash in as well. Whereas on a normal screen you would have the date of birth, you put the first

[illegible]
managing remote data and included issues such as troubleshooting home monitors, managing disconnections in a

resistance if it affected the time-constraints, increased workload and disrupted workflow (which has already worsened

lower system use when providers were >10 minutes behind schedule (RR 0.96, 95% CI 0.93–0.98, $p < .01$); participants

Patients’ lack of digital resources and digital literacy; Technology-related barriers (e.g., WIFI connection, problems pai

[illegible]
supply patients with pedometers and lack of patients' willingness to purchase their own devices); The limited ability

Challenges of patient enrollment.
All team members felt confident in being independently able to enrol a patient after 2–4 attempts. When asked to

[illegible]
so much aware of the technology, and definitely, there will be (…) internet issues and problems with computers and

[illegible]
abilities to measure and monitor their own symptoms (""“In my experiences, some people simply don’t send in their

[illegible]
Mentioned that all patient-generated data have to be analysed, to not miss clinically relevant events. This might

Patients may feel bored, turn off the device or ignore repeated messages over time; Poor telecommunications covera[illegible]
number of alerts was burdensome (and puts more responsibility on clinicians); responses to alerts varied considerably[illegible]

Staff acknowledged that ACDC (Advanced Comprehensive Diabetes Care) did increase their workload.

HCPs' concerns that DHI is a lower-quality substitute for face-to-face services ("Health professional #3 expressed ‘cond

limitations in terms of physical examiniation ; technical inabilities ; lack of devices for video-consultations and deafnes

One of the issues is that patients with suspected AF must appear several times in the hospital in connection with the 2

technology was the primary barrier for both patients and providers (discomfort with technology used to access telehe

[illegible]
adequate breadth and depth of Web site content to meet the diverse learning needs of patients with HF. Nurses

several key indicators critical to diagnosis were frequently not captured or recorded in open-text; increased time requ
[Digital aspects] Overall low literacy levels in the community could pose a challenge for some patients to participate

[illegible]
being late or on time, waiting and logging in) and senses (hearing and seeing). Technical issues sometimes led to

[Resource barriers – technology] tele-rehabilitation program not set up, lack of equipment, patients lack access to technology, lack of internet connection ; [Greater demand on staff time] increased time to deliver individualized

Misalignment between HCPs' expectations and DHI functionalities ("In cases where misalignment emerged between t

[illegible]
the suggested features: None reported using the cardiac rehabilitation referral option in the order, stating they

[illegible]
trend monitoring during the simulations ; Most participants commented that they needed additional prompting to

Lack of patient access to devices or device coverage and difficulty getting devices to patients; Challenges around ensu
NA
HCPs' concerns about algorithm-driven decision-making (in the DHI) and/or one-way digital communication via email t

/

[illegible]
compromised in the virtual environment because they have less information on which to base their clinical

DHI not user-friendly for HCPs; DHI not efficient for HCPs within thier patient management workflow ("Despite such ef

inadequate monitoring and evaluation frameworks for hypertension and T2D programmes; lack of interoperable e-hea

[main cultural gaps related to the adoption of IoHT]. From the doctors' point of view, they found that age and
personal attitudes as main issues. One doctor said: “these new methods [IoHT] are considerably increasing and the

lack of technology usability and user involvement during the design of the technology; (variability) user’s technical abi

Lack of wireless access; End-user errors; Difficulty integrating DHI into clinical workflow ("A few providers mentioned t

It might provide more work due to different approaches per patient; Implementation of extra digital dashboard alongs

Shifting directions & lack of national guidance; Lack of human, time resources (capacity in primary care is limited and [illegible]

HCPs perceive patients old age as a barrier ("The most prominent concern of 63% (10/16) of HCPs was related to the g

The primary concern of telehealth systems or remote health is privacy and accessibility issues.

Certain patient groups unsuitable for the DHI (virtual visits); the DHI (virtual visits) does not allow for necessary clinica
NA

not being able to learn in person ; lack of in-person contact; language of educational materials sometimes being difficu

Perception that the public often view pharamacists as 'shop assistants' rather than healthcare professionals (HCPs), w

substantial heterogeneity in the computational configurations and team structures used for AI clinical decision suppor

[illegible]
tele-rehabilitation. This made recruitment for virtual programming difficult and limited access for patients that would

NA
[illegible] emerged as a major deterrent to the use of EHR-DSS by the providers. One physician stated: ‘Majority of them [EHR-

more administrative time was required for appointments = increase in workload due to lack of interoperability betwee

[illegible] patients with HF were older

Problems associated with the manual insertion of data into a mobile application by patients; Perceptions of issues reg

[illegible]
they have access to a phone ; sharing phones meant that the targeted MoPoTsyo participant was not always near the

[illegible]
case their number would not work when the INSTEDD system tried to call) and the patients not updating new cell

With multiple modules available, prompt fatigue was highlighted as a barrier, especially with time constraints during c

technical challenges with usability of equipment (which takes more time to adapt to it ,making it less feasible to imple

usually greater uptake in pulmonary rehabilitation (more suitable for telehealth models) than in cardiac rehabilitation;

[illegible] technical problems during the web-based training sessions, such as problems with logging in and handling the sound
Perceptions of users’ digital literacy as a potential barrier, recognising a perceived age-related digital divide and unsup[illegible]

Technological difficulties (not suitable for the elderly); Insufficient guidance or overwhelming information; Aversion to

Overly complicated system (14%); [Lack of] Widespread use of electronic medical records (24%); No incentives in heal
old age and non-use ("The caveat I suppose might be that those that tend to have the chronic diseases tend to be the<br>older age group so they maynot be so tech savvy. We've got some patients who don't use mobile phones even now.

NA
[Ineffective user interaction] Experience that workloads did not decrease with a TM (telemonitoring) system (""I think

[illegible]
guidelines and reimbursement proceedings. (""[..] private insurance company rules vary by company and by state, so

Enrollment in the DHI without receiving a contextual introduction to the DHI from the relevant HCP ("Overall, the clini

digital therapeutic program would only be valuable if it was customizable to the patient’s changes in lifestyle, age rang

Challenges with the feasibility of individualized medication lists being used for SDMG and how to ensure the accuracy

having to manually input patient data in the application was described as time-consuming;

**Where in the article is this presented?**
pages 5-9
pages 114 - 116

p. 4-7

Main text pages 7-9, Figures 4 and 5

Results section pages 5-9, Discussion section pages 9-10
pages 3 - 6

Discussion section pages 6-7
p.5-7 (table 2 updated from Hannah)

page 382
pages 4 - 8

p. 5, 7-10

p. 9-11
Section 6.3, Section 6.4, Table 3

Results and Discussion sections, pages 4-8
Table 1

Table 7
page 5

Results section pages 4-6
pages 7 -9

p.5-12

Figure 1

Table 2, p. 6-9
p.5-11

p.5-7

page 600
p.4-10

Results section page 351

p.4-6

p.4-8

Results pages 3-4
Figure 1, p. 4-6

Results section pages 8-9
Table 5, p. 9-10, 12-13

pages 4 -6

Results page 8, table 2

pages 7-10

pages 5 - 8

p.7-9 + 11-12
p.3-8

p.3-8 (table 2 - updated by Hannah)
S3 Text, S9, and S10

Supplementary table S5

pages 4-5

p.8-11 + 13-14

p.7-8

Table 2, p. 3-5

p. 7-8

pages 5-8

pages 3-5

pages 83-84

Supplementary table 1 and pages 186 - 188

Table 2, p. 2675-2677
p.3-7 (p 5 -7 hannah update)

page 3085
Results section pages 243-244
page 78
pages 6-8

p.5-7 +9

pages 616-618

p.7-8
page 3-7

pages 2 - 4 and table 3

Supplementary figure 1.
Results and Discussion sections pages 8-12

page 13

p. 114-115
Page 1
Results and Discussion sections pages 7-12

Results section pages 3-5
pages 3-6 and supplementary data

Results section pages 2-5, Table 3, Discussin section page [illegible]

p.8-9
Findings; page 442

p.6-12

Results page 2-4

Figure 2, p. 5-8

Table 3, Table 4

Results section pages 8-9, Discussion section pages 10-11

pages 4 -6

Results section pages 1295-1296, Discussion section pages
pages 5-6

p.3-9
pages 4-9

p.2-8

pages 213-215

Results section pages 706-707

p. 923, 925
pages 7-10 and Figure 2

p.3-8

pages 4-8

pages 707 -709

results section
pages 9-10

p. 3-5

p.4-10

p.2-8

pages 6-8
pages 4 -8

Table 3, p. 4-7

Results section page 83, Tables 4 & 5
pages 5-7 and online supplemental file 4

page 3
page 362
results section

results section

Results section pages 4-9

p.4-13

p.263, Figure 2

p.3-5

	Author
Were any desired features or future wishes reported?	
	Adler
	Adler
	Adler
	Adler
	Adler
	Adler
	Adler
	Adler
	Adler
	Adler
	Adler
	Adler
	Adler
	Adler
	Adler
	Adler
	Adler
	Adler
	Al Hemyari
	Al Hemyari
	Al Hemyari
	Al Hemyari
	Al Hemyari
	Al Hemyari
	Al Hemyari
	Al Hemyari
	Al Hemyari
	Al Hemyari
	Al Hemyari
	Alaboud
	Alaboud
	Alaboud
	Alaboud
	Alaboud
	Alaboud

	Alaboud
	Alaboud
	Alaboud
	Alaboud
	Alaboud
	Alaboud
	Alaboud
	Alaboud
	Alaboud
	Alaboud
	Alaboud
	Alaboud
	Alarabyat
	Alarabyat
	Alarabyat
	Alarabyat
	Alarabyat
	Alarabyat
	Alarabyat
	Alarabyat
	Alarabyat
	Alarabyat
	Alarabyat
	Alarabyat
	Alarabyat
	Alarabyat
	Alarabyat
	Alarabyat
	Alarabyat
	Alarabyat
	Alarabyat
	Alarabyat
	Alarabyat
	Alarabyat
	Alarabyat
	Alarabyat
	Alarabyat

	Alarabyat
	Alarabyat
	Alarabyat
	Alarabyat
	Alesssa
	Alesssa
	Alesssa
	Alesssa
	Alesssa
	Alesssa
	Alesssa
	Alesssa
	Alesssa
	Alesssa
	Alesssa
	Alesssa
	Alesssa
	Alesssa
	Alesssa
	Alesssa
	Alesssa
	Alesssa
	Alesssa
	Alesssa
	Alesssa
	Alesssa
	Alesssa
	Alesssa
	Alesssa
	Alesssa
	Alesssa
	Alesssa
	Alesssa
	Alesssa
	Alesssa
	Alesssa
	Alesssa

	Alesssa
	Alesssa
	Alesssa
	Alesssa
	Andersson
	Andersson
	Andersson
	Andersson
	Andersson
	Andersson
	Andersson
	Andersson
	Andersson
	Andersson
	Andersson
	Andersson
	Andersson
	Andersson
	Andersson
	Andersson
	Andersson
	Andersson
	Andersson
	Andersson
[illegible]	Andersson
	Aquino
	Aquino
	Aquino
	Aquino
	Aquino
	Aquino
	Aquino
	Aquino
	Aquino
	Aquino
	Aquino
	Aquino

	Aquino
	Aquino
	Aquino
	Aquino
	Aquino
	Aquino
	Aquino
	Aquino
	Aquino
	Aquino
	Aquino
	Aquino
	Aquino
	Aquino
	Aquino
	Aquino
	Aquino
	Artanian
	Artanian
	Artanian
	Artanian
	Artanian
	Artanian
	Artanian
	Artanian
	Artanian
	Artanian
	Artanian
	Artanian
	Artanian
	Artanian
	Artanian
	Artanian
	Artanian
	Auton
	Auton
	Auton

	Auton
	Auton
	Auton
	Auton
	Auton
	Auton
	Auton
	Auton
	Auton
	Auton
	Auton
	Auton
	Auton
	Aw
	Aw
	Aw
	Aw
	Aw
	Aw
	Aw
[illegible] some tools to visualize data in general and it is incorporated in those same	Baratta
	Baratta
	Baratta
	Baratta
	Baratta
	Baratta
	Baratta
	Baratta
	Baratta
	Aquino
	Baratta
	Baratta
	Baratta
	Baratta
	Baratta
	Baratta
	Baratta

	Baratta
	Baratta
	Baratta
	Baratta
	Baratta
	Baratta
	Baratta
	Baratta
	Baratta
	Baratta
	Baratta
	Baratta
	Baratta
	Baratta
	Baratta
	Baratta
	Baratta
	Baratta
	Baratta
	Baratta
	Bazzano
	Bazzano
	Bazzano
	Bazzano
	Bazzano
	Bazzano
	Bazzano
	Bazzano
	Bazzano
	Bazzano
	Bazzano
	Bazzano
	Bazzano
	Bazzano
	Bazzano
	Bazzano
	Bazzano

	Bazzano
	Bazzano
	Bazzano
	Bazzano
	Bazzano
	Bazzano
	Bergstedt
	Bergstedt
	Bergstedt
	Bergstedt
	Bergstedt
	Bergstedt
	Bergstedt
	Bergstedt
	Bergstedt
	Bergstedt
	Bergstedt
	Bergstedt
	Bergstedt
	Bergstedt
	Bergstedt
	Bergstedt
	Bergstedt
	Bergstedt
	Bergstedt
	Bernadi
	Bernadi
	Bernadi
	Bernadi
	Bernardi
	Bernardi
	Bernardi
	Bernardi
	Bernardi
	Bernardi
	Bernardi
	Bernardi

	Bernardi
	Bernardi
	Bernardi
	Bernardi
	Bernardi
	Bernardi
	Bhandari
	Bhandari
	Bhandari
	Bhandari
	Bhandari
	Bhandari
	Bhandari
	Bhandari
	Bhandari
	Bhandari
	Bhandari
	Bhandari
	Bhandari
	Bhandari
	Bhandari
	Bhandari
	Bhandari
	Bhandari
	Bhandari
	Boriani
	Boriani
	Boriani
	Boriani
	Boriani
	Boriani
	Boriani
	Boriani
	Boriani
	Boriani
	Bouchard
	Bouchard

	Bouchard
	Bouchard
	Bouchard
	Bouchard
	Bouchard
	Bouchard
	Bouchard
	Bouchard
	Bouchard
	Bouchard
	Bouchard
	Bouchard
	Bouchard
	Bouchard
	Bouchard
	Bouchard
	Bouchard
	Bouchard
	Bouchard
	Bouchard
	Bouchard
	Bouchard
	Bouchard
	Bouchard
	Bouchard
	Bouchard
	Bouchard
	Bouchard
	Bouchard
	Bouchard
	Bouchard
	Breil
	Breil
	Breil
	Breil
	Breil
	Breil

	Breil
	Breil
	Brouns
	Brouns
	Brouns
	Brouns
	Brouns
	Brouns
	Brouns
	Brouns
	Brouns
	Brouns
	Brouns
	Brouns
	Brouns
	Brouns
	Brouns
	Brouns
	Brouns
	Brouns
	Brouns
	Brouns
	Brouns
	Brouns
	Brouns
	Brouns
	Brouns
	Brouns
	Brouns
	Brouns
	Brouns
	Brown
	Brown
	Brown
	Brown
	Brown
	Brown
	Brown

	Brown
	Brown
	Brown-Johnson
	Brown-Johnson
	Brown-Johnson
	Brown-Johnson
	Brown-Johnson
	Brown-Johnson
	Brown-Johnson
	Brown-Johnson
	Brown-Johnson
	Brown-Johnson
	Brown-Johnson
	Brown-Johnson
	Brown-Johnson
	Brown-Johnson
	Brown-Johnson
	Brown-Johnson
	Brown-Johnson
	Brown-Johnson
	Brown-Johnson
	Brown-Johnson
	Brown-Johnson
	Brown-Johnson
	Brown-Johnson
	Brown-Johnson
	Buis
	Buis
	Buis
	Buis
	Buis
	Buis
	Buis
	Buis
	Buis
	Buis
	Buis
	Buis

	Buis
	Buis
	Buis
	Buis
	Buis
	Buis
	Buis
	Buis
	Buis
	Buis
	Buis
	Buis
	Buis
	Buis
	Buis
	Buis
	Buis
	Buis
	Buis
	Buis
	Buis
	Buis
	Buis
	Buis
	Buis
	Buis
	Buis
	Buis
	Buis
	Buis
	Buis
	Buis
	Buis
	Buis
	Burunkaya
	Burunkaya
	Burunkaya

	Burunkaya
	Burunkaya
	Burunkaya
	Burunkaya
	Burunkaya
	Burunkaya
	Burunkaya
	Burunkaya
	Burunkaya
	Burunkaya
	Burunkaya
	Burunkaya
	Burunkaya
	Burunkaya
	Burunkaya
	Burunkaya
	Burunkaya
	Burunkaya
	Burunkaya
	Burunkaya
	Burunkaya
	Burunkaya
	Burunkaya
	Burunkaya
	Cao
	Cao
	Cao
	Cao
	Cao
	Cao
	Cao
	Cao
	Cao
	Cao
	Cao
	Cao
	Cao

	Cao
	Cao
	Cao
	Cao
	Cartledge
	Cartledge
	Cartledge
	Cartledge
	Cartledge
	Cartledge
	Cartledge
	Cartledge
	Cartledge
	Cartledge
	Cartledge
	Cartledge
	Cartledge
	Cartledge
	Cartledge
	Cartledge
	Cartledge
	Cartledge
	Cartledge
	Cartledge
	Cartledge
	Cartledge
	Cartledge
	Cartledge
	Cartledge
	Cartledge
	Cartledge
	Cartledge
	Cartledge
	Cartledge
	Cartledge
	Cartledge
	Cartledge

	Cher
	Cher
	Cher
	Cher
	Cher
	Cher
	Cher
	Cher
	Cher
	Cher
	Cher
	Cher
	Cher
	Chew
	Chew
	Chew
	Chew
	Chew
	Chew
	Chew
	Chew
	Chew
	Chew
	Chew
	Chew
	Chew
	Chew
	Chew
	Chew
	Chew
	Chew
	Chew
	Chew
	Chew
	Chew
	Chew

	Chew
	Chew
	Chew
	Chew
	Chew
	Chew
	Chew
	Chew
	Chew
	Chew
	Chew
	Chew
	Chew
	Chew
	Chew
	Chew
	Chew
	Chew
	Chew
	Chew
	Chew
	Chew
	Chew
	Chew
	Chew
	Chew
	Chew
	Chew
	Chew
	Chew
	Chew
	Chew
	Cornell
	Cornell
	Cornell
	Cornell
	Cornell

	Cornell
	Cornell
	Cornell
	Cornell
	Cornell
	Cornell
	Cornell
	Cornell
	Cornell
	Cornell
	Cornell
	Cornell
	Cornell
	Cornell
	Cornell
	Cornell
	Cornell
	Cornell
	Cornell
	Cornell
	Cornell
	Coylewright
	Coylewright
	Coylewright
	Coylewright
	Coylewright
	Coylewright
	Coylewright
	Coylewright
	Coylewright
	Danielsbacka
	Danielsbacka
	Danielsbacka
	Danielsbacka
	Danielsbacka
	Danielsbacka
	Danielsbacka

	Danielsbacka
	Danielsbacka
	Danielsbacka
	Danielsbacka
	Danielsbacka
	Danielsbacka
	Danielsbacka
	Danielsbacka
	Danielsbacka
	Danielsbacka
	Danielsbacka
	Danielsbacka
	Danielsbacka
	Danielsbacka
	Danielsbacka
	Danielsbacka
	Danielsbacka
	Danielsbacka
	Danielsbacka
	Danielsbacka
	Danielsbacka
	Danielsbacka
	Danielsbacka
	Danielsbacka
	Danielsbacka
	Danielsbacka
	Danielsbacka
	Danielsbacka
	Danielsbacka
	Danielsbacka
	Danielsbacka
	Danielsbacka
	Danielsbacka
	Danielsbacka
	Danielsbacka
	Danielsbacka
	Danielsbacka

	Danielsbacka
	Danielsbacka
	Danielsbacka
	Danielsbacka
	Danielsbacka
	Danielsbacka
	Danielsbacka
	Danielsbacka
	Danielsbacka
	Danielsbacka
	Danielsbacka
	Danielsbacka
	Danielsbacka
	Danielsbacka
	Danielsbacka
	Danielsbacka
	Danielsbacka
	Danielsbacka
	Danielsbacka
	Danielsbacka
	Danielsbacka
	Danielsbacka
	Danielsbacka
	Danielsbacka
	Danielsbacka
	Danielsbacka
	Danielsbacka
	Danielsbacka
	Danielsbacka
	Danielsbacka
	Danielsbacka
	Danielsbacka
	Danielsbacka
	Danielsbacka
	Danielsbacka
	Danielsbacka
	Danielsbacka

	Danielsbacka
	Danielsbacka
	Danielsbacka
	Danielsbacka
	Danielsbacka
	Danielsbacka
	Danielsbacka
	Danielsbacka
	Danielsbacka
	Danielsbacka
	Danielsbacka
	Danielsbacka
	Danielsbacka
	Danielsbacka
	Danielsbacka
	Danielsbacka
	Danielsbacka
	Danielsbacka
	Danielsbacka
	Demers
	Demers
	Demers
	Demers
	Demers
	Demers
	Demers
	Demers
	Demers
	Demers
	Demers
	Demers
	Demers
	Demers
	Demers
	Ding
	Ding
	Ding

	Ding
	Ding
	Ding
	Ding
	Ding
	Ding
	Ding
	Ding
	Ding
	Ding
	Ding
	Ding
	Ding
	Ding
	Ding
	Ding
	Ding
	Ding
	Ding
	Ding
	Ding
	Ding
	Dullabh
	Dullabh
	Dullabh
	Dullabh
	Dullabh
	Dullabh
	Dullabh
	Dullabh
	Dullabh
	Dullabh
	Dullabh
	Dullabh
	Dullabh
	Dullabh
	Dullabh

	Dullabh
	Dullabh
	Dullabh
	Einhorn
	Einhorn
	Einhorn
	Einhorn
	Einhorn
	Einhorn
	Einhorn
	Einhorn
	Einhorn
	Einhorn
	Einhorn
	Einhorn
	Einhorn
	Einhorn
	Einhorn
	Einhorn
	Einhorn
	Einhorn
	Einhorn
	Einhorn
	Einhorn
	Einhorn
	Einhorn
	Einhorn
	Einhorn
	Einhorn
	Einhorn
	Einhorn
	Einhorn
	Einhorn
	Einhorn
	Einhorn
	Einhorn

	Einhorn
	Einhorn
	Einhorn
	Einhorn
	Einhorn
	Einhorn
	Einhorn
	Einhorn
	Einhorn
	Einhorn
	Einhorn
	Einhorn
	Einhorn
	Einhorn
	Einhorn
	Einhorn
	Einhorn
	Einhorn
	Einhorn
	Einhorn
	Einhorn
	Einhorn
	Einhorn
	Einhorn
	Einhorn
	Einhorn
	Einhorn
	Einhorn
	Einhorn
	Einhorn
	Einhorn
	Einhorn
	Einhorn
	Einhorn
	Einhorn
	Einhorn
	Einhorn

	Einhorn
	Einhorn
	Einhorn
	Einhorn
	Einhorn
	Einhorn
	Einhorn
	Einhorn
	Einhorn
	Einhorn
	Einhorn
	Einhorn
	Ferguson
	Ferguson
	Ferguson
	Ferguson
	Ferguson
	Ferguson
	Ferguson
	Ferguson
	Ferguson
	Ferguson
	Ferguson
	Ferguson
	Ferguson
	Ferguson
	Ferguson
	Ferguson
	Ferguson
	Ferguson
	Ferguson
	Ferguson
	Ferguson
	Ferguson
	Flores-Sandoval
	Flores-Sandoval
	Flores-Sandoval

	Flores-Sandoval
	Flores-Sandoval
	Flores-Sandoval
	Flores-Sandoval
	Flores-Sandoval
	Flores-Sandoval
	Flores-Sandoval
	Flores-Sandoval
	Flores-Sandoval
	Flores-Sandoval
	Flores-Sandoval
	Frith
	Frith
	Frith
	Frith
	Frith
	Frith
	Frith
	Frith
	Gago
	Gago
	Gago
	Gago
	Gago
	Gago
	Gago
	Gago
	Gago
	Gago
	Gago
	Gago
	Gago
	Gago
	Gago
	Gago
	Gago
	Gago

	Gago
	Gago
	Gago
	Gago
[illegible] after exacerbation, when they might be more willing to improve their self-	Giordan
	Giordan
	Giordan
	Giordan
	Giordan
	Giordan
	Giordan
	Giordan
	Giordan
	Giordan
	Giordan
	Giordan
	Giordan
	Giordan
	Giordan
	Giordan
	Giordan
	Giordan
	Giordan
	Giordan
	Giordan
	Giordan
	Giordan
	Giordan
	Giordan
	Giordan
	Giordan
	Giordan
	Giordan
	Giordan
	Giordan
	Giordan
	Giordan

	Giordan
	Giordan
	Giordan
	Giordan
	Giordan
	Giordan
	Giordan
	Giordan
	Giordan
	Giordan
	Giordan
	Giordan
	Giordan
	Giordan
	Giordan
	Giordan
	Gomis-Pastor
	Gomis-Pastor
	Gomis-Pastor
	Gomis-Pastor
	Gomis-Pastor
	Gomis-Pastor
	Gomis-Pastor
	Gomis-Pastor
	Gomis-Pastor
	Gomis-Pastor
	Gomis-Pastor
	Gomis-Pastor
	Gomis-Pastor
	Gomis-Pastor
	Gomis-Pastor
	Gomis-Pastor
	Gomis-Pastor
	Gomis-Pastor
	Gomis-Pastor
	Gomis-Pastor
	Gomis-Pastor

	Gomis-Pastor
	Gomis-Pastor
	Gomis-Pastor
	Gomis-Pastor
	Gomis-Pastor
	Gomis-Pastor
	Gondi
	Gondi
	Gondi
	Gondi
	Gondi
	Gondi
	Gondi
	Gondi
	Gondi
	Gondi
	Gondi
	Gondi
	Gondi
	Gondi
	Gondi
	Gondi
	Gondi
	Gondi
	Gondi
	Gondi
	Gondi
	Gondi
	Gondi
	Gondi
	Gondi
	Gondi
	Gondi
	Gondi
	Gondi
	Gondi
	Gondi

	Gondi
	Gondi
	Gondi
	Gondi
	Gondi
	Gondi
	Gondi
	Gondi
	Gondi
	Gondi
	Gondi
	Gondi
	Gondi
Opportunities to monitor mental health symptoms and/or conditions were similarly identified across participants ("We have a lot of people with anxiety	Gordon
	Gordon
	Gordon
	Gordon
	Gordon
	Gordon
	Gordon
	Gordon
	Gordon
	Gordon
	Gordon
	Gordon
	Gordon
	Gordon
	Gordon
	Gordon
	Gordon
	Gordon
	Gordon
	Gordon
	Gordon
	Gordon
	Gordon

	Gordon
	Gordon
	Gordon
	Gordon
	Gordon
	Gordon
	Gordon
	Gordon
	Gordon
	Gordon
	Gordon
	Gordon
	Gordon
	Gordon
	Gordon
	Gordon
	Gordon
	Gordon
	Gordon
	Gordon
	Gordon
	Gordon
	Gordon
	Gordon
	Gordon
	Gordon
	Gordon
	Gordon
	Gordon
	Gordon
	Gordon
	Gordon
	Gordon
	Gordon
	Gordon
	Gordon
	Gordon

	Gordon
	Gregory
	Gregory
	Gregory
	Gregory
	Gregory
	Gregory
	Gregory
	Gregory
	Gregory
	Gregory
	Gregory
	Gregory
	Gregory
	Gregory
	Gregory
	Gregory
	Gregory
	Gregory
	Gregory
	Gregory
	Gregory
	Gregory
	Gregory
	Gregory
	Gregory
	Gregory
	Gregory
	Gregory
	Gregory
	Gregory
	Gregory
	Grey
	Grey
	Grey
	Grey
	Grey

	Grey
	Grey
	Grey
	Grey
	Grey
	Grey
	Grey
	Grey
	Grey
	Grey
	Grey
	Grey
	Grey
	Grey
	Grey
	Grey
	Grey
	Grey
	Grey
	Grey
	Grey
	Grey
	Grey
	Grey
	Grey
	Grey
	Grey
	Grey
	Grey
	Grey
	Grey
	Grey
	Grey
	Grey
	Grey
	Grey
	Grey

	Grey
	Grey
	Grey
	Grey
	Gupta
	Gupta
	Gupta
	Gupta
	Gupta
	Gupta
	Gupta
	Gupta
	Gupta
	Gupta
	Gupta
	Gupta
	Gupta
	Gupta
	Gupta
	Gupta
	Gupta
	Gupta
	Gupta
	Gupta
	Gupta
	Gupta
	Gupta
	Gupta
	Gupta
	Gupta
Potential implementation strategies suggested by the participants: Improved trai	Hammersley
	Hammersley
	Hammersley
	Hammersley
	Hammersley
	Hammersley

	Hammersley
	Hammersley
	Hammersley
	Hammersley
	Hammersley
	Hammersley
	Hammersley
	Hammersley
	Hammersley
	Hammersley
	Hammersley
	Hammersley
	Hammersley
	Hammersley
	Hammersley
	Hammersley
	Hammersley
	Hammersley
	Hammersley
	Hammersley
	Hammersley
	Hammersley
	Hammersley
	Hammersley
	Hammersley
	Hammersley
	Hammersley
	Hammersley
	Hammersley
	Hammersley
	Hammersley
	Hammersley
	Hammersley
	Hammersley
	Hammersley
	Hammersley
	Hammersley

	Hammersley
	Hammersley
	Hammersley
	Hammersley
	Hammersley
	Hammersley
	Hammersley
	Hammersley
	Hammersley
	Hammersley
	Hammersley
	Hammersley
	Hammersley
	Hammersley
	Hammersley
	Hammersley
	Hammersley
	Hammersley
	Hammersley
	Hammersley
	Harris
	Harris
	Harris
	Harris
	Harris
	Harris
	Harris
	Harris
	Harris
	Harris
	Harris
	Harris
	Harris
	Harris
	Harris
	Harris
	Harris

	Harris
	Harris
	Harris
	Harris
	Harris
	Harris
	Harris
	Harris
	Harris
	Harris
	Harris
	Harris
	Harris
	Harris
	Harris
	Harris
	Harris
	Harris
	Harris
	Harris
	Harris
	Harris
	Harvey
	Harvey
	Harvey
	Harvey
	Harvey
	Harvey
	Harvey
	Harvey
	Harvey
	Harvey
	Harvey
	Harvey
	Harvey
	Harvey
	Harvey

	Harvey
	Harvey
	Harvey
	Harvey
	Harvey
	Harvey
	Harvey
	Harvey
	Harvey
	Harvey
	Harvey
	Harvey
	Harvey
	Harvey
	Harvey
	Harvey
	Harvey
	Harvey
	Harvey
	Harvey
	Harvey
	Harvey
	Harvey
	Harvey
	Harvey
	Harvey
	Harvey
	Harvey
	Harvey
	Harvey
	Harvey
	Harvey
	Harvey
	Harvey
	Harvey
	Harvey
	Harvey
	Hassan

	Hassan
	Hassan
	Hassan
	Hassan
	Hassan
	Hassan
	Hassan
	Hassan
	Hassan
	Hassan
	Hassan
	Hassan
	Hassan
	Hassan
	Hassan
	Hassan
	Hassan
	Hassan
	Hassan
	Hassan
	Hassan
	Hassan
	Hassan
	Hassan
	Hassan
	Hassan
	Hassan
	Hassan
	Hassan
	Hassan
	Hassan
	Hassan
	Hassan
	Hassan
	Hassan
	Hassan
	Hassan

	Hassan
	Hassan
	Hassan
	Hassan
	Hassan
	Hassan
	Hassan
	Hassan
	Hassan
	Hassan
	Hassan
	Hassan
	Hassan
	Hassan
	Hassan
	Hauschildt
	Hauschildt
	Hauschildt
	Hauschildt
	Hauschildt
	Hauschildt
	Hauschildt
	Hauschildt
	Hauschildt
	Hauschildt
	Hauschildt
	Hauschildt
	Hauschildt
	Hauschildt
	Hauschildt
	Hauschildt
	Hauschildt
	Hauschildt
	Hauschildt
	Hauschildt
	Hauschildt
	Hauschildt

	Hauschildt
	Hauschildt
	Hauschildt
	Hauschildt
	Hauschildt
	Hauschildt
	Hauschildt
	Hauschildt
	Hauschildt
	Hauschildt
	Hauschildt
	Hauschildt
	Hauschildt
	Hauschildt
	Hauschildt
	Hauschildt
	Hauschildt
	Hellem
	Hellem
	Hellem
	Hellem
	Hellem
	Hellem
	Hellem
	Hellem
	Hellem
	Hellem
	Hellem
	Hellem
	Hellem
	Hellem
	Hellem
	Hellem
	Hellem
	Hellem
	Hellem
	Hellem

	Hellem
	Hellem
	Hellem
	Hellem
	Hellem
	Hellem
	Hellem
	Hellem
	Hellem
	Hellem
	Hellem
	Hellem
	Hellem
	Hellem
	Hellem
	Hellem
	Honein-AbouHaidar
	Honein-AbouHaidar
	Honein-AbouHaidar
	Honein-AbouHaidar
	Honein-AbouHaidar
	Honein-AbouHaidar
	Honein-AbouHaidar
	Honein-AbouHaidar
	Honein-AbouHaidar
	Honein-AbouHaidar
	Honein-AbouHaidar
	Honein-AbouHaidar
	Houle
	Houle
	Houle
	Houle
	Houle
	Houle
	Houle
	Houle
	Houle

	Houle
	Houle
	Houle
	Houle
	Houle
	Houle
	Houle
	Houle
	Houle
	Houle
	Houle
	Houle
[illegible] GPs stated they would be interested in viewing a patient’s data. Three options	Indraratna
	Indraratna
	Indraratna
	Indraratna
	Indraratna
	Indraratna
	Indraratna
	Indraratna
	Indraratna
	Indraratna
	Indraratna
	Indraratna
	Indraratna
	Indraratna
	Indraratna
	Indraratna
	Indraratna
	Indraratna
	Indraratna
	Indraratna
	Indraratna
	Indraratna
	Indraratna
	Indraratna
	Indraratna
	Indraratna

	Indraratna
	Indraratna
	Indraratna
	Indraratna
	Indraratna
	Indraratna
	Indraratna
	Indraratna
	Indraratna
	Indraratna
	Indraratna
	Indraratna
	Indraratna
Suggestions included medication reminders, monitoring and adjusting medication remotely by the healthcare team, and notifications to the pharmacy	Islam
	Islam
	Islam
	Islam
	Islam
	Islam
	Islam
	Islam
	Islam
	Islam
	Islam
	Islam
	Islam
	Islam
	Islam
	Islam
	Islam
	Islam
	Islam
	Islam
	Islam
	Islam
	Islam

	Islam
	Islam
	Islam
	Islam
	Islam
	Islam
	Islam
	Islam
	Islam
	Islam
	Islam
	Islam
	Islam
	Islam
	Islam
	Islam
	Islam
	Islam
	Islam
	Islam
	Islam
	Islam
	Islam
	Islam
	Islam
	Islam
	Islam
	Islam
	Islam
	Islam
	Islam
	Islam
	Jongsma
	Jongsma
	Jongsma
	Jongsma
	Jongsma

	Jongsma
	Jongsma
	Jongsma
	Jongsma
	Jongsma
	Jongsma
	Jongsma
	Jongsma
	Jongsma
	Jongsma
	Jongsma
	Jongsma
	Jongsma
	Jongsma
	Jongsma
	Jongsma
	Jongsma
	Jongsma
	Jongsma
	Jongsma
	Jongsma
	Jongsma
	Jongsma
	Kalinina 2020
	Kalinina 2020
	Kalinina 2020
	Kalinina 2020
	Kalinina 2020
	Kalinina 2020
	Kalinina 2020
	Kalinina 2020
	Kalinina 2020
	Kalinina 2020
	Kalinina 2020
	Kalinina 2020
	Kalinina 2020
	Kalinina 2020

	Kalinina 2020
	Kalinina 2020
	Kalinina 2020
	Kalinina 2020
	Kalinina 2020
	Kalinina 2021
	Kalinina 2021
	Kalinina 2021
	Kalinina 2021
	Kalinina 2021
	Kalinina 2021
	Kalinina 2021
	Kalinina 2021
	Kalinina 2021
	Kalinina 2021
	Kalinina 2021
	Kalinina 2021
	Kalinina 2021
	Kalinina 2021
	Kalinina 2021
	Kalinina 2021
	Kalinina 2021
	Kalinina 2021
	Kalinina 2021
	Kalinina 2021
	Kalinina 2021
	Kalinina 2021
	Kalinina 2021
	Kalinina 2021
	Kalinina 2021
	Kalinina 2021
	Kalinina 2021
	Kauw
	Kauw
	Kauw
	Kauw
	Kauw

	Kauw
	Kauw
	Kauw
	Kauw
	Kauw
	Kauw
	Kauw
	Kauw
	Kauw
	Kauw
	Kauw
	Kauw
	Kauw
	Kauw
	Kauw
	Kauw
	Kauw
	Kauw
	Kauw
	Kauw
	Kauw
	Kauw
	Kauw
	Kauw
	Kauw
	Kauw
	Kauw
	Kauw
	Kauw
	Kauw
	Kauw
	Kauw
	Kauw
	Kauw
	Kauw
	Kauw
	Kauw

	Kerr
	Kerr
	Kerr
	Kerr
	Khonsari
	Khonsari
	Khonsari
	Khonsari
	Khonsari
	Khonsari
	Khonsari
	Khonsari
	Khonsari
	Khonsari
	Khonsari
	Khonsari
	Khonsari
	Khonsari
	Klaiman
	Klaiman
	Klaiman
	Klaiman
	Klaiman
	Klaiman
	Klaiman
	Klaiman
	Klaiman
	Klaiman
	Klaiman
	Klaiman
	Klaiman
	Klaiman
	Klaiman
	Klaiman
	Klaiman
	Klaiman
	Klaiman

	Klaiman
	Klaiman
	Klaiman
	Klaiman
	Klaiman
	Kleiner
	Kleiner
	Kleiner
	Kleiner
	Kleiner
	Kleiner
	Kleiner
	Kobe
	Kobe
	Kocanda
	Kocanda
	Kocanda
	Kocanda
	Kocanda
	Kocanda
	Kocanda
	Kocanda
	Kocanda
	Kocanda
	Kocanda
	Kocanda
	Kocanda
	Kocanda
	Kocanda
	Kocanda
	Kocanda
	Kołtowski
	Kołtowski
	Kołtowski
	Kołtowski
	Ladegaard
	Ladegaard

	Ladegaard
	Ladegaard
	Ladegaard
	Ladegaard
	Ladegaard
	Ladegaard
	Ladegaard
	Ladegaard
	Ladegaard
	Ladegaard
	Ladegaard
	Ladegaard
	Ladegaard
	Ladegaard
	Ladegaard
	Ladegaard
	Ladegaard
	Ladegaard
	Ladegaard
	Ladegaard
	Ladegaard
	Ladegaard
	Ladegaard
	Leonard
	Leonard
	Leonard
	Leonard
	Leonard
	Leonard
	Leonard
	Leonard
	Leonard
	Leonard
	Leonard
	Leonard
	Leonard
	Leonard

	Leonard
	Leonard
	Leonard
	Leonard
	Leonard
	Leonard
	Leonard
	Leonard
	Leonard
	Leonard
	Leonard
	Leonard
	Leonard
	Leonard
	Leonard
	Leonard
	Leonard
	Leonard
	Leonard
	Leonard
	Leonard
	Leonard
	Leonard
	Leonard
	Leonard
	Leonard
	Leonard
	Leonard
	Leonard
	Lino
	Lino
	Lino
	Lukey
	Lukey
	Lukey
	Lukey
	Lukey

	Lukey
	Lukey
	Lukey
	Lukey
	Lukey
	Lukey
	Lukey
	Lukey
	Lukey
	Lukey
	Lukey
	Lukey
	Lukey
	Lukey
	Lukey
	Lukey
	Lyles
	Lyles
	Lyles
	Lyles
	Lyles
	Lyles
	Lyles
	Lyles
	Lyles
	Lyles
	Lyles
	Mahmoud
	Mahmoud
	Mahmoud
	Mahmoud
	Mahmoud
	Mahmoud
	Mahmoud
	Mahmoud
	Mahmoud
	Mahmoud

	Mahmoud
	Mahmoud
	Mahmoud
	Mahmoud
	Maria
	Maria
	Maria
	Maria
	Maria
	Maria
	Maria
	Maria
	Maria
	Maria
	Maria
	Maria
	Maria
	Maria
	Maria
	Maria
	Maria
	Maria
	Maria
	Maria
	Maria
	Maria
	Maria
	Maria
	Maria
	Maria
	Maria
	Maria
	Maria
	Maria
	Maria
	Maria
	Maria

	Maria
	Maria
	Maria
	Maria
	Marzolini
	Marzolini
	Marzolini
	Marzolini
	Marzolini
	Marzolini
	Marzolini
	Marzolini
	Marzolini
	Marzolini
	Marzolini
	Marzolini
	Marzolini
	Marzolini
	Marzolini
	Marzolini
	Marzolini
	Marzolini
	Marzolini
	Marzolini
	Marzolini
	Marzolini
	Marzolini
	Marzolini
	Marzolini
	Marzolini
	Marzolini
	Marzolini
	Matthiesen
	Matthiesen
	Matthiesen
	Matthiesen
	Matthiesen

	Matthiesen
	Matthiesen
	Matthiesen
	Matthiesen
	Matthiesen
	Matthiesen
	Matthiesen
	Matthiesen
	Matthiesen
	Matthiesen
	Matthiesen
	Matthiesen
	Matthiesen
	Matthiesen
	Matthiesen
	Matthiesen
	Matthiesen
	Matthiesen
	Matthiesen
	Matthiesen
	Matthiesen
	Matthiesen
	Matthiesen
	Matthiesen
	Matthiesen
	Matthiesen
	Matthiesen
	Matthiesen
	Matthiesen
	Matthiesen
	Matthiesen
	Matthiesen
	Matthiesen
	McCarthy
	McCarthy
	McCarthy
	McCarthy

	McCarthy
	McCarthy
	McCarthy
	McCarthy
	McCarthy
	McCarthy
	McCarthy
	McCarthy
	McCarthy
	McCarthy
	McGillion
	McGillion
	McGillion
	McGillion
	McGillion
	McGillion
	McGillion
	McGillion
	McGillion
	McGillion
	McGillion
	McGillion
	McGillion
	McGillion
	McGillion
	McGillion
	McGillion
	McGillion
	McGillion
	McGillion
	McGillion
	McGillion
	McGillion
	McGillion
	McGillion
	McGillion
	McGillion

	McGillion
	McGillion
	McGillion
	McGillion
	Meador
	Meador
	Meador
	Meador
	Meador
	Meador
	Meador
	Meador
	Meador
	Meador
	Meador
	Meador
	Meador
	Meador
	Meador
	Meador
	Meador
	Meador
	Meador
	Mehta
	Mehta
	Mehta
	Morton
	Morton
	Morton
	Morton
	Morton
	Morton
	Morton
	Morton
	Morton
	Morton
	Morton

	Morton
	Morton
	Muehlensiepen
	Muehlensiepen
	Muehlensiepen
	Muehlensiepen
	Muehlensiepen
	Muehlensiepen
	Muehlensiepen
	Nau
	Nau
	Nau
	Nau
	Nau
	Nau
	Nau
Requirements for long-term sustainability of virtual care for ACSCs: (1) maintena	Nayyar
	Nayyar
	Nayyar
	Nayyar
	Nayyar
	Nayyar
	Nayyar
	Nayyar
	Nayyar
	Nayyar
	Nayyar
	Nayyar
	Nayyar
	Nayyar
	Nayyar
	Nayyar
	Nayyar
	Nayyar
	Nayyar
	Nayyar
	Nayyar

	Nayyar
	Nayyar
	Nayyar
	Nayyar
	Nayyar
	Nayyar
	Nayyar
	Nayyar
	Nayyar
	Nayyar
	Nayyar
	Nayyar
	Nayyar
	Nayyar
	Nayyar
	Nayyar
	Nayyar
	Nayyar
	Nayyar
	Nayyar
	Nayyar
	Nayyar
	Nayyar
	Nayyar
	Nayyar
	Nayyar
	Nayyar
	Nayyar
	Nayyar
	Nayyar
	Nayyar
	Nayyar
	Nayyar
	Nayyar
	Nayyar
	Nayyar
	Nayyar

	Nayyar
	Nayyar
	Nayyar
	Nayyar
	Nayyar
	Nayyar
	Nayyar
	Nayyar
	Nayyar
	Nayyar
	Nayyar
	Nayyar
	Nayyar
	Nayyar
	Nayyar
	Nayyar
	Nayyar
	Nayyar
	Nayyar
	Nayyar
	Nayyar
	Nayyar
	Nayyar
	Nayyar
	Nayyar
	Nemis-White
	Nemis-White
	Nemis-White
	Nemis-White
	Nemis-White
	Nemis-White
	Nemis-White
	Nemis-White
	Nemis-White
	Nemis-White
	Nemis-White
	Nemis-White

	Nemis-White
	Nemis-White
	Nemis-White
	Nemis-White
	Nemis-White
	Nemis-White
	Nemis-White
	Nemis-White
	Nemis-White
	Nemis-White
	Nemis-White
	Nemis-White
	Otieno
	Otieno
	Otieno
	Otieno
	Otieno
	Otieno
	Otieno
	Palozzi
	Palozzi
	Palozzi
	Palozzi
	Palozzi
	Palozzi
	Palozzi
	Palozzi
	Palozzi
	Palozzi
	Palozzi
	Palozzi
	Palozzi
	Palozzi
	Palozzi
	Palozzi
	Palozzi

	Palozzi
	Palozzi
	Palozzi
	Palozzi
	Palozzi
	Palozzi
	Palozzi
	Palozzi
	Palozzi
	Palozzi
	Palozzi
	Palozzi
	Palozzi
	Palozzi
	Palozzi
	Palozzi
	Pelly
	Pelly
	Pelly
	Pelly
	Pelly
	Pelly
	Pelly
	Pelly
	Pelly
	Pelly
	Pelly
	Pelly
	Pelly
	Pelly
	Pelly
	Pelly
	Pelly
	Pelly
	Pelly
	Pelly
	Pelly

	Pelly
	Pelly
	Pelly
	Pelly
	Pelly
	Pelly
	Pelly
	Pelly
	Pelly
	Pelly
	Pelly
	Pelly
	Pelly
	Pelly
	Pelly
	Pelly
	Pelly
	Pelly
	Pelly
	Pelly
	Pelly
	Pelly
	Pelly
	Pelly
	Pelly
	Pelly
	Pelly
	Pelly
	Pelly
	Pelly
	Piskorz
	Piskorz
	Piskorz
	Piskorz
	Piskorz
	Piskorz
	Piskorz

	Piskorz
	Piskorz
	Piskorz
	Piskorz
	Piskorz
	Piskorz
	Piskorz
	Piskorz
	Piskorz
	Portz
	Portz
	Portz
	Portz
	Portz
	Portz
	Portz
	Portz
	Portz
	Portz
	Portz
	Portz
	Portz
	Portz
	Portz
	Portz
	Portz
	Portz
	Portz
	Portz
	Portz
	Portz
	Portz
	Portz
	Portz
	Portz
	Portz
	Portz

	Portz
	Portz
	Queiroz
	Queiroz
	Queiroz
	Queiroz
	Queiroz
	Queiroz
	Queiroz
	Queiroz
	Queiroz
	Queiroz
	Queiroz
	Rakers
	Rakers
	Rakers
	Rakers
	Rakers
	Rakers
	Rakers
	Rakers
	Rakers
	Rakers
	Rakers
	Rakers
	Rakers
	Rakers
	Rakers
	Rakers
	Rakers
	Rakers
	Rakers
	Rakers
	Rakers
	Rakers
	Rakers
	Rakers

	Rakers
	Rakers
	Rakers
	Rakers
	Rakers
	Rakers
	Rakers
	Rakers
	Rakers
	Rakers
	Rakers
	Rakers
	Rakers
	Rakers
	Rakers
	Rakers
	Rakers
	Rakers
	Rakers
	Rakers
	Riboli-Sasco
	Riboli-Sasco
	Riboli-Sasco
	Riboli-Sasco
	Riboli-Sasco
	Riboli-Sasco
	Riboli-Sasco
	Riboli-Sasco
	Riboli-Sasco
	Riboli-Sasco
	Riboli-Sasco
	Riboli-Sasco
	Riboli-Sasco
	Riboli-Sasco
	Riboli-Sasco
	Riboli-Sasco
	Riboli-Sasco

	Riboli-Sasco
	Riboli-Sasco
	Riboli-Sasco
	Riboli-Sasco
	Riboli-Sasco
	Riboli-Sasco
	Riboli-Sasco
	Riboli-Sasco
	Riboli-Sasco
	Riboli-Sasco
	Riboli-Sasco
	Riboli-Sasco
	Riboli-Sasco
	Riboli-Sasco
	Riboli-Sasco
	Riboli-Sasco
	Riboli-Sasco
	Riboli-Sasco
	Riboli-Sasco
	Riboli-Sasco
	Riboli-Sasco
	Riboli-Sasco
	Riboli-Sasco
	Riboli-Sasco
	Riboli-Sasco
	Riboli-Sasco
	Riboli-Sasco
	Riboli-Sasco
	Riboli-Sasco
	Riboli-Sasco
	Riboli-Sasco
	Riboli-Sasco
	Riboli-Sasco
	Riboli-Sasco
	Rodrigues
	Rodrigues
	Rodrigues

	Rodrigues
	Rodrigues
	Rodrigues
	Rodrigues
	Rodrigues
	Rodrigues
	Rodrigues
	Rodrigues
	Rodrigues
	Rodrigues
	Rodrigues
	Rodrigues
	Rodrigues
	Rodrigues
	Rodrigues
	Rodrigues
	Rodrigues
	Rodrigues
	Rodrigues
	Rodrigues
	Rodrigues
	Rodrigues
	Rodrigues
	Saifan
	Saifan
	Saifan
	Saifan
	Saifan
	Saifan
	Saifan
	Saifan
	Saifan
	Saifan
	Saifan
	Saifan
	Saifan
	Saifan

	Saifan
	Saifan
	Saifan
	Saifan
	Saifan
	Saifan
	Saifan
	Saifan
	Saifan
	Saifan
	Sanderson
	Sanderson
	Sanderson
	Sanderson
	Sanderson
	Sanderson
	Sanderson
	Sanderson
	Sanderson
	Sanderson
	Sanderson
	Sanderson
	Sanderson
	Sanderson
	Sanderson
	Sanderson
	Sanderson
	Sanderson
	Sanderson
	Sanderson
	Sanderson
	Sanderson
	Sanderson
	Santana
	Santana
	Santana
	Santana

	Santana
	Santos
	Santos
	Santos
	Santos
	Santos
	Santos
	Santos
	Santos
	Santos
	Santos
	Santos
	Santos
	Santos
	Santos
	Santos
	Santos
	Santos
	Santos
	Savickas
	Savickas
	Savickas
	Savickas
	Savickas
	Savickas
	Savickas
	Savickas
	Savickas
	Savickas
	Savickas
	Savickas
	Savickas
	Savickas
	Savickas
	Savickas
	Savickas
	Savickas

	Savickas
	Savickas
	Savickas
	Savickas
	Savickas
	Savickas
	Savickas
	Savickas
	Savickas
	Savickas
	Savickas
	Savickas
	Savickas
	Savickas
	Savickas
	Savickas
	Schepart
	Schepart
	Schepart
	Schepart
	Schepart
	Schepart
	Schepart
	Schepart
	Schepart
	Schepart
	Schepart
	Schepart
	Schepart
	Schepart
	Schepart
	Schepart
	Schepart
	Schepart
	Schepart
	Schepart
	Schepart

	Schepart
	Schepart
	Schepart
	Schepart
	Schepart
	Schepart
	Schepart
	Schepart
	Schepart
	Schepart
	Schepart
	Schepart
	Schepart
	Schepart
	Schepart
	Schepart
	Schepart
	Schepart
	Schepart
	Schepart
	Schepart
	Schepart
	Schepart
	Schepart
	Schepart
	Schepart
	Schepart
	Schepart
	Schepart
	Schepart
	Schepart
	Schepart
	Schepart
	Schepart
	Schepart
	Schepart
	Schepart

	Schepart
	Schepart
	Schepart
	Schepart
	Schepart
	Schepart
	Schertzer
	Schertzer
	Schertzer
	Schertzer
	Schertzer
	Schertzer
	Schertzer
	Schertzer
	Schertzer
	Schertzer
	Schertzer
	Schertzer
	Schertzer
	Schertzer
	Schertzer
	Schertzer
	Schertzer
	Schertzer
	Schertzer
	Schertzer
	Schertzer
	Schertzer
	Schertzer
	Schertzer
	Schertzer
	Schertzer
	Schertzer
	Schott
	Schott
	Schott
	Schott
	Schott

	Schott
	Schott
	Schott
	Schott
	Schott
	Schott
	Schott
	Schott
	Schott
	Sideris
	Sideris
	Sideris
	Sideris
	Sideris
	Sideris
	Sideris
	Sideris
	Sideris
	Sideris
	Sideris
To improve the usability and acceptability of text messages, key informants sugg	Singh
	Singh
	Singh
	Singh
	Singh
	Singh
	Singh
	Singh
	Singh
	Singh
	Singh
	Singh
	Singh
	Singh
	Singh
	Singh
	Singh

	Singh
	Singh
	Singh
	Singh
	Singh
	Singh
	Singh
	Singh
	Singh
	Singh
	Singh
	Singh
	Singh
	Singh
	Singh
	Singh
	Singh
	Singh
	Singh
	Singh
	Singh
	Singh
	Singh
	Singh
	Singh
	Singh
	Singh
	Singh
	Singh
	Singh
	Singhal
	Singhal
	Singhal
	Singhal
	Singhal
	Singhal
	Singhal
	Singhal

	Singhal
	Singhal
	Singhal
	Singhal
	Singhal
	Singhal
	Singhal
	Singhal
	Singhal
	Singhal
	Singhal
	Singhal
	Singhal
	Singhal
	Singhal
	Singhal
	Singhal
	Singhal
	Singhal
	Singhal
	Singhal
	Singhal
	Singhal
	Singhal
	Singhal
	Singhal
	Singhal
	Singhal
	Singhal
	Singhal
	Singhal
	Singhal
	Singhal
	Singhal
	Singhal
	Singhal
	Singhal

	Sivakumar
	Sivakumar
	Sivakumar
	Sivakumar
	Sivakumar
	Sivakumar
	Sivakumar
	Sivakumar
	Sivakumar
	Sivakumar
	Sivakumar
	Sivakumar
	Sivakumar
	Sivakumar
	Sivakumar
	Sivakumar
	Sivakumar
	Sivakumar
	Sivakumar
	Sivakumar
	Sivakumar
	Sivakumar
	Sivakumar
	Sivakumar
	Sivakumar
	Sivakumar
	Sivakumar
	Sivakumar
	Sivakumar
	Sivakumar
	Sivakumar
	Sivakumar
	Sivakumar
	Sivakumar
	Sivakumar
	Sivakumar
	Sivakumar

	Sivakumar
	Sivakumar
	Sivakumar
	Sivakumar
	Sivakumar
	Sivakumar
	Sivakumar
	Sivakumar
	Sivakumar
	Sivakumar
	Sivakumar
	Sivakumar
	Sivakumar
	Sivakumar
	Sivakumar
	Sivakumar
	Sivakumar
	Sivakumar
	Sivakumar
	Sivakumar
	Sivakumar
	Sivakumar
	Sivakumar
	Sivakumar
	Sivakumar
	Sivakumar
	Sivakumar
	Sivakumar
	Sivakumar
	Sivakumar
	Sivakumar
	Sivakumar
	Sivakumar
	Sivakumar
	Sivakumar
	Sivakumar
	Sivakumar

	Sivakumar
	Sivakumar
	Sivakumar
	Sivakumar
	Sivakumar
	Sivakumar
	Sivakumar
	Sivakumar
	Sivakumar
	Sivakumar
	Sivakumar
	Sivakumar
	Sivakumar
	Sivakumar
	Sivakumar
	Sivakumar
	Sivakumar
	Sivakumar
	Sivakumar
	Sivakumar
	Sobreiro
	Sobreiro
	Sobreiro
	Sobreiro
	Sobreiro
	Sobreiro
	Sobreiro
	Sobreiro
	Sobreiro
	Sobreiro
	Sobreiro
	Sobreiro
	Sobreiro
	Sobreiro
	Sobreiro
	Sobreiro
	Sobreiro

	Sobreiro
	Sobreiro
	Sobreiro
	Sobreiro
	Sobreiro
	Sobreiro
	Sobreiro
	Steinman (2020/4/e
	Steinman (2020/4/e
	Steinman (2020/4/e
	Steinman (2020/4/e
	Steinman (2020/4/e
	Steinman (2020/4/e
	Steinman (2020/4/e
	Steinman (2020/4/e
	Steinman (2020/4/e
	Steinman (2020/4/e
	Steinman (2020/4/e
	Steinman (2020/4/e
	Steinman (2020/4/e
	Steinman (2020/4/e
	Steinman (2020/4/e
	Steinman (2020/4/e
	Steinman (2020/4/e
Reducing Financing/costs for partnership	Steinman (10.21037/
	Steinman (10.21037/
	Steinman (10.21037/
	Steinman (10.21037/
	Steinman (10.21037/
	Steinman (10.21037/
	Steinman (10.21037/
	Steinman (10.21037/
	Steinman (10.21037/
	Steinman (10.21037/
	Steinman (10.21037/
	Steinman (10.21037/
	Steinman (10.21037/

	Steinman (10.21037/
	Steinman (10.21037/
	Steinman (10.21037/
	Steinman (10.21037/
	Steinman (10.21037/
	Steinman (10.21037/
	Steinman (10.21037/
	Steinman (10.21037/
	Steinman (10.21037/
	Steinman (10.21037/
	Steinman (10.21037/
	Steinman (10.21037/
	Steinman (10.21037/
	Steinman (10.21037/
	Steinman (10.21037/
	Steinman (10.21037/
	Steinman (10.21037/
	Steinman (10.21037/
	Steinman (10.21037/
	Steinman (10.21037/
	Steinman (10.21037/
	Steinman (10.21037/
	Steinman (10.21037/
	Steinman (10.21037/
	Steinman (10.21037/
	Steinman (10.21037/
	Steinman (10.21037/
	Steinman (10.21037/
	Steinman (10.21037/
	Suen
	Suen
	Suen
	Suen
	Suen
	Suen
	Suen
	Suen

	Suen
	Suen
	Suen
	Suen
	Suen
	Suen
	Suen
	Suen
	Suen
	Suen
	Suen
	Suen
	Suen
	Suen
	Suen
	Suen
	Teo
	Teo
	Teo
	Teo
	Teo
	Teo
	Teo
	Teo
	Teo
	Teo
	Teo
	Teo
	Teo
	Teo
	Teo
	Teo
	Teo
	Teo
	Teo
	Teo
	Teo

	Teo
	Teo
	Teo
	Teo
	Teo
	Teo
	Teo
	Teo
	Teo
	Teo
	Teo
	Teo
	Teo
	Teo
	Teo
	Teo
	Teo
	Teo
	Teo
	Teo
	Teo
	Teo
	Teo
	Teo
	Teo
	Teo
	Teo
	Teo
	Teo
	Teo
	Teo
	Teo
	Teo
	Teo
	Teo
	Teo
	Teo

	Teo
	Teo
	Teo
	Teo
	Teo
	Teo
	Teo
	Teo
	Teo
	Teo
	Teo
	Teo
	Teo
	Thomas
	Thomas
	Thomas
	Thomas
	Thomas
	Thomas
	Thomas
	Thomas
	Thomas
	Thomas
	Thomas
	Thomas
	Thomas
	Thomas
	Thomas
	Thomas
	Thomas
	Thomas
	Thomas
	Thomas
	Thomas
	Thomas
	Thomas
	Thomas

	Thomas
	Thomas
	Thomas
	Thomas
	Thomas
	Thomas
	Thomas
	Thomas
	Thomas
	Thomas
	Thomas
	Thomas
	Thomas
	Thomas
	Thomas
	Thomas
	Thomas
	Thomas
	Thomas
	Thomas
	Thomas
	Thomas
	Thomas
	Thomas
	Thomas
	Thomas
	Thomas
	Thomas
	Thomas
	Thomas
	Thomas
	Thomas
	Thomas
	Thomas
	Thomas
	Thomas
	Thomas

	Thomas
	Thomas
On the basis of these experiences, the physiotherapists suggested that in future	Thorup
	Thorup
	Thorup
	Thorup
	Thorup
	Thorup
	Thorup
	Thorup
	Thorup
	Thorup
	Thorup
	Thorup
	Thorup
	Thorup
	Thorup
	Thorup
	Thorup
	Thorup
	Thorup
	Thorup
	Thorup
	Thorup
	Thorup
	Thorup
	Thorup
	Thorup
Users (patients) highlighted the benefits of having multiple programmes ‘connected’ on a single digital platform, which healthcare professionals	Tighe
	Tighe
	Tighe
	Tighe
	Tighe
	Tighe
	Tighe
	Tighe
	Tighe

	Tighe
	Tighe
	Tighe
	Tighe
	Tighe
	Tighe
	Tighe
	Tighe
	Tighe
	Tighe
	Tighe
	Tighe
	Tighe
	Tighe
	Tighe
	Tighe
	Tighe
	Tighe
	Tighe
	Tighe
	Tighe
	Tighe
	Tighe
	Tighe
	Tighe
	Tighe
	Tighe
	Tighe
	Tighe
	Tighe
	Tighe
	Tighe
	Tighe
	Tighe
	Tighe
	Tighe
	Toft

	Toft
	Toft
	Toft
	Toft
	Toft
	Toft
	Toft
	Toft
	Toft
	Toft
	Toft
	Toft
	Toft
	Toft
	Toft
	Toft
	Toft
	Toft
	Toft
	Toft
	Toft
	Toft
	Toft
	Toft
	Toft
	Toft
	Toft
	Toft
	Toft
	Toft
	Toft
	Toft
	Toft
	Toft
	Toft
	Toft
	Toft
	Toft

	Toft
	Toft
	Toft
	Toft
	Toft
	Toft
	Toft
	Toft
	Toft
	Tsukada
	Tsukada
	Tsukada
	Tsukada
	Tsukada
	Tsukada
	Tsukada
	Tsukada
	Tsukada
	Tsukada
	Tsukada
	Tsukada
	Tsukada
	Tsukada
	Tsukada
	Tsukada
	Tsukada
	Tsukada
	Tsukada
	Van Emmenis
	Van Emmenis
	Van Emmenis
	Van Emmenis
	Van Emmenis
	Van Emmenis
	Van Emmenis
	Van Emmenis
	Van Emmenis

	Van Emmenis
	Van Emmenis
	Van Emmenis
	Van Emmenis
	Van Emmenis
	Van Emmenis
	Van Emmenis
	Van Emmenis
	Van Emmenis
	Van Emmenis
	Van Emmenis
	Van Emmenis
	Van Emmenis
	Van Emmenis
	Van Emmenis
	Van Emmenis
	Van Emmenis
	Van Emmenis
	Vandekerckhove
	Vandekerckhove
	Vandekerckhove
	Vandekerckhove
	Varnfield
	Varnfield
	Varnfield
	Vugs
	Vugs
	Vugs
	Vugs
	Vugs
	Vugs
	Vugs
	Vugs
	Vugs
	Vugs
	Vugs
	Vugs

	Vugs
	Vugs
	Vugs
	Vugs
	Vugs
	Vugs
	Vugs
	Vugs
	Vugs
	Vugs
	Vugs
	Vugs
	Vugs
	Vugs
	Vugs
	Vugs
	Vugs
	Vugs
	Vugs
	Vugs
	Vugs
	Vugs
	Vugs
	Vugs
	Vugs
	Waldman
	Waldman
	Waldman
	Waldman
	Waldman
	Waldman
	Waldman
	Waldman
	Waldman
	Waldman
	Waldman
	Waldman

	Waldman
	Waldman
	Waldman
	Waldman
	Waldman
	Waldman
	Waldman
	Waldman
	Waldman
	Waldman
	Waldman
	Waldman
	Waldman
	Waldman
	Waldman
	Waldman
	Waldman
	Waldman
	Waldman
	Waldman
	Waldman
	Waldman
	Waldman
	Waldman
	Waldman
	Waldman
	Wali 2021
	Wali 2021
	Wali 2021
	Wali 2021
	Wali 2021
	Wali 2021
	Wali 2021
	Wali 2021
	Wali 2021
	Wali 2021
	Wali 2021

	Wali 2021
	Wali 2021
	Wali 2021
	Wali 2021
	Wali 2021
	Wali 2021
	Wali 2021
	Wali 2021
	Wali 2021
	Wali 2021
	Wali 2021
	Wali 2021
	Wali 2021
	Wali 2021
	Wali 2021
	Wali 2021
	Wali 2021
	Wali 2021
	Wali 2021
	Wali 2021
	Wali 2021
	Wali 2021
	Wali 2021
	Wali 2021
	Wali 2021
	Wali 2021
	Wali 2021
	Wali 2021
	Wali 2023
	Wali 2023
	Wali 2023
	Wali 2023
	Wali 2023
	Wali 2023
	Wali 2023
	Wali 2023
	Wali 2023

	Wali 2023
	Wali 2023
	Wali 2023
	Wali 2023
	Wali 2023
	Wali 2023
	Wali 2023
	Wali 2023
	Wali 2023
	Wali 2023
	Wali 2023
	Wali 2023
	Wali 2023
	Wali 2023
	Wali 2023
	Wali 2023
	Wali 2023
	Wali 2023
	Wali 2023
	Wali 2023
	Wali 2023
	Wali 2023
	Wali 2023
	Wali 2023
	Wali 2023
	Wali 2023
	Wali 2023
	Wali 2023
	Wali 2023
	Watson
	Watson
	Watson
	Watson
	Watson
	Watson
	Watson
	Watson

	Watson
	Watson
	Watson
	Watson
	Watson
	Watson
	Yao
	Yao
	Yao
	Yao
	Yao
	Yao
	Yao
	Yao
	Yao
	Yao
	Yao
	Yao
	Yao
	Yao
	Yao
	Yao
	Yao
	Zolezzi
	Zolezzi
	Zolezzi
	Zolezzi
	Zolezzi
	Zolezzi
	Zolezzi
	Zolezzi
	Zolezzi
	Zolezzi
	Zolezzi
	Zolezzi
	Zolezzi
	Zolezzi

	Zolezzi
	Zolezzi
	Zolezzi
	Zolezzi
	Zolezzi
	Zolezzi
	Zolezzi
	Zolezzi
	Zolezzi
	Zolezzi
	Zolezzi
	Zolezzi
	Zolezzi
	Zolezzi
	Zolezzi

Extract	Summary of the extract (if needed)
Nurses believed SMS particularly	remember to take their medicine
Some nurses replied that most p	most patients had consistent access to network
Many nurses expressed concerns	(barrier) phone sharing, not having own phone - missing mes
not having a phone - one particip	TO - not having a phone ; (barrier) lack of phone accessibiilty
One nurse reported that a partic	(Facilitator) ability to change number of messages sent
As only the assigned nurse had ac	Only one nurse attached to one person's medical data
Some nurses suggested that SMS	Not useful for older people
One policy maker voiced concern	(barrier) Financing/cost of SMS delivery
Other nurses reported that there	(barrier) signal network problems
Healthcare providers also reporte	Not a functioning network
Some CVD nurses reported ongoi	data workflow
when doctors who were not part	Not entering appointment information into the system
Electricity was also a concern, and	(barrier) limited electiricty
] The ICT component of the traini	(faciltator) Training for ICT
Some nurses suggested that in so	Overloading patients with content (SMS messages)
Some CVD nurses reported ongoi	Finding/entering codes
as all levels of health provider we	increased access to medical records
as all levels of health provider we	better abilty to track management
The most stated reason for not using telemedicine during COVID-19 was incompatibility with s	
how to use the device	
Technology issues<br>related to the device I	
to poor internet	
providers either indicated that	
issues	
table 6	
communicating via	
identified face-to-face	
providers identified phone call-	
how to use the platform	of content
Perception that home blood pres	Home blood pressure more acccurate than hosptial
The health care team identified d	
("As a provider, I may have hundr	Alerting to direct attention rather than going through every
("As a provider, I may have hundr	Alerting to direct attention rather than going through every
When they share this data, the pa	patients want to know their data has been received
participants emphasized that inte	need for tech to be integrated into workflow

The health care professionals rep	
No standardized way to collect ar	
the health care professionals' pre	legal concerns of patient privacy hindered ability to integrate
privacy concerns	CONCERNS - data privacy
confidentiality concerns	CONCERNS - data confidentiality
When they share this data, the pa	transparency - patients want to know their data is kept priva
Easily and quick visualization of d	being able to sort data descriptively or show in a graph leads
("Clinicians don't want to see dai	See up/down trends versus all information
most preferred graphical represe	Preference for graphical representation as it reduces cogniti
medical complications ("We may	increased monitoring can prevent readmissions, events, sym
and to the provide the healthcare	immediate guidance
could help reduce information loa	
. Some patients use this service to	Worry that patients will exploit the system
services are limited to a few	telehealth services are not accessible to patients - long wait
analysis documented that some	inequitable access to care due to (barrier) Nepotism causes
telehealth is not suitable for all	telehealth is not suitable for all diseases
among patients about	low awareness of how (or when) to use the system
pointed out that the system	Devices breaking down
pointed out that the system	Internet problems
may also misuse the telehealth	Perception that HCPs might misuse the telehealth system/ov
The first is improper use by patier	improper use
The findings showed that some p	Concern of patient overreliance of the system
This is related to the nature of the	it has a lot of medical information that the patient may not u
activated for those patients who	view that some patients need it
services are limited to a few	telehealth services are limited to a few clinics and hospitals i
issues with being able to be	Perceived invasion of privacy - patients can contact 24/7
providers (22) said: This is a life-	Responsiblity/liability - I might be accountable if I misdiagno
the culture of Jordanian people,	preference for female doctors might limit uptake from male
some of the patients have some	Perception that some patients fear using technology
some of the patients have some	patients do not accept the change
substitution for a face-to-face	perception that there is a need for people who cannot go to
telehealth could be effective	only for patient follow-up and monitoring their status and m
substitution for a face-to-face	
responsibilities of diagnosing	Shared responsbilities - perception that this would be challe
(22) said: "How I can depend on	difficulty trusting in other HCPs' diagnosis
the culture of Jordanian people,	Workforce - lack of female healthcare professionals able to
areas lacked some services	clinical resources - in remote areas - The telehealth clinics in

[illegible] telehealth clinics two days a	data opening hours
[illegible] follow up, or schedule	
[illegible] telehealth could be effective	useful for managing
[illegible] telehealth could be effective	follow-up and monitoring their status
[Doctors] felt that they would be	scientifically tested
Poor credibility of the DHI, poor a	nd doubt about their continued availability
Poor credibility of the DHI, poor a	poor accuracy
[Doctors] felt that they would be	were based on practice guideline
Personalsable to patients: "Docto	to patient goals
language barrier: "Participants also mentioned the unavailability of apps in users' own languag	
Reminder function: "Both doctors	or a generic reminder for a nonspecified task (Braun and Qa
Appealing visual presentation, practical features: "Most doctors and patients liked the 5 apps'	
[illegible] with smartphone technology	
They felt that some apps are not	They felt that some apps are not detailed enough to capture
Older age, low education level: "A	
Older age, low education level: "A	low education
Adequate information provision (	Doctors generally criticized apps, like Braun and ESH, that la
Reminder function: "Both doctors	Both doctors and patients preferred apps, like Cora, that pro
Official endorsement: "Some doctors stressed the importance of official endorsement by, for i	
Privacy concerns: "Most patients	high-profile individuals.
Privacy concerns: "Most patients	high-profile individuals.
ne doctor suggested that profit-motivated app development may not lead to the best quality i	
Providing training for DHI users: "Most doctors and patients thought that some level of trainin	
Providing training for DHI users: "Most doctors and patients thought that some level of trainin	
Adequate information provision (amount, readability, quality): "Participants found Qardio's la	
Poor credibility of the DHI, poor accuracy of the DHI: "Doctors expressed concerns about the c	
One doctor suggested that profit-motivated app development may not lead to the best quality	
[Doctors] felt that they would be	or had been checked by doctors;
Poor credibility of the DHI, poor accuracy of the DHI: "Doctors expressed concerns about the c	
Inadequate amount of functions (	or a poor layout (Hyten and Braun) would confuse patients."
Adequate amount of detail and good usability for data entry function: "Participants favored ap	
Adequate amount of detail and g	well-organized
Inadequate amount of functions (	Too Complex- that too much detail (Hyten) or a poor layout
Inadequate amount of functions (	Too simple - concerned that apps with too few functions
Appealing visual presentation, pr	high-quality graphs and tables
Appealing visual presentation, practical features: "Most doctors and patients liked the 5 apps'	
Appealing visual presentation, pr	text

Adequate information provision (	Adequate information provision (amount, readability, quality
Adequate information provision (amount, readability, quality): "Participants found Qardio's la	
Inadequate amount of functions (	with too few functions (eg, Qardio and ESH) may lead to pat
Inadequate amount of functions (	conversely, that too much detail (Hyten)
DHI functions/features perceived	perceived as helpful
of the intention of the messages	
Lack of patient engagement with	Not all professionals viewed the graphs with the patients du
Lack of patient engagement with DHI: "Not all professionals viewed the graphs with the patien	
Lack of patient engagement with	passive patients - not engaging
Professionals' judgement that	and did not provide enough benefits to make it worthwhile."
and lack of HCPs education about these: "The optional motivational messages in our study we	
Meeting the HCPs where they are	, the professionals' opinions and preferences about technolo
Lack of patients' understanding a	ack of patients' understanding about the purpose of DHI fun
HCP education/training about the DHI and to explain/educate the patient about the DHI: "The	
HCP education/training about the DHI and to explain/educate the patient about the DHI: "The	
and lack of HCPs education abou	need for sufficient HCP education
helpful visualisations of data: "Th	
DHI enabling a more holistic and	more holistic
Patients' engagement with the DI	
DHI enabling a more holistic and	personalised approach to care
tool, some of the professionals	
The DHI enabling a more equal relationship / partnership between HCPs and patients: "Both p	
Patients' engagement with the DI	
DHI enabling a new/enhanced wa	a new/enhanced way to communicate with patients:
Professionals' judgement that	time-consuming
(facilitator - benefical for at-risk p	for high risk patients - having BP values that could change su
telemonitoring may allow for imp	TO - Increased accesses to resources - improved access to pe
could] be even a more useful too	for women to self-identify
at high risk for preeclampsia)	
(patients at high risk for	
appropriate equipment, such as	BP cuffs
currently available	currently available telemonitoring systems are not tailored f
because it would be easier for	
highlighted the need for patient	Alerts automatically-generated from the patient data
highlighted the need for patient	Alerts automatically-generated from the patient data
educates patients on self-	app the fosters patients to take an active role of their care
should include evidence-based ac	use of evidence-based alerts

barriers (eg, Financing/cost of a	
the financial means to	some patients can't cover the Financing/costs of the BP mor
throwing [blood pressure	but because my app doesn't tell me to do anything, I stay ho
throwing [blood pressure	oh, like I'm waiting for somebody to get back to me
who currently manage their	work, and I'm not really into, like, the whole
(Barrier- wouldn't be used for the	Not a high perceived need for this DHI for most patients
that can incorporate evidence-based protocols and standards for PHRPE to facilitate their deci	
patients are inserting into their	checking my phone all the time and I don't think that should
patients are inserting into their	hen it becomes a safety thing, because I'm not checking my
will help motivate them	Convincing the patient off the added benefit/need to do so
should facilitator decision-making for clinicians ; Providers suggested displaying BP results, me	
should facilitator decision-making for clinicians ; Providers suggested displaying BP results, me	
that can incorporate evidence-ba	evidence-based protocols and standards for PHRPE
time demanding, it's	
could] be even a more useful too	and come to triage maybe in time before they get too sick
increase clinician workload.	Increase workload - off-clinic hours, timely assessments
increase clinician workload.	alerting in off-work hours
HCPs perceive the DHI as clinically	perform more titrations and reach target doses faster signif
Lack of financial reimbursement f	as well as arrange physician remuneration
Lack of financial reimbursement f	he financial resources necessary to acquire any required add
the DHI is well-integrated into pa	integrated into routines
the DHI solves a practical problen	perceived need for it
Lack of financial reimbursement f	lack of reimbursement
HCPs' positive attitude towards th	data (perceived) positive perceptions
. The tasks of clinicians involved ir	a complete training plan
Poor HCP preparedness for the D	poor training
HCPs perceive the DHI as easy to	ease to use
dedicated staff to support the DH	workforce - nurses
dedicated staff to support the DH	dedicated nursing staff
time-consuming in the absence o	lack of NPHW - Workforce - absensce of help ,
. The tasks of clinicians involved ir	Clearly defined roles
Negative impact on HCPs' worklo	(already) having limited time
the DHI has no negative influence	no negative influence on patient-HCP rapport
Negative impact on HCPs' worklo	a time-consuming intervention is not likely to be used,
understanding: ("...promotes	
Accessibility limitations: ("...only s	
Accessibility limitations: ("...only s	

dependent on whether	
adjunct to usual care, does not	
(“...useful aid when titrating	
adjunct to usual care, does not	
have avoided admissions”;	Admissions avoidance
(“...allows trends to be spotted	of diganosis
(“...allows trends to be spotted	
(“...allows trends to be spotted	
(“...useful aid when titrating	Medication optimizations
gains due to fewer	workflow - due to fewer appointments
(“Sometimes can be difficult to	On-boarding can be complicated and time-consuming
(“Sometimes can be difficult to	manage the additional alert
(“Sometimes can be difficult to	
User-friendly	User-friendly
Some participants were deterred	didn't know what they were getting into my signed up for in
Some participants were deterred	Didn't know how to sign
(screening) uptake by males was	Males did screening less
Occasional poor network respons	Poor network responsiveness
The CHW found her work empow	Feeling that work is empowering
Screening was well received by th	Positive reception of screening by the community
Increased patient-clinician engag	Increasing self-management - Maintaining, pushing a little m
e were able to access how things	to blood pressure data
and inform individualized treatm	
saved time and made the visit mo	for more patient-focused consulation
Showing data over the past 2 wee	or selecting specific data
The health professionals reporte	Technical problems
expressed that not all health	Lack of health care systems that can handle DHI
Proper integration into existing p	Need for funcational/operational integration of new system
Patients sometimes stopped usin	Lack of incentivization
patients are inserting into their	hen it becomes a safety thing, because I’m not checking my
mentioned that receiving the BP	placed an expectation on them to reach out to their patients
to help patients troubleshoot	shifted to tech support - tech side
how to adequately coach patient	Training patients
would only be notified when a pa	trends
would only be notified when a pa	graphical
Showing data over the past 2 wee	personalizing visualtion

mentioned using MAs (medical	
successful hypertension	team-based care
centralization and different care t	
centralization and different care t	Organization - clearly defined roles and models
would only be notified when a pa	would only be notified when a patient’s BP was too high or l
Efficiency with remote blood pre	Improved efficacy of remote BP monitoring - more insight ir
The health professionals mention	Improved monitoring of health status
of the health professionals noted	more of a comprehensive image of BP levels
the potential of remote BP monit	better evaluation of progress
implied that it improved clinic eff	Improved clinical efficiency
('I think the key thing that it does	
("We have a tighter relationship.	Improved patient/clinician relationship
It saves a lot of time when you’re	time saving
saved time and made the visit mo	saved time and allowed for more patient-focused consulatio
Time lost (""“But it does put that a	look through it [BPc readings]
health professionals discussed no	Losing less engaged patients
health professionals often had	Increased calls due to tech support
received a deluge of BP data in	Increased worload - time lost
received a deluge of BP data in	Increased worload - time lost
reported that it was not feasible	real-time checking not feasible
Convenience of telehealth visits (patients living in rural areas, having issues with transportatio	
Telehealth visits are not as compr	to pressure kit, scale for weight measurement, blood glucos
Teleheath visits cannot substitute	patients with multiple chronic conditions, where a patient ha
Issues related to technology unreliability (internet connection) and proficiency among clinician	
Issues related to technology unreliability (internet connection) and proficiency among clinician	
Challenges in getting an interpret	
Issues related to technology unreliability (internet connection) and proficiency among clinician	
Issues related to technology unre	low-income
Convenience of teleheath makes	the locations from which they take calls can be distracting
Issues related to technology unreliability (internet connection) and proficiency among clinician	
Convenience of teleheath makes	, not following through lab tests prior the visit
Convenience of teleheath makes	less attention
Delays and scheduling issues occu	patients are unprepared;
Mismatched expectations of telel	of telehealth efficiency and wait times
Convenience of teleheath makes	makes the patients value it differently from in-person visit
Teleheath visits cannot substitute all in-person visits (meeting with a new patient, patients wit	
Teleheath visits cannot substitute all in-person visits (meeting with a new patient, patients wit	

Challenges in getting an interpret	interpreter
Mismatched expectations of tele	scheduling issues
Mismatched expectations of telehealth efficiency and wait times due to delays and scheduling	
Convenience of telehealth visits (	mobility challenges
Convenience of telehealth visits (	better for those having issues with transportation
Mismatched expectations of telehealth efficiency and wait times due to delays and scheduling	
The anticipated benefits (e.g., red	
The CDS tool for ordering and managing the duration of monitoring may decrease the autonor	
The CDS tool for ordering and ma	decreased autonomy
The CDS tool for ordering and managing the duration of monitoring may decrease the autonor	
Disagreement among clinicians on whether they should reduce telemetry monitoring ordering	
The process needed to go throug	not adapted to the workflow
The process needed to go through for ordering with the CDS tool may be disruptive to HCPs' w	
Disagreement among clinicians o	and not use the CDS tool);
The CDS tool can be used inappropriately, sometimes intentionnally by some clinicians to still	
The CDS tool can be used inappropriately, sometimes intentionnally by some clinicians to still	
Disagreement among clinicians o	and not use the CDS tool);
Disagreement among clinicians o	
The anticipated benefits (e.g., red	reducing Financing/costs of telemetry
The anticipated benefits (e.g., red	reduction in inappropriate telemetry monitoring use,
The anticipated benefits (e.g., red	reduced inappropriate testing
The CDS tool helps HCPs stay up t	helps deliver standard care
The CDS tool helps HCPs stay up t	
The anticipated benefits (e.g., red	patient experience
The anticipated benefits (e.g., red	reduction in inappropriate telemetry monitoring use,
“creative people” and can	
adoption of telehealth towards	data colleague expectations
“creative people” and can	reluctance to change/new
scrutiny, reliability,	not open for innovation and following clear-cut rules
, empowering patients about mar	data self-management
Harder to define patients' cohort	who to refer
suggested that doctors' requests	data evidence-based
reducing Financing/cost	due to fewer hospitalizations
The need for a telehealth nurse to	paying a nurse to specialise in telehealth
(does the DHI fit in the way HCPs	concerns about if the DHI fits into workflow
Lack of clear clinical guidelines th	
A health care professional (e.g.,	data nphw

better recovery for the patients b	recovery
As a commissioner, it might reduce people going into	
whether the integrity and profess	data worker protection
concerns of safety of the patient	
Uncertainty around clinical safety	data clinical safety
Increased workload of primary ca	more calls to doctors from patients
Availability/accessibility of requir	TO -Accessibility of moblie phones, even in remote areas
Older age; illiteracy; digital illitera	(barrier) illiteracy - ability to read the text messages
Older age; illiteracy; digital illitera	(barrier) digital illiteracy
Older age; illiteracy; digital illitera	FOR OLDER (barrier) old age - ability to read the text messag
Older age; illiteracy; digital illitera	(barrier) disadvantages to rural areas
DHI containing reliable and high-c	Need for reliable, high-quality information from DHI
Lack of financial resources for DH	software development Financing/costs
Lack of financial resources for DH	Financing/cost of SMS delivery
Inadequate patient record syster	Existing, inadequate patient record systems (to implement D
Lack of ... commitment from the a	Lack of committment from authorised bodies in hopitals
governmental and multi-sectorial	uch as establishing a separate department to monitor the pr
governmental and multi-sectorial	need for multi-sectoral collaboration
DHI linked with a reliable and tru	If linked to a health system - increased trustworthiness from
Limited technical workforce("HCV	Limited technical workforce for implementation
High general workload of HCPs ("	and no one is going to record as we don’t have time -lack of
HCPs' perceived clinical usefulnes	Improve adherence to medication/treatment
time-saving ("Some stated that g	TO services - Time-saving, reduced need to travel
time-saving ("Some stated that g	less time to travel to appointments
High general workload of HCPs ("	Increased workload for data entry
Anxiety in people who test positive for AF (65%)	
, validation by scientific associatio	validated through scientific organzations
valid comparisons between devices/apps	
randomized controlled trials of specific apps or devices,	
clear reporting of scientific validation for devices/apps	
,, testimonials/interviews,	
reporting of case studies)	
more rigorous regulations by Hea	checking by regulations
risk of bleeding due to OAC (oral anticoagulation) against potential but unproven benefit (39%	
false reassurance in case of nega	False reassurance
Potential for educating and prompting patients and family members on strategies for self-mon	
HCPs are concerned that the embedded AI would not be capable of supporting precision med	

(if it is too large of a change, HCPs may not adopt it)	
The advanced capabilities of soci	showing trends of data
They are not the service provider. I do not want them to be the service provider. I want them	
Need for an informed by patient/healthcare provider-centred design that is grounded in exist	
Need for an informed by patient/healthcare provider-centred design that is grounded in exist	
Need for a dedicated, hospital-integrated data management system with requisite staffing, su	
Perceived lack of evidence of safe	of its safety
Need for a dedicated, hospital-integrated data management system with requisite staffing, su	
Need for an informed by patient/healthcare provider-centred design that is grounded in exist	
Perceived lack of evidence of safety for patients and of value added beyond current remote pa	
Perceived lack of evidence of safe	concerns of patient safety
Potential for educating and prom	engaging family members to help with self-management
Limited responsibility for direct p	
They are not the service provider	perceptions of limitations
HCPs worry that the implementat	reroute attention away from complex health-related proble
I like them as the mediator almost. Like they are the	Good as mediator
I like them as the mediator almost. Like they are the	but not as taking over
Limited responsibility for direct patient care as there is potential for missing nuances in chang	
Limited responsibility for direct p	
Limited responsibility for direct p	ability to pick up nuances
HCPs are concerned that the embedded AI would not be capable of supporting precision med	
Need for a dedicated, hospital-integrated data management system with requisite staffing, su	
Need for a dedicated, hospital-in	technical support
The advanced capabilities of social robots in data collection, collation and transmission would	
The advanced capabilities of soci	data collection
The advanced capabilities of social robots in data collection, collation and transmission would	
The advanced capabilities of social robots in data collection, collation and transmission would	
Limited responsibility for direct p	missing nuances
They do not have the ability to do	they might miss important things
Potential for educating and prompting patients and family members on strategies for self-mon	
so the bulk of the time I have with	more time for patient conversation
The main concerns were about th	data security concerns
For the multiple hierarchical regre	eHealth literacy
technical reliability of software (2	technical reliabilty of software
Concerns about reimbursement	reimbursement
For the multiple hierarchical regre	performancy expectancy
Concerns about hygiene were me	hygiene

followed by the trustworthiness	trustworthiness of content
lack of or limited options for acce	options for access to patients
Despite the challenges of implem	
Few-patient suitable and applicat	not suitable for all patients
Some HCPs would like more and r	insecurity
Use of Fast@home by HCPs was limited by patients' motivation, how informed they were abo	
Lack of communication between	between temporary flex workers and HCP
Use of Fast@home by HCPs was l	mental capacity
Technical issues were encountered (e.g.: problems administering accounts; internet connectio	
Lack of evidence of the added val	Lack of evidence
Technical issues were encountere	internet connection
Technical issues were encountere	lack of computers
Implementating the eRehabilitati	not priotized by managers
The DHI is perceived as a good solution to compensate for less time with patients.	
Use of the DHI was limited by HCPs' motivation to change to adopt the tool and to motivate ot	
Use of Fast@home by HCPs was limited by patients' motivation, how informed they were abo	
Use of the DHI was limited by HCPs' motivation to change to adopt the tool and to motivate ot	
Use of the DHI was limited by HCI	to change
Use of the DHI was limited by HCI	HCPs were less likely motivated to use the tool if other colle
Use of Fast@home by HCPs was l	how informed they were - those with lower explanation (kno
Some HCPs would like more and regular training to keep up their skills for using the eRehabilit	
Implementating the eRehabilitation tool creates conflicts with HCPs' already busy schedules ar	
Technical issues were encountere	problems administering accounts
Despite the challenges of implem	
Despite the challenges of implem	
Despite the challenges of implem	
Despite the challenges of implem	accessible helpdesk
Despite the challenges of implementing the tool, support by clinical champion (who sent regul	
Implementating the eRehabilitation tool creates conflicts with HCPs' already busy schedules ar	
The eRehabilitation tool allows more multidsciplinary work in the rehabilitation care of patient	
Greater access to evidence-based	greater access to evidence-based care for patients
sustaining reliable internet conne	data internet/wifi
[illegible] the group meeting	sufficient clinic space is lacking
appreciation of accurate informa	The DHI provided accurate information about nutrition
sustaining adequate clinic staff	having/sustaining adequate clinical staff
availability to recruit for and adm	having the staff to administer the trainings
aligning the group sessions on da	scheduling challenges

Allows for increased resources fo	increased resources for patients are available
good for patients living further a	benefical for patients living further away from clinics
HCP perception of clinical benefit	seeing a clinical benefit is a facilitator
Technological glitches - mainly difficulties with Bluetooth pairing ("Technological glitches impa	
Technological glitches - mainly di	Bluetooth pairing between digital scales, blood pressure cuf
Technological glitches - mainly di	difficulties of bluetooth pairings
Financing/cost; time;	
clinic/staff resources ("Challenges for all stakeholders revolved around Financing/cost, time, p	
poor technology integration to clinical workflow ("technology	clinical workflow
Additional workload through DHI	That’s what’s so overwhelming, people expect that I’m goin
Additional workload through DHI	
Additional workload through DHI	
provider education and training;	
clinic/staff resources ("Challenges for all stakeholders revolved around Financing/cost, time, p	
clinic/staff resources ("Challenge	uncertainty of genetic testing results
Financing/cost; time;	
clinic/staff resources ("Challenge	clinic/staff resources."
clinic/staff resources ("Challenge	workflow
HCP perception of benefits to the patient-provider relationship	can speed medication adjustments - we’re able to titrate th
HCP perception of benefits to the patient-provider relationship	even increase adherence
HCP perception of benefits to the patient-provider relationship	strengthen relationships
Technological glitches - mainly di	was a serious barrier to implementation and required signif
Technological glitches - mainly di	time/effort to set up the devices
Additional workload through DHI	
Additional workload through DHI	
Positive perception that BPTrack	clinic workflow
Data was appealing to have, The pharmacist also appreciated access to readings, commenting	
Perception of utility of collecting the patient log in a mobile device “It’s a very portable record	
Perception of utility of collecting	
Desire for accurate measurement	
The stakeholders also indicated a	
perceived reservations of timely r	
Concern that the data from the application did not go direclty into the EHR. The provider had t	
recommendations for the frequen	
Perceived benefits when integrat	into routine practice
Belief that solutions such as BPTr	
Lowering the provider-side burden of managing the data will be necessary, mainly through int	

The stakeholders also indicated a	
Concern that the data from the application did not go direclty into the EHR. The provider had t	
concerns about long-term sustain	getting that data into [the EHR]…
Ensuring ease of use and minimal	So, I worry that if they have to type in their readings, that th
concerns about accuracy - The ph	having the cuff on correctly - "Make sure the blood pressure
perceived reservations of timely r	
BPTrack to manage hypertensive	
Perception that it might not work	need for non-digital options - not for everyone
Perception that it might not work	
Perception that regular visits are	less openness over the phone - things you won't, they won't
The stakeholders also indicated a	
Perception that regular visits are necessary and help the patient feel more open to talking ("I s	
Perception of utility of collecting t	Perception of utility of collecting the patient log in a mobile
Ensuring ease of use and minimal	
Ensuring ease of use and minimal	
Ensuring ease of use and minimal	"It has to be very simplistic, especially for our older patients
Ensuring ease of use and minimal	minimize workload burden
The application lent itself to improved patient education "I liked that it graphs the numbers ou	
The application lent itself to improved patient education "I liked that it graphs the numbers ou	
The application lent itself to impr	improved patient education
The application lent itself to impr	readability
Lowering the provider-side burde	lowering workload burden for HCPs
Data was appealing to have, The pharmacist also appreciated access to readings, commenting	
Concerns about the long-term su	"I think… like anything else it… when it starts, the patient m
Concerns about the long-term sustainability of frequent monitoring - the physician explained	
Desire for accurate measurement	
Appreciation of access to blood p	more accurate than recall
Appreciation of access to blood pressure readings and acceptance of incoporating the BPTrack	
The stakeholders also indicated a	
Perception of utility of collecting t	
Data was appealing to have, The pharmacist also appreciated access to readings, commenting	
Positive perception that BPTrack	
Concern that the data from the a	
perceived reservations of timely r	
	who used social media

data storage	
	challenges
Respondents who utilized smart devices to monitor their own health	
	Concerns
high costs for patients	
	Lack of
and security	
concerns regarding data security/privacy	
	lack of relevant training
Respondents who attended ESC c	courses on digital health
	lack of user-friendly technologies
lack of defined clinicial pathways (39%)	
time-consuming (28%), lack of financial reimbursement (52%)	
measuring the steps may not be	intensity and duration
If simple explanations are provide	Health literacy
their concern on some patients’	
notifications. I think those	push notifications
the patient education	on DHI - and patient not consulting doctor anymore
the patient education	patient not consulting doctor anymore
especially for an extended	
doctors especially during	need for this feature
them know that the information	monitored
them know that the information	
healthcare provider is	HCP advocating for app use
towards the app.	
motivating the users could	and the potential benefits of the app.

presentation is really nice,	
summary	report
great to be able to	
benefits	
Working with insurers to accept r	working with them
clinicians perceived that telehealt	TO services
ability to deliver more individualiz	
perception that telehealth delivery changed group dynamics and lacked peer support, which is	
Financing/cost of social interactio	
patients are not as accountable o	
issue, which adversely	long waiting time
patients are not as accountable o	
an email address and they do	
equipment, internet, and data	equipment
an email address and they do	not having email address
an email address and they do	o not have home internet or home Wi-Fi."
inequitable access (challenges wi	Technical skills
remote delivery reduced the capa	nformation about home life that can guide support and refe
remote delivery reduced the capability to gather incidental patient information, such as non-v	
challenges with funding in the private health	
remote delivery reduced the capability to gather incidental patient information, such as non-v	
difficulty in undertaking physical a	
difficulty managing large group d	space
and software user-caps	software user-caps
Physical space was the strongest	limitations on space capacity
and challenges with funding in th	insurance did not fund remote CR delivery
encounters create	
Incorporating family members to help out with patients' lack of technology skills	
perception that telehealth delive	the lack of social support from DHI
fear of cheaper Financing/cost of	to their job security
Clinician concern that heath servi	low trust in the organziation deciding telehealth future
limited videoconference group ca	limitations on space capacity
of use of pre-recorded data - but	
increased documentation require	Documentation
takes longer to document the encounter in the EMR system than it does to perform the encou	
think the clinicians can either.	
me three hours to do in a group	

HCPs prefer an e-health tool integ	
Potential of using an e-tool to gai	easy Access to patient data
Multi-language app is favoured by	
patients' inability to use technolo	
HCPs want an e-tool that provide	more interaction with a member of the clinical team
HCPs worry that it would be diffic	patient seek human touch
Some HCPs have concerns over c	CONCERNS -data privacy
Some HCPs have concerns over c	CONCERNS - data confidentiality
The e-tool could help reduce Fina	
HCPs believe that the use of e-to	empower patients
HCPs believe that the use of e-to	reassurance
The e-tool could increase anxiety	
The messaging function in an e-to	data responding
them—someone is monitoring,	patients feel reassured
interviewed were acutely aware	
noted	
agreed that "sometimes it's a bit	not appropraite for those with low health literacy
physicians interviewed appeared	more accurate - better now than manually recording from pa
agreed that "sometimes it's a bit	not appropraite for those with low social support
the remote BP monitoring data	would better reflect their BP in ordinary life
it's easier to fake it - multiple	they can't fake it
agreed that "sometimes it's a bit	
from the system surfaced as a	readings had been uploaded to the server
about whether telemedicine	not sure if the quality of care is the same
apprehension centered on the	not sure if their messages were received by patients
participant failed to upload their	lack of communication from the system if technical problem
of visibility of certain	why BP was high?
a bit [impaired].	hearing
acknowledging the	more information in-person
Face-to-face, if I	lack of body language
Face-to-face, if I	lack of body language
lack of visibility (clinicians),	
Face-to-face, if I	patients might not be paying attention
participant failed to upload their	failed to upload their readings,
patients' irregular submission of	patients' irregular submission of data ,
clinicians reported that some	canceling - patients still coming in instead of doing virtual ca
miscommunication over the	

miscommunication over the	barriers over the phone
also highlighted that some	barriers over the phone
also highlighted that some	
concerned about overblown	
might expect an immediate	
agreed that “sometimes it’s a bit	Concerns
have	need this before
trust relationship resulted in	
happening, staff reminded	
such a case might result in grave	
emphatically that, if 2	
agreed that “sometimes it’s a bit	
clinicians reported that some	patients still coming in instead of doing virtual calls
interviewed were acutely aware	
interviewed were acutely aware	
such a case might result in grave	of the care provided
confidence in the remote	lacked trust in remote consultations
readings they had submitted to	trust of the evidence that the HCP is reporting
monitoring program seems to	
physicians interviewed appeared	of the readings
it's easier to fake it - multiple	of the BP readings
readings...and I cannot stop	
in this program were “more	patients listened more - more receptive to advice
compliments,	
compliments,	
the remote BP monitoring data	remove white coat effects
was easier to build interpersonal	
was easier to build interpersonal	understanding patients' lifesyle and questions
acknowledging the	convenience of care
acknowledging the	time savings
primarily geared	of the clinic - if the data is not accurate
readings...and I cannot stop	volume - lots of readings
the program	
the program	perceived it was suitable
practice staff knowledge	
issues	not loading or downloading properly
but unfortunately, they have not	

app was clicked on to open the	‘Refreshing’ issues
licence per practice is high and	each licence per practice is high
licence per practice is high and	reserach funding necessary
leading to the removal of	Computer issues
difficulties connecting to the	connecting to the practice server
health networks)	can't support due to other prioritizes
described doctors forgetting to	
our nurse to start using it now	
regulations increased the time	digital health regulation
regulations increased the time	changes to the definition of a “medical device"
regulations increased the time	
workforce	workforce shortages - nurse leaving
the resources were more	using nurse instead of GP
health networks)	compared to other things, not a priority
be difficult due to competing	
many stakeholders (Australia	Time to build relationships with many stakeholders - working
regulations increased the time	institutional risk and required additional legal review and
be difficult due to competing	
be difficult due to competing	
spontaneously logging out which	
consuming and challenging	
iPDAs helped patients understar	
(2) led to the implementation of	
the iPDAs improved decisional qu	
(3) increased SDM (shared decisio	
Perception of patient satisfaction	
One clinician wrote that the iPDA	
Feeling that visits with an iPDA to	
Feeling that visits with an iPDA to	
the iPDAs improved decisional qu	
complement yet not a	
presented in one	
people, in particular the	(lack) access computer
don’t own a computer,	
don’t own a computer,	or a phone or something and are able to
don’t own a computer,	or a phone or something and are able to
that is easy to use were	up-to-date

disadvantage that was raised	only in swedish
knowledge of	didn't needa lot of digital literacy to understand/use
choosing the pragmatic use of	the functions in the system that suited their organisation
to the DPE early in the	
to the DPE early in the	
choose	able to choose length of session
patients themselves have a	DPE to access the information
patient’s choice to use the	
that enhanced	
people, in particular the	unable to log in
communication when using the	only one-way communication
communication when using the	
was considered important	evalution of the DHT
disadvantage that was raised	
people, in particular the	
highlighted the	
that they became accustomed	
teething trouble, but	Unfamiliarity
how	
which functions to activate in	ability to adapt it based on the indivdiual work constraints
educational	patient programmes
perceived that the head of	the department
towards the DPE were	with a positive attitude towards the DPE
heads of department were not	heads of department were not involved practically
secondary prevention	might affect secondary prevention
instructions to patients on	message function was not supervised at all times.
communication when using the	
and motivation towards trying	
for digital alternatives	
for digital alternatives	
not willing to use digital	not willing
for digital alternatives	
instructions to patients on	CLEAR Communication
not willing to use digital	CONCERNS
with the	patient during visits,
to the DPE early in the	early in the rehabilitation process and the whole year
to the DPE early in the	to the DPE early in the rehabilitation process and

needing any	HCPs helping each other out
rehabilitation needs	
have thoughts that the DPE is a	(perception) of lower value - that the DPE is a less worthy alt
not willing to use digital	
that is easy to use were	ability to use it as a pedagogic tool.
and motivation towards trying	having a positive attitude
uncertainty towards and	uncertainty
patient questions using the	as perceived as positive
complement yet not a	
that enhanced	
with the introductory	content with the training
with the introductory	work manual
participants expressed a wish for	a wish for briefings throughout - not just the beginning of im
that they became accustomed	
learn to use the system	
requested a deeper pedagogic	"we haven’t had anything pedagogic to explain"
that giving patients access to	
easy to use in daily life	comprehensible
system looking old fashioned	sometimes unclear
to the DPE early in the	acknowledged
that is easy to use were	easy to use
digital system should be easy to	it was easy to use/administer
knowledge of	
system looking old fashioned	
system looking old fashioned	old-fashioned
patient questions using the	easy
system looking old fashioned	how to find functions and facts about the patients
have enough staff down here	
need for support and guidance	need for support
need for support and guidance	(extra) Support needed
to receive rapid support when	receive rapid support
towards the DPE were	
towards the DPE were	to be given time for implementation.
routines to be able to get the	routines
without designated time for it	not having designated time for it
have enough staff down here	Didn't have enough staff
with treatment routines	

patients that had utilised the	workflow - less patients contacting the clinic
that enhanced	provided common ground to stand on.
educational programme	
educational programme	SOCIAL
communication when using the	
involved during	
reach out	
reach out	
that enhanced	
changes necessary for	lifestyle changes
appointment, you	
informed by taking	shared understanding
was also that commonly asked	
were that a greater	being more secure in terms of their diagnosis
educational programme	less social interaction
educational programme	
opportunities for the	
another	
of course, so there are a lot of	makes my job easier,
Institutional and cross-sectoral buy-in ("...implementation would need to be a team effort and	
HCPs' concerns about consequen	increasing erors - false-positive readings/measurements
lack of availability/access to devic	lack of availability of electronic communication devices
commitment/support from leade	require clinic leadership support
Institutional and cross-sectoral buy-in ("...implementation would need to be a team effort and	
HCPs' concerns about complete automation of data analysis/clinical decision-making through	
HCPs' concerns about complete a	concerns regarding complete automation
HCPs' concerns about accuracy of	of measurements from DHIs/digital devices;
Institutional and cross-sectoral bu	would need to be a team effort
robust clinical protocols for DHI ("	robust clinical protocols for DHI
dedicated and trained staff to sup	nurses - use of nurses
dedicated and trained staff to sup	e.g., nurses responsible for training patients
nd trained staff to support the implementation of the DHI	
dedicated and trained staff to support the implementation of the DHI ("Each also stressed the	
reduces important face-to face	reduces important face-to face interaction time

	Intention
More than half of respondents reported that they would benefit from a “review of validated d	
	unclear data
from professional and scientific societies regarding the use of digital devices	
legislation clarifying medical liabi	clarifying
e “potential need for confirmatory testing” (41.5%) (	
	for patient
	noisy data
	paramedical support
	too much effort
BP and symptom data together	patient-centered data alongside clinican recorded data
HDPM	compliance with with reporting BP and symptom data
supported the HDPM integration	Hospital
time-consuming technical suppor	the pilot surfaced limitations in the EHR app developer’s inte
positive view of HDPM’s	(clinican) workflow
to integrate HDPM data into the EHR.	
A clinical champion and expertise	clinical champion
amount of	
BP and symptom data together	
amount of	that requires immediate attention
BP and symptom data together	BP and symptom data together in one report
workflows to triage patients so	optimize workflows - to triage patients
workflows to triage patients so	to triage patients
supported the HDPM integration	Hospital IT personnel
stated that	importance of self-care

stated that	
amount of	volume - the amount of data flowing into the EHR
amount of	
support in	
MyBP was felt to increase the	awarenss of the importance
a personal BP monitor was a	personal BP monitor
fashion," which is important	results in timely manner
BP reading or interfacing with	not automated - manually filling in
with HBPM has "… gotta be over	of home blood pressure monitoring
a more reliable source of	more reliable source of information
data	data aggregated
precision while managing	
the cuff	
as a potential barrier to MyBP	
tech-savvy enough."	
practice)	communication about results
teams appreciated the	communication channels
Was she seated at rest for five minutes?	
measurements patients	continuous measurements
regarding implementation,	lack of continuous data
provider continued to explain	low
their treatment	
creating a "stable schedule and a	
also sending them messages to	
afford	
[is] not cutting into my busy	(clinican) workflow
compatibility with the current	
that process to what would	
some decision-making "	
that direct electronic	
"champion" for MyBP would be	
their blood pressure.	
earlier intervention, for	earlier intervention
that symptoms or	
someone has had	
program could lead to input	input errors
error was also a concern as an	measurement technique error

would want to see	
people I think kinda overdo it.	
engaged patient	need to be engaged
capability.	low motivation
advantage for MyBP, reporting	providers could easily imagine using it
was concern that patients	those struggling with self-management
that symptoms or	Concerns
interaction … they want to do	
teams appreciated the	
considered to be a potential	
she is at the “crux of one of our	too many other things going on
will be a group that doesn’t want	disinterest
the program could address	
MyBP’s educational videos assiste	educational videos
patients were motivated but	
important issue facing her	
program could lead to input	
wonder about accuracy overall.	of measurements
a more reliable source of	
error was also a concern as an	can't verify how patients do it
Was she seated at rest for five mi	don't have the context
[is] not cutting into my busy	
complexity was also seen as a	
was easy for patients	easy for patients to use
it seems pretty simply designed.”	
helpful because they can see	averages
program reports gave patients	The program reports
patients have the context to	
a million things to do and so	
expand office staff	office staff
sta—team members	need new team members
personnel to help with	dedicated MyBP personne
a million things to do and so	
relationship	
program reports gave patients	
Other providers felt that MyBP w	setting up a routine
systematic HBPM into	

that MyBP would help patient	
measurements patients	
to measure their BP in a	convenience of their home
somewhere else to get it done”	
categorized or overtreated	white coat- reduced now
potential is there for people to	
circumstances where MyBP	
platform for patients to not only	
the program to quickly	
the program to quickly	
believe that uptake of the fax	
buried in paper	
Devices that measure accurately;	
HCPs can access data anywhere and anytime;	
Poor accuracy of measurements t	
devices that have the ability to measure several parameters (as opposed to one single parame	
individual patient needs	
possibility for the HCP to individualise/personalise the device features to the individual patien	
Change the parameters, so you aren’t going to constantly get alerts you don’t need. S	
older age of patients ("Difficulty in use due to user not being technologically advanced");	
devices that collect data continuously (as opposed to intermittently);	
older age of patients ("Difficulty in use due to user not being technologically advanced");	
connected and it’s got a wire.	
HCPs' concerns that the device/D	wasteful
the DHI includes communication	communication back to the patient
HCPs' concerns that the device/D	It was also reported that hospitals often purchase cheap dev
Delirious patients pulling leads of	patients taking the devices off
HCPs' concerns about patient safety of the device ("Concern was expressed about potential de	
alarms not well-explained "Beeping causing anxiety and provoking panic";	
high wearing comfort of the device;	
heavy and restrictive physical design of the digital device ("In terms of overall device wearabili	
the device/DHI supports data interpretation and recommendation of actions;	
currently record is not real or	
alarms not well-explained "Beeping causing anxiety and provoking panic";	
The virtual care model offers flex	remote areas - live outside of the city area
The virtual care model offers flex	higher attendence/enrollment in the program
Difficulties in providing appropriate care	

Difficulties in providing appropria	appropriate care
Ability to accommodate to patien	vision and hearing difficulties
Ability to accommodate to patien	
Ability to accommodate to patien	adatped based on language preferences
Difficulties in providing appropriate care (e.g.: teaching patients how to check their blood suga	
Difficulties in providing appropria	such as physical contact
difficulties: teaching patients how	difficulty to train patients remotely
through the virtual model with	
The virtual care model offers flexibility, which enhances patient attendance and engagement i	
Difficulties in providing appropria	difficulty in providing trusting relationships
Difficulties in providing appropria	difficulities in the establishment of trusting relationships
Possibility to implement a person	support according to patient need.
Possibility to implement a person	DHT can adapt to patient exericse abilities - not a standard 1
Poor integration of the DHI into t	poor integration into the workflow
HCPs' experience/expertise and s	reassurance from HCP involvement
HCPs' experience/expertise and s	Human factors - need the human aspect
HCPs' experience/expertise and s	Professional experience, knowledge of condition from HCP i
Lack of time	lack of time
Other work commitments;	data other tasks
Presence of social support within	family support use of DHT
The perceived benefit of using AL	and to promote their engagement
Patients' limitations in digital acc	access to phone
Patients' limitations in digital acc	(lack) access computer
Patients' limitations in digital liter	data digital literacy
regarding hypertension and	data health literacy
with MyChart to vary by age,	data old age
issues	data technical issues
Prohibitive Financing/cost of BP r	data competing Financing/costs
Prohibitive Financing/cost of BP monitors ordered in ALTA along with presence and interaction	
In-clinic challenges with HCP's ac	data internet/wifi
patients are not forthcoming	not being truthful of their medication adherence
MyChart offers,	data limited understanding
staff stress and burnout is an	desire to improve staff stress and burnout
patients - staff are eager to	data
hypertension program did not	already existing strong hypertension programs did perceived
Training opportunities for staff fo	No strandardized training
Demands for technical support pc	Responsiblity/liability - new responsibilites of tech support

navigating social and clinical	data
preference for other forms of cor	as opposed to the DHT
Demands for technical support pc	
The perceived benefit of using AL	educate patients of the risks of uncontrolled hypertension
I think that if we have constant cc	communication = motivation
them and keep on track.”	
And they gain more knowledge c	increased knowledge of their condition
monitoring of the main	Automation - less manual documentation
and optimize consultations	
access are able to come through	for those that need it most
allows other avenues because	reassurance
allowed for timely action.	
as well as tools to facilitate medication adherence	
visits to the clinic and optimize	
and optimize consultations	
communicate with different	HCP collaboration
track different activities (eg,	levels of intensity
clincians mentioned the importar	Personalization feedback
a desire for personalized education features (eg, daily guide for diet and liquid intake) accordi	
track different activities (eg,	Can track a range of activites
patients with a stable or long-	patients with a stable or long-time condition
clincians mentioned the importar	Automation
track different activities (eg,	automatic
clincians mentioned the importar	Automation
team for each patient, what the	
overwhelming sometimes. So	
insufficient digital literacy	
clincians mentioned the importar	of personalizing the feedback
participants suggested potential	Clinicans have more information and can actually do someth
the ability to share audio-visual ir	
patients with a stable or long-	stable or long-time condition - referring to age
patients with a stable or long-	
Patients do learn from that interaction [automated feedback]	
lack of remuneration,	
need for connectivity with other	
Then they just lose interest. [...]	
I think that if we have constant cc	communication is a facilitator

exacerbation, when they might	Introducting the app at an appropriate time
or their family member is able to help	
could be helpful.	
learning how to use an app	
in there, how much information	
when you need to be 45”	
"They do get confused [...] We see patients accidently doubling up on doses because of generi	
overwhelming sometimes. So	
overwhelming sometimes. So	
("The app creates a graph that they can see and we can see.	
are part of the team, also taking	
team-based monitoring.	
importance of all clinicians being	
combination of what else is	don't have time to do it
workload,	
monitoring of the main	Automation - less manual documentation
HCPs perceive a clinical benefit of	of evidence
HCPs perceive a clinical benefit of	
Increases the Financing/cost-effe	reducing both scheduled and urgent visits
Increases the Financing/cost-effectiveness of resources by reducing both scheduled and urgen	
HPCs perceive the DHI improves	to medication
HCPs perceive the DHI improves continuity of care and provider-to-provider communication ("	
HCPs perceive the DHI improves continuity of care and provider-to-provider communication ("	
HCPs percveive the DHI improves	"Improves patients’ experience
Lack of accreditation of mobile he	Lack of accreditation
lack of funding ("Lack of guarantee of the long-term economic sustainability of research proje	
lack of funding ("Lack of guarantee of the long-term economic sustainability of research proje	
Poor interoperability/connectedness with other digital systems ("Risk of not sharing the patien	
Poor interoperability/connectedness with other digital systems ("Risk of not sharing the patien	
HCPs percveive the DHI improves	to the two-way health care provider-patient communication
Risk of not protecting confidentia	CONCERNS of not protecting confidential patient data
Risk of creating inequalities in patient care due to resistance to use technology or the digital d	
Lack of institutional guidelines to	Lack of institutional guidelines
HPCs perceive the DHI improves patient self-management ("Improves patients’ knowledge of	
HPCs perceive the DHI improves	education of health disease
HCPs perceive the DHI increases a	traveling difficulties
HCPs perceive the DHI increases a	

HCPs perceive the DHI increases a	
Risk of creating inequalities in patient care due to resistance to use technology or the digital d	
Risk of creating inequalities in patient care due to resistance to use technology or the digital d	
HCPs percveive the DHI improves	
HCPs percveive the DHI improves	"Improves patients’ experience
Increase in workload for staff;	
.all the work that’s done in-betwe	
Improvement in hypertension ma	better ability to manage hypertension
"I feel that sometimes [SMBP] ke	prevents unncessary adjustment of medication
.I ask them to monitor at home ju	data comprehensive
and that SMBP helps diagnose	- because they have more of a realistic measurement of blo
and that SMBP helps diagnose - b	- because they have more of a realistic measurement of blo
accuracy of BP cuffs. Accuracy of	data
Other felt that although incentive	logistic problems of SMBP,
A common barrier perceived by a	
Many felt that older patients have	
most physicians were influenced	data evidence-based
evidence behind [not doing	if evidence showed it was not effective, then they wouldn't u
and not the behavior of their coll	the behaviour of other HCPs would not have a perceived infl
Financing/cost of BP cuffs	data
included obtaining cuffs through	data equipment
The Financing/cost is all on the pa	data equipment
Increasing incentives - financial in	
Common emotions included feeli	data optimism
Physicians indicated that their pa	data optimism
. Patient incentives— now, that’s	
or using pharmacies that offer BP	using pharmacies that offer BP cuffs
Changes to guidelines influence u	guidelines that emphasize use of DHT
Insurance isn’t going to pay for a	data insurance companies
If their insurance pays for the blo	data insurance companies
considering SMBP a part of their duties as a physician served as motivators for some ;	
Nearly all respondents indicated t	hypertension management through SMBP could be assisted
or in some cases performed com	responsibility transferred to a nurse
as they perceived SMBP to fall un	feeling it felt under their job description
assistance from other health care	data sharing
But I think once someone is on-b	
Some PCPs found it concerning	Concerns of others not using : data concerns

However, most felt that with som	
Some physicians asked patients t	not trusting home values
Some physicians asked patients t	not trusting patients ability to accurately measure BP cuff
A few physicians felt that non-ph	feeling that non-physican health workers might not be quali
When patients can use digital too	
Several physicians thought that i	involving different HCPs for interpreting data etc
another barrier commonly identif	lack of workflow support
A common problem was lack of ti	no specific time allocated to review incoming readings
[lack of time to review incoming r	data reviewing
physician stress	stress likely related to increased workload
respondents expressed frustratio	data stress
the overwhelming number of rea	data negative emotions
manage white coat hypertension	data stress
TM as familiar (67%; 4/6)	
Participants found TM easy to un	easy to understand
Having remote readings readily a	Readings readibly accessible
TM provided reliable clinical data	
all the practitioners were able to	greater access to blood pressure readings
ability to access patients virtually	FOR - ability to reach patients at home
This enabled more clinically informed patients visits and made good use of the time allotted fo	
e, providing visibility to the ongoing health status of their complex patients at home regularly	
participants felt that TM would b	monitoring
We could immediately tackle if th	treatment
which the care team deemed to have potentially saved the patient's life:	
ability to track and trend multiple	ability to track and trend multiple readings over time
clinicians reflected on how patien	Perceived impressions of patient improvemetn
however, monitoring patients aft	Espeically for complex patients
TM enabled a higher level of care.	
Participants felt that TM facilitated communication within the model and between individual c	
So that all depends on the readin	To - readings
TM provided reliable clinical data	Reliable data
I think majority of our patients ca	perception from HCPs that it could benefit all patients
Other routine metrics such as ten	e.g., temp added as a feature for COPD patients
A patient was alerting frequently.	
their feedback could improve TM in the future (5/6, 83%).	
Clinicians reported that TM provi	Clinicans could obtain more contextual information of the pa
Professionals considered essentia	more information on the patient's mental health

Several clinicians had previously used a TM system based on health coaching, allowing them t	
[illegible] experience implementing	long experience implementing Telehomecare
Other participants felt that the tr	integrated into clinical workflow - can be incorporated into c
CCC was successfully embedded a	Embedded into normal work
TM can be easily integrated into the exisitng work (4/6,67%)	
In a few instances, participants fo	Overdepending on the clinical team to reach out
However, after reviewing the rea	not answered the symptom questions as intended
One administrator was observed to describe their support for clinician engagement with TM a	
Clinicians frequently reviewed the TM dashboard, discussing TM during in-person patient app	
TM value in their work (5/6, 83%)	
Clinicians seemed to internalize t	perceived need for the DHT
All clinicians felt that the ability t	Feeling it's important
they would support TM in the future (100%)	
Clinicians seemed to internalize t	perceived need for the DHT
Clinicians wondered if their patients would use it and what kinds of benefits might be realized	
Knowledge of the individual patient, their clinical conditions, and their specific clinical context	
"It was similar because we do ask	No added workload - part of their usual responsibilites
Aligning TM-embedded workflows with TM responsibilities facilitated clinician engagement in	
TM alerts aligned well with certai	Alignment with existing responsiblities
By sharing patients on TM, the te	Responsibility/liability - Shared task sharing among HCPs
. The responsibilities associated w	Tasks can be transferred easily - not one person responsible
Having the bulk of TM responsibilities within the RN workflow was initially described as stress	
Integrating TM in the social conte	Help from informal cargivers supported TM monitoring and
Some staff members felt that their complex patients may be apprehensive about TM technolo	
familiar with its broad purpose	undestanding its purpose
information, so it's a little bit mor	Visualization - ability to process the data
Clinicians felt that visual different	Visualization - ability to process the data
("if we could get the system to id	Visualization - alert severity by color
By differentiating the workflow a	Responsiblity/liability - having clear roles
The nature of the TM caused that	Workforce - having additonal workforce support
Having the bulk of TM responsibil	Responsiblity/liability - only one person having the main role
We're able to follow them [the pa	Better monitoring
This frequency of increased clinic	sustained patient-provider communications
For the pharmacist, preexisting routines involved calling the patients weekly to obtain reading	
participants felt that TM would b	no additonal appts
TM facilitated sharing of clinical v	reduced clinical workload
which helped with the prep work	Less prep work for composing clincial history

Engagement with TM relied on cl	Required time investment to learn how to use it
for incorporating symptom	no standardized process for incorporating symptom informa
efficient	symptom reporting
of the treatment you want to	as soon as possible to help prevent further harm.
savvy are still willing to use	even those with low technical skills
similar goal could be	concerning patient symptom data trigger an alert in the
similar goal could be	concerning patient symptom data trigger an alert in the
similar goal could be	
patients should be able to	to report symptoms.
savvy are still willing to use	even those with low technical skills
oncology, cardiology, and	
to get that information? I think	
this by describing a	
for incorporating symptom	for incorporating symptom information into the EHR,
institution requires that data	
infrastructure to	
app	
ensure adoption, the app should	low interaction
ensure adoption, the app should	
ensure adoption, the app should	
like if you had them	too much inputing
based model rather	
process for providers or staff to	
oncology, cardiology, and	care should all be involved
based model rather	
based model rather	
institution requires that data	the institution requires that data from external apps be revi
be motivated to use the app if	faster way to communicate with their provider
suggested that the app could	
patients	be motivated to use the app if they understood that it was
be motivated to use the app if	faster way to communicate with their provider
be motivated to use the app if	faster way to communicate with their provider
the dynamics of remote	
reported that remote	
of	themselves
patients or conduct physical	missing important signs
patients or conduct physical	fear of misdiagnosis

patients or conduct physical	
the dynamics of remote	appropriate for stable ones
for initial	sensitive topics - breaking bad news
of uncertainty and	experience, conducting remote consultations became
longer to question	no visual cues
somebody looks well	
somebody looks well	or unwell when they walk in... Whereas obviously I
they tried to use their own body	
patients or conduct physical	
patients or conduct physical	
of uncertainty and	consultations
dynamic could work well	having an established relationship
tried to create space for	intentionally asking questions related to concerns etc
for initial	
cases	confidence in their ability to assess remotely
cases	some doubt in their ability to assess remotely
cases	some doubt in their ability to assess remotely
we do relate to people	
do not raise all their concerns	feeling patients don't raise all their concerns
cases	
meant that clinicians	
dynamic could work well	
do not raise all their concerns	less sharing of information
longer to question	easy
remote consultations also	harder to gauge if the patient understood
myself quite a lot to check	keep having to ask if the patient understands
more questions in	lack of natural pauses
of uncertainty and	makes it more draining for HCPs
of uncertainty and	
were thought to offer	
were thought to offer	
were thought to offer	convenience of care
were thought to offer	safe money on travel Financing/costs
were thought to offer	helpful for working patients
reported that remote	more focused,
reported that remote	
patients or conduct physical	fear of misdiagnosis

they tried to use their own body	they didn't feel as reassured
clinician can be	feeling less well cared for and uncertain of
longer to question	patients
of uncertainty and	makes it more draining for HCPs
Multiple hypertension guidelines	Physicians autonomy - individualize BP goals and medicatio
Multiple hypertension guidelines	
Multiple hypertension guidelines	
Need for a large BP cuff	adjusting size needed
participate, we provided	virtual hypertension
pharmacist training to alleviate	
pharmacist training to alleviate	
Physicians wanted to be informed	prompt calling after patients were randomized
Cumbersome vCCC authorization	
Patients will need to be motivated to participate	
relinquishing their duties to the	didn't want to give up their duties
relinquishing their duties to the	HCP expectations!
hypertension and assisted	
hypertension and assisted	
hypertension and assisted	education
physicians	
to be informed of the	nurses and clinic staff
relinquishing their duties to the	
Cumbersome vCCC authorization	
Re-designed pharmacist note tem	of reporting template - improved reporting template
accept new referrals in a	streamlined intake process
Incorporated illustrations of daily	of daily monitoring report, the traffic light
management,	
accept new referrals in a	monitoring workload
accept new referrals in a	
Pharmacists perceived daily moni	
Pharmacists perceived daily moni	
Perception of better integration ("	'Well, I already do home BP (blood pressure) monitoring wit
Increases patients' awareness of	hypertension
Increases patients' awareness of	
Facilitates patient independence ("	'Usually weekly, because of the way I'm recruiting them in
With respect to patients, HCPs pe	
Perception that patients are treat	

Perception that patients are treat	
Perception that patients are treat	
With respect to patients, HCPs pe	
Lack of time ("‘I don’t have time t	don't see a clinical benefit
implementation was an evolving	
GP champions for telehealth play	that clinics were supporting it's use
Automatic workflow ("‘Well, I don’t go looking at them,[Docman reports] they come to me au	
For some finding appropriate staf	a lack of IT skills
Efficiency and storage of data cor	unclear about how to store data
Efficiency and storage of data cor	unclear about how to store data
Technical problems were quickly	technical problems were fixed quickyl
Perception of better integration (	good tech - works well
Automatic workflow ("‘Well, I dor	workflow
HCPs liked the summary BP repor	integrated within the exisitng result managing systems
GP champions for telehealth play	
For some finding appropriate staf	finding staff to lead was a problem
GP champions for telehealth play	that clinics were supporting it's use
They also reported a sense that p	
Lack of time ("‘I don’t have time t	for the HCP
Perception that it is well thought out ("‘I think it seems to be well thought out. The protocols a	
(observation notes) GPs were not happy with email communication as there was no natural cl	
People want it ("and people like i	somebody who was offered it and then clinically it wasn’t q
Becoming mainstream ("‘that’s pr	
Need to spread out the work (‘Dr XX took on board some of it	
Becoming mainstream ("‘that’s pr	Mainstream now
People want it ("and people like i	they want it and like it
Perception it will be well adopted	
They also reported a sense that p	
They also reported a sense that p	
Practice staff valued the clear pro	
The preferred approach for contacting patients using Scale-	telelphone calls were preferred
Perception that IT is complex ("‘I	complex
Perception that the initial registra	complex
HCPs liked the summary BP repor	liked the summary BP report (via Docman
HCPs liked the summary BP repor	clear - clear prompts
Perception that it is well thought	
implementation was an evolving	who they would use the DHT on

(‘Dr XX took on board some of it	
Practice staff valued the clear pro	
Practice staff valued the clear pro	clear protocals
(obersvation notes) some practice	on who to contact
(obersvation notes) some practice	allocating time
(obersvation notes) some practices allocated time for triaging telemonitoring data (to a nurse	
(obersvation notes) some practice	
and that telehealth increases wor	
(‘Dr XX took on board some of it	
Time for setting up systems for id	
Time for setting up systems for id	
For some finding appropriate staf	finding staff to lead was a problem
(observation notes) Most practice	adding in extra time to deal with DHI tasks
Lack of time ("‘I don’t have time t	
Perception that the initial registra	data entry
Perception that the initial registra	initial registration
Increases patients' awareness of	
and that telehealth increases wor	
too much workload ("‘They’re [th	
(observation notes) GPs were not happy with email communication as there was no natural cl	
Facilitating shared decision-makir	empowering patients
Facilitating shared decision-makir	Shared decision making - More informed and aware of the o
making patients more informed a	More informed and aware of the options
clinic consultations and	consultations
clinic consultations and	
If they’ve got these things to disc	consulation
the personal summary	
that good explanation of what the disease is and the disease process. It gives people a bit of cl	
maybe and I like the score	patients would be more honest - increased accuracy of how
maybe and I like the score	patients would be more honest
might be concerned and I might t	of care
might be concerned and I might t	of care
purposes, because if it is and if lots of patients are saying I’m unsure about something, then it	
Desire for different languages	language
Yeah, so I think what you’re sayin	exporting this output
options: health professional	Need for more treatment options
The opportunity to complete valu	ability to fill in concerns and open-ended statements

Desire for increased font size	Font to small for older patients
Two health professionals questio	FOR- lower eduation levels
would find the information	FOR- English as a second language
know, somehow send it in such a way that it’s just attached to the electronic patient record or	
information/usability changes	Misidentification
agreed that patients don't want	
are not optional ("Cardiologist 8:	
("Cardiologist 4: “Well I certainly	misinformation
controversial statement when	misinformation and broad claims
('Cardiologist 1: “That’s just wron	misinformation
Layout of the display ("Nurse 10:	Ease to use / simplicity
Benefit of simplified and straightf	Simple design
Cardiologist 1: “Yeah the only thi	Unfamiliarity
Layout of the display ("Nurse 10:	pictures to convey meaning
Good contrasts ("Cardiologist 1: “	Contrast
use of visual cues ("Nurse 11: “Nu	pictures to convey meaning
Desire for increased font size	Font to small for older patients
some medical terminology that	langauge too complex
start to make some doctors think	Quality and safety in practice
facilitates understanding patient	Increased information about other factors happening in pati
facilitates understanding patient	Increased information about other factors happening in pati
is to cut down on the time of the	
Optimizing alert transmissions thi	standardized (alert) protocols
50% Satisfaction with current patient-to-staff ratio	
alerts without the patient being	*Patient has to be present to change alert: go to care*
alerts without the patient being	*cannot change alerts easily without patients present*
transmissions resulting from	*nontailied alerts are a barrier*
issues such as dealing with noncli	*over-alerting*
knowledge regarding electrocardiography	
Loss of connectivity to remote mo	
88% perceived connectivity was a	connectivity to remote monitors was identified as a major
88% perceived connectivity was a	connectivity to remote monitors was identified as a major
50% Knowledgeable of wearable health devices:	
administrative/financial issues	
The Device Clinic is not viewed as	*profitable : not seen as profitable*
the number of unbillable tasks	
areas	

27% perception that their clinic had the technology to process wearable health data;	
33% of clinics allowed for the inte	LACK of integration
come into the office for in-clinic	
Lack of patient buy-in	*lack of*
International (HL7) into the	*standards for the transfer of clinical and administrative data*
billing	
the country for alert	*alert management standardization*
issuing a remote monitor at time	issuing a remote monitor at time of implant
issuing a remote monitor at time	issuing a remote monitor at time of implant
burnout	
50% satisifcation with the degree of education that patients, staff and physicians received fror	
50% satisifcation with the degree of education that patients, staff and physicians received fror	
Optimizing alert transmissions -m	Training on how to use the monitor
with “Robust, long, on-the-job	
lack of keeping staff trained	*Lack of training*
Barriers of staff training	
lengthy process of training	
be an issue	
increasing remote staffing	workforce
having a full-time remote team working from home	
technician,	HIRING workforce- specalised person
person to check insurance and	HIRING workforce- dedicated office person
with third-party platforms that	to work on the interface - deal with the technical side of thir
International (HL7) into the	*standards for the transfer of clinical and administrative data*
and handling lack of uniformity w	*lack of uniformity with how to handle alters*
such as lack of CIED-trained staff	*Workforce*
lack of staff in general	*Workforce*
The low supply of knowledgeable	*low Workforce*
difficulty in showing productivity, lack of benchmarks, budget, and workflow	
50 % perception that their clinic was adequately staffed to handle a large volume of remote r	
41% satisifcation with the present workflow for responds to alerts , patient follow-ups and pro	
44% satisification with how their clinic processed alert transmissions after hours	
troubleshooting home monitors	
timely matter	
transmissions was identified as a	*volume of data’*
hours	*outside clinic hours*
“I think majority of nurses enjoy h	for nurses

because it gave them an opportu	for nurses
psychological reward (for staff) for helping patients and utilising prevention in a healthcare se	
“If the check-up takes longer, tha	fewer patients
“If the check-up takes longer, tha	fewer patients
If fewer patients are seen, obviou	
Most participants (7 out of 11 pa	
The most common advantage wa	more regular access
the shortage of dentists, a long w	currenlty long wait time for patients
Some staff thought that targeting	could be simplified to age only
When discussing possible models	advertisement could be made online, with leaflets in the pra
that software should be able to c	flag for at risk patients
Patients that are screened positiv	automated referral
that software should be able to c	flag for at risk patients
nd the amount of administrative	clinician leading - non-clinicians felt the responsibility of a re
Some staff thought that targeting	anyone who wants to get it can
Remuneration (lack of) was also d	
Most participants felt that screen	guidelines for ensuring equitable care
Most participants felt that screen	t screening should be free to NHS patients
Remuneration (lack of) was also d	
There were concerns from clinicia	workflow - dental appt
All participants thought that patie	workflow - dental appt
but it would not have a priority b	not a priority
Participants felt that patients and	
Three males and two females we	
but felt having a spare surgery wa	CONCERNS
Some suggested doing the screen	during lunch or at best time for clinic
included being comfortable with	they share similar tasks
Three females were positive abou	
Three females were positive abou	
I think especially knowing the info	about number of a fib
Participants received information	
Participants felt training was essential, it should be led by experts, hands-on, and during worki	
Participants felt training was esse	should be hands-on
Participants felt training was esse	during work hours
“I think it'd have to be hands on.	hands-on
Participants felt that patients and	but they might feel sceptical about dentists screening for a
“I think it's a brilliant idea. I think	

was simple, quick and the device	simple, quick and easy to clean
was simple, quick and the device	simple, quick and easy to clean
and that there would be resistanc	would be a barrier if it effects these thigns
and that there would be resistanc	would be a barrier if it effects these thigns
I feel like we can be, especially no	currently very busy
the shortage of dentists, a long w	shortage of dentists
Participants were keen however,	having clear roles
Clinicians felt dental nurses or tre	dental nurses or treatment co-ordinators (in private practice
I feel like we can be, especially no	currently very busy
psychological reward (for staff) fo	reward for helping people
Participants felt that patients and	
“I think it's a brilliant idea. I think	not too much work to implement it
were regarding the increased workload,	
nd the amount of administrative	admin time
nd the amount of administrative	admin time
prioritized factors for CV risk	
reminded the provider about patient-specific CVD risks	
commit to taking a statin	
my patients	
when the provider saw the	DHT printout not ready in time
Data is not current	not current data
If it actually worked	
my patients	
reports for	
uncontrolled CVD risk factors in	
uncontrolled CVD risk factors in	
50%)	
I usually ignore pop-ups	
available.	
I don’t find it useful/helpful	not finding it useful
reported that they got CVD risk	no perceived need
CV Wizard-	
end up reviewing it on my own	
Patients like it	
I don’t find it useful/helpful	not finding it useful
provided a useful visual aid for patient education (23/32, 72%),	

My staff is not trained	staff is not trained
Wizard in	
understanding of CV	having a better understanding of the tool
better understanding of CV	other staff
the provider	
when the provider saw the	DHT printout not ready in time
Making CV Wizard interactive	
provided a useful visual aid for patient education (23/32, 72%),	
provided a useful visual aid for patient education (23/32, 72%),	
I don’t get the printouts	readibilty - not understanding prinouts
not enough time during the visit	
me	lack of sharing - only shows up for certain HCPs
barrier: time in the workflow	
Revised workflows	revising workflow
Mas	
not enough time during the visit	
seems to take a lot of time to	
controlled BP	data health outcomes
increased knowledge about hype	data health literacy
reduced emergency department	data health outcomes
greater medication adherence	data adherence
appointment attendance	data retention
ability to create more tailored	presonalization
increased use of self-managemen	data self-management
	not having a smartphone or digital resources in general
	not having a smartphone or digital resources in general
Another staff member shared that larger cuff attachments	data size
Patient education on hypertensi	found a link between knowledge and self-efficacy
Patients’ lack of digital resources	data digital literacy
patients who are mentally	patients who are mentally or physically unsound.
high level of social needs	variety of patients with different social needs - financial, lite
problems pairing the BP devices	connectivity
problems pairing the BP devices	connectivity
as significant facilitators. FQHCs	prior experience of being apart of other hypertension grant
Giving patients a free BP cuff	provding free equipment to patients
Limited grant funding resulted in	limite grant funding
Insufficient pay motivated staff turnover	data staff compenstation

Technology-related barriers (e.g.,	data internet/wifi
At FQHC 1 and 2, staff shared having to manually transfer	data ehr
many patients had a cellphone	data pairing
Program champions and significa	program champions
Program champions and significa	Mentorship
"A common problem with me [is] when a patient upgrades	thinking device automatically sends over data
Both FQHC 2 and 3 acknowledged their inability to	not able to bill insurance
social support from community h	from community health workers motivated program engage
Patient education on hypertensio	educating patients on hypertension
Program champions and significa	significant training opportunities
increased trust in the healthcare	data health system
Limited workforce capacity	workforce
FQHC 2 shared hiring a program manager	Hiring specalised role - project manager
remote monitoring company,	data third-party
and their heavy workload make b	
, and alleviation of mental burden	data burden
	Behavioral intention of use (73%)
	easier to communicate
	time efficiency
	if other HCPs use it, then more motivated
	perceived usefulness (82%)
	social influence (86%)
	perceived ease of use (77%)
Perceived advantage of increasing	
HCPs appreciated that the compu	data ease
patient follow-up and	data retention
Perceived advantage that the too	data evaluation
perceived advantage of interdisci	complements current health care services
The limite ability of HCPs (to guid	data digital literacy
The limited ability of both the tar	data digital literacy
Pedometers are designed only fo	patients who cannot walk might be disadvantaged
Financing/cost of technologies	data

Financing/cost of technologies ar	data equipment
Difficulty in accessing informatior	difficulty accessing info in a secure web platform
A web application and easy-to-us	with services already in place
lack of patients' willingness to pu	data willingness
Concerns over confidentiality and	CONCERNS - confidentiality
Concerns over confidentiality and	CONCERNS - security in terms of online transmissions of dat
Concerns about protecting confid	CONCERNS - confidentiality
; Lack of interest on the part of sc	data interest
The pedometer offers patients a s	simple and accessible measurment and not complicated
interdisciplinary communications	complements current health care services
Provides a sense of security for pa	data safety
Lack of time required for training	
All members praised their orientation; all benefited from 1-on-1 approach to observing and th	
All three cardiologists and two of three GPs stated they would be interested in viewing a patie	
The main advantage described was having an accurate long-term BP (blood pressure) record	
. One cardiologist praised the ability to detect asymptomatic atrial fibrillation, which resulted i	
Troubleshooting technology thing	FOR - elderly
Troubleshooting technology things over the phone	
Troubleshooting technology thing	new roles providing technical support
All three cardiologists preferred a report based option, generated monthly, in either an electro	
Difficulty in identifying the operat	Different operating systems - different smartphones
I rarely used the manuel	
All three cardiologists and two of three GPs felt TCC (teleclinical care) was likely to be a useful	
Different views on responsibility<br>on managing alerts, GP1 was	*responsibility/liability - different perceptions on who should*
Of the three cardiologists, the res	*responsibility/liability - different perceptions on who should*
Medicolegal liability, two of the th	*responsibility/liability*
GPs may not be comfortable with	*uncomfortable*
Educating the patient on using th	Workload training- time consuming
All members praised their orientation; all benefited from 1-on-1 approach to observing and th	
as well as being able to use the peripheral devices themselves, to understand the complete pi	
One team member commented on the importance of hearing a formal presentation about TC	
felt that automatic sphygmomano	
user friendly manual	
App installation	
All three cardiologists preferred a report based option, generated monthly, in either an electro	
The others preferred to contact t	Having someone to contact for assitance or troubleshooting
Challenges of completing the enrolment were generally related to time constraints in the busy	
Challenges of completing the enrolment were generally related to time constraints in the busy	

There are things that need to be d	that can be done by an adjunct staff member
I do think enrolment needs to be	Workforce - sharing the responsibility
App installation and device pairin	App installation and device pairing
App installation and device pairin	App installation and device pairing
“Do I want to be called about eve	*they only wanted to be called for big changes*
Time constraints were the major	*Time*
concerns about the volume of ale	volume
Troubleshooting technology thing	FOR - elderly
. It would probably take 10–15 h per week to do a proper job of it.”	
Burden of receiving alerts of TCC,	*volume of alerts*
Burden of receiving alerts of TCC, two out of the three cardiologists expressed concerns about	
questions from family members	
When there are only 15-min slots	*Less time and less inclined to look t additional info*
Participants agreed that smart homes could potentially play a critical role in HF self-manageme	
Patient education is fundamental to enable successful self-management. However, people wit	
The system would enable contact with and monitoring by their healthcare team, which could l	
The system would enable contact with and monitoring by their healthcare team, which could l	
Participants also indicated that the smart home system could reinforce self-management goal	
Sometimes, patients struggle<br>(…), if they need something, if	nd help meet patient demands, which can be a major obstac
Participants agreed that smart homes could potentially play a critical role in HF self-manageme	
identify issues early and prevent r	prevent re-hospitalisation
Participants mentioned that even identifying simple physical changes using the smart home co	
Participants agreed that receivin	more objective measure
But if it can be picked up with the smart home system, it can be picked up early stage, which c	
The system would enable contact with and monitoring by their healthcare team, which could l	
The patient might be thinking, okay, this shortness of breath is (…) probably usual for them, no	
The timely response of the system to identify issues would be highly valuable	
You might get a false reading,	
with suggestions for adding other	language FOR - suggestions for adding other languages
In addition, having an intelligent h	FOR - disabled : having an accommodating environment
monitoring and adjusting medicat	abilty to adjust medication needs remotely
In addition, a feature to monitor p	so it works with patients with implantable devices
Having alerts to notify people with HF of the targets and providing the range of normal values	
Having alerts to notify people with HF of the targets and providing the range of normal values	
Having alerts for providers could allow for more timely responses	
it might be [that] people are not s	awareness of technology
Low levels of literacy are also con	lower levels of literacy

wanted to see	centralization
Participants expressed they want	among HCPs
There was an overall consensus t	as primary concern/focus
Participants highlighted that mos	FOR - older people
Participants highlighted that mos	comorbidites
particularly language barriers	language FOR - people with language barriers
might be technical issues/errors	
reminders, and the ability of the	medication reminders
The final factor influencing the u	data Financing/cost/funding/Financing/cost
More importantly, they were con	CONCERNS Financing/cost
and definitely, there will be (…) in	internet
The participants,, could see the potential of smart homes that could integrate a range of teleh	
smart home system was viewed	into the smart home system
They suggested that the system b	Integrated into exisiting healthcare systems
smart home system was viewed	Communicaiton
Participants agreed that if smart	need help from insurance companies to cover Financing/cos
In addition, the security of the pa	CONCERNS - data privacy
In addition, the security of the pa	CONCERNS - data confidentiality
In addition, the security of the pa	CONCERNS - data confidentiality
Varying cultural backgrounds	data socioculturall values
Participants discussed that it would be necessary to provide support to set up and use the tec	
Participants discussed that it would be necessary to provide support to set up and use the tec	
Reliability of technology (("“You might get a false reading, might be technical issues)	
Participants highlighted the need	simplifying
Participants highlighted the need	simplifying
could be central to facilitating	use of nurses
good for the GP to have a phone	technical support
and notifications to the pharmacy	use of pharmacists
The system would enable contact	reduce anxiety
Participants also discussed that the centralisation of data could support the role of healthcare	
Participants also discussed that the centralisation of data could support the role of healthcare	
More importantly, they were con	time
Perception that patients bear the	patients are responsible for collecting their data properly
Perception that daily home meas	data comprehensive
Some HCPs argued that the digita	feeling that they could achieve a normal blood pressure
They also argued that digital data	data is objective i.e., more reliable and not influenced by su
Similarly, some HCPs mention tha	more objective data

Similarly, some HCPs mention tha	standardized clinical decision-making
standardization of the measurem	
perception that the data is not su	data is not sufficient enough to make clinical follow-up decis
A few HCPs argued that in case th	
Lack of contextual information	
Some HCPs argued that confidenc	more work experience meant higher confidence in their inte
This includes the need for inform	need for integration of information found in the EPR (electro
Some HCPs mentioned they often	having to remind patients to send their data
(""In my experiences, some	feeling it is the patient's responsibility to collect their data
(""In my experiences, some	
A few argued that it is also the pa	patients should contact the HCP in case of health changes or
Perception that HCPs should keep	
HCPs (""You have the responsibili	less ability to verify health data among collegues
So yes, it depends on my actions	the one HCP is fully responsible for the patient
Two nurses argued that that intro	Nurses feeling that they had to take on a physicians role and
Whereas in the clinic, you know,	reduced sharing responsibilites
HCPS trusted the data from the d	data accuracy
Some HCPs mentioned they often	not trusting patients to send data correctly
Other HCPs argued that no specia	BP measurement considered hard data 'objective' or speakin
A few HCPs argued that in case th	
Others, who consider the digital t	perception that work experience should have no effect on in
. And then in hindsight, you think	becoming more careful , worried about making a mistakes/c
[also reduces white coat hyperten	not high HP because of white coat experience
reduce the number of visits (64.5	reduce number of visits
reduce the number of exacerbations/complications (48.4% agreed, 33.3% disagreed)	
reduce the number of hospitalizatons (46.2% agreed, 31.2% disagreed)	
increase adherence to treatment improve (58.1% agreed, 32.3% disagreed)	
achievement of target BP values (51.6% agreed, 34.4% disagreed)	
older age (32.3% advisable, 65.6%	
working patients (58.1% advisabl	on the other hand, non-working is seen as a barrier
patients with severe forms of dis	high severity of disease
patients at high risk of disease (39.8% advisable, 58.1% not advisable)	
patients receiving drug treatment (57.0% advisable, 40.9% not advisable)	
“Is it possible to provide remote monitoring and consultation of patients with hypertension? t	

“Is it possible to provide remote monitoring and consultation of patients with hypertension? t	
	difficulities in training patients
	interpretation of data
Lack of time for remote consultation (77% on average)	
More than 2/3 respondents used in their daily lives such digital means of communication	
	less unnecessary hosptial visitsa
centralized telemedicine system	
	they couldn't see the usefulness
More than 2/3 respondents used	internet access from a home computer (83.3%) or mobile de
	severity
	A 'portrait' of a patient would be: it is a patient of predomir
technical complications (48.2%)	
	Lack of
cost of equipment (financial) (43.1%)	
	not ready to use
difficulties with the education of patients (46.8%)	
lack of confidence in the reliability of data collection and transmission of data (48.6%)	
lack of confidence in the reliability of data collection and transmission of data (48.6%)	
transmission of data	
difficulties with the interpretation of results (26.5%)	
Remote care is one of most impo	monitored more frequently
Remote care is one of most impo	
Remote care is one of most impo	
mHealth facilitated with on-dema	
mHealth facilitated with on-dema	

Healthcare professionals mentior	
(managers) Care was no longer ti	
Remote care is one of most impo	
Remote care is one of most impo	
mHealth facilitated with on-dema	
and software were	
They also mentioned that for mH	I can imagine that an unstable heart failure patient will be m
They also mentioned that for mH	patient selection - use for some patients but not all - the HCI
generated	and secruity
(managers) Many participants cau	
They also mentioned that for mH	higher disease severity means more motivated to use
(managers) Current lack of an ade	lack of adeqduate business model
; It would initially result to a loss	loss of income
(managers)Finally, it was mentior	HCPs are required to invest in the implementation
(managers)Finally, it was mentioned by managers (n=3, 60%) that healthcare professionals, wl	
Reduction in healthcare Financing	
Lack of integration into daily prac	increase in clinic Financing/costs
(managers) Current lack of an ade	lack of a business model
Lack of integration into daily prac	daily practice
Healthcare professionals (n=6, 75%) also stressed the relevance of adequate instructions for p	
They also mentioned that for mHealth to be effective, adequate patient motivation and patien	
(managers)Finally, it was mentior	loss of money at first
(managers) Current lack of an ade	
(managers) Current lack of an ade	he value of mHealth programs has yet to be proven for the g
(managers) Managers mentionec	
(managers)Finally, it was mentior	of HCP staff for their time or for at-home care vs in-person c
(managers)Finally, it was mentior	complexity of regulations
(managers) Many participants cau	, you need adequate software with the right software devel
Healthcare professionals (n=6, 75%) also stressed the relevance of adequate instructions for p	
(managers) Need for it to replace	needs to replace (not an addition!)
Lack of integration into daily prac	should replace part of usual care
Healthcare professionals (n=6, 75	adequate instructions
Many participants argued that devices and software of adequate quality are already available	
Mentioned that all patient-genera	
Reduction in healthcare Financing	research nurse
Mentioned that all patient-genera	
Perception that workload would r	because you still need a team that assesses all the generatec

Lack of data	Lack of
need clinical exam	
barrier to initation of therapy (5.6%)	
information deficit (10%)	
Patients may feel bored, turn off	patients might feel bored
Perceived potential of solving the	adherence
Perceived potential of solving …..	
Following-up with patients using	mix of means to communicate with patient
and other technical issues that m	data technical issues
Need for surveying patients and c	data pilot study
Desire for more testing of its effe	need/desire for RCT to test effectiveness
Poor telecommunications covera	in remote areas
lack of electronic health system t	
Desire for better reimbursement	data reimbursement
Ethical concerns (e.g. how to prot	CONCERNS- protect patients' privacy
Perception that phone visits hold	liking of phone visits
developing text message	limiting the amount of messages to the patient
Perceived potential of solving …..	in taking medications
managing alerts integrated	existing clinic workflow.
small number of alerts were	
alerts occurred whenever there	
that the regularity of alerts	
with a phone call, but only made	
responsible for their health,	
interventions, would	don't understand their own illness
alerts were in the EMR and	the alerts were in the EMR
alerts were in the EMR and	Existing processes
attention to alerts at “off” times	not paying attention to alerts
shuffle of clinic flow, “One is the	not checking the alerst all the time
shuffle of clinic flow, “One is the	not checking the alerst all the time
not answer when	not answering
that some patients may	how adherent they are
barrier to success in	and sodium restrictions
interventions, would	patients don't feel responsible for their own health
that the regularity of alerts	locating where the alerts are coming from
were hard to analyze in a	analyzing data
alerts occurred whenever there	not meaningful

alerts occurred whenever there	making them unreliable in identifying actual problems.
shuffle of clinic flow, “One is the	busyness of the workflow
varied widely because	different criteria for responding
shuffle of clinic flow, “One is the	
intervention	
4.34 (indicating that supporting factors were colleges in the pharmacies that were very encou	
healthcare providers (question	
Time that pharmacists could sper	having time
thought that the	
4.34 (indicating that supporting fa	
4.34 (indicating that supporting fa	chief pharmacists
long risk-assessment	
Staff enjoyed the team dynamic A	increased collaboration between the nurse and medication r
Staff acknowledged that ACDC (A	
HCPs perceiving the benefit of DHI for increasing access to services (e.g., in rural areas):	
Other participants described telehealth as the result of ‘token funding’ to negate the need to	
("Although some participants exp	
consumer/patient//clinician invol	
consumer/patient//clinician invol	
consumer/patient//clinician involvement in the design of healthcare services (including DHI);	
collaboration of all stakeholders in the development/design of a DHI ("Participants highlighted	
the DHI does not suit the local	I think getting people on board, who actually live in the area
unreliable internet and phone ser	internet
unreliable internet and phone ser	network
("Although some participants exp	expressed concern,
HCPs' concerns that DHI is a lower-quality substitute for face-to-face services ("Health profess	
the DHI does not suit the local	that the DHI does not suit the local needs
Other participants described telehealth as the result of ‘token funding’ to negate the need to	
Other participants described telehealth as the result of ‘token funding’ to negate the need to	
HCPs' concerns that DHI is a lower-quality substitute for face-to-face services ("Health profess	
collaboration of all stakeholders in the development/design of a DHI ("Participants highlighted	
limitations in terms of physical ex	data clinical testing
deafness of patient	deafness
technical inabilities	data
lack of devices for video-consulta	data equipment
GPs and nurses from the general	the ease of using
The GPs’ assessment was that the	the patient benefited

The GPs experienced that the patients had an easier course and that they were happy to avoic	
The GPs experienced that the pat	
The GPs experienced that the pat	
positivie experience 'positive abo	
The GPs experienced improved w	work efficiency
The GPs experienced improved w	eliminating work hours
positive experience collaborating with other experts: The GPs experienced understandable an:	
The GPs experienced improved work efficiency through communications with cardiologists, th	
The physically or mentally weake:	physically ill or mentally unstable might not adhere to the in
Only a few times, the GPs experie	few technical problems
The GPs experienced improved w	saving money - think it's a huge financial saving in reality
In addition, it made sense for the	as long as we are able to get professional supervision with a
positivie experience 'positive abo	
positivie experience 'positive abo	It is a feature we have been missing,
they think it's easier because the	HCP and clinic
The perception from the GPs was	collaboration
The GPs experienced that the pat	collaboration
they think it's easier because the	HCP and clinic
In addition, it made sense for the	primary care - means better/easier access to care
The perception from the GPs was	confidence in patient's use of the holster
The GPs experienced that the pat	very confident in what we can offer them
The GPs experienced improved w	
primary barrier.	
telehealth decreased the	no reduction in quality of care
minimizing	
the quality of care], and, in fact,	
follow-up	
perception that	"lost in translation" over the telephone.
also described patients not	for telehealth visit
patients that are video equipped	video equipped in their homes
that no matter how much you	don't have email
trouble, obviously, you know,	trouble I think, you know, there's really no role for e-
don't really think it's, in some	not appropriate for HF of limited
impact because our, our folks	Adjusting calls for high-risk patients - more targeted follow-u
that no matter how much you	
that no matter how much you	they just don't get how to like do video appointments.

patients	hearing
patients	eyesight
beginning just because the VA	glitches
described that VA furnished	
minimizing	a lack of laboratory work or physical examinationswas seen a
for patients who need more	not having lab tests
described that VA furnished	providing equipment: (computer, iPad, or monitoring equipm
beginning just because the VA	lack of systems in place
approved telehealth quickly.	approved telehealth quickly.
think the institution at, at, as a	litte guidance from top-down
think the institution at, at, as a	poor leadership
perception that	"lost in translation" over the telephone.
the number of	
the outpatient clinics. Rather	outpatient clinic
that no matter how much you	they don't have family to help them
patients were reluctant to use	
learning curve	when adapting
see. I've found sometimes that	to read and give medical dosages correctly
that no matter how much you	
described that newer	
the stethoscope on the patient's	not easy to get a good reading
honest, that technology, it just,	
honest, that technology, it just,	
approved telehealth quickly.	approved telehealth quickly.
that despite rapid adoption,	need for clearer guidelines
think the institution at, at, as a	the utilization of, of telephones
because I, like I said, I'm one	
because I, like I said, I'm one	
beginning just because the VA	trying to understand how it works
Does ICT use favor communicatio	communication between the different points of
communication with the patient	
communication with the patient	
Another theme of the focus grou	
Information is not sufficient - Alth	
Nurses also made suggestions to	using voice-overs and/or other features
suggestion was made to have link	option to have more content if patients needed it
was making scenarios reality-	necessity of incorporating real-world context and challenges

.” Nurses advocated that scenario	
. Nurses acknowledged the challe	
Clinicians raised the challenge in	example from text: their quality of life might be to eat a hot
There was also uncertainty	data digital literacy
Challenges of differences in patie	education differences among patients was challenging
("I'm just thinking of some of our	data old age
Nurses also made suggestions to	
Participants were uncertain that	
As one clinician queried: “[Our] p	data old age patients
Initially, when using the Web site	data reward system concerns
One participant said, “I really like	
Overall, participants were suppor	data supportive
Specifically, nurses were concern	unclear explanations of what the purpose of the reward syst
One suggestion to address this co	pirmer document for patients before they use the websit
concern	
. Or even greater concern to parti	due to reward system techniques
providers indicated that access to	TO - patient historic information
providers indicated that access to	benefiting NCD control
providers indicated that access to	retention
it standardized patient informatio	it standardized patient information
where patients with NCD often ha	not well adapt for patients with multiconditions
These were intended to reduce d	couldn't add abnormal findings - patients with abnormal dat
was the need for an updated	lack of adaptability - need to update list based on medicatio
eliminated data loss and allowed	less data loss
eliminated data loss and allowed	secure storing of information
format required their information	Increase time requirement to enter patient data twice
standardizing patient information	easier consultations
The availiablity of phones: Partici	nearly all patients have access to phones
Overall low literacy levels in the	(barrier) low literacy skills to use text messaging
[pervasive socioeconomic challen	lack of sustainable funding sources
poor staff compensation	(barrier)poor staff compensation
although the hospital has institut	gap in communicaiton between records that aren't digitalize
serves as a regional referral	seting is referral centre - ideal to implement innovation to h
although the hospital has institut	signal outage
contradictory religous beliefs to j	(barrier) religious views against medical advice
Secondly there is so much equipn	lack of instruments and drugs
inadequate equipment in the	lack of diagnositc equipments

Participants lamented on working	most hospital wards are understaffed and thus unable to ta
Other concurrent and competing	(barrier) - lack of time: concurrent and competing family ob
They expressed concerns about t	concern about additional workload that would be required w
Physicians highlighted other conc	staff burnout
, especially for patients who live f	FOR - rural or further away people
, especially for patients who live f	FOR - limited mobility
Reduced burden of health care a	data stress
physical stress	data burden
the future reduction of waiting ti	reduced waiting times -quicked access to care
the future reduction of waiting ti	reduced waiting times -quicked access to care
Perception that patients and care	Reassurance
Physicians perceived that telecon	workflow
Also, there were cases where the	quicker treatment
(1) early detection of cases that s	earlier detection of cases that need to be referred
Perception that the patient is bet	data management
Perception that the patient is bet	I believe they will be better treated and more satisfied with
Perception that the patient is bet	Less visits for the patient
Perception that the patient is bet	data treatment
Teleconsultations may contribute	expedited communication - efficiency
Physicians discussed that real-tim	better accuracy/documentation
financial stress of going in-person	reduced finanical stress of going in-person
Perception that patients and care	data positive reception
Perception that patients and care	data interaction
Cardiologist 4: I think patients the	sense of reassurance helped with acceptance
Physicians stated different prefer	the use of videoconferencing for synchronous communicatio
[illegible]<br>informatics, however it was	data technical support
[illegible]<br>informatics, however it was	data time
[illegible]<br>things, to	data medical decision making
think it makes a difference becau	workflow efficiency
Sometimes, they [the GPs] can m	data diagnosis
[illegible]<br>cannot examine the patient	HCP collaboration
Technical issues sometimes led to	audio cutting out
[illegible]<br>would occasionally	audio cutting out
Cardiologists noticed that they ca	data clinical testing
senses (hearing and seeing).	data senses
stated that administrative suppor	Workforce
Often, the timing of teleconsultat	data scheduling

When they were scheduled in adv	scheduling in advance
Our schedules are always busy wi	Time
waiting and logging in)	wating for patient
"the least favourable is, without a	data time
Program financial reimbursement	
safety/liability/privacy barriers	safety
General access barriers to technology	data access to technology
lack of internet connection	data access to internet/wifi
patients lack access to technolog	
Lack of patient access to exercise equipment	access to exericse equipment
videos adapted to frail elderly (lo	
videos that don't require equipm	
Patient difficulty using technology (education and RE	data digital literacy
secure and private	data
safety/liability/privacy barriers	privacy Barriers
During the pandemic, eligibility criteria for open CRPs shifted to	patients attending open CPR were often less sick - potential
monetary funds for exericse equi	data equipment
reduced face-to-face sessions (m	exercise tests, risk strafic
lack of equipment	lack of (tech) equipment
exercise safety concerns	staff
exercise safety concerns	patient
tele-rehabilitation program not se	program not set up
safety/liability/privacy barriers	liability
patients declining virtual care wa	data declining
tech support/training for staff & p	for staff
tech support/training for staff & p	for patients
barriers related to hospital appro	
easy to use for patients and staff	for staff
easy to use for patients and staff	for patients
redeployment/laid off	data staffing
increased time to deliver individu	
increased patient access in rural a	
Good "task-technology fit", i.e., H	HCPs perceive the DHI as valuable in practice
electrophysiologists considered the tool useful for standardizing	
Perceived HCPs' clinical demand f	the demand for screening support among
Perceived HCPs' clinical demand for the DHI ("Several of the electrophysiologists emphasized t	

and the prediction tool could fun	specific junior to senior help
Easy access to the DHI and allevia	easy access
Transparency regarding the data	Knowing the data creates transparency and enables in-situ v
Good "task-technology fit", i.e., H	was high when patient cases concerned VT/VF, as the risk pr
Good "task-technology fit", i.e., H	In cases where the task-technology fit was lower (Case 1, Ca
Collaboration with local HCPs on	and adaption of the tool to establish a human-AI partnership
task-technology fit was lower	cases I have looked at, the alarm has not given me much.
Ability to personalise the DHI to l	remote monitoring clinics may want to be able to adjust the
Ability to personalise the DHI to l	threshold of the prediction tool
Collaboration with local HCPs on	and allowing the local team of clinicians to decide collectivel
Collaboration with local HCPs on how they will decide to trust the DHI ("Therefore, initial impl	
Collaboration with local HCPs on how they will decide to trust the DHI ("Therefore, initial impl	
DHI development in collaboration	
Collaboration with local HCPs on how they will decide to trust the DHI ("Therefore, initial impl	
Adoption can also be achieved through building trust in the tool by means of validation studie	
"Several electrophysiologists expl	based on team experiences from real-world use : positive ex
DHI development in collaboration	clinical practice - be carried out as close as possible to imple
Good "task-technology fit", i.e., H	had expectations that the prediction tool would bring new a
tool would bring new and more	that's fine, but it's not something groundbreaking, and
expectations and DHI	
Perceived HCPs' clinical demand f	high demand for workflow support
Perceived HCPs' clinical demand f	due to the increasing number of nonspecialized hospitals
It's always nice when something is supportive, I would say, but isn't it a "nice to have" and not	
something is supportive, I would	low perceived need
Managing HCPs' expectations abo	
Managing HCPs' expectations abo	HCP expectations!
Trust in the accuracy of the DHI (i	measurements/prediction
"Several electrophysiologists expl	and from experiencing that the prediction tool actually confi
Easy access to the DHI and allevia	alleviates the need for clicking through several web pages in
Easy access to the DHI and allevia	easy access
DHI development in collaboration	
Easy access to the DHI and allevia	readability - as well as how well it presents data
the prediction tool could decreas	ould decrease electrophysiologists' patient information sear
One HCP would have liked more i	PA done and a reason if they do not engage in PA (e.g., bore
Perception that alerts help promp	
and made them ask 'why?' when	increased questioning and understanding of patient
or did not think their patients we	

Perception that too many best pr	
One HCP would have liked more i	PA done and a reason if they do not engage in PA (e.g., bore
Perception that it's not disruptiv	not disrupting clinical care
Not using the suggested features	
Not using the suggested features	preferences for doing something a different way
or did not think their patients we	not listening to the suggestions by the system because of be
Perception that it's a good idea,	
easy to navigate	data user-friendly
Several HCPs expressed the desir	
Perception that too many best pr	
Ease of use, speaking of the simp	
Others commented that having the ability to examine vital signs trends remotely would be inv	
Reflection that the system acts as	
Participants also commented on t	monitoring
Others commented that having th	
Others commented that having th	monitoring
Reflections on wireless device ma	create efficiency in nursing work
Nurses found that the hospital-to	allowed them to use their critical thinking skills
Many felt that the clinical interfa	timely decisions about clinical action
Reflections on wireless device ma	patient safety
Reflection that the system acts as	clinical nursing skills - acts as an enabler of their clinical nurs
Participants also commented on their perception of Guardian as an aide to day-to-day nursing	
Reflections on wireless device ma	automation of data entry
Participants also commented on their perception of Guardian as an aide to day-to-day nursing	
Some nurses cited unfamiliarity w	unfamiliarity
Lack of familiarity and confidence	unfamiliarity
Lack of familiarity and confidence	confidence
Lack of familiarity and confidence with the technical aspects of the eTrAC interface were discu	
Participants also commented on t	of Guardian as an aide to day-to-day nursing work
they emphasized the importance	
they emphasized the importance	
Many felt that the clinical interfa	reassurance for HCPs
Some nurses cited unfamiliarity with more advanced aspects of the system as a barrier to eng	
Ease of use, speaking of the simp	Ease of use
Many felt that the clinical interfa	readability - the alerts and the scores are visible and you ca
Ease of use, speaking of the simp	of use
Most participants commented that they needed additional prompting to assign hospital-to-ho	

Lack of familiarity and confidence	technical support
Participants also commented on t	takes the workload off of everyday care
Reflections on wireless device ma	I think half our shifts are wasted with data entry—we don't
Reflections on wireless device ma	less time needed for data entry
Combining telehealth with SMBP	patients check in more frequently
My health center began distributing home blood pressure monitors to patients (40 (61.54%))	
access to care	data access to care
Lack of patient access to devices	To home BP device
difficulty getting devices to patien	problems giving devices to patients
difficulutes around ensuring devic	data validating
care teams' difficulty receiving ac	not receiving sufficient data
care teams' difficulty receiving ac	not receiving accurate numbers
We expanded what we had already been doing with our home BP monitoring program and "A	
Technical difficulties using device	data technical issues
We expanded what we had already been doing with our home BP monitoring program and "A	
device coverage	difficulties with device coverage for patients
"My health center began using h	during telehealth encounters
prioritizing telehealth make neces	prioritizing
My health center started a forma	between patient and HCP
My health center started a formal protocol to train patients to take blood pressure measurem	
Challenges around ensuring patie	data validating (size)
"My health center began using home blood pressure measurements for vital signs during tele	
We expanded what we had already been doing with our home BP monitoring program and "A	
4 cardiologists (67%) stated a pre	when the data should be available for the HCPs
Perception of usefulness in mana	usefulness of management
Perception of usefulness in mana	usefulness
Self-efficacy	
Dedicated time for HCPs to delive	Designated time
outcome expectacies	
HCPs' concerns about algorithm-c	taking away from patient-centred care and shared decision-r
"Some nurses had concerns that one-way notifications conflict with their role of providing tail	
HCPs receiving positive patient fe	
HCPs receiving positive patient feedback about the DHI ("Perceptions about supporting patien	
HCPs' concerns about algorithm-c	one-way communication (only provider to patient, not other
HCPs' concerns about the reliabili	notifications for patients not being received
perceived acceptability of the intervention to patients	
Training about the DHI for HCPs to imcrease HCPs' self-efficacy and outcome expectations ("P	

HCPs' concerns about algorithm-driven decision-making (in the DHI) and/or one-way digital co	
HCPs' concerns about the reliability of one-way digital communication cia email ("GPs' concer	
"Administration" barriers (26 (27%))	
"Data security" (13 (14%))	data security
"Lack of data for patients benefit	lack of data of benefits
"Purchase of technology equipment" (23 (24%));	
"Poor internet connection" (12 (13%));	
"Present insufficient reimbursement" (25 (26%))	
"No reimbursement" (17 (18%)); "	
Quick and easy for the HCP to del	workflow - enrolment
HCP perception that DHI is clinica	DHI is clinically worthwhile
HCP perception that DHI is clinica	ongoing support to patients with high BP that the practice w
HCP perception that DHI is clinica	data educating
HCP perception that DHI is clinica	viewed as a simple way of delivering education
HCP perception that DHI is clinica	and addressing a clinical/service need
Quick and easy for the HCP to del	Quick and easy for the HCP to deliver the DHI
[agree and strongly agree added	
For encounters involving single is	
For encounters involving single is	
For encounters involving single is	clinically
During the COVID-19 pandemic, p	of care
Providers expressed concerns about patients "falling through the cracks" when administrative	
amongst provider survey respond	
During the COVID-19 pandemic, patients and providers agreed that virtual care for ACSCs (am	
The predominant sentiment amo	
The predominant sentiment amo	
The predominant sentiment amo	Financing/cost-saving
The predominant sentiment among focus groups participants was that virtual care – dependin	
Overall, providing the option of v	respect for individual preferences
Overall, providing the option of v	
allow for short check ins ""In addition to, you know, the usual every 3, 6 or 12 month appoint	
A shared frustration among patie	
safety and feeling of loneliness ''	
safety and feeling of loneliness ''	
waiting times ""But that for me is the most frustrating … if I'm running behind, the patient get	
Overall, providing the option of v	
Effectiveness: "From a standard c	pretty confident diagnosis and management plan

Effectiveness: “From a standard c	pretty confident diagnosis and management plan
allow for short check ins "“In add	shorter check-ins more regularly
[illegible] involving multi-morbid patients	
[illegible] involving multi-morbid patients	
; allowed HCPs to sufficiently add	address patient needs
same quality of care using video (	video
same quality of care using phone	phone
For encounters involving single is	
[illegible] involving multi-morbid patients	
However, providers expressed dif	not able to provide emotional support
However, providers expressed dif	hard to communicate complex information
Some providers and administrativ	feels more impersonal
waiting times "“But that for me is	nobody to inform the patient that the doctor is running behi
Patient and HCP relationship com	relationship compromised
[illegible] involving multi-morbid patients	fear of making the wrong decision
A shared frustration among patie	
A shared frustration among patie	
Some providers and administrativ	
Some providers and administrativ	
Some providers and administrativ	
For many of these reasons, phone	(lack) access computer
Financing/cost '“If a patient does	
[illegible] involving multi-morbid patients	Severity - For complex encounters involving multi-morbid pa
[illegible] involving multi-morbid patients	if I don’t think they’re going to fully understand the risks an
Provider focus group participants echoed concerns that virtual care for ACSCs (Ambulatory Ca	
A shared frustration among patie	Lack of communication - to patient
[illegible] involving multi-morbid patients	providers felt care is compromised in the virtual environmer
The predominant sentiment among focus groups participants was that virtual care – dependin	
[illegible] involving multi-morbid patients	
[illegible] involving multi-morbid patients	
Provider focus group participants echoed concerns that virtual care for ACSCs (Ambulatory Ca	
Provider focus group participants	sensory impairments,
For many of these reasons, phone visits were viewed as particularly easy to access compared t	
Provider focus group participants	crowded environments
Financing/cost '“If a patient does	
Requirements for long-term sustainability of virtual care for ACSCs: (1) maintenance of physica	
Effectiveness: “From a standard c	e… And if we have access to the other testing that we would

For complex encounters involving multi-morbid patients	when a physical exam was needed
Patient and HCP relationship com	from video
; plan to continue using video visits (80%)	
waiting times "“But that for me is the most frustrating … if I'm running behind, the patient get	
Provider focus group participants echoed concerns that virtual care for ACSCs (Ambulatory Ca	
Requirements for long-term susta	
waiting times "“But that for me is the most frustrating … if I'm running behind, the patient get	
Effectiveness: “From a standard c	perception that if you already had a first encounter then one
Requirements for long-term susta	through medical education and professional development
Patient and HCP relationship com	better to get to know the patient more personally
For many of these reasons, phone	
For many of these reasons, phone	phone visits
For many of these reasons, phone	phone visits
Some providers and administrativ	not having the time
Requirements for long-term susta	technical support - hiring
Requirements for long-term susta	admin - hiring
Requirements for long-term susta	clerical support - hiring
Requirements for long-term susta	
Providers expressed concerns abo	was not optimized for a virtual environment
Providers expressed concerns about patients “falling through the cracks” when administrative	
waiting times "“But that for me is	no assistant - Nobody is there to tell them the doctor is runn
[illegible] general, it's [virtual care] led to	comproimised relationship
; patients from diverse backgrounds can benefit from video visits (85%);	
same amount of time on the video visit (70%)	
; same amount of effort (55%);	
HCPs' ability to access DHI data remotely ("However, having the ability to access data remotel	
HCPs' ability to access DHI data re	worfklow- less in-office disruptions for providers
Lack of internet access and poor i	
Lack of DHI flexibility to adapt/mo	HCP needs
Lack of DHI flexibility to adapt/modify according to local end-user (HCP) needs ("Although the	
DHI not efficient for HCPs within t	contributing to “alert fatigue
Lack of DHI flexibility to adapt/mo	lack of autonomy
DHI providers to engage with and understand/address HCPs' needs ("Conducted a needs asses	
DHI providers to engage with and understand/address HCPs' needs ("Conducted a needs asses	
HCPs' ability to access DHI data re	In addition, the need to utilize and securely store vast amou
Lack of internet access and poor i	poor internet speed
DHI not efficient for HCPs within their patient management workflow ("Despite such efforts, u	

Lack of integration with existing e	Lack of integration with existing electronic health record sys
Lack of DHI integration to existing	existing IT systems
Lack of integration with existing e	
Availability of technical support to	easy-to-understand user manual
DHI not user-friendly for HCPs;	
DHI not efficient for HCPs within their patient management workflow ("Despite such efforts, u	
DHI not efficient for HCPs within t	ncluded too many “clicks” to accomplish a given task,
Availability of technical support to	technical support availability
HCPs' ability to access DHI data re	with reduced travel for study staff
Lack of integration with existing e	
Lack of integration with existing e	
Lack of DHI integration to existing	need for double log-in and double entry of patient data
high mobile phone coverage and	High mobile phone coverage
internet connectivity, poor	poor technology
high mobile phone coverage and	Good internet connectivity
multiple systems making it	Patient data across multiple systems
internet connectivity, poor	Poor internet connectivity
interaction e-health platforms.	lack of provider–patient interaction e-health platforms.
health platforms emerged from	Lack of interoperable e-health platforms
A doctor said: “[an important dri	making diagnosis earlier
all the doctors agree that IoHT te	better management of specific cardiac conditions
All the doctors interviewed agree	data medical decision making
, and have a better result from th	data patient management
as well as job quality seems to be	data job quality
About the perceived outcome of	error rates
About the perceived outcome of	communication
About the perceived outcome of	lowering Financing/costs
such in the case that a patient wh	safer clinical way
The main answers were, in fact,	Allowing HCPs to have autonomy and participation in the de
patients (e.g., patients’ literacy,	literacy
patient complaints related to lack	lack of technical knowledge
Ageing	ageing
patients (e.g., patients’ literacy,	ageing
schooling for patients [is	Less school, means less likely to uptake among patients
past experiences	data
A doctor said: “ . . . the most impo	patients believing the HCP is always monitoring, even during
and the perception of a good serv	data empowerment

economic motivation serves as a	positive pressure
All the doctors have positive perc	Seen as non-threatening to their jobs
All the doctors have positive perc	seen as supporting their jobs
One doctor said: “these new met	sensitivity
Doctors have positive perception	Doctors think that patients will have positive reactions
Fear for the unknown	data fear
patients (e.g., patients’ literacy,	usability
. . . It depends, for example, for	data organization
for those who do not have a good	data organization
patient feels more protected and	patient more protected/followed
“The patient can quickly contact a	patients can get a quick answer regarding a symptom
Perception of better patient quali	easy access to care
the improvement of its quality of	data quality-of-life
others see the IoHT applications a	could allow for seeing patients as part of the team
highlighted active and aware	collaboration in terms of decision making
perceiving that it would be increa	
whether patients with a history	terms of AI
characteristics should be	
suggested catering to education	education levels
motivate patients with a history	to time since MI and patient goals
in frequency over time and be	decrease over time
in frequency over time and be	the patients with a history of MI complete their goals.
user based on needs.	
specialist follow ups, and	
entered	
whether patients with a history	choice if they want it or not
whether patients with a history	
were discussed, including	
theories, behaviour	
characteristics should be	
encouraged feedback loops	feedback loops
should be determined based on	
to be	
were discussed, including	theoretical approaches are necessary
entered	reducing redundant notifications
all socioeconomic groups,	how to finance the app
motivate patients with a history	

hierarchical structure that	
that are lower down	
the novelty effect, including	
motivate patients with a history	
communicate	
regulations	Regulations
during or near the end of	cardiac rehabilitation
patients with a history of MI will	confidence
member	family member becomes a champion
have a “champion in	genearl - family or friends
including additional features, a	
history of MI can meet potential	
functionality to see friends’	
“patient.	
whether patients with a history	not many (half) would use it
have	not a fan of using the phone
to be	
navigator should be used to	videos
the “champion” as a “buddy”	
with a history of MI will not	
importance of the output being	
disclaimer on the	need to ensure trustworthiness by flagging false info
encouraged feedback loops	
technology format, including	
hierarchical structure that	
suggested catering to education	easy to understand content
importance of the output being	interpretable by HPs
entered	visual clutter
appeals, such as larger headings	
dedicated health care team	
table 4	Age <60 years
table 4	Working city (compared to <100.000 inhabitants)
table 4	Relevance perception of telemonitoring as complement of f
table 4	Knowledge or use of a professional remote monitoring platf
table 4	Knowledge or use of a professional remote monitoring platf
table 4	Currently using a professional platform
table 4	No need training to practice telemonitoring

table 4	
table 4	higher expectations of patients telemedicine preferences
doctors considered security	security confidentiality is highly important
half of the responders answered	
62% referred to need	need for training
themselves well trained to	others well trained
half of the physicians considered	
answers, according to the	
around half of the health	
Interoperability of the DHI with e	electronic health record
driven data collection.	
Lack of wireless access;	
provide functionality to request a spiritual provider or social worker based on remote monitor	
DHI is accessible in terms of language and literacy ("Suggestions to improve accessibility, langu	
DHI is accessible in terms of language and literacy ("Suggestions to improve accessibility, langu	
provide functionality to request a spiritual provider or social worker based on remote monitor	
provide functionality to request a spiritual provider or social worker based on remote monitor	
HCP can personalise the DHI functionality to the individual patient's needs ("Many providers d	
Ability to tailor data privacy/data	HCPS can tailor data-sharing options
Ability to tailor data privacy/data sharing function to individual patinet's needs/preferences ("	
disabilities such as diminished	
disabilities such as diminished	those with vision problems
disabilities such as diminished	hearing problems
Difficulty integrating DHI into clinical workflow ("A few providers mentioned technical difficult	
Interoperability of the DHI with e	clinical systems
Difficulty integrating DHI into clin	clinical workflow
Difficulty integrating DHI into clin	ability to fit sensor-generated data monitoring into the clini
DHI-generated data not easily uable in clinicla practice ("However, many providers raised conc	
Interoperability of the DHI with other Apps ("Beyond clinical interoperability, providers discus	
End-user errors;	errors by patients
Data collection is appropriate to t	concerns of only trusting subjective data from patients
were concerned with facilitating	was seen as a barrier - HCPs also thought objective data wou
DHI collects data in a patient-cen	just patient-reported outcomes
DHI collects data in a patient-centred / convenient way;	
DHI presents HCPs with good visualisations of data ("Providers almost unanimously preferred	
DHI presents HCPs with good visualisations of data ("Providers almost unanimously preferred	
no associated plan as to how to monitor that data;	

no associated plan as to how to monitor that data;	
The technology allows an abundance of data to be	quantity - large volume of transmissions
Lack of motivation among health professionals (3.42)	
Limited access to electronic devices (3.80);	
Inability of patients to use smartphones (3.81)	
Risk of data privacy breach is too	data privacy
Lack of evidence regarding its eff	lack of evidence
Lack of reimbursement/funding c	
Lack of motivation among patients (3.66)	
Lack of legal regulation of digital	lack of legal regulations
Lack of reimbursement/funding c	
Lacl of recognition of and/or trus	in the DHT in general
Lack of evidence regarding its eff	lack of evidence of its cost effectivness
Panels most valuable for switchin	identify subgroups at risk of CVD using routine care of a pan
Panels most valuable for switchin	switching medication
More personalized consult frequency;	
More personalized consult freque	frequency
Less care burden for patient and professional;	
DHI could imporve lifestyle, self-management, and shared decision-making;	
DHI could imporve lifestyle, self-management, and shared decision-making;	
DHI could imporve lifestyle, self-n	lifestyle
concerns about not capturing a risk factors within the EMR	
Panels most valuable for switchin	perceived benefit for those with uncontrolled hypertension
Limited reach among the low-lite	low literacy
Cooperation agreements with external partners in the region are essential for establishing the	
Not all risk factors can be express	lack of qualitative data extracted
patient desire (and ability) to pur	for a trial period
Reliance on one-time subsidies ra	lack of compensation for end-users to implement the interv
Reliance on one-time subsidies ra	no structural reimbursement
Reliance on one-time subsidies rather than structural reimbursement and the lack of compens	
patient desire (and ability) to pur	(and ability) to purchase their own blood pressure monitor
Pre-existing technical infrastructu	pre-exisiting technical infrastructure in place
patient desire (and ability) to pur	especially if they can connect the device to the EPD
Implementation of extra digital d	EMR - medical record
Interoperability between systems in the region and healthcare domains;	
Implementation manager respon	Local representatives
patient desire (and ability) to pur	e motivating for patients to receive reimbursement for a tria

patient desire (and ability) to pur	desire
‘Earlier, we organised two evenin	patients seeing their GPs made a difference
Cooperation agreements with ext	having external partners (regional)
clear agreements about the<br>evaluation of implementation	
Inability to use devices that patie	patient owned)
Clear description of the purpose and benefits of panel management;	
such as introductions, training<br>sessions, and e-learning	
Clear instructions and training during kick-off meeting with all stakeholders;	
Clear instructions and training during kick-off meeting with all stakeholders;	
Clear manual at the start of the ir	clear manual
Ambassadors being GPs or PNs in	seen as essential opinion leaders
an implementation manager acco	implementation manager
Implementation manager respons	in-charge
specifying whom to contactfor te	technical support
There should be a clear protocol<br>for managing patient panels. (…)	
There should also be a<br>standardised implementation	standardised implementation plan
Less care burden for patient and professional;	
no technical tasks directed<br>to PNs	
It might provide more work due t	more work
Excessive data processing may le	data processing
Good communication with NHSE & within integrated care systems (frequent opportunities to s	
Differential digital access & litera	digital access
Storage & distribution of BPMs (c	unequal distribution
Equity concerns (Unequal distribution of BPMs might reinforce existing inequalities and issues	
Diversity of communication channels offered to fit each patient (including non-digital options)	
Clinical targets as incentives to in	
customized frequency of contact	reducing requests
Differential digital access & litera	digital literacy
Development of practice-specific	practice-specific
Differential capacities (skills set v	among different discplines
Good communication with NHSE & within integrated care systems (frequent opportunities to s	
Explain importance of BP tracking	CONTENT
Differential capacities (skills set vary and practices not used to working together);	
Differential digital access & litera	older
Differential digital access & litera	economically deprived patients
Equity concerns (Unequal distribution of BPMs might reinforce existing inequalities and issues	
Equity concerns (Unequal distribu	sick patients

BPMs on prescription / on loan fo	on loan
BPMs on prescription / on loan fo	on prescription
Lack of financial incentives;	
Additional funding would facilitat	
Limited & differential engagement with patients (the least engaged are those needing most ca	
Clinical targets as incentives to in	[illegible] money would be helpful. 130,000—
Incorporation into daily practice &	Incorporation into daily practices
Make it part of the day-to-day lor	
[illegible] integration of UCLP into EHR	integration of UCLPartners Proactive Care Framework for hy
Lack of guidance on IT & lack of unification of IT tools (multiplicity of resources available can b	
Centralisation at practice & PCN levels (one person per practice and one person at PCN level in	
Top-down approach that limits th	Top-down approach that limits the influence of HCPs on pro
Limited & differential engagement with patients (the least engaged are those needing most ca	
Person-centred care (explain to patients importance and customized frequency of contact by	
Shifting directions & lack of national guidance;	
Person-centred care (explain to patients importance)	
Assessment and training to make	
Assessment and training to make	
Extremely clear and exhaustive onboarding material and integration of UCLPartners Proactive	
Lack of guidance on IT & lack of unification of IT tools (multiplicity of resources available can b	
Centralisation at practice & PCN levels (one person per practice and one person at PCN level in	
Centralisation at practice & PCN l	One-person in charge at each clinic
Task-sharing between different a	
Well-placed role of pharmacists t	clearly defined responsibilty roles
Well-placed role of pharmacists t	use of pharmacists
Lack of human, time resources (c	Lack of human,
Lack of human, time resources (c	time resources
Top-down approach that limits the influence of HCPs on processes and high staff turnover tha	
Storage & distribution of BPMs (d	lack of BP cuffs
Lack of guidance on IT & lack of u	lack of technical support
[illegible] devise process & responsibilities	
Lack of guidance on IT & lack of unification of IT tools (multiplicity of resources available can b	
Limited tracking capacity (too tim	Limited tracking capacity
Substantial additional workload n	perceived additional workload
HCP perceive the DHI to address a	
HCPs perceive that DHI provides o	objective
HCPs perceive that DHI enables helpful extension of the therapeutic relationship beyond the a	

HCPs perceive that DHI provides (	more objective data - no longer have to rely on single measu
HCPs perceive the DHI to support	
HCPs perceive the DHI to support	
HCPs perceive the DHI to support	
DHI supports the HCP in providing personalised care, e.g., through advance information on the	
DHI supports the HCP in providing personalised care, e.g., through advance information on the	
HCPs perceive DHI to increase acc	
HCPs perceive DHI to increase acc	
HCPs perceive DHI to increase acc	
HCPs perceive DHI helpful for pat	helpful for patient education
HCPs perceive DHI helpful for pat	improved education
HCPs perceive that DHI enables h	on-going support after acute care
HCPs concerns about reduction ir	One HCP explicitly mentioned the importance of face-to-fac
DHI supports the HCP in providing	t eHealth could help them to get more information about pa
DHI can be personalised to the pa	
HCPs perceive the DHI to support	can control what information they receive and how they rec
HCPs perceive patients old age as	patients old age as a barrier
DHI ("Difficulties with	such as bugs and slow development of the technology
HCPs concerns about reduction ir	patients would generally prefer face-to-face contact over dig
HCPs concerns about reduction ir	One HCP explicitly mentioned the importance of face-to-fac
("Furthermore, it was frequently	low
DHI ("In addition, they	was no help desk for patients or HCPs
DHI ("In addition, they	Lack of technical support for the DHI
Telehealth technology has a majo	TO - Increased accessibility/medical care for rural people
"Of course, patients' files are ava	To - data
They also explained that they will	Greater access to medical records
accessibility ("The physician may	Accessibility of medical record 'off-clock'
Telehealth technology has a majo	Medical decision making
The presence of direct contact an	Prevents major damage/risk of late infectionsthrough increa
The findings from data obtained a	and procedures
effective and structured services	streamlined services
The healthcare providers clarified	optimizing consulation
More importantly, they will collec	Improved organization/documentation
accessibility ("The physician may	Documentation - Recording of consultation
("Of course, patients' files are av	To - data
The healthcare providers clarified	accredited data/scientific websites during consultation
patients "Currently, there is	FOR - Perceived benefits for bedridden and elderly patients

culture and development should	providers out of cutlural norms/preferences
telehealth systems or remote	accessibility issues. (general)
culture and development should	data cultural norms
highly secured; privacy (“It has	Clearly stated privacy rules
restricted to the person who is	data restricted access
Therefore, telehealth the CVD pa	data patient contact responsibility
the healthcare providers stated tl	deal with patients in a very intimate and friendly manner
telehealth systems or remote	CONCERNS of privacy
culture and development should	always available
The opinions of different particip	Qualified staff as a Facilitator for telehealth
58% indicated that they would use virtual visits for most patient follow-up visits;	
easy-to-use and user-friendly digi	easy-to-use
were least appropriate for patien	UnsuitableFOR specific patient groups, like heart failure
were least appropriate for very si	Unsuitable FOR sick patients
were least appropriate for patien	, cognitive impairment
were least appropriate for patien	hearing impairment
were least appropriate for patien	Unsuitable or least appropriate FOR patients with communic
the DHI (virtual visits) does not al	clinical tests - cannot perform the necessary clinical tasks
Specific tasks that were reported	clinical tests
observing nonverbal communicat	Human factors - reduced nonverbal communication
equipment required for virtual vis	data equipment
adopting an electronic medical re	data emr
provision of a physical setup with	data ready-to-use
reviewing medication list,	challenges with reviewing medication list
providing dedicated rooms or cub	data space
Not suitable for new referrals	Unsuite for new patients
provide patient instructions/train	data training
The preferred type of virtual visit	telephone
The preferred type of virtual visit	video
providing an easy-to-use and pati	easy-to-use
online requisitions and referrals	challenging was making online reuisitions and referrals throu
adapting other clinical workflows	workflow adaption to suite DHT
and establishing a consistent proc	use and administration of guidelines for how to conduct virt
The use of e-health technology w	
Other priorities for CVD research	predict
Other priorities for CVD research involved technology to predict and prevention cardiovascula	
and the importance of the patien	patient

and the importance of the patien	
perceptions about the success	educational content aligned
had internet access and used a	
had internet access and used a	used a cell phone to access the Internet
had internet access and used a	
failure included a lack of in-	difficult for some
failure included a lack of in-	a lack of diabetes-specific content
were already using the	
self-learning	
self-learning	
difficulties, the	difficult for HCPs to encourage their patients
failure included a lack of in-	lack o a lack of in-person contact, f in-person contact
delivery of the educational	
delivery of the educational	Live sessions
failure included a lack of in-	
failure included a lack of in-	and patient-friendly digital platform.
reported that the main difficulty	difficulties using the tec
failure included a lack of in-	difficult for some
failure included a lack of in-	not very intuitive for patients
CPs (clinical pharmacists) reporte	Patients interest in tech
and GPS admitted that they did n	Increased knowledge of AF-related risks
which in addition to AF, helped id	Helps identify other heart rhythem disorders
and was more reliable than conve	more reliable than conventional tools
comprehensive pre-	less time for pre-appointment information
unplanned parking	the waiting time
about patients who were	disadvantaged for those care home residents who had limite
considered to be more	type of setting
was the concept of a	desire for a personalised health screening plan
that AF screening could be	at the moment eligbility to AF screening has certain guidelin
AF was a particular challenge in	the nature of AF might make uptake hard
flexible system, giving	wait or return for appointment
about patients who were	housebound patients might have less access
follow-up Financing/costs as barr	Financing/cost of tech equipement
Financing/costs	data parking
GPs in the vaccination clinic said	data among hcps
about the AF screening role	data optimism
AF was a particular challenge in	data health

about the AF screening role,	data communication skills
pharmacists suggested targeting	increasing Accessibility of DHI by going to public locations
a little bit ad	data space
practice were thought to	
They expressed a strong 'faith' in	Strong faith in device
pharmacists' clinical abilities.	staff doubted pharamcists' clinical abilities
perceived pharmacists as 'shop	Perceived public perception of not taking pharmacists seriou
emphasised the convenience of h	
same-day screening seemed to	tech allows for same day screening
mentioned by GPS who	need for more staffing members
multidisciplinary team,	pharamacists
a typical community pharmacy	data staffing
less welcoming and less set up	related to atmosphere
complained about the co-	juggling two tasks meant limited time to complete tasks
through websites, text	data health
have to follow them up so it	perceived lack of time to follow-up on patients
of data to detect anomalies	
these tools	
diagnostic efficacy	diagnostic efficiency
misses/false negatives	
need	
are too common when clinicians	false alarms
[health] record without you	pull from the [health] record
automatically	
systems to	treatment decisions.
result in a loss of control	loss of control
administration—who are not	
security, I'm scared of artificial	
create better automated	with centralized data
champions of the tool need to	need to prove this, justify this
lack sufficient data to support a	value.
need	
dramatically improve outcome	
offer	no real evidence that improve patient management
support a new AI app	cost effective evidence
support clinical practice	with using AI
support clinical practice	with using AI

often poses a challenge owing to	of AI
cardiology-specific AI tools also	
expensive to implement	
and larger practices often do not	don't have the budget
are reluctant to invest in a new	are reluctant to invest in a new AI tool if low staff buy-in
usability of cardiovascularbased	(clinical) workflow
frequently are incompatible with	Tools from different vendors aren't compatible
administrators expressed	
automatically	
create better automated	same format.
most likely	
information officers	
administrators identified several	
administrators identified several	
department/specialty who will	
about not achieving a	
Cloudbased	CONCERNS - patient information,
in any	
about not achieving a	
are reluctant to invest in a new	if clinicians won't use then the hospital won't pay, relucant t
administration—who are not	Feel replaced
providers about a “super AI”	
these tools	lack of awareness/education on what tools use AI
clinical features	
represents a significant barrier	
understanding of AI, lack of trust	
security, I’m scared of artificial	
need	developed by third party vendors.
a big	pharmaceutical company
with other	
usability of cardiovascularbased	
AI app for	too complex - specific to EHR
these risk calculators.and EPIC, it	
frankly, finding vendors who can	amount of data
compiled and scanned for	
for	in the organization itself
organization are	Leadership changes

they are pressured due to time.”	
a sensor was being used for	current status
frankly, finding vendors who can	amount of data
frankly, finding vendors who can	have to analyze by hand,
predictors,	
burden for clinicians	
and patients remained motivated	
Several participants also admitte	found quality of care was not diminished
Many were pleasantly surprised t	improvements in physical fitness
You can see huge differences in t	data health outcome
When participants considered the	of patients' home environment
use of physical handling	data alternative measurement
“I feel that the patients are benef	feeling happy to be able to provide care and seeing that pati
Participants spoke about how sor	it limited access to people who had technology
and some of them don’t have em	don't have email
a lot of people were not that fam	
One of our biggest challenges is t	
and lowering Financing/costs ass	lowering Financing/costs
Participants also spoke about the	cannot do a test to measure physical capacity online
Because of the inability to condu	patients had to come to do test
Discussing post-pandemic utilizat	data perceived value
… and I do feel from the patients’	they feel value in getting this treatment
Participants shared concerns for	data exercise
People feel supported and conne	
and I would sometimes spend 45-	time to train the patient on how to use zoom
A hybrid approach that combines	
patients can transfer exercise tra	learning exercise at home, already sets them up for a good e
improving accessibility related to	data access to care (rural areas)
Participants also valued the abilit	
makes transition to at-home care	easier transition to home environment
As noted by most participants, te	fosters social support
another challenge was the initial	setting up patients/providers on the platform
Useful features of the DHI based	Integrated into EHR
The IDeA also seemed to reinforc	knowledge
The IDeA also seemed to reinforc	confidence in their decison making
Most Clinicians, Regardless of Spe	More Successfully Conveyed Risk Information Than Verbal D
Cardiologists shared their assum	data health literacy

They highlighted that the IDeA he	Consultation now focused more on the patient preferences
It was noted that auto-populatior	Automation
("Cardiology clinicians shared tha	based on clinical needs and preferences for who needs it mo
choosing to use the IDeA with pa	clinicians mainating autonomy over use of DHI
as this is required in certain parts	already included in their tasks, they have to include this step
Primary care clinicians, however,	Incorporation into current clinical decision making processes
as well as the opportunity to cut a	Ease-of-use ; already part of the EHR
perceived efficiency of the visual	Visualization - easy to read data
time-saving features of the DHI	Time-saving
The DHI would be more trustwort	by clinical leadership
Clinicians expected that the syste	information flow
Clinicians expected that the syste	information flow
Clinicians expected that the system would replace the need for patients to supply information	
The DHI would be more trustwort	embedded it into current scope of practice protocols for RNs
Efforts will be needed to minimiz	c. from the EHR into the monitoring note template
Efforts will be needed to minimiz	enrolling the patient in the continuity HF clinic so
Efforts will be needed to minimiz	nd possibly asking the patients to send in their vital signs and
The DHI would be more trustwort	more information regarding the basis of the notification;
Communication flow should be as	tools need to be standardized across the clinic
Communication flow should be as	communication flow
Physicians thought that implemer	standardize protocals
Physicians thought that implemer	reduce medical errors
Physicians thought that implemer	for timely treatment modification
Text message-based reminders w	Retention
Text message-based reminders w	Adherence
This was best supported by a nurs	TO - mobile phones: Increased patient accessibility to mobile
about the fidelity of physicians’	varied CVD manifestations and prognostic factors
acceptability of text messages,	Need for local language translation
be effective, it can complement	emanating from the national program for prevention and
sincerely hope that because we	introduced DSS in the National program
interface	
some physicians, slow typing	slow typing skills
some physicians, slow typing	Technical skills - Lack of HCP technical skills
This was best supported by a nurse: ‘[E]ven a person in village today has a mobile, he [patient]	
DSS (desicision support system)	healthcare professionals
opposed the use of EHR-DSS as	Perceived rejection from older, more experience HCPs
that the hospital leadership	

be effective, it can complement	activites
obtaining legal permission,	lack of leadership - hard to convince the CEOS
that the hospital leadership	to support clinic change
about the fidelity of physicians’	manifestations
obtaining legal permission,	concerns with obtaining legal permission
C-QIP strategy, key informants	data collection etc
obtaining legal permission,	CONCERNS- data privacy
obtaining legal permission,	CONCERNS - data privacy issue
intervention drives the need for	Reluctance of technology due to perceived view of taking av
intervention drives the need for	Reluctance of technology due to perceived view of taking av
opposed the use of EHR-DSS as	Perceived rejection from older, more experience HCPs
some physicians, slow typing	Technology-averse attitudes
sincerely hope that because we	Resistance from HCPs
that the hospital leadership	
some physicians, slow typing	(barrier) time to learn how to use a new DHI system
intervention drives the need for	educate them
intervention drives the need for	all implementation actors
understanding the needs of	that patients expect medical doctors to do the counselling;
Further, physicians supported the	Workforce - Need for NPHWs (non-physician health workers
strongly believed in the involvem	Workforce -Need for NPHWs to provide patient education a
Health administrators expressed	Workforce - Need for various implmentation actors - NPHWs
intervention drives the need for	Clear plan for implementation
obtaining legal permission,	implementing something with multiple stakeholders -
sincerely hope that because we	Workforce - Need for additional support to manage data
that the hospital leadership	
C-QIP strategy, key informants	data collection etc
C-QIP strategy, key informants	was perceived as one way to help create an environment
some physicians, slow typing	(barrier) time to learn how to use a new DHI system
hospitals have introduced all this	Increased workload for entering the data
consultations	consultation and travel time
perceived telemedicine	workflow
because I’m more efficient	
monitoring data,	
consultations	consultation
possibility of clinicians	
a difference in “chemistry”	
a difference in “chemistry”	

consultation was that	communication discrepencies
some technical	
consultation was that	Latency delays
access to a computer with	a webcam, personal headset and high-speed internet,
choice of modality was very	equipment for video consultations:
choice of modality was very	
face consultation	sensitive topics - inappropriate for that
based on the purpose of	worsening patients were seen to be preferred for in-person
choice of modality was very	
give patients the	
patients and healthcare systems	COVID-19 - patients more receptive to telehealth
patients and healthcare systems	COVID-19 -healthcare more receptive to telehealth
was deemed by most	lack of physical examination
perceived a significant increase	currently no shared EHS
disruptions would occur on the	to internet connection issues,
access to a computer with	computers not set up properly
perceived a significant increase	currently no shared EHS
agreed that new	
access to a computer with	
access to a computer with	
expressed fears of top-down	
telemedicine posed privacy	privacy challenges
want	CONCERNS - of home environment
want	CONCERNS - of home environment
agreed that new	
based on the purpose of	
based on the purpose of	
deemed that clinics were	
possible by video but perceived	
deemed that clinics were	
perceived that more preparation	
consultations	less waiting time (waiting for patients)
possibility of clinicians	saving a journey into hospital,
that more administrative	more admin work
that more administrative	for admin work
perceived a significant increase	the General Practice
as to whether telemedicine	

Participants also identified that a	
collect real-time and day-to-day	
may positively affect their ability	timely delivery of care
may positively affect their ability	check-ups
may positively affect their ability	check-ups
may positively affect their ability	check-ups
providing opportunities for	apps could limit clinic visits,
collect and assess real-time	prevent adverse events.
trends in these data to better	better assess the patient
by being able to collect, share,	prevention of HF hospitalizations.
apps that use artificial	alerting patients who need immediate care
providing opportunities for	it save patient time
providing opportunities for	related to costs - including travel and parking Financing/cost
providing opportunities for	related to costs - including travel and parking Financing/cost
patients face difficulties with	
of how an app supported	supporting care for sick patients
to take “ownership of their	
patients to have awareness of	
support patients in goal setting	Through goal setting
participants felt that apps presen	
Apps can also generate automate	
.. So, it’s that extra time, it’s not	patients have more time to ask questions/reflect
One of the frequently mentioned	data
Apps were viewed as a medium f	
A commonly perceived benefit to	provide nutrition educaiton
Apps could also support food skil	
It was also identified by several p	
Several participants thought that	communication between HCP and patients
A participant observed as follows: "Also, it may allow patients to kind of write down question	
We certainly have a high number of elderly, or frail elderly in our clinic so a lot of them aren’t,	
There was also concern that pati	esp in rural areas
There was also concern that pati	esp in rural areas
We certainly have a high number	of internet
Participants felt that apps providing personalized and tailored information to patients were va	
One participant saw a unique opp	
included a personalized medicati	
Perhaps prompting with a screen that says“you require a compliance of at least 90% to see eff	

It was noted that gamification ca	not suitable for patients with HF, who are on average older.
Apps can also generate automate	
the app that would automatically remind them: “Okay it’s time to take your pills.”	
the option for patients to persona	
the majority [of HCPs] were unaw	lack of awareness of credible apps to recommend
how technology savvy patients ar	
("Now to use it [app], our whole team would have to adopt it. Meaning they would have to ha	
Apps may also provide additional benefits when	So, if you can incorporate things that the patient can, let’s sa
Participants felt that HF apps nee	
In addition, they can potentially collect and assess real-time	
Participants viewed apps as being	
characteristics associated with aging, such as decline in physical and cognitive functioning, we	
Participants expressed that some patients may lack motor	may lack motor function, have arthritis, be missing digits,
patients have difficulty with their	difficulty with their vision
patients might experience cogniti	cognitive challenges
Participants felt that language ca	participants not comfortable with an app in English
Perhaps prompting with a screen that says“you require a compliance of at least 90% to see eff	
Participants expressed that integ	
the app that would automatically remind them: “Okay it’s time to take your pills.”	
Financing/costs involved in down	operating Financing/costs - downloading and using
If app use was time consuming, it	
("If I need to spend hours in each	no money
Some of them felt that	“fun,” “enjoyable,” “engaging,” and “interactive
Some of them felt that	
The HCPs felt that if an app for HI	needs to be designed in a mature way
Other participants questioned the	
A participant stated that they had	
It was noted that gamification ca	not suitable for the patient demographics
Other participants questioned the	perceived dislike from patients
and compatible with different types of devices and platforms (smartphones, tablet devices, an	
In addition, it would be beneficial	integrated into practice
it would be beneficial to have apps be integrated into health care system, including the hospit	
It was suggested that an app for H	with other apps
. Apps also need to provide simpl	meaningful information
and that those who are highly en	
An HCP commented as follows:...having the possibility	using messaging
In addition, participants were cor	Lack of in-person interaction

Participants felt that HF apps nee	patient confidentality
also helpful to have the data	It’s also helpful to have the data when the patient comes to
buy-in from HCPs and clinical staf	
. You might have a hard time gett	older patients might resist
Moreover, buy-in from HCPs and	
A participant stated that they had “never seen a successful cardiovascular gamification in an a	
time and workload required to	
. Apps also need to provide simple, practical, and meaningful information	
. Apps also need to provide simple, practical, and meaningful information	
as well as be easy to use, simple, user-friendly,	
trends in these data to better	
of sodium is a nice way in some	easy, visualiztion
because apps can present inform	
would be a visual reminder of ho	visualization
Moreover, technical support and	technical support
data sent to us then obviously,	
("If I need to spend hours in each	
time and workload required to	
time and workload required to	
time and workload required to	patient data from an app may interfere with app use.
Perceptions of ICT (information a	perception of essential
Perceptions of ICT (information a	convience
Perception that ICTs allow for the	objective
Perception that ICTs allow for the opportunity to provide greater convenience to themselves a	
Perceived benefit for changing pa	
Perception that ICTs allow for the opportunity to provide greater convenience to themselves a	
Perception that ICTs allow for the	giving greater knowledge about the health status of the pers
The usefulness perceived by heal	ensuring greater comfort
Perceptions of ICT (information a	
Perceptions of ICT (information a	monitoring
Highlighting that ICT has the pote	in the future of prevention of complications
The importance and potential of	monitoring
The importance and potential of	safety of patient
Preference in the use of technolo	
Perception that it is important to	interaction of patients : choice
Perceptions of issues regarding se	
Problems associated with the ma	problems with the manual insertion of data

An important finding of the techn	
Perceptions of issues regarding se	CONCERNS
Perceptions of issues regarding se	CONCERNS
Perceptions of ICT (information a	perception of essential
Perceptions of issues regarding se	
Perceptions of issues regarding se	a clear denial of confidence of data security
Increased opportunities for healt	easier to follow-up on their patients
PEs (peer educators) viewed mHe	PEs (peer educators) viewed mHealth mobile-based message
mHealth messages are seen not j	Support PEs in their work
sharing phones meant that the ta	Sharing phones - limited accessibility
They found it was desired that m	reduced interaction - Less workoad - less frequent interactio
Participants highlighted that olde	Phone literacy
Other barriers to cell phone acces	limited cell phone literacy
Participants highlighted that olde	Older people have less accesss/phone literacy
Participants highlighted that olde	poorer people have less accesss/phone literacy
Participants highlighted that olde	Rural communities less accesss/phone literacy
low promotional SIM card	patients buying new network plans because of Financing/co
sharing phones meant that famil	Support from the community from sharing phones
and a preference for a familiar, f	(Facilitator) annoucing MoPoTyso before the start of the me
A few PEs preferred text messag	aligned to text-messaging
They found it was desired that m	reduced interaction - Less workoad - less frequent interactio
mHealth messages are seen not j	Health messages are seen not just as ways to educate, supp
participants viewed mHealth as a	Perceived patient benefit for supplementing information, m
participants viewed mHealth as a	the messages could help improve patient's practice lifestyle
Perception that patients with fre	good relations with their peer educator
the relative advantage (that mHe	Observed benefit
change	(barrier) desired outcome not achieved
interventions to solve structural	Equity care - will not increase access for those of poverty, lo
(e-health (tablet) only) included	due to numbers always changing
members so were not able to	TO reaching patients (barrier) accessibility - sharing phone n
access despite the provision of	TO - wifi in rural areas
and access to NCD care across	(barrier) TO quality care
These patients also had updated	had updated phone numbers
(e-health (tablet) only) included	TO mobile numbers
members so were not able to	TO - a phone number, patients sharing phone numbers
system for PEs or patients	ability to update numbers
adaptable once the study had	(barrier) not adaptable

Preference for mHealth message	alignment - Messages that align wiht PE's goals
with international and national	Intervention aligned with international and national plans
Literacy	literacy
adaptability and trialability (patie	Co-design with end users
adaptability and trialability (patie	Co-design with end users
Low education	(barrier) low education and literacy
interventions to solve structural	Equity care - will not increase access for those of poverty, lo
strength and quality of the evider	(facilitator) Validty of the intervention
maintain current phone	mobile phone coverage - (barrier) Financing/cost of the cell
phone coverage and tablets do	(barrier) inconsistent service of mobile phone coverage
not able to coordinate with the	Not integrated into exissting clinical workflows/systems
as a relative priority and were	
(e-health (tablet) only) included	Documentaiton - not updating new phone numbers in the sy
was not seen as a priority for	Digital health not see as a priority
eHealth groups wanted tablets	Desire for ehealth to facilitate work
Perception that patients with fre	good relations with their NGO
the MoPoTsyo system (linked	external policies
the MoPoTsyo system (linked	Incentives - high cell phone coverage among patients
Structural characteristics (MoPoT	work structure - (facilitator) growing, mature organization
complexity (messages were easy	easy to receive messages
tablets regardless of coaching by	User-friendly barriers - difficulty using the tablets
adaptable once the study had	(barrier) too complex
Structural characteristics (MoPoT	work structure - (facilitator) growing, mature organization
the MoPoTsyo system (linked	across different clinics
not able to coordinate with the	Increased workload
maintain current phone	(barrier) increased workload to follow-up on patients' chang
provides a phone call that PEs	
provides a phone call that PEs	Provides phone call that PEs would not have time to make
provides a phone call that PEs	(Facilitator) ehealth facilitates data entry
HCPs had confidence that the rec	HCPs had confidence that the recommendations from the Cl
Helps users identify their own ne	own need for further education
Perceived advantage of use as an	
The SGLT2 module encouraged collaboration between the staff and gave more autonomy to c	
Still requires the use of clinical ac	
With multiple modules available,	
HCPs had confidence that the rec	
Resources provided specifically fo	

Still requires the use of clinical ac	
Still requires the use of clinical ac	to factors such as age, health literacy, comorbidities, and lik
Still requires the use of clinical ac	
Still requires the use of clinical ac	comorbidities,
The SGLT2 module encouraged co	more autonomy to clinical health assistants
believed that to maximize the	
The SGLT2 module encouraged co	
Participants believed a lack of familiarity, training and knowledge with SGLT2 inhibitors hinder	
The SGLT2 module encouraged co	collaboration between the staff
Competing priorities and time pre	
Still requires the use of clinical ac	(by following the recommendations blindly, it would reduce
HCPs had confidence that the rec	and explaining to the patients to instill their confidence and
Not used when the consultation w	
Not used when the consultation w	
Competing priorities and time pre	workforce shortages
Competing priorities and time pre	
uncomfortable/not fitting cuff	
I usually spend for	helps focus on seenig only those in -person that need it
don't have to come	for the clinc
that issues with patients' BP	faster intervention
felt that their relationship with	
improved ownership and sense of empowerment by patients	
result is anxious patients due to DHI	
brought by the intervention. It	
saved patient's clinic vists, travelling and waiting time, especially for working patients	
saved patient's clinic vists, travell	
saved patient's clinic vists, travelling and waiting time, especially for working patients	
shown	
HCPs could receive patients' BP	
Sometimes [the time] I	for the patient in the waiting room
don't have to come	
acces to data - patients	
inaccurate readings due to cuffs n	
times as us…they will	
patient	history of stroke
consultations might	physical examinations better in person
consultations might	

Functions and interface was man	not automated
patient	
patient	
patient	non-working people
that “patients may feel more	
Gateway issues (technical) - connection issues	
Gateway issues (technical) - connection issues	
that “patients may feel more	not used to do this
that could integrate BP measuren	
usability - flight mode button clos	accidently switching to flight mode when meaning to turn of
of unfriendly	not answering the calls - unresponsiveness
of unfriendly	
Due to PDPA (personal data protection act), it keeps logging us out	
data confidentiality - unauthorize	CONCERNS
that “patients may feel more	lack of confidence for televisit
stage, like on standby,	it gets piled up more and more.
patient	if unable to self-manage
that “patients may feel more	to a person over the phone asking many healthrelated
patient	
that “patients may feel more	
patient reduced confidence and	of the treatement/intervention
usability - flight mode button close to shut off button	
need for more user-friendly machine	
not very neat and	
not very neat and	
not very neat and	not intuitive
the readings from the portal	
times as us…they will	
a Jobstack function was created	
healthcare	healthcare teams need to work with other clinic staff
care managers and care	limited staffing
care managers and care	limited time
call the patients, if	
every day to just	open time in the schedule to perform these tasks
every day to just	open time in the schedule to perform these tasks
stage, like on standby,	more care coordinators and care managers
stage, like on standby,	

stage, like on standby,	
times as us…they will	
not very neat and	
S005 (female, care manager)	
I usually spend for	
intervention become	phone follow-up with the trial participants concurrently.
Sometimes [the time] I	time spent on the call
don't have to come	
care managers and care	
the readings from the portal	
of unfriendly	
a Jobstack function was created	
I usually spend for	
prior experience with telehealth	
A lot of them [consumers] came l	
'And we've also found that it can be quite difficult to develop rapport with people from Indige	
The need for increased monitorin	
In addition to safety, monitoring v	confidence in exercising
While the benefits for clients wer	
However, clinicians in rural areas	
'I think a big uncaptured cohort o	
To support risk management, clin	objective data
However, strategies are required	indigenous people
health services and clinicians nee	
this involved a standardized train	and evaluation services
To support risk management, clin	of exercise
This was particularly pertinent fo	
The Financing/cost of using the ir	low
However, this would require bett	remote monitoring infrastructure.
but I think it (COVID-19) [helped u	COVID-19
'A lot of them are using that sort	with tech or telehealth
The Financing/cost of using the ir	
In other services, the lack of perm	to pay staff
A lot of them [consumers] came l	reduced touch
The ease of set up was greatly inf	
The ease of set up was greatly inf	the telehealth system or platform
'… [having] the system set in plac	portal and the virtual waiting room set up

Space and the need for dedicated	general ICT infrastructure - with appropriate hardware, soft
Better quality checks and evaluat	to perform evaluation services
the presence of telehealth champ	telehealth champions - staff that encourage telehealth use a
A lot of them [consumers] came b	thinking their vitals are not being checked
Additionally, financial incentives (	not motivating for HCPs
And while staff recognized that vi	
'[Telehealth] has probably opene	
whereas in cardiac with a recent e	concerns of patient safety
The ease of set up was greatly inf	also enable opportunities for one-on-one contact with the c
Space and the need for dedicated	a dedicated room
Space and the need for dedicated	
One state-wide service had built-	targeted areas of high needs,
Cardiac rehab patients are typical	
Cardiac rehab patients are typical	greater anxiety in both consumers and clinicians to be seen
I think if we can set it up well at t	
'I think a big uncaptured cohort o	'I think a big uncaptured cohort of people are our Aboriginal
Adoption was also supported by p	
Some participants also felt that staff willingness played a role in greater reluctance among the	
Some services, mainly located in	little interest
Some services, mainly located in	limited interest
When well-supported to use telel	tech support
training sessions whenever availa	
Many services are integrating hybrid models of care (e.g. provide a combination of in-person,	
The ease of set up was greatly inf	
Many services mentioned that es	
Resource issues placed additiona	Resource issues
the presence of telehealth champ	
Staffing issues due to recruitmen	understaffed
There was a clear need for a risk s	framework for who gets telehealth and who doesn't
To overcome limited staff resourc	pooling resources across a health district and taking turns fo
To overcome limited staff resourc	pooling resources across a health district and taking turns fo
There was also a desire to utilize	rural general practitioners or rural physiotherapists
The centralization of education m	of educational models
One service also felt that a state-v	greater partnerships between tertiary and primary care.
this involved a standardized train	
this involved a standardized train	of recording, and feedback of sessions
with developing updated safety p	

rolling programmes (e.g. consumers can commence at any week of the programme and contir	
Many services mentioned that es	
perception that home-based exer	adherence in the long-term
Both nurses expressed that video	psychological well-being - holistic care
The physiotherapists questioned	lack of touch to guide patients
This meant that the physiotherap	quality of the training might be reduced
perception that home-based exer	transferable
web-based sessions increased the	safety during training
The physiotherapists expressed w	
general functional level and	there might still be physical progress
the individualization created a po	based on their fitness levels
physiotherapists agreed that the	aligned with clinical guidelines/goals
HCPs could not continuously follo	patients challenged technically
("Sometimes, they needed a lot o	
technical problems during the we	data
Delays in starting the session and	
both nurses experienced difficulti	technical problems
In addition, intermittent poor inte	wifi
Thus, patients and physiotherapis	better physical fitness
The video call provided security a	past relationship - if the patient already knew the nurse befo
("Sometimes, they needed a lot of guidance because...then they switched the camera on in th	
The physiotherapists questioned the quality of the exercises performed, as the web-based tra	
Both nurses expressed that video	to see the patients' physical condition
In addition, intermittent poor inte	very small image
("Sometimes, they needed a lot of guidance because...then they switched the camera on in th	
("Sometimes, they needed a lot of guidance because...then they switched the camera on in th	
patients should be divided into gr	splittnig up by patient physical function ing
For the physiotherapists, the indiv	individualization seemed double-sided as it was time-consu
They felt the platform could improve accessibility to self-management 'support' in situations w	
Healthcare professionals perceive	data sharing
They felt the platform could improve accessibility to self-management 'support' in situations w	
Healthcare professionals also reg	more accountable for managing their health condition
Healthcare professionals also reg	support users to 'make some informed choices' and take a 's
Healthcare professionals also reg	informed choices
Healthcare professionals observe	nd I think the real benefit of a digital [platform] is that you ca
Healthcare professionals observed that an evidence-based platform that is compatible with us	
Healthcare professionals also outlined advantages of data sharing and being linked with patien	

Healthcare professionals observed that an evidence-based platform that is compatible with us	
Healthcare professionals observe	
Perceptions that most people are	‘most people have phones,
Healthcare professionals felt that a comprehensive platform, which would accommodate user	
Healthcare professionals felt that a comprehensive platform, which would accommodate user	
Healthcare professionals felt that a comprehensive platform, which would accommodate user	
("HP8: ‘not everybody comes in t	if it's too generic, then it won't be good
HCPs suggested that a digital plat	
Healthcare professionals also out	flagging system
Perceptions that most people are	that most people are IT (information technology) literate
Perceptions of users’ digital literacy as a potential barrier, recognising a perceived age-related	
Perceptions of users’ digital literacy as a potential barrier, recognising a perceived age-related	
Healthcare professionals also out	or having your [HP] share the link [with other HPs] - to supp
Healthcare professionals also out	her HPs], where they actually look at what you’ve been doin
Healthcare professionals also outlined advantages of data sharing and being linked with patier	
Healthcare professionals also outlined advantages of data sharing	
Perceptions that most people are	old age not a problem
Perceptions of users’ digital literacy as a potential barrier, recognising a perceived age-related	
Perceptions of users’ digital literacy as a potential barrier, recognising a perceived age-related	
Healthcare professionals also per	
Healthcare professionals observed that an evidence-based platform that is compatible with us	
HCPs suggested that a digital plat	
Healthcare professionals identifie	the Financing/cost of using Salvio
Healthcare professionals observe	into usual care
Healthcare professionals identifie	and the potential disadvantages of using a platform, which i
: ("‘[users] were told that it was a	
.’ ("[users] feeling like they were	
.’ ("[users] feeling like they were being watched, even when we knew they weren’t… [it] just b	
Healthcare professionals observe	timing for platform delivery t
Healthcare professionals also outlined advantages of data sharing and being linked with patier	
Healthcare professionals also outlined advantages of data sharing and being linked with patier	
’. One healthcare professional ref	data sharing
Perceptions of users’ digital literacy as a potential barrier, recognising a perceived age-related	
Perceptions of users’ digital litera	lack of family social support
Healthcare professionals perceive	no privacy concerns
Both users (patients) and healthc	support and guidance
User-friendly device	

Performance tracking and assessm	
Performance tracking and assessment allows for self-management abilities;	
Aversion to tasks or challenging a	could lead to de-motivation
Lack of access to technology afte	
Lack of access to technology afte	
A structured and personalized pro	
Tailored to individual needs and preferences;	
Tailored to individual needs and preferences;	
Tailored to individual needs and preferences;	
Aversion to tasks or challenging a	different physical capabilities
Absence of adaptations personalized to individual needs (limited opportunities to discuss expe	
Incorporating meaningful activitie	
Vulnerable patients (depression,	metnally unstable
Not applicable to patients with ex	extended hospital stays, post-surgery fatigue, and complicat
Addressing the needs, preference	using family as a help
Addressing the needs, preference	
It is a limitation [that the intervention did] not	
Promoting awareness of and motivation for regular physical activity and enhances exercice en	
Overemphasis on physical activiti	lack of mental health focus
Technological difficulties (not suitable for the elderly);	
Not applicable to patients with extended hospital stays, post-surgery fatigue, and complicatio	
Lack of coordination of delivery w	
Contact criteria remained unclear, as the	
Contact criteria remained unclear, as the	
Diminished energy, initiative, and	
Diminished energy, initiative, and	
Promoting awareness of and mot	
Ensuring prompt and timely delivery;	
Commencing exercise promptly after surgery;	
Diminished energy, initiative, and	
Diminished energy, initiative, and	
Uncertainty regarding expectations, responsibilities, and roles;	
Inadequate involvement (conflict	inadequate support
Feasible as a complementary inte	
Prompt and ample support (subsequent phone consultations, text messaging capability, availa	
Insufficient guidance or overwhelming information;	
Insufficient guidance or overwhelming information;	
User-friendly device	

A structured and personalized pro	
Insufficient guidance or overwhelming information;	
Well-defined/manual for delivery	
Constraints on time during interv	
Constraints on time during interv	
Lack of cross-sectoral collaboration;	
Lack of coordination of delivery w	
Insufficient guidance or overwhelming information;	
Unacknowledged workload press	workload pressure
HCPs perceive patient benefit ("P	"Emergency visits will be reduced", 23%; "It is useful for effi
HCPs perceive patient benefit ("P	"Patients can be treated earlier"
HCPs perceive patient benefit ("P	("Patients can receive better care at home through daily mo
HCPs perceive unjustified patient	
HCPs perceive unjustified patient	
Financing/costs for equipment installation (62%);	
[Lack of] Widespread use of elec	EMR specific
No incentives in health insurance	
[Concerns about] Personal inforn	CONCERNS
"It is not realistic because so many problems need to be overcome", 24%)	
Overly complicated system (14%)	complexity
[Poor design of] User interface fo	
[Poor design of] User interface fo	
HCPs' concerns about feasibility of DHI within daily workload/workstreams ("It is difficult for 1	
HCPs' concerns about feasibility of DHI within daily workload/workstreams ("It is difficult for 1	
HCPs' concerns about feasibility o	CONCERNS
; Healthcare providers cannot monitor patients all day long", 35%;	
HCPs' concerns about feasibility of DHI within daily workload/workstreams ("It is difficult for 1	
HCPs' concerns about feasibility of DHI within daily workload/workstreams ("It is difficult for 1	
HCP–participants recommended	educational approach
HCP–participants suggested that	
HCP–participants suggested that	
HCPs had two key provisos: 'user-	
old age and non-use ("The caveat	of tech - We've got some patients who don't use mobile pho
highly tailored intervention facilit	
highly tailored intervention facilit	
An app that patients can modify/	of patients having control over the DI's settings
Participants emphasised the need	Participants emphasised the need for additional features to

old age and non-use ("The caveat	
HCP–participants recommended messages about the benefits of medication or the consequen	
old age and non-use ("The caveat	
Feedback on adherence levels - si	
HCPs had two key provisos: traini	template that could be incorporated in existing computer sy
HCPs talked positively about how	a 5 minute VBI [very brief face to face discussion] componen
their HCP ("If you are having	
HCP–participants suggested that	
HCPs talked positively about how, if done in a non-judgemental way (a 5 minute VBI [very brie	
their HCP ("If you are having	
HCPs had two key provisos: traini	training to help them deliver the VBI within the tight timing
HCPs had two key provisos: training to help them deliver the VBI within the tight timing of 5 m	
HCPs had two key provisos: traini	
Participants emphasised the need	lower the cognitive load needed to understand
Above all, participants emphasise	
Feedback on adherence levels - si	
their HCP ("If you are having	between different pharamcists
time ("I don't think it's [very brief face to face discussion before DI) is going to end at five minu	
Value of fewer clicks for patients	data click-ability (patients)
The cardiologist was given remot	
They considered that Zoom was e	No need to install another software package
They considered that Zoom was e	Fewer steps (less interaction) to get to DHT = better uptake
All clinicians agreed (or strongly a	Improved efficiency in caring for their patient
Incorporating education of the ap	incorporating education of the app meant patients had prior
Burden after that was reduced co	Less burdon now
the HF nurses expressed the need	lack of - need for context info from the patient
Currently, TM puts the HF clinic in	seent to optimize patient therapy
does not support their current de	does not support their decision making process
This makes HF patients feel safe i	sense of safety among patients
Another participant expressed th	worry of patient workload for patients with co-morbidities o
and that using the TM system som	
that the system does not take (ch	
clinicians suggest adaptable TM s	
thereby, clinicians suggest adapta	
by allowing the system to be used	
Complexitiy of HF - HF can manife	HF is a complex condition which may not be possible to addr
Clinicians believe that this should	data patient values

Clinicians believe that the main re	
This suggested the difficulties of r	Limitations of remote - cannot evaluate/test patient properl
They are concerned that current s	
[problem of TM not integrated in	data ehr
clinicians often need to switch be	
One participant mentioned that u	information overlooked
(do not take recommendations fr	
[result of HCPs monitoring] as a c	
Clinicians state that patients ofte	
Clinicians experience that patient	
Then [the patient] has totally mis	
Besides including primary care pr	Patients taking an active role could alleviate some workload
“One reason is for patients to be	patients taking responsibility of their own health should be t
Currently, TM puts the HF clinic in	View/perceived as a tool to support
Due to high false alarm rates, HR	high false alarms led to mistrust of alarms
One participant questioned whet	feeling you cannot monitor HF with the current system
but they also expressed concerns	
Can we treat a patient only on the	
Currently, TM puts the HF clinic in	changed role betwen clinic and TM
“I don’t trust those alarms	
Clinicians described that the inter	too much clicking back and forth to get to info
Clinicians expressed the opportun	divide workload among different HCPs
and they must note the same info	having to input same information across platforms
Experience that workloads did no	Workload didn't decrease
This results in an inefficient and t	
(“Major insurance companies and	relaxed regulations from insurance companies
I had better follow-up encounters on telemedicine	better follow-up compared to before
Perceptions that some patients w	perception that patients were less stressed
Concerns regarding challenges in	dementia patients
Participants cite concerns regardi	private insurance company rules vary by company and by sta
Reimbursement uncertainty - Ove	uncertanity on who should reimburse services
clinicians with less than 15 years’	more years in work related to less readiness
Concerns regarding challenges in	non-English speaking
Concerns regarding challenges in	eldery
Concerns regarding challenges in	rural patients
Concerns regarding challenges in	non-verbal
Older patients ("“Difficulty coordi	older patients not having video capabilities

Technology problems from the pa	
And we should launch randomize	use of RCT to evalute effectiveness
concern for lack of payment for s	CONCERNS
Expectation of their institute to p	
Cannot properly diagnosis a patie	how to test cardiac patients without some back up cardiac te
Cannot properly diagnosis a patie	limited by what can see on video screen and what patient h
Need for integration within an EM	
Administrative barriers ("“Good id	medical council discouragement
About 18% reported malpractice	
90% of all respondents stated tha	willing to provide services from home and use own devices
Not willing to use it ("A combined	not willing to use it regardless of integration
Willingness to use telehealth eve	willing to use DHI even if not integrated
major insurance companies and Medicare have relaxed	reimbursing for telehealth
Participants cite concerns regardi	private insurance company rules vary by company and by sta
On subgroup analyses of practice	EHR requirement in urban more often than rural places
regulatory issues	data regulatory
Reimbursement ("I think it [i]s un	reimbursing is discouraging phone visits
Administrative barriers ("“Good id	ligitation issue
Service issues ("However, with M	data insurance companies
I think that home cardiac rehab reimbursement should be	rapid reimbursement deployment
Perception that telehealth is not s	
concerns of patient interest (54%	
need for staff training	data training
Need for set up prior to the visit (	data prior to appointment
87% stated that non-MDs such as	other HCPs should be able to use telehealth services
Perception of space for	there is space, 'mental space'/prepardness for telehealth ser
HCP perception about patients' d	even for low digital literacy
HCPs' perception of clinical benef	Medly were highlighted to help provide a level of care contin
HCPs' perception of clinical benef	monitor trends in patient heart rate, blood pressure, weight
HCPs unable to conduct full asses	cannot assess their condition correctly
HCPs unable to conduct full asses	cannot assess their condition correctly
HCPs' perception of clinical benef	
HCPs' perception of clinical benef	
HCPs' perception of clinical benef	
HCPs perception that DHI contrib	quality of care was not reduced going virtual
HCPs' concerns about loss of face	less face-to-face contact
HCPs unable to conduct full asses	reduced visual cues

HCPs' concerns about loss of face	poorer clinician-patient relationships;
Poor integration with existing ele	
HCP perception about patients' d	still approriate with those with low digital literacy : unseque
HCPs' perception of clinical benef	continuous monitoring
HCPs unable to conduct full asses	unable to conduct full assessments
HCPs unable to conduct full asses	reduced visual cues
HCPs unable to conduct full asses	reduced visual cues
HCPs unable to conduct full asses	lack of patient body language
Poor integration with existing ele	
Poor integration with existing ele	
Enrolment in the DHI without rec	not being recruited by their doctor
Enrolment in the DHI without rec	not being recruited by their doctor
Increased workload for HCPs due	
Availability of family members/in	
HCPs unable to conduct full asses	less caregiver support over the phone
Enrolment in the DHI without rec	not being recruited by their doctor
HCP perception about patients' d	
HCPs' perception of clinical benefit / benefit for patient management of the DHI ("Although cl	
Availability of a formal technical s	technical support availability
Availability of a formal technical support service for the DHI ("With physical distancing require	
Provision of dedicated staff to support the DHI ("To accommodate the influx of patients during	
Provision of dedicated staff to su	dedicated nursing staff
Provision of dedicated staff to su	nurses - use of nurses
Increased workload for HCPs due	difference in weekend and weekday
Increased workload for HCPs due	patient caseload.
Increased workload for HCPs due	to alerts
HCPs perception that DHI contrib	quality of care was not reduced going virtual
Availability of family members/in	
viewed as a valuable	
making was also highlighted as	
making was also highlighted as	usually make key assessments with in-person visits
them to modify patient care	
viewed as a valuable	
regions were also noted to	
with any kind of pitfalls with rega	gaps in data
can be different for pediatric	
associated with adolescents	disease severity and specific cardiac conditions

associated with adolescents	disease severity and specific cardiac conditions
interaction would also need to	adjusting frequency of interaction
interaction would also need to	
to further incentivize program	
to further incentivize program	
need	adjusting reminder frequency
to further incentivize program	icon size and colors
expressed that often younger	data sharing with or without caregiver
preferences of	modifiable access for the caregivers
if it’s cumbersome,	if there's no meaningful feedback from the HCPs
be language barriers as well. So	
if it’s cumbersome,	
clinician’s felt that the	
care behaviours, clinicians	example of incentivies
care behaviours, clinicians	example of incentivies
felt wary about the benefits	clinical capabilities are limited
of you to make some decisions is	
care behaviours, clinicians	, building incentive for patient usage would be crucial.
care behaviours, clinicians	
clinicians suggested that by	feeback between the two
that to support patient usage,	he digital tool could be linked to the caregiver’s phone.
expressed that often younger	
felt wary about the benefits	clinical capabilities are limited
I think some of it may be just nav	becoming comfortable with the DHT
to read and understand. I’d like	easy to read and understand
the use of patient trends within	
effectiveness of a digital tool,	respective to each patient age range
to read and understand. I’d like	easy to read and understand
an easy-to-understand metric	easy-to-understand metric of
Intervention was facilitated by ph	clearly defined responsibilty roles
and workload impact	data
Concerns with equitable access to	inequitable concerns
and how to ensure the accuracy (	data validating
Challenges with the feasibility of	data patient (individualised medication) needs
Different preferred modes of com	data preferences
Concerns with HCP remuneration	CONCERNS of remuneration
(pharmacists should be primary r	pharamacists should be compensated

(real time monitoring not practic	data workflow
Concerns with privacy of the data	CONCERNS - privacy
Physicans and pharmacists want t	responsibility/roles on patients
Concerns with medicolegal liabilit	liability- concerns with medicolegal liability
Concerns with the credibility of S	credibility
supportive of this intervention	Support for pharamacists to do the intervention
Performance expectancy (β = 0.1	It is helpful for my work.
Performance expectancy (β = 0.1	It can help me obtain patient ECG data at any time.
Performance expectancy (β = 0.1	It can help me diagnose AF.
Performance expectancy (β = 0.1	It can help me screen patients for AF.
Perceived risk	I am worried about patients not being able to cooperate.
social influence (β = 0.404	Media ads (TV ads or Internet ads) recommend it, and I will
price perception (β = 0.585)	cost is reasonable
price perception (β = 0.585)	lowering costs - it does not increase the cost for a patient
social influence (β = 0.404	or higher-level physician, and I will use it.
Perceived risk	I am worried about patients not being able to cooperate.
price perception (β = 0.585)	In terms of health, it is worth it.
social influence (β = 0.405	The guideline recommends it, and I will use it.
Perceived risk	I am concerned about the possible disclosure of patient priv
social influence (β = 0.403	It is recommended by a peer
social influence (β = 0.403	It is recommended by a peer
social influence (β = 0.406	Other community health facilities have used it, and I will use
Perceived risk	I am worried that the results will not be accurate.
can enter all the patient history	
accessing personal information	
accessibility,	
accessible education	
consuming as reported by a CRx	
consuming as reported by a CRx	entering information about patients
data in the application was	
data in the application was	
anyone can handle. No tricky	
recommended adding more	
bilingual flexibilty	
gather the information so it will	score
data in the application was	manually input patient data- lack of automation
this theme	

[illegible] regarded as having significant	
willness to use	
[illegible] people. It should be beneficial”	
[illegible] people. It should be beneficial”	motivation to recommend to others
[illegible] information was regarded as	
mostly comprehensive	not fully comprehensive
[illegible] and menus and content of	
[illegible] personal information and	
[illegible] application	
interactive	
[illegible] and menus and content of	it is easy to find each icons and menus
[illegible] anyone can handle. No tricky	
[illegible] application	
[illegible] gather the information so it will	
[illegible] beginning is hard, but when you	of content

Minor code	Major code
Adherence	Clinical benefit
Access to internet/WIFI	Accessibility
Access to mobile phone	Accessibility
Access to mobile phone	Accessibility
	Adaptability
Multiple users	Adaptability
Old age	Appropriate
Long-term sustainability	Financing/cost
Internet/WIFI	ICT infrastructure
Internet/WIFI	ICT infrastructure
Workflow	Integration
Documentation	Mis (use)
Electricity	Power and energy infrastructure
Training	Training/Educating
Interaction (patient)	Usability
Documentation	Workload
Access to patient data	Accessibility
Management	clinical benefit
HCP Speciality	Adaptability
Digital literacy	Capability
Technical issues	Technical stability
Internet/WIFI	ICT infrastructure
Planned use	Intention to use
Privacy	Privacy
Not first-encounter	Technology attitudes
Comfortable	Technology attitudes
Video visits	Type
Phone visits	Type
unclear	Usability
Accuracy	Clinical benefit
Access to patient data	Accessibility
Alerting	Automation
Alerting	Automation
Feedback	Communication
Workflow	Integration

EHR	Integration
Standardization	Policies and laws
Legal factors	Policies and laws
privacy	Privacy
confidentiality	Privacy
Transparency	Privacy
Easy/quick	Visualization
Trends	Visualization
Graphical representation	Visualization
Health outcomes	Clinical benefit
Efficiency	Clinical benefit
Cognitive load	Health worker benefit
Personal gain	Mis (use)
Access to care	Accessibility
Equitable access to care	Accessibilty
Medical history	Appropriate
Health literacy	Capability
Technical issues	Technical stability
Internet/WIFI	ICT infrastructure
Ignoring	Mis (use)
Improper	Mis (use)
(Over)reliance	Mis(use)
Medical information	Misunderstanding
perceived value	Motivation
lack of services in clinics	Physical space/infrastructure
Personal privacy	Privacy
liability	role/responsibilities
HCP Gender preference	sociocultural
Fear	Technology attitudes
reluctance/resistance	Technology attitudes
Addition / not substitution	Technology attitudes
Not first-encounter	Technology attitudes
Tool	Technology attitudes
HCP expertise	Trustworthiness
HCP expertise	Trustworthiness
socioculturall	work infrastructure
Resources	Work infrastructure

opening hours	work infrastructure
Time	Work infrastructure
Management	clinical benefit
retention	clinical benefit
Evidence-based	Evidence-based
Access to technology	Accessibility
Accuracy	Accuracy of DHI content
Guidelines	Adaptability
Personalized	Adaptability
Language	Adaptability
Personalized	Adaptability
Calculation	Automation
Digital literacy	Capability
contextual information	Content
Old age	Demographics
Education	Demographics
Evidence-based	Evidence-based
Reminders (holistic)	Feedback
Official endorsement	Policies and laws
Personal privacy	Privacy
Privacy	Privacy
Money-making	Technology attitudes
Training	Training/Educating
Training	Training/Educating
Accuracy of health information	Trustworthiness
Accuracy	Trustworthiness
Organization	Trustworthiness
HCP approved	Trustworthiness
Accuracy	Trustworthiness
Simplicity (patients)	Usability
Ease-of-data entry	Usability
Organization	Usability
Complexity (patients)	Usability
Simplicity (patients)	Usability
Graphical representation	Visualization
Color	Visualization
Summarizing	Visualization

Data	Visualization
Amount/readability (patients)	Visualization
Boredom	Motivation
cognitive load	workload
Perceived value	Motivation
ignoring	Mis (use)
Skipping features	Mis (use)
	Motivation
Engaging	Motivation
Perceived value	Motivation
Irritation	Technology attitudes
	Technology attitudes
Explanation	Training/Educating
Training	Training/Educating
Training patient	Training/Educating
Educating	Training/Educating
Graphical representation	Visualization
Quality-of-care	Clinical benefit
Shared-decision making	Patient-focused care
Tailored care	Patient-focused care
Tailored care	Patient-focused care
Shared-decision making	Patient-focused care
Discussion	Patient-HCP
Communication	Patient-HCP
Time	Workload
high-risk	Demographics
Access to care	Accessibility
Self-management	Patient empowerment
Tailored care	Patient-focused care
self-management	Patient empowerment
Access to equipment	Accessibility
Health concern/problem	Adaptability
Filling-in	Automation
Alerting	Automation
Alerting	Automation
self-management	Content
Evidence-based	Evidence-based

	Financing/cost
Equipment	Financing/cost
(Over)reliance	Mis(use)
(Over)reliance	Mis(use)
Perceived value	Motivation
	Need
Guidelines	Policies and laws
Personal privacy	Privacy
Liability	Role/responsibilities
Educating	Training/educating
Trends	Visualization
Easy/quick	Visualization
Guidelines/protocols	work infrastructure
Time	Workload
Health outcomes	Clinical benefit
Responding	Workload
Interaction	Workload
Efficiency	Clinical benefit
Staff compensation	Financing/cost
Hiring	Financing/cost
Workflow	Integration
Practical problem	Need
Reimbursement	Policies and laws
(perceived) positive perceptions	Technology attitudes
Training	Training/Educating
Training	Training/educating
User-friendly	Usability
NPHW	work infrastructure
Supportive	work infrastructure
NPHW	work infrastructure
Defined roles	work infrastructure
Time	Workload
Rapport	Patient-HCP
Time	Workload
Self-management	Patient empowerment
Access to mobile phone	Accessibility
Digital literacy	Capability

Technical issues	Technical stability
Addition / not substitution	Technology attitudes
Tool	Technology attitudes
Quality-of-care	Clinical benefit
Health outcomes	Clinical benefit
Efficiency	Clinical benefit
Identification	Clinical benefit
Treatment	Clinical benefit
Accuracy	Clinical benefit
Efficiency	Clinical benefit
Implementation	Workload
Reviewing	Workload
Time	Workload
User-friendly	Usability
Health literacy	Capability
Literacy	Capability
Male	Demographics
Internet/WIFI	ICT infrastructure
Empowerment	Motivation
Positive reception	Motivation
Self-management	Patient empowerment
Access to patient data	Accessibility
Tailored care	Patient-focused care
Productive	Patient-focused care
Data selection	Autonomy
Technical issues	Technical stability
Systems	ICT infrastructure
Existing systems	Integration
incentivization	Motivation
	patient safety
Increased responsibility	Role/responsibilities
Shifted roles	Role/responsibilities
Training	Training/Educating
Trends	Visualization
Graphical representation	Visualization
Summarizing	Visualization
supportive	work infrastructure

NPHW	work infrastructure
Team-based	work infrastructure
centralization	work infrastructure
Defined roles	work infrastructure
Interaction	Workload
Efficiency	Clinical benefit
Management	Clinical benefit
Comprehensive	Clinical benefit
Evaluation	Clinical benefit
Efficiency	Clinical benefit
Rapport	Patient-HCP
Rapport	Patient-HCP
Time	Workload
Time	Workload
Reviewing	Workload
Follow-up	Workload
Technical support	Workload
Reviewing	Workload
Follow-up	Workload
Interaction	Workload
Access to care (rural areas)	Accessibility
Access to equipment	Accessibility
Medical history	Appropriate
Digital literacy	Capability
Digital literacy	Capability
Non-native speaker	Demographics
Old age	Demographics
Socioeconomic status	Demographics
Patient home (distracting)	Environment
Internet/WIFI	ICT infrastructure
Listening	Mis (use)
Listening	Mis (use)
Preparedness	Mis (use)
Expectations	Misunderstanding
Value	Technology attitudes
Addition / not substitution	Technology attitudes
Not first-encounter	Technology attitudes

Staffing (expertise)	work infrastructure
Scheduling	work infrastructure
Efficiency	clinical benefit
Access to care (for sick)	Accessibility
Access to care	Accessibility
Waiting time	Workload
Quality-of-care	Clinical benefit
Outliers	Adaptability
	Autonomy
Patient outliers	Autonomy
Disagreement (among HCPs)	Collaboration
Workflow	Integration
Workflow	Integration
Ignoring	Mis (use)
Improper	Mis (use)
Fidelity	Mis (use)
Peer pressure	Social influence
Disagreement	Technology attitudes
Cost-effectiveness	Clinical benefit
Efficiency	Clinical benefit
Efficiency	Clinical benefit
Standardization	Clinical benefit
Quality-of-care	Clinical benefit
Satisfaction	Psychological
Safety	Patient safety
Perceived value	Motivation
Peer pressure	Social influence
Reluctance/resistance	Technology attitudes
Conservative	Technology attitudes
self-management	Patient empowerment
Health concern/problem	Adapatability
Evidence-based	Evidence-based
Hospitalization	Financing/cost
Staff compoensation	Financing/cost
workflow	Integration
Guidelines	Policies and laws
NPHW	work infrastructure

health outcome	clinical benefit
Cost-effectiveness	Clinical benefit
Worker protection	Health worker benefit
Safety	Patient safety
clinical safety	Patient safety
Responding	Workload
Access to mobile phone	Accessibility
Literacy	Capability
Digital literacy	Capability
Old age	Demographics
Rural or remote areas	Demographics
Evidence-based	Evidence-based
Tech	Financing/cost
Tech	Financing/cost
Recording system	ICT infrastructure
Commitment	Leadership
Designated branch	Policies and laws
multisectoral collaboration	Policies and laws
Health system	Trustworthiness
Staffing (expertise)	work infrastructure
Time	Workload
Adherence	Clinical benefit
Access to care (rural areas)	Accessibility
Time	Workload
Documentation	Workload
Anxiety	Psychological
Evidence-based	Evidence-based
Validating	Accuracy of DHI content
Evidence-based	Evidence-based
Evidence-based	Evidence-based
Evidence-based	Evidence-based
Evidence-based	Evidence-based
Regulations	Policies and laws
Health outcome	Clinical benefit
Reassurance	Patient-focused care
Self-management	Patient empowerment
Patient needs	Adaptability

	Adaptability
	Automation
HCP responsibility	Autonomy
With patients	Co-design
With HCPs	Co-design
security	Data
Evidence-based	Evidence-based
Data management system	ICT infrastructure
Workflow	Integration
Perceived value	Motivation
	Patient safety
Family	Social support
Sceptical (ability)	Technology attitudes
Reluctance/resistance	Technology attitudes
Threating (prioritized)	Technology attitudes
Tool	Technology attitudes
Reluctance/resistance	Technology attitudes
Sceptical (accuracy)	Technology attitudes
Accuracy	Trustworthiness
DHT ability	Trustworthiness
DHT ability	Trustworthiness
staffing	work infrastructure
Staffing (expertise)	work infrastructure
Efficiency	Clinical benefit
Documentation	Clinical benefit
Data collection	Clinical benefit
Efficiency	clinical benefit
Accuracy	Clinical benefit
Reducing error rate	Clinical benefit
Health literacy	Capability
Productive	Patient-focused care
Patient privacy	Privacy
Digital literacy	Capability
Technical issues	Technical stability
Reimbursement	Financing/cost
Perceived value	Motivation
Side effects	Patient safety

Content	Trustworthiness
Access to care	Accessiblity
Program champions (HCPs)	Leadership
Medical history	Appropriate
Digital literacy	Capability
Digital literacy	Capability
Among HCPs	Communication
Mental/physical fitness	Demographics
Technical issues	Technical stability
Evidence-based	Evidence-based
Internet/WIFI	ICT infrastructure
Computers	ICT infrastructure
Authority	Leadership
Perceived value	Motivation
	Motivation
	Motivation
Peer pressure	Social influence
Peer pressure	Social influence
Peer pressure	Social influence
Explanation	Training/Educating
Training	Training/Educating
Scheduling	work infrastructure
Organization	work infrastructure
Supportive (within clinic)	work infrastructure
Delineating Responsibilities/roles	work infrastructure
Organization	work infrastructure
Contact person	work infrastructure
NPHW	work infrastructure
Time	Workload
Among different disciplines	Collaboration
Access to care	Accessibility
Internet/WIFI	ICT infrastructure
Space	Physical space/infrastructure
Accuracy of health information	Trustworthiness
staffing	work infrastructure
staffing	work infrastructure
Scheduling	work infrastructure

Access to (health) resources	Accessibility
Rural or remote areas	Accessibilty
	Clinical benefit
Technical issues	Technical stability
Pairing	Technical stability
Pairing	Technical stability
	Financing/cost
	Financing/cost
Workflow	Integration
Expectations	Misunderstanding
Setting expectations	Process
Setting expectations	Process
[illegible]	Training/Educating
[illegible]	Training/Educating
Accuracy	Trustworthiness
time	work infrastructure
Resources	Work infrastructure
Time	work infrastructure
Efficiency	Clinical benefit
adherence	Clinical benefit
Rapport	Patient-HCP
Technical support	Workload
Implementation	Workload
Reviewing	Workload
Follow-up	Workload
Efficiency	Clinical benefit
Access to patient data	Accessibility
Access to mobile phone	Accessibility
Access to mobile phone	Accessibility
Accuracy	Accuracy of DHI content
Accuracy	Accuracy of DHI content
(under)alerting	Alerting accuracy
Calculation	Automation
With HCPs	Co-design
Workflow	Integration
Workflow	Integration
EHR	Integration

EHR	Integration
EHR	Integration
EHR	Integration
Documentation	Mis (use)
Improper	Mis (use)
Information overlooked	Mis (use)
(Over)reliance	Mis(use)
Reluctance/resistance	Technology attitudes
Works	Technology attitudes
patient responses	Trustworthiness
Accuracy	Trustworthiness
Hybrid	Type
Convenience (patients)	Usability
Simplicity (patients)	Usability
User-friendly for patient	Usability
Simplicity (patients)	Usability
Simplicity (patients)	Usability
Graphical representation	Visualization
Color	Visualization
Graphical representation	Visualization
Amount/readability (patients)	Visualization
Burden	Workload
Access to patient data	Accessibility
Boredom	Motivation
Burden	Workload
Medical decision making	Clinical benefit
Accuracy	Clinical benefit
Management	Clinical benefit
Adherence	Clinical benefit
Data sharing	Patient-HCP
Data sharing	Patient-HCP
Time	Workload
Documentation	Workload
Interaction	Workload
[illegible] participants believed	Health worker benefit
	Patient-focused care
Familiarity	Experiences

Past habits	Experiences
	Workload
Increased responsibility	Role/responsibilities
Digital literacy	Capability
Health literacy	Capability
Storage	Data
Sharing	Data
Technical issues	Technical stability
Evidence-based	Evidence-based
Usage	Experiences
Familiarity	Experiences
Staff compensation	Financing/cost
	Financing/cost
	Financing/cost
EHR	Integration
Reimbursement	Policies and laws
Privacy	Privacy
Patient privacy	Privacy
Interest	Technology attitudes
Training	Training/educating
Educating	Training/Educating
User-friendly	Usability
Guidelines/protocols	Work infrastructure
Time	Workload
Type of exercise	Content
Literacy levels	Adaptability
Health literacy	Capability
Reminders	Feedback
(Over)reliance	Mis (use)
(Over)reliance	Mis(use)
	Motivation
Communication	Patient-HCP
Setting expectations	Process
Setting expectations	Process
HCPs	Social support
(perceived) positive perceptions	Technology attitudes
Explanation (patients)	Training/Educating

Design	Usability
Summarizing	Visualization
Trends	Visualization
Communication	Patient-HCP
Insurance companies (teamwork)	Policies and laws
Access to care	Accessibility
Tailored care	Patient-focused care
Patients	Social support
Social interaction	Social support
	Motivation
access to care	Accessibility
Retention	Clinical benefit
Access to internet/Wifi	Accessibility
Access to equipment	Accessibility
Email	Accessibility
Internet/WIFI	Accessibility
Digital literacy	Capability
Contextual information	Content
Mental health	Content
Funding	Financing/cost
Non-verbal	Human aspect
Clinical testing	Human aspect
Capacity	ICT infrastructure
Capacity	ICT infrastructure
Space	Physical space/infrastructure
Insurance companies	Policies and laws
Regulations	Policies and laws
Family	Social support
Patients	Social support
Threating	Technology attitudes
Organization	Trustworthiness
Capacity	work infrastructure
Responding	Workload
Documentation	Workload
Documentation	Workload
Capacity	Workload
Time	Workload

EHR	Integration
Access to patient data	Accessibility
Language	Adaptability
Digital literacy	Capability
Among HCPs	Collaboration/connectivity
Touch	Human aspect
Privacy	Privacy
confidentiality	Privacy
Cost-effectiveness	Clinical benefit
	Patient empowerment
Reassurance	Patient-focused care
Anxiety	Psychological
Responding	Workload
Reassurance	Patient-focused care
Accuracy	Accuracy of DHI content
Accuracy	Accuracy of DHI content
Health literacy	Appropriate
Accuracy	Clinical benefit
social support	Appropriate
Comprehensive	Clinical benefit
Accuracy	Clinical benefit
Health literacy	Capability
Feedback	Communication
Quality-of-care	Clinical benefit
One-way	Communication
Systems	Communication
Contextual information	Content
Sensory constraints	Demographics
Quality-of-care	Clinical benefit
Non-verbal	Human aspect
Visual cues	Human aspect
Visual cues	Human aspect
Listening	Mis (use)
Documentation	Mis (use)
Forgetting	Mis (use)
Ignoring	Mis (use)
Communication	Misunderstanding

Communication	Misunderstanding
Communication	Misunderstanding
	Misunderstanding
Expectations	Misunderstanding
Expectations	Misunderstanding
	Patient safety
Rapport	Patient-HCP
Trust	Patient-HCP
Setting expectations	Process
Liability	Role/responsibilities
Patient contact responsibility	Role/responsibilities
	Social support
Reluctance/resistance	Technology attitudes
Accuracy	Trustworthiness
DHT ability	Trustworthiness
HCP expertise	Trustworthiness
DHT ability	Trustworthiness
Accuracy	Trustworthiness
DHT ability	Trustworthiness
Accuracy	Trustworthiness
Accuracy	Trustworthiness
Data	Visualization
Interaction	Patient-HCP
Trust	Patient-HCP
Rapport	Patient-HCP
Stress	Psychological
Rapport	Patient-HCP
Patient understanding	Patient-HCP
Simplicity/efficiency (care)	Patient-focused care
Time	Workload
Organization	Trustworthiness
Interaction	Workload
User-friendly	Usability
	Appropriate
Digital literacy	Capability
Technical issues	Technical stability
Technical issues	Technical stability

Technical issues	Technical stability
	Financing/cost
Funding	Financing/cost
Systems	ICT infrastructure
Connectivity	ICT infrastructure
	Leadership
Forgetting	Mis (use)
	Motivation
Regulations	Policies and laws
Device regulations	Policies and laws
Legal factors	Policies and laws
Staffing (expertise)	work infrastructure
NPHW	work infrastructure
staffing	work infrastructure
Time	work infrastructure
Cross-sectional collaboration	work infrastructure
Liability	work infrastructure
Time	Workload
Juggling tasks	Workload
Time	Workload
Implementation	Workload
Decision-making	Patient-HCP
Evidence-based	clinical benefit
Patient understanding	Patient-HCP
Shared-decision making	Patient-focused care
Satisfaction	Psychological
Shared-decision making	Patient-focused care
Time	Workload
Efficiency	Clinical benefit
Shared-decision making	[illegible]
Addition / not substitution	Technology attitudes
Access to (health) resources	Accessibility
Access to technology	Accessibility
Access to mobile phone	Accessibility
Access to technology	Accessibility
Access to mobile phone	Accessibility
Reliability	Accuracy of DHI content

Language	Adaptability
Digital literacy	Appropriate
Selective use	Autonomy
Repeat	Autonomy
Time/location	Autonomy
Selective use	Autonomy
Health	Autonomy
Selective use	Autonomy
Health literacy	Capability
Digital literacy	Capability
One-way	Communication
One-way	Communication
Quality checks (DHT)	Content
Non-native speaker	Demographics
Old age	Demographics
Evidence-based	Evidence-based
Familiarity	Experiences
Familiarity	Experiences
Visual cues	Human aspect
Workflow	Integration
Multiple software	Integration
Authority	Leadership
Authority	Leadership
Authority	Leadership
Skipping features	Mis (use)
Information overlooked	Mis (use)
	Misunderstanding
	Motivation
	Motivation
Want	Motivation
Willingness	Motivation
	Patient buy-in
Communication (messaging)	Patient-HCP
Patient confidentially	Privacy
During consultation	Process
Timely introduction	Process
Timely introduction	Process

HCPs	Social influence
Society	Social/economic influence
Value	Technology attitudes
Sceptical (general)	Technology attitudes
Tool	Technology attitudes
(perceived) positive perceptions	Technology attitudes
Concerns	Technology attitudes
(perceived) positive perceptions	Technology attitudes
Tool	Technology attitudes
Educating	Training/Educating
Training	Training/Educating
Materials	Training/Educating
Continuous	Training/Educating
Familiarity	Training/Educating
Testing	Training/Educating
Training patient	Training/Educating
Hybrid	Type
user-friendliness	Usability
unclear	Usability
Convenience	Usability
User-friendly for patient	Usability
User-friendly	Usability
Simplicity	Usability
Simplicity	Usability
Design	Usability
User-friendly	Usability
Data	Visualization
Staffing	Work infrastructure
Contact person	work infrastructure
Staffing (expertise)	work infrastructure
Contact person	work infrastructure
Supportive (within clinic)	work infrastructure
Time	work infrastructure
Workflow	work infrastructure
Time	work infrastructure
Staffing (expertise)	work infrastructure
Adherence	Clinical benefit

Efficiency	Clinical benefit
Discussion	Patient-HCP
Social interaction	Patient-HCP
Interaction	Patient-HCP
Patient understanding	Patient-HCP
Interaction	Patient-HCP
Access to care	Accessibility
Access to (health) resources	Accessibility
Health literacy	Capability
Sustainability of BC	Patient empowerment
Simplicity/efficiency (care)	Patient-focused care
Shared-decision making	Patient-focused care
Productive	Patient-focused care
Self-efficacy	Patient empowerment
Patients	Social support
Social interaction	Social support
Equitable access to care	Accessibility
Stress	Psychological
	Workload
Cross-sectional collaboration	work infrastructure
Reducing error rate	clinical benefit
Electronic communication devices	ICT infrastructure
Authority	Leadership
	Staff buy-in
Concerns	Technology attitudes
Concerns	Technology attitudes
Accuracy	Trustworthiness
Supportive (within clinic)	work infrastructure
Guidelines/protocols	work infrastructure
NPHW	work infrastructure
Defined roles	work infrastructure
Staffing (expertise)	work infrastructure
Supportive (within clinic)	work infrastructure
Interaction	Patient-HCP
Private practice	Demographics
Specialist	Demographics
Accuracy	Trustworthiness

DHT ability	Trustworthiness
Planned use	Intention to use
Medical decision making	clinical benefit
Evidence-based	Evidence-based
Evidence-based	Evidence-based
Interpretation of data	Visualization
Hospital approved	Trustworthiness
Liability	Policies and laws
Management	clinical benefit
	Accuracy of DHI content
Reimbursement	Awareness
Evidence-based	Evidence-based
Staff compensation	Financing/cost
EHR	Integration
Planned use	Intention to use
Reimbursement	Policies and laws
Data	Visualization
Staffing (expertise)	Work infrastructure
Time	Workload
Burden	Workload
Time	Workload
Burden	Workload
Patient file	Integration
Adherence	Clinical benefit
IT personnel	Co-design
Health management information sy	ICT infrastructure
Workflow	Integration
EHR	Integration
Program champions (HCPs)	Leadership
Liability	Role/responsibilities
Data	Trustworthiness
Data	Visualization
Summarizing	Visualization
Workflow	work infrastructure
Guidelines/protocols	work infrastructure
Staffing (expertise)	work infrastructure
Health literacy	Capability

Health literacy	Capability
Interaction	Workload
Burden	Workload
Support	Patient-focused care
Adherence	Clinical benefit
Access to equipment	Accessibility
Efficiency	Accuracy of DHI content
Filling-in	Automation
Adherence	Clinical benefit
Accuracy	Clinical benefit
Calculation	Automation
Diagnosis	Clinical benefit
Where (purchase)	Awareness
Health literacy	Capability
Digital literacy	Capability
Feedback	Communication
communication channels	Communication
Contextual information	Content
Continuous collection	Data
Continuous	Data
Socioeconomic status	Demographics
Treatment	Clinical benefit
Reminders	Feedback
Reminders	Feedback
	Financing/cost
Workflow	Integration
Workflow	Integration
Workflow	Integration
Medical decision making	Clinical benefit
EHR	Integration
Program champions (HCPs)	Leadership
Comprehensive	Clinical benefit
Efficiency	Clinical benefit
Quality-of-care	Clinical benefit
Information overlooked	Mis (use)
Documentation	Mis (use)
Improper	Mis (use)

Information overlooked	Mis (use)
(Over)reliance	Mis(use)
Engaging	Motivation
	Motivation
Perceived value	Motivation
Self-management	Motivation
	Patient safety
Interaction	Patient-HCP
Communication	Patient-HCP
Insurance companies	Policies and laws
Burden	Psychological
Interest	Technology attitudes
Educating	Training/Educating
Materials	Training/Educating
Training	Training/Educating
Training	Training/Educating
Patient ability	Trustworthiness
Accuracy	Trustworthiness
Accuracy	Trustworthiness
Patient ability	Trustworthiness
Reliability	Trustworthiness
Simplicity	Usability
Simplicity (patients)	Usability
User-friendly for patient	Usability
User-friendly	Usability
Summarizing	Visualization
Summarizing	Visualization
Data	Visualization
Time	work infrastructure
Staffing (expertise)	work infrastructure
Staffing	work infrastructure
Designated group	work infrastructure
Time	Workload
Rapport	Patient-HCP
Interaction	Patient-HCP
Self-management	Patient empowerment
Self-management	Patient empowerment

	Motivation
Health literacy	Capability
Simplicity/efficiency (care)	Patient-focused care
Access to care	Accessibility
Stress	Psychological
Anxiety	Psychological
Stress	Psychological
Shared-decision making	Patient-focused care
Time	Workload
Burden	Workload
Documentation	Workload
Documentation	Workload
Accuracy	Accuracy of DHI content
Access to patient data	Accessibility
Accuracy	Accuracy of DHI content
Several parameters	Adaptability
Patient needs	Adaptability
Patient need perceptions	Autonomy
Alert setting	Autonomy
Digital literacy	Capability
Continuous collection	Data
Old age	Demographics
technical issues	Technical stability
	Ecological-friendliness
	Communication
budgetary allocations	Financing/cost
Listening	Mis (use)
side effects	Patient safety
Explanation	Training/Educating
Comfort (patients)	Usability
Comfort (patients)	Usability
Interpretation of data	Visualization
Data	Visualization
Anxiety	Psychological
Access to care (rural areas)	Accessibility
Attendance/enrolment	Clinical benefit
Quality-of-care	Clinical benefit

quality-of-care	Clinical benefit
Communication impediments	Adaptability
patient needs	Adaptability
language	Adaptability
Old age	Appropriate
Touch	Human aspect
Training	Training/Educating
among HCPs	Communication
Access to care (for sick)	Accessibility
Relationship	Patient-HCP
Trust	Patient-HCP
Patient needs	Adaptability
Patient abilities	Adaptability
Workflow	Integration
HCP reassurance	Patient-HCP
HCP expertise	Trustworthiness
HCP expertise	Trustworthiness
Time	Workload
Other tasks	Workload
Family	Social support
Adherence	Clinical benefit
Access to mobile phone	Accessibility
Access to technology	Accessibility
Digital literacy	Capability
health literacy	Capability
old age	demographics
Technical issues	Technical stability
Competing Financing/costs	Financing/cost
	Financing/cost
Internet/WIFI	ICT infrastructure
Truthfulness	Mis (use)
Limited understanding	Misunderstanding
Health worker benefit	Motivation
	Motivation
	Perceived need
Standardization (training)	Policies and laws
Shifted roles	role/responsibilities

	Social support
Preferences	Technology attitudes
Technical support	Workload
health literacy	Capability
Self-management	Patient empowerment
Self-management	Patient empowerment
Health literacy	Capability
Documentation	Workload
Productive	Patient-focused care
access to care	Accessibility
Reassurance	Patient-focused care
Health outcome	Clinical benefit
Adherence	Clinical benefit
efficiency	Clinical benefit
Optimizing	Clinical benefit
among HCPs	Collaboration
Sensitivity	Accuracy of DHI content
Patient needs	Adaptability
Personalized	Adaptability
Incorporation (activities)	Adaptability
Medical history	Appropriate
Alerting (patients)	Automation
Filling-in	Automation
Alerting (patients)	Automation
Alert setting	Autonomy
Digital literacy	Capability
Digital literacy	Capability
Feedback	Communication
self-management	Content
Sharing	Data
old age	Demographics
old age	Demographics
Feedback	Communication
Staff compensation	Financing/cost
Connectivity	Integration
Boredom	Motivation
Communication	Patient-HCP

Timely introduction	Process
Family	Social support
Family	Social support
Time patient	Training/Educating
Training	Training/educating
Accuracy	Trustworthiness
Convenience (patients)	Usability
User-friendly for patient	Usability
User-friendly for patient	Usability
Graphical representation	Visualization
NPHW	work infrastructure
Team-based	work infrastructure
Team-based	work infrastructure
Time	Workload
	Workload
Documentation	Workload
Documentation	clinical benefit
Innovation	clinical benefit
Efficiency	clinical benefit
Financing/cost-effectiveness	clinical benefit
Adherence	Clinical benefit
Continuity-of-care	Clinical benefit
Among HCPs	Communication
two-way	communication
Evidence-based	Evidence-based
Funding	Financing/cost
Long-term sustainability	Financing/cost
Other apps	Interoperability
Digital systems	Interoperability
Communication	Patient-HCP
Patient confidentially	Privacy
Reluctance/resistance	Technology attitudes
Guidelines/protocols	work infrastructure
Self-management	Patient empowerment
Health literacy	Capability
Access to care	Accessibility
Access to (health) resources	Accessibility

Equitable access to care	Accessibility
Equitable access to technology	Accessibility
Equitable access to care	Accessibility
Communication	Patient-HCP
communication	Patient-HCP
	Workload
Reimbursement	Financing/cost
Management	Clinical benefit
Treatment	Clinical benefit
Comprehensive	Clinical benefit
Diagnosis	Clinical benefit
Accuracy	Clinical benefit
	Accuracy of DHI content
Workflow	Adaptability
Digital literacy	Capability
Old age	demographics
Evidence-based	Evidence-based
Evidence-based	Evidence-based
Evidence-based	Evidence-based
	Financing/cost
(loaning) equipment	Financing/cost
equipment	Financing/cost
Financial incentives	Motivation
Optimism	Motivation
Optimism	Motivation
Financial incentives	Motivation
Established	Physical space/infrastructure
Guidelines	policies and laws
Insurance companies	Policies and laws
Insurance companies	Policies and laws
Assigned	Role/responsibilities
NPHW	Work infrastructure
NPHW	Work infrastructure
Assigned	Role/responsibilities
NPHW	Work infrastructure
Family	social support
Concerns	Technology attitudes

training	training/educating
Accuracy	Trustworthiness
patient ability	Trustworthiness
HCP expertise	Trustworthiness
Patient portals	Type
sharing	work infrastructure
Workflow	work infrastructure
Workflow	work infrastructure
Reviewing	Workload
stress	Psychological
stress	Psychological
Negative emotions	Psychological
Stress	Psychological
Familiarity	Experiences
User-friendly	Usability
Access to patient data	Accessibility
Access to patient data	Accessibility
Access to patient data	Accessibility
Access to care	Accessibility
Productive	Patient-focused care
Health outcome	Clinical benefit
Management	Clinical benefit
efficiency	Clinical benefit
Health outcome	Clinical benefit
Documentation	Clinical benefit
Health outcome	Clinical benefit
Health outcome	Clinical benefit
Optimizing	Clinical benefit
among HCPs	Communication
Access to patient data	Accessibility
Accuracy	Accuracy of DHI content
Health concern/problem	Adaptability
Health concern/problem	Adaptability
(over)alerting	Alerting accuracy
With HCPs	Co-design
Contextual information	Content
Mental health	Content

Past habits	Experiences
Past habits	Experiences
Workflow	Integration
workflow	Integration
Workflow	Integration
(Over)reliance	Mis(use)
Misinterpret	Misunderstanding
Supportive	Motivation
Positive reception	Motivation
Perceived value	Motivation
Perceived value	Motivation
perceived value	Motivation
Supportive	Motivation
	Need
	Patient buy-in
Patient understanding	Patient-HCP
workflow	Integration
workflow	Integration
Clinical duties	Alignment
sharing roles	work infrastructure
Sharing	Role/responsibilities
Reduced shared responsibility	Role/responsibilities
Informal caregiver	Social support
Perceived patient view (sick)	Technology attitudes
Explanation	Training/Educating
Summarizing	Visualization
Easy/quick	Visualization
Color	Visualization
Delineating Responsibilities/roles	work infrastructure
Staffing (expertise)	work infrastructure
	Workload
safety	Patient safety
Communication	Patient-HCP
Time	Workload
Hospital visits	Workload
	Workload
Documentation	Workload

Implementation	Workload
Standardization	work infrastructure
Efficiency	Clinical benefit
Timely response	Accuracy of DHI content
Digital literacy	Appropriate
Alerting	Automation
Alerting	Automation
Alerting (patients)	Automation
Alert setting	Autonomy
Digital literacy	Capability
Among HCPs	Collaboration
Real-time	Data
Staff compensation	Financing/cost
Recording system	ICT infrastructure
EHR	Integration
Existing systems	Integration
	Patient buy-in
Simplicity (patients)	Usability
User-friendly for patient	Usability
Simplicity (patients)	Usability
Interaction (patient)	Usability
Team-based	work infrastructure
Guidelines/protocols	work infrastructure
Cross-sectional collaboration	work infrastructure
Sharing	work infrastructure
Supportive (within clinic)	work infrastructure
Device regulations	work infrastructure
Communication	Patient-HCP
Discussion	Patient-HCP
Responsiveness	Patient-focused care
Simplicity/efficiency (care)	Patient-focused care
Responsiveness	Patient-focused care
Routine check-ups	Technology attitudes
Efficiency	Clinical benefit
Comprehensive	Clinical benefit
Identification	Clinical benefit
Diagnosis	Clinical benefit

Quality-of-care	Clinical benefit
Medical history	Appropriate
Medical history	Appropriate
Familiarity	Experiences
Visual cues	Human aspect
Non-verbal	Human aspect
non-verbal cues	Human aspect
Non-verbal	Human aspect
Clinical testing	Human aspect
Information overlooked	Mis (use)
Perceived value	Motivation
Rapport	Patient-HCP
Discussion	Patient-HCP
Not first-encounter	Technology attitudes
Comfortable	Technology attitudes
Concerns	Technology attitudes
Concerns	Technology attitudes
In-person preference	Technology attitudes
Patient responses	Trustworthiness
HCP expertise	Trustworthiness
Rapport	Patient-HCP
Rapport	Patient-HCP
Interaction	Patient-HCP
Communication	Patient-HCP
Patient understanding	Patient-HCP
Communication	Patient-HCP
Communication	Patient-HCP
Burden	Psychological
Satisfaction	Psychological
Access to care	Accessibility
Access to care (rural areas)	Accessibility
Simplicity/efficiency (care)	Patient-focused care
Equitable access to care	Accessibility
Access to care	Accessibility
Productive	Patient-focused care
Productive	Patient-focused care
Negative emotions	Psychological

Reassurance	Patient-focused care
Reassurance	Patient-focused care
Time	Workload
Burden	Workload
Patient need perceptions	Autonomy
Personalized	Adaptability
Health concern/problem	Adaptability
Size	Adaptability
	Advertising
Alerting	Automation
Alerting	Automation
Among HCPs	Communication
EHR	Integration
	Motivation
Sharing	Role/responsibilities
Sharing	Role/responsibilities
Training	Training/Educating
Educating	Training/Educating
Materials	Training/Educating
Explanation (patients)	Training/Educating
Explanation	Training/Educating
Explanation	Training/Educating
Simplicity	Usability
Ease-of-data entry	Usability
Workflow	work infrastructure
Guidelines/protocols	work infrastructure
Team-based	work infrastructure
	Workload
Reviewing	Workload
Interaction	Workload
Burden	Workload
	Integration
Health literacy	Capability
Health literacy	Capability
Self-management	Patient empowerment
Self-management	Patient empowerment
Quality-of-care	Clinical benefit

Treatment	Clinical benefit
Relative advantage	Clinical benefit
Accuracy	Clinical benefit
Relative advantage	Clinical benefit
	Adaptability
	Advertising
Workflow	Automation
Digital literacy	Capability
storage	Data
storage	Data
Technical issues	Technical stability
	Integration
Workflow	Integration
Existing systems	Integration
Program champions (HCPs)	Leadership
	Leadership
Program champions (HCPs)	Leadership
Positive reception	Motivation
Perceived value	Motivation
	Need
Personal privacy	Privacy
Timely introduction	Process
mainstream	Social/economic influence
Increased responsibility	Role/responsibilities
	Social influence
Like/dislike	Technology attitudes
(perceived) positive perceptions	Technology attitudes
Embraced	Technology attitudes
Like/dislike	Technology attitudes
Training	Training/Educating
Phone visits	Type
Simplicity	Usability
Simplicity	Usability
Summarizing	Visualization
Amount/readability	Visualization
Guidelines/protocols	Work infrastructure
adapting (Guidelines/protocols)	work infrastructure

Sharing	work infrastructure
Supportive (within clinic)	work infrastructure
Guidelines/protocols	work infrastructure
Guidelines/protocols	work infrastructure
Time	work infrastructure
Delineating Responsibilities/roles	work infrastructure
NPHW	work infrastructure
Delineating Responsibilities/roles	work infrastructure
Sharing	work infrastructure
Scheduling	work infrastructure
Time	work infrastructure
Staffing	work infrastructure
Time	work infrastructure
Time	workload
Documentation	workload
Implementation	workload
Discussion	Patient-HCP
	workload
Reviewing	workload
Interaction	Workload
	Patient empowerment
Shared-decision making	Patient-focused care
Health literacy	Capability
Tailored care	Patient-focused care
Productive	Patient-focused care
Tailored care	Patient-focused care
Tailored care	Patient-focused care
Self-management	Patient empowerment
Accuracy	Clinical benefit
Accuracy	Clinical benefit
Management	Clinical benefit
Efficiency	Clinical benefit
Patient pathway	Clinical benefit
language	Adaptability
Exporting	Adaptability
Treatment options	Adaptability
Qualitative data	Data

old age	Demographics
Education	Demographics
Non-native speaker	Demographics
EHR	Integration
Instructions	Misunderstanding
Patient preference for HCP leading	Autonomy
Disagreement	Technology attitudes
Accuracy of health information	Trustworthiness
Accuracy of health information	Trustworthiness
Accuracy of health information	Trustworthiness
User-friendly	Usability
User-friendly for patient	Usability
User-friendly	Usability
pictures	Visualization
Contrast	Visualization
pictures	Visualization
Font size	Visualization
Medical language (for patients)	Visualization
Safety	Patient safety
understanding	Patient-HCP
Patient understanding	Patient-HCP
Time	Workload
Guidelines/protocols	work infrastructure
Staffing	Work infrastructure
Access to care	Accessibility
Tailoring alerts	Adaptability
Tailoring alerts	Adaptability
(over)alerting	Alerting accuracy
Health literacy	Capability
Technical issues	Technical stability
Connectivity	Technical stability
Technical issues	Technical stability
Familiarity	Experiences
	Financing/cost
Profiting	Financing/cost
Billing	Financing/cost
Internet/WIFI	ICT infrastructure

Equipment	ICT infrastructure
EHR	Integration
	Motivation
	Patient buy-in
Standardization	Policies and laws
Billing	Policies and laws
Standardization	Policies and laws
Timely introduction	Process
Timely introduction	Process
Burnout	Psychological
Training	Training/Educating
Training	Training/Educating
Training	Training/educating
Training	Training/educating
Training	Training/educating
Training	Training/educating
Time HCP	Training/educating
Data management	Visualization
Staffing	work infrastructure
Designated group	work infrastructure
Staffing (expertise)	work infrastructure
Staffing (expertise)	work infrastructure
Third-party	work infrastructure
Standardization	work infrastructure
Guidelines/protocols	work infrastructure
Staffing (expertise)	work infrastructure
Staffing	work infrastructure
Staffing (expertise)	work infrastructure
Milestones	work infrastructure
Staffing	Work infrastructure
Workflow	Work infrastructure
Guidelines/protocols	Work infrastructure
Technical support	Workload
Time	Workload
Interaction	Workload
Interaction	Workload
Increased responsibility	Role/responsibilities

clinical skills	Clinical benefit
Prevention	Clinical benefit
Quality-of-care	Clinical benefit
Efficiency	Clinical benefit
Quality-of-care	Clinical benefit
Meaningful	Clinician empowerment
Access to care	Accessibility
Access to care	Accessibility
Patient age	Adaptability
	Advertising
Alerting	Automation
Referral	Automation
Alerting	Automation
HCP responsibility	Autonomy
Selective use	Autonomy
Staff compensation	Financing/cost
	Financing/cost
(free) services	Financing/cost
Staff compensation	Financing/cost
Workflow	Integration
Workflow	Integration
	Leadership
Positive reception	Motivation
Positive reception	Motivation
Confidentiality	Privacy
Timely delivery	Process
Clinical duties	Alignment
Concerns	Technology attitudes
Sceptical (general)	Technology attitudes
Educating	Training/Educating
Training	Training/Educating
Training	Training/Educating
Testing	Training/Educating
Time HCP	Training/Educating
Testing	Training/Educating
HCP expertise	Trustworthiness
Simplicity	Usability

Convenience	Usability
User-friendly	Usability
Time	work infrastructure
Workflow	work infrastructure
Time	work infrastructure
Staffing (expertise)	work infrastructure
Delineating Responsibilities/roles	work infrastructure
NPHW	work infrastructure
Time	workload
Satisfaction	Psychological
Anxiety	Psychological
Implementation	Workload
	Workload
Documentation	Workload
Time	Workload
Clinical goals/targets	Alignment
Reminder	Clinical benefit
Adherence	Clinical benefit
Quality-of-care	Clinical benefit
Efficiency	Accuracy of DHI content
Accuracy	Accuracy of DHI content
Accuracy	Accuracy of DHI content
(under)alerting	Alerting accuracy
Revising	Autonomy
Data	Integration
Data	Integration
Forgetting	Mis (use)
Ignoring	Mis (use)
Instructions	Misunderstanding
Perceived value	Motivation
	Need
Before consultation	Process
Increased responsibility	Role/responsibilities
Like/dislike	Technology attitudes
Useful	Technology attitudes
Useful	Technology attitudes

Training	Training/Educating
Training	Training/Educating
Explanation	Training/Educating
Training	Training/Educating
HCP expertise	Trustworthiness
Readiness	Usability
Interactive	Usability
	Visualization
	Visualization
Amount/readability	Visualization
Time	work infrastructure
Sharing	work infrastructure
Time	work infrastructure
Workflow	work infrastructure
Supportive (within clinic)	work infrastructure
Time	Workload
Time	Workload
Health outcomes	Clinical benefit
Health literacy	Capability
Health outcomes	Clinical benefit
adherence	Clinical benefit
retention	Clinical benefit
Tailored care	Patient-focused care
self-management	patient empowerment
Access to technology	Accessibility
Access to mobile phone	Accessibility
Size	Adaptability
Health literacy	Capability
Digital literacy	Capability
Mental/physical fitness	Demographics
socioeconomic status	demographics
Pairing	Technical stability
Technical issues	Technical stability
Familarity	Experiences
(loaning) equipment	Financing/cost
Long-term sustainability	Financing/cost
staff compenstation	Financing/cost

Internet/WIFI	ICT infrastructure
EHR	Integration
Pairing	Interoperability
Program champions (HCPs)	Leadership
Mentorship (HCPs)	Leadership
Expectations	Misunderstanding
Reimbursement	Policies and laws
HCPs	Social support
Educating	Training/educating
Training	Training/educating
Health system	Trustworthiness
Staffing	work infrastructure
Staffing	work infrastructure
Third-party	work infrastructure
	Workload
Burden	psychological
Planned use	Intention to use
Planned use	Intention to use
Communication	Patient-HCP
Efficiency	Clinical benefit
Peer pressure	Social influence
Patient privacy	Privacy
Management	Clinical benefit
Perceived value	Motivation
Peer pressure	Social influence
User-friendly	Usability
Interaction	Patient-HCP
Support	Patient-focused care
	Motivation
Ease	Clinical benefit
Retention	Clinical benefit
Evaluation	Clinical benefit
Among HCPs	Communication
Digital literacy	Capability
Digital literacy	Capability
Mental/physical fitness	Demographics
	Financing/cost

equipment	Financing/cost
Systems	ICT infrastructure
Compatibility	Integration
Willingness	Motivation
Patient privacy	Privacy
Patient confidentially	Privacy
confidentiality	Privacy
Interest	Technology attitudes
User-friendly for patient	Usability
workflow	work infrastructure
Reassurance	Patient-focused care
Time	Work infrastructure
Training	Training/educating
Access to patient data	Accessibility
Accuracy	Clinical benefit
Diagnosis	Clinical benefit
old age	Demographics
Technical issues	Technical stability
technical issues	Technical stability
Patient file	Integration
	Interoperability
Training resources	Mis (use)
perceived value	Motivation
Disagreement	Role/responsibilities
Disagreement	Role/responsibilities
Liability	Role/responsibilities
comfortable	Technology attitudes
Time patient	Training/educating
one-on-one training	Training/educating
testing	Training/educating
Training	Training/educating
Accuracy	Trustworthiness
user-friendly	Usability
Installing steps	Usability
Summarizing	Visualization
Contact person	work infrastructure
Time	work infrastructure
Scheduling	work infrastructure

NPHW	work infrastructure
NPHW	work infrastructure
Implementation	Workload
Time	Workload
Interaction	Workload
Time	Workload
Interaction	Workload
Technical support	Workload
Technical support	Workload
Interaction	Workload
Burden	Workload
Responding (family)	Workload
Reviewing	Workload
Self-management	Patient empowerment
Health literacy	Capability
Reassurance	Patient-focused care
Anxiety	psychological
Self-management	Patient empowerment
Responsiveness	Patient-focused care
Health outcome	Clinical benefit
Health outcome	Clinical benefit
Management	Clinical benefit
Accuracy	Clinical benefit
health outcome	Clinical benefit
Safety	Patient safety
clinical safety	Patient safety
Timely response	Accuracy of DHI content
Data	Accuracy of DHI content
language	Adaptability
Communication impediments	Adaptability
Patient (individualised medication)	Adaptability
Health concern/problem	Adaptability
Alerting	Automation
Alerting (patients)	Automation
Alerting	Automation
Digital literacy	Capability
Literacy	Capability

	Collaboration/connectivity
Among HCPs	Collaboration/connectivity
	Data
old age	Demographics
Severity	Demographics
language	Demographics
Technical issues	Technical stability
Reminders	Feedback
	Financing/cost
	Financing/cost
Internet/WIFI	ICT infrastructure
Central hub	Integration
Existing systems	Integration
Existing systems	Integration
Communication	Patient-HCP
Insurance companies	Policies and laws
Patient privacy	Privacy
Patient confidentially	Privacy
Patient security	Privacy
	sociocultural
training	Training/educating
training	Training/educating
Accuracy	Trustworthiness
User-friendly	Usability
User-friendly for patient	Usability
NPHW	work infrastructure
Staffing (expertise)	work infrastructure
NPHW	work infrastructure
Anxiety	psychological
Time	Workload
burden	Workload
Time	Workload
Patient data responsbility	Role/responsibilities
Comprehensive	Clinical benefit
Comprehensive	Clinical benefit
objective	Clinical benefit
Accuracy	Clinical benefit

Standardization	Clinical benefit
Standardization	Accuracy of DHI content
Sufficiency	Accuracy of DHI content
Contextual information	Content
Contextual information	Content
Years of work experience	Demographics
EHR	Integration
Documentation	Mis (use)
Documentation	Mis (use)
Forgetting	Mis (use)
Patient contact responsibility	Role/responsibilities
HCP leading	Autonomy
Reduced shared responsibility	Role/responsibilities
increased responsibility	Role/responsibilities
Shifted roles	Role/responsibilities
increased responsibility	Role/responsibilities
Accuracy	Trustworthiness
Patient ability	Trustworthiness
Interpreration of data	Visualization
Interpretation of data	Visualization
Guidelines/protocols	Work infrastructure
burden	Psychological
Stress	Psychological
Efficiency	Clinical benefit
Health outcome	Clinical benefit
Health outcome	Clinical benefit
Adherence	Clinical benefit
Behaviour change	Clinical benefit
old age	demographics
work status	demographics
Severity	demographics
High-risk	demographics
Technical issues	Technical stability
Equipment	Financing/cost
	Financing/cost
Addition	Technology attitudes
Sceptical (ability)	Technology attitudes

(perceived) positive perceptions	Technology attitudes
Training	Training/Educating
Reliability	Trustworthiness
	Usability
Time	Workload
Time	Workload
Past habits	Experiences
Medical history	Appropriate
DHT	Awareness
Old age	Demographics
Old age	Demographics
Efficiency	clinical benefit
security	Data
perceived value	Motivation
Health outcomes	clinical benefit
Treatment	clinical benefit
Adherence	clinical benefit
Behaviour change	clinical benefit
Access to internet/Wifi	Accessiblity
Old age	Appropriate
Old age	Demographics
Severity	Demographics
work status	Demographics
Technical issues	Technical stability
Familiarity	Experiences
	Financing/cost
Planned use	Intention to use
	Training/Educating
DHT ability	Trustworthiness
Accuracy	Trustworthiness
Data security/privacy	Trustworthiness
Interpretation of data	Visualization
Management	Clinical benefit
Quality-of-care	Clinical benefit
Support	Patient-focused care
Simplicity/efficiency (care)	Patient-focused care
Responsiveness	Patient-focused care

Access to care	Accessibility
Access to care (for sick)	Accessibility
Quality-of-care	Clinical benefit
Continuity-of-care	Clinical benefit
Efficiency	Clinical benefit
Reliability	Accuracy of DHI content
Medical history	Appropriate
selective use	Autonomy
Privacy	Data
secure	Data
Severity	Demographics
Business model	Financing/cost
	Financing/cost
	Financing/cost
Staff compensation	Financing/cost
NPHW	Financing/cost
	Financing/cost
Business model	Financing/cost
Workflow	Integration
Expectations	Misunderstanding
Perceived value	Motivation
Financial incentives	Motivation
Insurance companies	Policies and laws
Policy	Policies and laws
Regulations	Policies and laws
Reimbursement	Policies and laws
Regulations	Policies and laws
Privacy	Privacy
Setting expectations	Process
substitution	Technology attitudes
substitution	Technology attitudes
Training	Training/Educating
Safety	Trustworthiness
Data	Visualization
NPHW	work infrastructure
Reviewing	Workload
Reviewing	Workload

	Data
Clinical testing	Human aspect
Treatment	Clinical benefit
	Misunderstanding
Boredom	Motivation
Adherence	Clinical benefit
Interaction	Patient-HCP
Variety	Adaptability
Technical issues	Technical stability
Evidence-based	Evidence-based
Evidence-based	Evidence-based
Network	ICT infrastructure
EHS	ICT infrastructure
Reimbursement	Policies and laws
Patient privacy	Privacy
Phone visits	Type
Interaction (patient)	Usability
self-efficacy	Patient empowerment
Workflow	Integration
Accuracy	Accuracy of DHI content
Reliability	Accuracy of DHI content
(over)alerting	Alerting accuracy
Selective use	Autonomy
Health	Autonomy
Health literacy	Capability
EHR	Integration
Existing systems	Integration
Information overlooked	Mis (use)
Information overlooked	Mis (use)
Ignoring	Mis (use)
Ignoring	Mis (use)
Self-management	Motivation
Adherence	Motivation
Patient health responsibility	Role/responsibilities
User-friendly	Usability
Interpretation of data	Visualization
Data	Visualization

Data	Visualization
Workflow	work infrastructure
Guidelines/protocols	work infrastructure
	workload
Self-management	Patient empowerment
Supportive (within clinic)	Work infrastructure
Among HCPs	Collaboration
Time	Work infrastructure
Perceived value	Motivation
Among HCPs	Collaboration
Authority	Leadership
Time	Workload
Among HCPs	Collaboration
	Workload
Access to care (rural areas)	Accessibility
Quality-of-care	clinical benefit
Access to care (rural areas)	Accessibility
With patients	Co-design
With HCPs	Co-design
Consumer	Co-design
Multi-stakeholder collaboration	Co-design
Locally-adapted	Co-design
Internet/WIFI	ICT infrastructure
Network	ICT infrastructure
Concerns	Technology attitudes
Money-making	Technology attitudes
Concerns	Technology attitudes
Money-making	Technology attitudes
Organization	Trustworthiness
Quality-of-care	Trustworthiness
Cross-sectional collaboration	work infrastructure
Clinical testing	Human aspect
Sensory constraints	demographics
Technical issues	Technical stability
Equipment	ICT infrastructure
User-friendly	Usability
Simplicity/efficiency (care)	Patient-focused care

Reassurance	Patient-focused care
Access to care	Accessibility
Simplicity/efficiency (care)	Patient-focused care
Diagnosis	Clinical benefit
Efficiency	Clinical benefit
Cost-effectiveness	Clinical benefit
Support from other HCPs	Collaboration
Among HCPs	Communication
Mental/physical fitness	Demographics
Technical issues	Technical stability
Technical issues	Technical stability
	Financing/cost
Supervision	Leadership
Positive reception	Motivation
	Need
Familiarity	Patient-HCP
Rapport	Patient-HCP
Communication	Patient-HCP
Familiarity	Patient-HCP
Setting	Physical space/infrastructure
DHT ability	Trustworthiness
DHT ability	Trustworthiness
Time	Workload
Technical issues	Technical stability
Quality-of-care	clinical benefit
Quality-of-care	clinical benefit
Management	clinical benefit
Discussion	Patient-HCP
Communication	Patient-HCP
Access to technology	Accessibility
Access to technology	Accessibility
Email	Accessibility
Medical history	Appropriate
Medical history	Appropriate
Patient need perceptions	Autonomy
Digital literacy	Capability
Digital literacy	Capability

Sensory constraints	Demographics
Sensory constraints	Demographics
Technical issues	Technical stability
(loaning) equipment	Financing/cost
Clinical testing	Human aspect
Clinical testing	Human aspect
equipment	ICT infrastructure
Systems	ICT infrastructure
Authority	Leadership
Top-down approach	Leadership
Authority	Leadership
Communication	Misunderstanding
Communication	Patient-HCP
Setting	Physical space/infrastructure
Family	Social support
Reluctance/resistance	Technology attitudes
Familiarity	Training/Educating
Patient ability	Trustworthiness
Phone visits	Type
Simplicity (patients)	Usability
Simplicity (patients)	Usability
Simplicity (patients)	Usability
Simplicity	Usability
Supportive (within clinic)	work infrastructure
Guidelines/protocols	work infrastructure
Standardization	work infrastructure
Staffing (expertise)	work infrastructure
NPHW	work infrastructure
Implementation	Workload
Among different disciplines	Collaboration
Communication	Patient-HCP
Responsiveness	Patient-focused care
decision-making	Patient empowerment
Sufficiency	Accuracy of DHI content
Variety	Adaptability
More content	Adaptability
Realistic content	Adaptability

Patient needs	Adaptability
Patient education level	Appropriate
	Autonomy
Digital literacy	Capability
Education	Demographics
old age	Demographics
Animations	Gamification
Concerns of patient-liking	Gamification
Old age patients	Gamification
Reward system concerns	Gamification
Perceived value	Motivation
supportive	Motivation
Explanation	Training/Educating
Prior to appointment	Training/Educating
Repetition	Training/Educating
shame	Psychological
Access to patient data	Accessibility
Management	Clinical benefit
retention	Clinical benefit
Standardization	Clinical benefit
co-morbidities	Adaptability
outliers	Adaptability
supply changes	Adaptability
storage	Data
security	Data
Interaction	Workload
Consultation	Workload
Access to mobile phone	Accessibility
Literacy	Capability
Long-term sustainability	Financing/cost
staff compensation	Financing/cost
(non)-digitalized ICT structures	ICT infrastructure
Setting	Physical space/infrastructure
electricity	Power and energy infrastructure
religious	sociocultural
Resources	Work infrastructure
Resources	Work infrastructure

staffing	work infrastructure
Time	Workload
implementation	Workload
burnout	Workload
Access to care (rural areas)	Accessibility
Access to care (for sick)	Accessibility
stress	Psychological
burden	Workload
Access to care	Accessibility
Waiting time	Workload
Reassurance	Patient-focused care
Efficiency	Clinical benefit
Efficiency	Clinical benefit
Diagnosis	Clinical benefit
Management	Clinical benefit
Satisfaction	Psychological
Hospital visits	Workload
treatment	Clinical benefit
Among HCPs	Communication
Among HCPs	Communication
In-person	Financing/cost
Positive reception	Motivation
Interaction	Patient-HCP
HCP reassurance	Patient-HCP
Video visits	Type
Technical support	Workload
time	Workload
Medical decision making	Clinical benefit
Efficiency	Clinical benefit
Diagnosis	Clinical benefit
among HCPs	Collaboration
Technical issues	Technical stability
Technical issues	Technical stability
Clinical testing	Human aspect
Senses	Human aspect
NPHW	work infrastructure
Scheduling	work infrastructure

Scheduling	work infrastructure
Time	Workload
Waiting time	Workload
Time	Workload
Program reimbursement	Financing/cost
Safety	Patient safety
access to technology	Accessibility
Access to internet/Wifi	Accessibility
access to technology	Accessibility
Access to equipment	Accessibility
Patient abilities	Adaptability
Patient abilities	Adaptability
Digital literacy	Capability
Privacy	Data
Privacy	Data
Severity	demographics
(loaning) equipment	Financing/cost
Clinical testing	Human aspect
Equipment	ICT infrastructure
Exercise	Patient safety
Exercise	Patient safety
	Physical space/infrastructure
Liability	Policies and laws
Declining	Technology attitudes
training	training/educating
training	training/educating
Hospital approved	Trustworthiness
User-friendly	Usability
User-friendly for patient	Usability
staffing	work infrastructure
Consultation	Workload
Access to care (rural areas)	Accessibility
Perceived value	Motivation
	Clinical benefit
Accuracy	Clinical benefit
Identification	Clinical benefit
Efficiency	Clinical benefit

Among HCPs	Communication
Access to technology	Accessibility
Transparency	Accuracy of DHI content
Health concern/problem	Adaptability
Health concern/problem	Adaptability
Human-AI partnership	Adaptability
Health concern/problem	Adaptability
Workflow	Autonomy
Alert setting	Autonomy
Selective use	Autonomy
Trust	Autonomy
With HCPs	Co-design
With HCPs	Co-design
Among HCPs	Collaboration
Evidence-based	Evidence-based
Past habits	Experiences
Workflow	Integration
Expectations	Misunderstanding
Expectations	Misunderstanding
Expectations	Misunderstanding
	Need
	Need
	Need
	Need
Training	Training/Educating
Training	Training/Educating
Accuracy	Trustworthiness
Accuracy	Trustworthiness
Click-ability	Usability
Convenience	Usability
	Visualization
Data	Visualization
Time	Workload
Type of exercise	Content
Discussion	Patient-HCP
Patient understanding	Patient-HCP
	Accuracy of DHI content

(over)alerting	Alerting accuracy
Reason for no PA	Content
workflow	Integration
skipping features	Mis (use)
Skipping features	Mis (use)
Listening	Mis (use)
Perceived value	Motivation
User-friendly	Usability
staffing	work infrastructure
Interaction	Workload
Simplicity	Usability
Access to patient data	Accessibility
Digital literacy	Capability
Management	clinical benefit
Management	clinical benefit
Management	Clinical benefit
Efficiency	clinical benefit
Medical decision making	clinical benefit
Quality-of-care	clinical benefit
Clinical safety	Patient safety
Clinical skills	Adaptability
Alerting	Automation
Data collection	Automation
Alerting	Automation
Digital literacy	Capability
Digital literacy	Capability
Digital literacy	Capability
Willingness	Motivation
Tool	Technology attitudes
Testing	Training/Educating
Training	Training/Educating
Confidence	Psychological
Familiarity	Training/Educating
User-friendly	Usability
Simplicity (patients)	Usability
Simplicity	Usability
Guidelines/protocols	work infrastructure

Staffing (expertise)	work infrastructure
	Workload
Documentation	Workload
Time	Workload
self-management	patient empowerment
(loaning) equipment	Financing/cost
Access to care	Accessibility
Access to equipment	Accessibility
Access to technology	Accessibility
Validating	Accuracy of DHI content
Sufficiency	Accuracy of DHI content
Accuracy	Accuracy of DHI content
	Automation
Technical issues	Technical stability
Past habits	Experiences
Equipment	Financing/cost
Workflow	Integration
Prioritizing	Motivation
Data sharing	Patient-HCP
Training	Training/Educating
Size	Usability
Workflow	Work infrastructure
Self-management	Patient empowerment
During consultation	Process
managment	clinical benefit
perceived value	Motivation
Self-efficacy	Clinician empowerment
Time	work infrastructure
Management	Clinical benefit
Shared-decision making	Patient-focused care
Tailored care	Patient-focused care
perceived value	Motivation
Positive patient feedback	Motivation
One-way	Communication
One-way	Communication
Positive reception	Motivation
Training	Training/Educating

Accuracy	Trustworthiness
Reliability	Trustworthiness
Admin	Work infrastructure
security	Data
Evidence-based	Evidence-based
	Financing/cost
Internet/WIFI	ICT infrastructure
Reimbursement	Policies and laws
Reimbursement	Policies and laws
Enrolment	work infrastructure
	Clinical benefit
Support	Patient-focused care
Supportive	Patient-focused care
perceived value	Motivation
	Need
Delivering	Workload
Quality-of-care	Clinical benefit
Clinical safety	Patient safety
Effective	clinical benefit
Effective	clinical benefit
Efficiency	clinical benefit
Retention	clinical benefit
Tailored care	Patient-focused care
Access to care	Accessibility
Equitable access to care	Accessibility
Access to care (rural areas)	Accessibility
Equitable access to care	Accessibility
Equitable access to care	Accessibilty
Tailored care	Patient-focused care
Tailored care	Patient-focused care
Responsiveness	Patient-focused care
Frustration	Psychological
Isolation/loneliness	Psychological
Reassurance	Patient-focused care
Waiting time	workload
Continuity-of-care	Clinical benefit
Diagnosis	Clinical benefit

Management	Clinical benefit
Continuity-of-care	Clinical benefit
Diagnosis	Clinical benefit
Medical decision making	Clinical benefit
	Clinical benefit
Quality-of-care	Clinical benefit
Quality-of-care	Clinical benefit
Clinical safety	Patient safety
Clinical safety	Patient safety
Emotional support	Patient-HCP
Communication	Patient-HCP
Interaction	Patient-HCP
Communication	Patient-HCP
Rapport	Patient-HCP
Stress	Psychological
Frustration	Psychological
Stress	Psychological
Burnout	Psychological
Fatigue	Psychological
Burnout	Psychological
Access to technology	Accessibility
Access to mobile phone	Accessibility
Medical history	Appropriate
Health literacy	Capability
Digital literacy	Capability
	Communication
Contextual information	Content
old age	Demographics
Severity	Demographics
Severity	Demographics
Non-native speaker	Demographics
Sensory constraints	Demographics
Patient home (distracting)	Environment
Patient home (distracting)	Environment
Phone coverage	Financing/cost
Staff compensation	Financing/cost
Clinical testing	Human aspect

Clinical testing	Human aspect
Visual cues	Human aspect
Planned use	Intention to use
Expectations	Misunderstanding
Policy (equity)	Policies and laws
Regulations (privacy and data secur	Policies and laws
Setting expectations	Process
Not first-encounter	Technology attitudes
Educating	Training/Educating
Video visits	Type
Phone visits	Type
Simplicity (patients)	Usability
Simplicity	Usability
Time	work infrastructure
Staffing (expertise)	work infrastructure
Staffing (expertise)	work infrastructure
Staffing (expertise)	work infrastructure
New hires	work infrastructure
Workflow	work infrastructure
Organization	work infrastructure
Contact person	work infrastructure
time	workload
Equitable access to care	Accessiblity
Time	Workload
Effort	Workload
Access to patient data	Accessibility
Efficiency	Clinical benefit
Access to internet/Wifi	Accessibility
HCP preferences	Adaptability
Iterative approach	Adaptability
(over)alerting	Alerting accuracy
HCP needs	Autonomy
With HCPs	Co-design
With HCPs	Co-design
storage	Data
Internet/WIFI	ICT infrastructure
Workflow	Integration

EHR	Integration
Existing systems	Integration
Increased responsibility	Role/responsibilities
Materials	Training/Educating
User-friendly	Usability
User-friendly	Usability
Click-ability	Usability
Staffing (expertise)	work infrastructure
Hospital visits	Workload
Time	Workload
Interaction	Workload
Redundancy	Workload
Network	ICT infrastructure
Poor tech	Technical stability
Internet/WIFI	ICT infrastructure
Mutliple systems	ICT infrastructure
Internet/WIFI	ICT infrastructure
EHS	ICT infrastructure
e-health platforms	Interoperability
Efficiency	Clinical benefit
Management	Clinical benefit
Medical decision making	Clinical benefit
Documentation	Clinical benefit
Job quality	Clinical benefit
Reducing error rate	Clinical benefit
Communication	Clinical benefit
Cost-effectiveness	Clinical benefit
Safety	Patient safety
	Autonomy
Literacy	Capability
Digital literacy	Capability
Old age	Demographics
Old age	Demographics
Education	Demographics
	Experiences
Expectations	Misunderstanding
Empowerment	Motivation

Economic	Social/economic influence
Threating	Technology attitudes
Tool	Technology attitudes
	Technology attitudes
(perceived) positive perceptions	Technology attitudes
Fear	Technology attitudes
User-friendly for patient	Usability
Organization	work infrastructure
Organization	work infrastructure
Reassurance	Patient-focused care
Responsiveness	Patient-focused care
Access to care	Accessibility
Quality-of-life	Patient-focused care
Shared-decision making	Patient-focused care
Shared-decision making	Patient-focused care
Documentation	Workload
Digital literacy	Capability
Equitable access to technology	Accessibility
Patient abilities	Adaptability
Personalized	Adaptability
Interaction frequency	Adaptability
Tailoring alerts	Adaptability
Patient needs	Adaptability
Personalized	Adaptability
Filling-in	Automation
	Autonomy
Digital literacy	Capability
With HCPs	Co-design
With patients	Co-design
With patients (underrepresented)	Co-design
Feedback	Communication
Mental health	Content
old age	Demographics
Evidence-based	Evidence-based
Reminders	Feedback
	Financing/cost
Reward system	Gamification

	Gamification
Reward system concerns	Gamification
Patient life	Integration
incentivization	Motivation
Communication	Patient-HCP
Privacy	Privacy
Timely introduction	Process
fear	psychological
Family (champions)	Social support
	Social support
Champion	Social support
Patients	Social support
Friends	Social support
Patients	Social support
Embraced	Technology attitudes
Like/dislike	Technology attitudes
training	Training/Educating
Materials	Training/Educating
Training (caregivers)	Training/Educating
	Trustworthiness
Accuracy of health information	Trustworthiness
Accuracy of health information	Trustworthiness
Simplicity (patients)	Usability
	Visualization
	Visualization
Data	Visualization
Data	Visualization
Amount/readability	Visualization
Font size	Visualization
Supportive (within clinic)	work infrastructure
old age	Demographics
City	Demographics
Perceived value	Motivation
Familiarity	Experiences
Past habits	Experiences
Past habits	Experiences
Familiarity	Experiences

Staff compensation	Financing/cost
Preferences	Technology attitudes
Confidentiality	Privacy
Willingness	Motivation
Training	Training/Educating
Training	Training/Educating
Perceived value	Motivation
Preferences	Technology attitudes
Reimbursement	Awareness
EHR	Integration
Interaction	Workload
Access to internet/Wifi	Accessibility
Personalized	Adaptability
Language	Adaptability
Literacy levels	Adaptability
Personalized	Adaptability
Patient responses	Automation
Patient need perceptions	Autonomy
Tailoring data-sharing options	Autonomy
Tailoring privacy	Autonomy
old age	Demographics
Sensory constraints	Demographics
Sensory constraints	Demographics
Technical issues	Technical stability
Existing systems	Integration
Workflow	Integration
workflow	Integration
Workflow	Integration
Other apps	Interoperability
Improper	Mis (use)
Patient responses	Trustworthiness
Patient ability	Trustworthiness
Simplicity (patients)	Usability
Convenience (patients)	Usability
Graphical representation	Visualization
Trends	Visualization
Guidelines/protocols	work infrastructure

Defined roles	work infrastructure
Interaction	Workload
	Motivation
Access to technology	Accessibility
Digital literacy	Capability
Privacy	Data
Evidence-based	Evidence-based
Funding	Financing/cost
	Motivation
Regulations	Policies and laws
Reimbursement	Policies and laws
DHT ability	Trustworthiness
Cost-effectiveness	Trustworthiness
Identification	clinical benefit
Management	clinical benefit
Tailored care	Patient-focused care
Responsiveness	Patient-focused care
Burden	workload
Self-management	Patient empowerment
Shared-decision making	Patient-focused care
Quality-of-life	Patient-focused care
Sufficiency	Accuracy of DHI content
Medical history	Appropriate
Literacy	Capability
	Collaboration
Qualitative data	Data
Reimbursement	Financing/cost
Staff compensation	Financing/cost
Long-term sustainability	Financing/cost
Reimbursement	Financing/cost
Equipment	Financing/cost
IT resources	ICT infrastructure
Connectivity	Integration
EHR	Integration
Systems (regional)	Interoperability
	Leadership
Financial incentives	Motivation

	Motivation
Familiarity	Patient-HCP
multisectoral collaboration	Policies and laws
Evaluation frameworks	Policies and laws
Device regulations (patient-owned)	Policies and laws
Explanation	Training/Educating
Training	Training/Educating
Training	Training/Educating
Training	Training/Educating
Materials	Training/Educating
HCP expertise	Trustworthiness
Staffing (expertise)	work infrastructure
Defined roles	work infrastructure
Staffing (expertise)	work infrastructure
Guidelines/protocols	work infrastructure
Standardization	work infrastructure
Burden	workload
technical support	Workload
	Workload
Documentation	Workload
Cross-sectional collaboration	work infrastructure
Access to technology	Accessibility
Equitable access to technology	Accessiblity
Equitable access to technology	Accessiblity
Communication	Adaptability
Clinical goals/targets	Alignment
Alert setting	Autonomy
Digital literacy	Capability
Locally-adapted	Co-design
Among different disciplines	Collaboration
Clinic-national health level	Communication
Self-management	Content
(clinical) skill sets	Demographics
old age	Demographics
Socioeconomic status	Demographics
High-risk	Demographics
Severity	Demographics

(loaning) equipment	Financing/cost
Reimbursement	Financing/cost
Financial incentives	Financing/cost
Funding	Financing/cost
IT resources	ICT infrastructure
Financial incentives	Incentivization
Workflow	Integration
Workflow	Integration
EHR	Integration
Comptability	Integration
Program champions (HCPs)	Leadership
Top-down approach	Leadership
Engaging	Motivation
Discussion	Patient-HCP
National guidance	Policies and laws
Educating	Training/Educating
Training	Training/Educating
Assessment	Training/Educating
Materials	Training/Educating
Training	Training/Educating
Centralization	work infrastructure
Defined roles	work infrastructure
Sharing	work infrastructure
Defined roles	work infrastructure
Staffing (expertise)	work infrastructure
Staffing	work infrastructure
Time	work infrastructure
Staff turnover	work infrastructure
Resources	Work infrastructure
Staffing (expertise)	work infrastructure
Guidelines/protocols	Work infrastructure
Multiple systems	Workload
Reviewing	Workload
	Workload
Patient education	Need
Accuracy	Clinical benefit
Management	clinical benefit

Accuracy	Clinical benefit
self-managment	Patient empowerment
Self-management	patient empowerment
self-management	Patient empowerment
Productive	Patient-focused care
Tailored care	Patient-focused care
Access to care	Accessibility
Responsiveness	Patient-focused care
Support	Patient-focused care
Health literacy	Capability
Health literacy	Capability
Support	Patient-focused care
Interaction	Patient-HCP
Patient understanding	Patient-HCP
Personalized	Adaptability
Data selection	Autonomy
Old age	Demographics
Technical issues	Technical stability
In-person preference	Technology attitudes
Hybrid	Type
User-friendly	Usability
Contact person	work infrastructure
Staffing (expertise)	work infrastructure
Access to care (rural areas)	Accessibility
Access to patient data	Accessibility
Access to patient data	Accessibility
Access to patient data	Accessibility
Medical decision making	Clinical benefit
Health outcome	Clinical benefit
efficiency	Clinical benefit
efficiency	Clinical benefit
optimizing	Clinical benefit
Efficiency	clinical benefit
Documentation	Clinical benefit
Access to patient data	Accessibility
Productive	Patient-focused care
Access to care (for sick)	Accessibility

[illegible] preferences	Accessibility
	Accessibility
Cultural norms	Adaptability
Privacy	Data
Privacy	Data
Communication	Patient-HCP
Rapport	Patient-HCP
Privacy	Privacy
HCP Gender preference	sociocultural
Staffing (expertise)	work infrastructure
Planned use	Intention to use
User-friendly	Usability
Medical history	Appropriate
Severity	Demographics
Mental/physical fitness	Demographics
Sensory constraints	Demographics
Language	Demographics
Clinical testing	Human aspect
Clinical testing	Human aspect
Non-verbal	Human aspect
Equipment	ICT infrastructure
EMR	ICT infrastructure
Ready-to-use	ICT infrastructure
Patient file	Integration
space	Physical space/infrastructure
Not first-encounter	Technology attitudes
Training	Training/educating
Phone visits	Type
Video visits	Type
User-friendly for patient	Usability
	Usability
workflow	work infrastructure
Guidelines/protocols	work infrastructure
Communication	Patient-HCP
Identification	Clinical benefit
prevention	Clinical benefit
	Technology attitudes

caregivers	Technology attitudes
Patient needs	Adaptability
Access to internet/Wifi	Accessibility
Access to internet/Wifi	Accessibility
Access to mobile phone	Accessibility
Language	Adaptability
Health concern/problem	Adaptability
Past habits	Experiences
Self-learning skills	Mis (use)
	Motivation
Self-management	Motivation
Interaction	Patient-HCP
Video visits	Type
Video visits	Type
User-friendly for patient	Usability
User-friendly for patient	Usability
	Usability
Medical language (for patients)	Visualization
Data	Visualization
Interest	Technology attitudes
Health literacy	Capability
Diagnosis	Clinical benefit
Relative advantage	Clinical benefit
Documentation	Clinical benefit
Access to care	Accessibility
Access to care	Accessibility
Access to care	Accessibility
Personalized	Adaptability
Medical history	Appropriate
Medical history	Appropriate
	Autonomy
Severity	Demographics
	Financing/cost
parking	Financing/cost
Among HCPs	Leadership
Optimism	Motivation
Health	Motivation

Communication	Patient-HCP
Setting	Physical space/infrastructure
space	physical space/infrastructure
	physical space/infrastructure
Accuracy	Trustworthiness
HCP expertise	Trustworthiness
HCP expertise	Trustworthiness
convenience	Usability
Efficiency	Usability
staffing	work infrastructure
Staffing (expertise)	Work infrastructure
staffing	work infrastructure
Supportive (within clinic)	Work infrastructure
Juggling tasks	Workload
Health literacy	Capability
Follow-up	Workload
Identification	Clinical benefit
AI use	Awareness
Efficiency	Clinical benefit
Accuracy	Clinical benefit
Validating	Accuracy of DHI content
Accuracy	Accuracy of DHI content
Filling-in	Automation
	Automation
	Autonomy
	Autonomy
Developer-lens	Co-design
Storage	Data
security	Data
Evidence-based	Evidence-based
Evidence-based	Evidence-based
Evidence-based	Evidence-based
Evidence-based	Evidence-based
Evidence-based	Evidence-based
Evidence-based	Evidence-based
Familiarty	Experiences
Familiarty	Experiences

	Financing/cost
	Financing/cost
[illegible]	Financing/cost
	Financing/cost
Reluctant	Financing/cost
Workflow	Integration
Compatibility	Integration
EHR	Integration
EHR	Integration
Digital systems	Interoperability
Authority	Leadership
Authority	Leadership
Perceived value	Motivation
Perceived value	Motivation
perceived value	Motivation
	Need
Patient confidentially	Privacy
Patient privacy	Privacy
	Staff buy-in
	Staff buy-in
Threating	Technology attitudes
Concerns	Technology attitudes
Educating	Training/Educating
Explanation	Training/Educating
DHT ability	Trustworthiness
Human-AI collaboration	Trustworthiness
Accuracy	Trustworthiness
Organization	Trustworthiness
Organization	Trustworthiness
DHT ability	Trustworthiness
	Usability
Simplicity	Usability
Data	Visualization
Data	Visualization
Amount/readability	Visualization
Evaluation frameworks	Work infrastructure
Staffing (expertise)	Work infrastructure

Time	Workload
Reviewing	Workload
Interaction	Workload
Reviewing	Workload
Equitable access to care	Accessibility
Burden	Workload
	Motivation
Quality-of-care	clinical benefit
health outcome	clinical benefit
health outcome	clinical benefit
holistic	clinical benefit
Clinical testing	Clinical benefit
Meaningful	clinician empowerment
Access to technology	Accessibility
Email	Accessibility
Digital literacy	Capability
Rural or remote areas	demographics
	Financing/cost
Clinical testing	Human aspect
Clinical testing	Human aspect
Perceived value	Motivation
Perceived value	Motivation
Exercise	Patient safety
patients	social support
Time patient	Training/Educating
Hybrid	Type
Sustainability of BC	Patient empowerment
Access to care (rural areas)	Accessibility
Access to care (for sick)	Accessibility
Sustainability of BC	Patient empowerment
patients	social support
Implementation	Workload
EHR	Integration
Health literacy	Capability
Medical decision making	Clinical benefit
Decision-making	Patient empowerment
Health literacy	Capability

Tailored care	Patient-focused care
Filling-in	Automation
Selective use	Autonomy
Patient need perceptions	Autonomy
Workflow	Integration
Planned use	Intention to use
User-friendly	Usability
Statistical information (patients)	Visualization
Time	Workload
Authority	Leadership
Accuracy	Clinical benefit
Efficiency	Clinical benefit
Burden	Workload
Workflow	Integration
EHR	Integration
Familiarity	Patient-HCP
Before consultation	Process
Explanation	Training/Educating
Standardization	work infrastructure
Workflow	work infrastructure
Standardization	Clinical benefit
Reducing error rate	Clinical benefit
Efficiency	Clinical benefit
Retention	Clinical benefit
Adherence	Clinical benefit
Access to mobile phone	Accessibility
Health concern/problem	Adaptability
Language	Adaptability
International/national plans	Alignment
National program	Alignment
Digital literacy	Capability
Digital literacy	Capability
Digital literacy	Capability
Literacy	Capability
Developer-lens	Co-design
old age	Demographics
	Financing/cost

Existing systems	Integration
Authority	Leadership
Authority	Leadership
Fidelity	Mis (use)
Legal factors	Policies and laws
Standardization	Policies and laws
Patient privacy	Privacy
Patient confidentially	Privacy
Reluctance/resistance	Technology attitudes
Human aspect	Technology attitudes
Reluctance/resistance	Technology attitudes
Like/dislike	Technology attitudes
Reluctance/resistance	Technology attitudes
Training	Training/Educating
Time HCP	Training/Educating
Educating	Training/educating
Educating	Training/educating
HCP expertise	Trustworthiness
NPHW	work infrastructure
NPHW	work infrastructure
Staffing (expertise)	work infrastructure
Clear plan	work infrastructure
stakeholders	work infrastructure
Staffing (expertise)	work infrastructure
Resources	Work infrastructure
Standardization	work infrastructure
NPHW	work infrastructure
Time	Workload
Documentation	Workload
Time	Workload
Efficiency	clinical benefit
Efficiency	clinical benefit
Assessment	Clinical benefit
Efficiency	Clinical benefit
Remote work	Health worker benefit
Rapport	Patient-HCP
Trust	Patient-HCP

Communication	Patient-HCP
Technical issues	Technical stability
Technical issues	Technical stability
Access to technology	Accessibility
Access to technology	Accessibility
Patient needs	Adaptability
Medical history	Appropriate
Medical history	Appropriate
Patient need perceptions	Autonomy
	Autonomy
Catastrophes	Environment
Catastrophes	Environment
Clinical testing	Human aspect
EHS	ICT infrastructure
Internet/WIFI	ICT infrastructure
Computers	ICT infrastructure
Health records	Interoperability
Trust	Patient-HCP
Space	Physical space/infrastructure
Ready-to-use	Physical space/infrastructure
Quotas	Policies and laws
Privacy	Privacy
Personal privacy	Privacy
Personal privacy	Privacy
Not first-encounter	Technology attitudes
Addition / not substitution	Technology attitudes
Routine check-ups	Technology attitudes
Future direction	Technology attitudes
Reliability	Trustworthiness
Hybrid	Type
Organization	Work infrastructure
Waiting time	Workload
Hospital visits	Workload
Documentation	Workload
Time	Workload
Waiting time	Workload
Time	Workload

	Motivation
Access to patient data	Accessibility
Efficiency	Clinical benefit
Retention	Clinical benefit
Management	Clinical benefit
Treatment	Clinical benefit
Efficiency	Clinical benefit
Health outcomes	Clinical benefit
Management	Clinical benefit
Health outcomes	Clinical benefit
efficiency	Clinical benefit
Time	Workload
Equitable access to care	Accessibility
Equitable access to care	Accessibility
Access to care	Accessibility
Access to care (for sick)	Accessibility
Self-management	Patient empowerment
Health literacy	Capability
Self-management	Patient empowerment
Support	Patient-focused care
Tailored care	Patient-focused care
Shared-decision making	Patient-focused care
Self-management	Patient empowerment
Access to (health) resources	Accessibility
Health literacy	Capability
Health literacy	Capability
Decision-making	Patient empowerment
Communication	Patient-HCP
Discussion	Patient-HCP
Email	Accessibility
[illegible]	Accessibility
Access to internet/Wifi	Accessibility
Internet/WIFI	Accessibility
Personalized	Adaptability
geographical location	Adaptability
Personalized	Adaptability
Personalized	Adaptability

Old age	Appropriate
	Automation
Reminders	Automation
Alert setting	Autonomy
Credible apps	Awareness
Digital literacy	Capability
among HCPs	Collaboration
Images	Content
Secure	Data
Real-time	Data
old age	Demographics
old age	Demographics
Mental/physical fitness	Demographics
Sensory constraints	Demographics
Mental/physical fitness	Demographics
Non-native speaker	Demographics
Reminders (specific)	Feedback
Reminders	Feedback
Reminders	Feedback
	Financing/cost
Staff compensation	Financing/cost
	Financing/cost
(de) motivation	Gamification
Quizzes	Gamification
Designed maturely	Gamification
Concerns of patient-liking	Gamification
Doubt	Gamification
Old age patients	Gamification
	Incentivization
Compatibility	Integration
Workflow	Integration
EHR	Integration
Compatibility	Integration
Perceived value	Motivation
Self-management	Motivation
Communication (messaging)	Patient-HCP
Interaction	Patient-HCP

Confidentiality	Privacy
Before consultation	Process
	Staff buy-in
Reluctance/resistance (old age)	Technology attitudes
Reluctance/resistance	Technology attitudes
Sceptical (general)	Technology attitudes
Time patient	Training/Educating
User-friendly	Usability
Simplicity	Usability
User-friendly	Usability
Trends	Visualization
Graphical representation	Visualization
	Visualization
Graphical representation	Visualization
Staffing (expertise)	work infrastructure
Guidelines/protocols	work infrastructure
Time	Workload
Time	Workload
Interaction	Workload
Reviewing	Workload
	Need
Efficiency	Clinical benefit
Accuracy	Clinical benefit
	Clinical benefit
Self-management	Patient empowerment
	Patient-focused care
Productive	Patient-focused care
Quality-of-life	[illegible]
prevention	Clinical benefit
Management	Clinical benefit
Health outcomes	Clinical benefit
Management	Clinical benefit
Safety	Patient safety
Data collection	Automation
	Autonomy
Security	Data
Documentation	Mis (use)

Communication	Patient-HCP
Privacy	Privacy
Security	Privacy
Essential	[illegible]
Data security/privacy	Trustworthiness
Data security/privacy	Trustworthiness
Follow-up	Workload
Medical history	Appropriate
Support	Clinical benefit
Access to mobile phone	Accessibility
	Adaptability
Digital literacy	Capability
Digital literacy	Capability
Old age	Demographics
socioeconomic status	Demographics
Rural or remote areas	Demographics
Network	Financing/cost
community	Social support
Organization	Trustworthiness
Messaging	Type
Interaction (patient)	Usability
self-management	Patient empowerment
self-management	Patient empowerment
self-management	Patient empowerment
rapport	Patient-HCP
support	Clinical benefit
Behaviour change	Clinical benefit
Equitable access to care	Accessibility
Access to patient	Accessibility
Access to patient	Accessibility
Access to internet/Wifi	Accessibility
Access to care	Accessibility
Access to mobile phone	Accessibility
Access to mobile phone	Accessibility
Access to mobile phone	Accessibility
Contact info	Adaptability
	Adaptability

Clinical goals/targets	Alignment
International/national plans	Alignment
Literacy	Capability
with patients	Co-design
with HCPs	Co-design
Education	Demographics
Socioeconomic status	Demographics
Evidence-based	Evidence-based
phone coverage	Financing/cost
Network	ICT infrastructure
workflow	Integration
Commitment	Leadership
Documentation	Mis (use)
Prioritizing	Motivation
	Motivation
rapport	Patient-HCP
	Policies and laws
Network incentives	Policies and laws
Organization	Trustworthiness
Convenience (patients)	Usability
User-friendly	Usability
User-friendly	Usability
	work infrastructure
Supportive (across clincs)	work infrastructure
	Workload
Follow-up	Workload
Time	Workload
Time	Workload
Documentation	Workload
Accuracy of health information	Trustworthiness
Health literacy	Capability
Identification	Clinical benefit
among HCPs	Collaboration
Shared-decision making	Patient-focused care
Fatigue	Psychological
Guidelines	Accuracy of DHI content
GP-specific tailored	Adaptability

Co-morbidities	Adaptability
Age	Appropriate
Health literacy	Appropriate
Medical history	Appropriate
	Autonomy
HCP responsibility	Autonomy
NPHWs	Autonomy
Health literacy	Capability
Among HCPs	Collaboration/connectivity
Rural or remote areas	Demographics
(Over)reliance	Mis (use)
HCP reassurance	Patient-HCP
Time	Work infrastructure
Time	work infrastructure
Staffing	work infrastructure
Time	Workload
Comfort (patients)	Usability
Efficiency	Clinical benefit
Efficiency	Clinical benefit
Efficiency	Clinical benefit
Rapport	Patient-HCP
	Patient empowerment
Anxiety	Psychological
Simplicity/efficiency (care)	Patient-focused care
Access to care	Accessibility
Waiting time	Workload
Hospital visits	Workload
Self-management	Patient empowerment
Responsiveness	Patient-focused care
Waiting time	Workload
Time	Workload
Access to patient data	Accessibility
Accuracy	Accuracy of DHI content
Work schedule (patients)	Adaptability
Medical history	Appropriate
Old age	Appropriate
old age	Appropriate

	Automation
Digital literacy	Capability
Health literacy	Capability
work status	Demographics
Old age	Demographics
Technical issues	Technical stability
Connectivity	Technical stability
Familiarity	Experiences
Data	Integration
Improper	Mis (use)
Ignoring	Mis (use)
Cooperation	Mis (use)
Regulations (privacy and data secu	Policies and laws
Confidentiality	Privacy
Confidence	Psychological
Increased responsibility	Role/responsibilities
	Social support
Cultural norms	sociocultural
Interest	Technology attitudes
Comfortable	Technology attitudes
Reliability	Trustworthiness
User-friendly for patient	Usability
User-friendly for patient	Usability
Simplicity (patients)	Usability
User-friendly	Usability
Simplicity	Usability
User-friendly	Usability
Scheduling	work infrastructure
Delineating Responsibilities/roles	work infrastructure
Cross-sectional collaboration	work infrastructure
Staffing	work infrastructure
Time	work infrastructure
Delineating Responsibilities/roles	work infrastructure
Time	work infrastructure
Scheduling	work infrastructure
Staffing (expertise)	work infrastructure
Sharing	work infrastructure

Supportive (within clinic)	work infrastructure
	workload
Time	Workload
Reviewing	Workload
Time	Workload
Juggling tasks	Workload
Interaction	Workload
	Workload
	Workload
Waiting time	Workload
Follow-up	Workload
	Workload
	Workload
Past habits	Experiences
Quality-of-care	Clinical benefit
Rapport	Patient-HCP
Reassurance	Patient-focused care
Self-efficacy	Patient empowerment
Access to care (rural areas)	Accessibility
Access to internet/Wifi	Accessibility
Access to mobile phone	Accessibility
Objective	Accuracy of DHI content
Cultural norms	Adaptability
With patients	Co-design
Quality checks (DHT)	Content
Real-time	Data
Rural or remote areas	Demographics
Socioeconomic status	Demographics
poor tech	Technical stability
Catastrophes	Environment
Familiarity	Experiences
Internet	Financing/cost
Funding	Financing/cost
Touch	Human aspect
Systems	ICT infrastructure
Systems	ICT infrastructure
Ready-to-use	ICT infrastructure

Equipment	ICT infrastructure
Systems	ICT infrastructure
Program champions (HCPs)	Leadership
Expectations	Misunderstanding
Financial incentives	Motivation
	Motivation
Perceived value	Motivation
	Patient safety
Communication	Patient-HCP
space	Physical space/infrastructure
Ready-to-use	Physical space/infrastructure
Policy (equity)	Policies and laws
Anxiety	Psychological
Anxiety	Psychological
Patients	Social support
Indigenous groups	sociocultural
Interest	Technology attitudes
Reluctance/resistance	Technology attitudes
Interest	Technology attitudes
Interest	Technology attitudes
Tech support	Training/Educating
Training	Training/Educating
Hybrid	Type
User-friendly	Usability
Workflow	Work infrastructure
Resources	Work infrastructure
Supportive (within clinic)	work infrastructure
Staffing	work infrastructure
Guidelines/protocols	work infrastructure
Cross-sectional collaboration	work infrastructure
Supportive (across clinics)	work infrastructure
Locally-based staff	work infrastructure
Centralization	work infrastructure
Cross-sectional collaboration	work infrastructure
Standardization (training)	work infrastructure
Standardization	work infrastructure
Guidelines/protocols	work infrastructure

Technical support	Workload
Time	Workload
Adherence	Clinical benefit
Comprehensive	Clinical benefit
Quality-of-care	Clinical benefit
Quality-of-care	Clinical benefit
Reassurance	Patient-focused care
Reassurance	Patient-focused care
Patients	Social support
Health outcomes	Clinical benefit
Personalized	Adaptability
Clinical goals/targets	Alignment
Digital literacy	Capability
Digital literacy	Capability
Technical issues	Technical stability
Technical issues	Technical stability
Technical issues	Technical stability
Internet/WIFI	ICT infrastructure
Perceived value	Motivation
Familiarity	Patient-HCP
Time patient	Training/Educating
Quality-of-care	Trustworthiness
Video visits	Type
Image size	Visualization
Technical support	Workload
Time	Workload
Management (patient capacity leve	Workload
Time	Workload
Access to care	Accessibility
Access to patient data	Accessibility
Self-management	patient empowerment
Self-management	patient empowerment
Decision-making	Patient empowerment
Shared-decision making	Patient-focused care
Support	Patient-focused care
Sustainability of BC	Patient empowerment
screening	Clinical benefit

Management	Clinical benefit
Behaviour change	Clinical benefit
[illegible]	Accessibility
Personalized	Adaptability
Patient needs	Adaptability
Patient preferences	Adaptability
Personalized	Adaptability
	Automation
Alerting	Automation
Digital literacy	Capability
Digital literacy	Capability
Digital literacy	Capability
Among HCPs	Collaboration/connectivity
Feedback	Communication
sharing	Data
Sharing	Data
Old age	Demographics
old age	demographics
Old age	Demographics
Evidence-based	Evidence-based
Evidence-based	Evidence-based
	Experiences
	Financing/cost
Workflow	Integration
Workflow	Integration
	Misunderstanding
Expectations	Misunderstanding
Expectations	Misunderstanding
Prioritizing	Motivation
Data sharing	Patient-HCP
Communication	Patient-HCP
Privacy	Privacy
Family	Social support
Family	Social support
Data security/privacy	Trustworthiness
Contact person	work infrastructure
user-friendly	Usability

Self-management	Patient empowerment
Self-management	Patient empowerment
	Motivation
Access to technology	Accessibility
Access to technology	Accessibility
Personalized	Adaptability
Personalized	Adaptability
Patient preferences	Adaptability
HCP preferences	Adaptability
Patient abilities	Adaptability
Personalized	Adaptability
Patient needs	Adaptability
Medical history	Appropriate
Medical history	Appropriate
With patients	Co-design
Family	Co-design
Mental health	Content
Self-management	Content
Mental health	Content
old age	demographics
Severity	Demographics
Workflow	Integration
Communication	Misunderstanding
Communication	Misunderstanding
	Motivation
	Motivation
	Motivation
Timely delivery	Process
Timely introduction	Process
Fatigue	Psychological
Fatigue	Psychological
Unclear roles	work infrastructure
Family	Social support
Addition	Technology attitudes
Training	Training/Educating
Training	Training/Educating
Training	Training/Educating
User-friendly for patient	Usability

Structured	Usability
Simplicity (patients)	Usability
Guidelines/protocols	work infrastructure
Time	work infrastructure
Time	Work infrastructure
Cross-sectional collaboration	work infrastructure
Organization	work infrastructure
Cognitive load	Workload
Burden	Workload
Efficiency	clinical benefit
Treatment	clinical benefit
Quality-of-care	clinical benefit
Stress	Psychological
Burden	Psychological
Equipment installation	Financing/cost
EHS	ICT infrastructure
Health insurance incentives	Motivation
Privacy	Privacy
Sceptical (general)	Technology attitudes
Simplicity	Usability
User-friendly	Usability
User-friendly for patient	Usability
Workflow	work infrastructure
Capacity	work infrastructure
	Workload
Capacity	Workload
Capacity	Workload
	Workload
Educating	Training/Educating
treatment	Clinical benefit
Communication	Patient-HCP
Access to technology	Accessibility
Mobile phone	Accessibility
Personalized	Adaptability
Patient needs	Adaptability
Modification	Autonomy
Selective use	Autonomy

digital literacy	capability
Self-management	Content
old age	demographics
Feedback	Communication
Existing systems	Integration
Rapport	patient-HCP
Interaction	Patient-HCP
Communication (messaging)	Patient-HCP
Rapport	Patient-HCP
patient contact responsibility	Role/responsibilities
Training	Training/Educating
Seeing	Training/Educating
User-friendly	Usability
Simplicity (patients)	Usability
User-friendly for patient	Usability
Graphical representation	Visualization
Sharing	work infrastructure
Time	Workload
Click-ability (patients)	Usability
Training	Training/Educating
Installing steps (patients)	Usability
Simplicity (patients)	Usability
Efficiency	Clinical benefit
Training	Training/Educating
Burden	Workload
Contextual information	Content
Optimizing	Clinical benefit
Support	Clinical benefit
Reassurance	Patient-focused care
Multiple systems	Workload
False alarms	Accuracy of DHI content
Co-morbidities	Adaptability
Patien needs	Adaptability
Patient needs	Adaptability
Multiple users	Adaptability
Health concern/problem	Adaptability
patient values	Automation

Health	Autonomy
Clinical testing	Human aspect
Workflow	Integration
EHR	Integration
Multiple software	Integration
Information overlooked	Mis (use)
Listening	Mis (use)
(Over)reliance	Mis(use)
Misinterpret	Misunderstanding
Expectations	Misunderstanding
Documentation	Misunderstanding
Self-management	Motivation
Patient contact responsibility	Role/responsibilities
Tool	Technology attitudes
Sceptical (accuracy)	Technology attitudes
Sceptical (ability)	Technology attitudes
Concerns	Technology attitudes
Sceptical (ability)	Technology attitudes
Tool	Technology attitudes
Accuracy	Trustworthiness
Click-ability	Usability
sharing	work infrastructure
redundancy	Workload
	Workload
Workflow	Workload
Insurance companies	Policies and laws
Retention/ Relative advantage	Clinical benefit
Stress	Psychological
Medical history	Appropriate
regulations	Awareness
Reimbursement	Awareness
Years of work experience	Demographics
Non-native speaker	Demographics
Old age	Demographics
rural or remote areas	Demographics
Sensory constraints	Demographics
Old age	Demographics

Technical issues	Technical stability
Evidence-based	Evidence-based
Concerns	Financing/cost
Expectation differences	Financing/cost
clinical testing	Human aspect
Clinical testing	Human aspect
EHR	Integration
Authority	Leadership
Malpractice	Mis (use)
Willingness	Motivation
Willingness	Motivation
Willingness	Motivation
Reimbursement	Policies and laws
Insurance companies	Policies and laws
Inequities	Policies and laws
Regulatory	Policies and laws
Reimbursement	Policies and laws
Legal factors	Policies and laws
Insurance companies	Policies and laws
Reimbursement	Policies and laws
Not first-encounter	Technology attitudes
Interest	Technology attitudes
training	training/educating
Prior to appointment	training/educating
sharing	work infrastructure
	Work infrastructure
Literacy levels	Adaptability
Medical decision making	Clinical benefit
Accuracy	Clinical benefit
Diagnosis	clinical benefit
Accuracy	clinical benefit
Medical decision making	Clinical benefit
Comprehensive	Clinical benefit
Health outcomes	Clinical benefit
Quality-of-care	Clinical benefit
Rapport	Patient-HCP
Relationship	Patient-HCP

Rapport	Patient-HCP
Multiple systems	Workload
Digital literacy	Appropriate
Continuous collection	Data
Clinical testing	Human aspect
Visual cues	Human aspect
Clinical testing	Human aspect
Non-verbal	Human aspect
EHR	Integration
Existing systems	Integration
Familiarity	Patient-HCP
Trust	Patient-HCP
Increased responsibility	Role/responsibilities
Family	Social support
Informal caregivers	Social support
HCP expertise	Trustworthiness
User-friendly for patient	Usability
Trends	Visualization
Staffing (expertise)	work infrastructure
Contact person	work infrastructure
Staffing (expertise)	work infrastructure
Supportive (within clinic)	work infrastructure
NPHW	work infrastructure
Scheduling	work infrastructure
Capacity	Workload
Responding	Workload
Quality-of-care	Clinical benefit
Informal caregivers	Social support
Medical decision making	clinical benefit
Medical decision making	clinical benefit
Medical decision making	Clinical benefit
Prevention	Clinical benefit
Support	Patient-focused care
Access to care (rural areas)	Accessibility
Accuracy	Accuracy of DHI content
Patient age	Adaptability
Health concern/problem	Adaptability

Personalized	Adaptability
Interaction frequency	Adaptability
Patient needs	Adaptability
Patient preferences	Adaptability
Language	Adaptability
Tailoring alerts	Adaptability
Modification	Autonomy
Tailoring data-sharing options	Autonomy
Tailoring data-sharing options	Autonomy
Feedback	Communication
Language	Demographics
Technical issues	Technical stability
Reminders	Feedback
	Gamification
Reward system	Gamification
Clinical testing	Human aspect
Touch	Human aspect
incentivization	Incentivization
Perceived value	Motivation
Communication	Patient-HCP
Informal caregivers	Social support
Informal caregivers	Social support
Clinical capabilities	Technology attitudes
Familiarity	Training/Educating
User-friendly	Usability
Trends	Visualization
Data management	Visualization
Amount/readability	Visualization
Easy/quick	Visualization
Defined roles	work infrastructure
	Workload
Equitable access to technology	Accessibility
Validating	Accuracy of DHI content
Patient (individualised medication) (	Adaptability
Among HCPs	Collaboration
Concerns	Financing/cost
staff compensation	Financing/cost

Workflow	Integration
Privacy	Privacy
patient contact responsibility	Role/responsibilities
liability	role/responsibilities
Accuracy of health information	Trustworthiness
Supportive	work infrastructure
perceived value	Motivation
Access to patient data	Accessibility
Diagnosis	Clinical benefit
Identification	Clinical benefit
Patient understanding	Patient-HCP
TV	Advertising
	Financing/cost
	Financing/cost
Authority	Leadership
Cooperation	Mis(use)
perceived value	Motivation
Guidelines	Policies and laws
Patient privacy	Privacy
Peer pressure	Social influence
Peer pressure	Social influence
Peer pressure	Social/economic influence
Accuracy	Trustworthiness
Access to patient data	Accessibility
Access to patient data	Accessibility
Access to (health) resources	Accessibility
Access to (health) resources	Accessibility
Time	workload
Documentation	workload
Time	workload
Documentation	workload
Literacy levels	Adaptability
More content	Adaptability
Language	Adaptability
Filling-in	Automation
Documentation	Automation
Engaging	Motivation

perceived value	Motivation
Willingness	Motivation
perceived value	Motivation
Peer pressure	Social influence
Accuracy of health information	Trustworthiness
Accuracy of health information	Trustworthiness
User-friendly	Usability
Convenience	Usability
Simplicity	Usability
Interactive	Usability
Simplicity	Usability
Medical language (for patients)	Visualization
	Visualization
Summarizing	Visualization
Data	Visualization

Category	World Heart Federation categories: clinicians / healthcare provi
perceived benefit	C
facilitator	P
barrier	P
barrier	P
facilitator	T
barrier	T
barrier	C
barrier	HS
barrier	T
barrier	T
barrier	T
barrier	C
barrier	T
facilitator	C
barrier	T
barrier	C
perceived benefit	C
perceived benefit	C
Barrier	T
Barrier	C
Barrier	T
Barrier	T
Facilitator	C
Barrier	C
Barrier	C
Barrier	C
Facilitator	T
Barrier	T
Barrier	T
perceived benefit	C
barrier	C
facilitator	T
facilitator	T
facilitator	T
facilitator	T

facilitator	T
barrier	HS
barrier	HS
barrier	C
barrier	C
facilitator	P
facilitator	T
facilitator	T
facilitator	T
perceived benefit	C
perceived benefit	C
perceived benefit	C
barrier	P
barrier	P
barrier	P
barrier	C
barrier	P
barrier	T
barrier	T
barrier	C
barrier	P
barrier	P
barrier	P
facilitator	C
barrier	HS
Barrier	C
perceived drawback	C
barrier	P
barrier	P
barrier	P
Barrier	C
Barrier	C
facilitator	C
barrier	C
barrier	C
barrier	HS
barrier	HS

barrier	HS
barrier	HS
perceived benefit	C
perceived benefit	C
facilitator	T
barrier	C
barrier	T
facilitator	T
facilitator	T
barrier	T
facilitator	T
facilitator	T
barrier	C
Barrier	T
barrier	C
barrier	C
barrier	T
facilitator	T
facilitator	HS
barrier	C
barrier	C
barrier	C
facilitator	P
facilitator	C
barrier	C
barrier	C
barrier	C
facilitator	C
barrier	C
barrier	T
facilitator	T
facilitator	T
Barrier	T
Barrier	T
facilitator	T
facilitator	T
facilitator	T

barrier	T
facilitator	T
perceived drawback	P
perceived drawback	P
facilitator	C
barrier	P
barrier	C
barrier	P
barrier	P
barrier	C
barrier	P
facilitator	C
barrier	P
facilitator	C
facilitator	C
barrier	C
facilitator	T
perceived benefit	C
perceived benefit	P
perceived benefit	P
perceived benefit	P
perceived benefit	P
perceived benefit	C
perceived benefit	C
perceived drawback	C
Facilitator	P
Perceived benefit	P
Perceived benefit	P
Perceived benefit	P
Perceived benefit	P
Barrier	P
Barrier	T
Facilitator	T
Facilitator	T
Facilitator	T
Facilitator	T
Facilitator	T

Barrier	T
Barrier	P
barrier	P
barrier	P
Barrier	P
Barrier	C
Facilitator	HS
Barrier	C
perceived drawback	C
Facilitator	P
Facilitator	T
Facilitator	T
Facilitator	HS
barrier	P
Perceived benefit	C
perceived drawback	C
perceived drawback	C
Perceived benefit	C
Barrier	HS
Barrier	HS
facilitator	T
facilitator	C
Barrier	HS
facilitator	C
Facilitator	C
Barrier	C
facilitator	T
facilitator	HS
facilitator	HS
barrier	HS
Facilitator	HS
Barrier	C
perceived benefit	C
perceived drawback	C
perceived benefit	P
barrier	P
barrier	P

barrier	T
facilitator	C
facilitator	C
perceived benefit	C
perceived benefit	C
perceived benefit	C
perceived benefit	C
perceived benefit	C
perceived benefit	C
perceived benefit	C
perceived drawback	C
perceived drawback	C
perceived drawback	C
facilitator	T
barrier	P
barrier	P
barrier	P
barrier	T
facilitator	C
facilitator	P
Perceived benefit	P
Perceived benefit	C
Perceived benefit	P
Perceived benefit	P
Facilitator	C
Barrier	T
Barrier	HS
Facilitator	T
Barrier	P
barrier	C
perceived drawback	C
perceived drawback	C
Facilitator	P
Facilitator	T
Facilitator	T
Facilitator	T
Facilitator	HS

Facilitator	HS
Facilitator	HS
Facilitator	HS
Facilitator	HS
Facilitator	C
Perceived benefit	C
Perceived benefit	C
Perceived benefit	C
Perceived benefit	C
Perceived benefit	C
Perceived benefit	C
Perceived benefit	C
Perceived benefit	C
Perceived benefit	C
perceived drawback	C
perceived drawback	C
perceived drawback	C
perceived drawback	C
perceived drawback	C
perceived drawback	C
perceived benefit	P
barrier	P
barrier	C
barrier	C
barrier	P
barrier	P
barrier	P
barrier	P
barrier	P
barrier	T
barrier	P
barrier	P
barrier	P
barrier	C
barrier	P
Barrier	C
Barrier	C

barrier	HS
barrier	HS
perceived drawback	C
perceived benefit	P
perceived benefit	P
perceived drawback	C
perceived benefit	C
barrier	T
barrier	C
facilitator	C
barrier	C
barrier	T
barrier	T
barrier	C
barrier	C
barrier	C
barrier	C
barrier	C
perceived benefit	C
perceived benefit	C
perceived benefit	C
perceived benefit	C
perceived benefit	C
perceived benefit	P
facilitator	C
barrier	C
barrier	C
barrier	C
barrier	C
perceived benefit	P
barrier	T
facilitator	T
Facilitator	HS
barrier	HS
barrier	T
barrier	HS
facilitator	HS

perceived benefit	C
perceived benefit	C
perceived drawback	C
barrier	C
barrier	C
perceived drawback	C
Facilitator	P
Barrier	P
Barrier	P
Barrier	P
Barrier	P
Facilitator	T
Barrier	HS
Barrier	HS
Barrier	HS
Barrier	HS
Facilitator	HS
Facilitator	HS
Facilitator	P
Barrier	HS
Barrier	C
perceived benefit	C
perceived benefit	P
perceived benefit	P
Perceived drawback	C
Perceived drawback	P
Facilitator	T
Facilitator	T
Facilitator	T
Facilitator	T
Facilitator	T
Facilitator	T
Facilitator	HS
Perceived drawback	C
Perceived drawback	P
perceived benefit	P
Barrier	T

Barrier	T
facilitator	T
facilitator	C
facilitator	T
facilitator	T
facilitator	T
barrier	T
facilitator	HS
facilitator	T
barrier	C
barrier	C
facilitator	P
barrier	C
barrier	C
barrier	C
facilitator	C
Barrier	C
Barrier	C
barrier	C
Barrier	C
barrier	C
facilitator	HS
facilitator	HS
perceived benefit	C
perceived benefit	C
perceived benefit	C
perceived benefit	C
perceived drawback	C
perceived drawback	C
perceived benefit	P
perceived benefit	P
barrier	C
Facilitator	C
barrier	T
barrier	C
Facilitator	C
barrier	C

barrier	C
perceived drawback	P
facilitator	HS
barrier	C
barrier	C
barrier	P
barrier	C
barrier	P
barrier	T
barrier	T
barrier	T
barrier	HS
barrier	HS
facilitator	C
barrier	C
barrier	P
barrier	C
barrier	C
barrier	C
barrier	P
facilitator	C
barrier	HS
barrier	HS
facilitator	HS
facilitator	HS
facilitator	HS
facilitator	HS
facilitator	HS
barrier	C
perceived benefit	C
perceived benefit	P
barrier	T
barrier	HS
facilitator	C
barrier	HS
barrier	HS
barrier	HS

perceived benefit	P
perceived benefit	P
perceived benefit	C
barrier	T
barrier	T
barrier	T
barrier	T
barrier	C
barrier	T
Barrier	P
facilitator	HS
facilitator	HS
facilitator	C
barrier	C
barrier	C
barrier	HS
barrier	HS
barrier	HS
perceived benefit	C
perceived benefit	C
perceived benefit	C
perceived drawback	C
perceived drawback	C
perceived drawback	C
perceived drawback	C
perceived benefit	C
perceived benefit	C
Facilitator	P
Facilitator	P
Facilitator	T
barrier	T
barrier	T
Facilitator	T
Facilitator	T
Facilitator	T
Facilitator	T
Facilitator	T

barrier	T
barrier	T
Facilitator	T
barrier	P
barrier	P
barrier	C
barrier	C
Barrier	P
barrier	C
barrier	C
barrier	C
Facilitator	T
Facilitator	T
Facilitator	T
Facilitator	T
facilitator	T
facilitator	T
Facilitator	T
Facilitator	T
facilitator	T
Facilitator	T
Facilitator	C
perceived benefit	P
perceived drawback	P
perceived drawback	P
perceived benefit	C
perceived benefit	C
perceived benefit	C
perceived drawback	C
perceived benefit	C
perceived benefit	C
perceived benefit	C
perceived drawback	C
perceived drawback	C
perceived benefit	C
perceived benefit	P
Facilitator	C

Facilitator	C
perceived drawback	C
perceived drawback	C
Barrier	P
Barrier	P
Barrier	T
Barrier	T
Barrier	T
Barrier	T
Facilitator	C
Facilitator	C
Barrier	C
Barrier	P
Barrier	T
Barrier	T
Barrier	HS
Barrier	C
Barrier	P
Barrier	P
Barrier	C
Facilitator	C
Barrier	T
Barrier	HS
perceived drawback	C
facilitator	T
facilitator	T
Barrier	P
facilitator	T
Barrier	P
barrier	P
Barrier	P
facilitator	C
facilitator	HS
facilitator	HS
facilitator	P
facilitator	C
facilitator	P

facilitator	T
facilitator	T
facilitator	T
perceived benefit	C
Facilitator	HS
Perceived benefit	P
Perceived benefit	P
Perceived drawback	P
Perceived drawback	P
Perceived drawback	P
perceived drawback	P
Perceived drawback	C
Barrier	P
Barrier	P
Barrier	P
barrier	P
Barrier	P
Barrier	T
Barrier	T
Barrier	HS
Barrier	T
barrier	T
Barrier	HS
Barrier	HS
Barrier	HS
Barrier	HS
barrier	HS
Facilitator	P
barrier	P
Barrier	C
Barrier	C
Barrier	HS
Perceived drawback	C
Perceived drawback	C
perceived drawback	C
Perceived drawback	C
Perceived drawback	C

facilitator	T
perceived benefit	C
facilitator	T
barrier	P
facilitator	T
barrier	T
barrier	C
barrier	C
perceived benefit	C
perceived benefit	P
perceived benefit	P
perceived drawback	P
perceived drawback	C
perceived benefit	P
facilitator	T
Barrier	T
Barrier	C
perceived benefit	C
Barrier	C
perceived benefit	C
perceived benefit	C
Facilitator	P
Barrier	T
perceived drawback	C
Barrier	T
Barrier	T
Barrier	T
Barrier	P
perceived drawback	C
Barrier	T
Barrier	T
Barrier	T
Barrier	P
Barrier	P
Barrier	P
Barrier	P
barrier	C

Barrier	P
Barrier	P
Barrier	P
Barrier	P
Barrier	P
barrier	C
Facilitator	C
Facilitator	C
facilitator	HS
perceived drawback	C
facilitator	C
Facilitator	P
Barrier	P
Barrier	P
Barrier	P
Barrier	P
Barrier	P
facilitator	P
Facilitator	P
facilitator	C
Facilitator	C
Barrier	T
perceived benefit	C
perceived benefit	C
perceived benefit	C
perceived benefit	P
perceived drawback	C
perceived drawback	C
perceived benefit	P
perceived benefit	P
barrier	C
perceived drawback	C
Facilitator	T
Facilitator	C
Barrier	C
Barrier	T
Barrier	T

Barrier	T
Barrier	T
Barrier	HS
Barrier	HS
Barrier	HS
Barrier	HS
Barrier	C
Facilitator	C
Barrier	HS
Barrier	HS
Barrier	HS
Barrier	HS
Facilitator	HS
Barrier	HS
Barrier	HS
Facilitator	HS
Barrier	HS
Barrier	C
Barrier	C
perceived drawback	C
perceived drawback	C
perceived benefit	C
perceived benefit	C
perceived benefit	C
perceived benefit	P
perceived benefit	P
perceived benefit	P
perceived benefit	C
perceived benefit	C
perceived benefit	P
Barrier	C
Facilitator	P
Barrier	P
Barrier	P
Barrier	P
Barrier	P
Facilitator	T

Barrier	T
Facilitator	C
Facilitator	C
Facilitator	P
Facilitator	P
Facilitator	P
facilitator	P
Facilitator	P
Facilitator	P
Barrier	P
Barrier	T
Barrier	T
Facilitator	T
Barrier	P
Barrier	P
Facilitator	T
Facilitator	C
Barrier	C
Barrier	T
Facilitator	T
Barrier	T
Facilitator	HS
Facilitator	HS
Barrier	HS
Barrier	C
Barrier	C
Barrier	P
Facilitator	C
Facilitator	P
Facilitator	P
Barrier	P
Facilitator	P
Facilitator	C
Barrier	P
Facilitator	C
Facilitator	HS
Facilitator	HS

Facilitator	C
Facilitator	HS
Barrier	P
Barrier	P
Facilitator	C
Facilitator	C
Barrier	C
Facilitator	C
facilitator	C
Facilitator	P
Facilitator	C
Facilitator	C
Facilitator	C
Facilitator	C
Facilitator	C
Facilitator	C
Facilitator	T
Facilitator	T
Barrier	T
Facilitator	T
Facilitator	T
Facilitator	T
Facilitator	T
Barrier	T
Barrier	T
Facilitator	T
Barrier	T
Barrier	HS
Facilitator	HS
Facilitator	HS
Facilitator	HS
Facilitator	HS
Facilitator	HS
Facilitator	HS
Barrier	HS
Barrier	HS
perceived benefit	C

perceived benefit	C
perceived benefit	C
perceived drawback	C
perceived drawback	C
perceived drawback	C
perceived benefit	C
perceived benefit	P
perceived benefit	P
perceived benefit	P
perceived benefit	P
perceived benefit	P
perceived benefit	P
perceived benefit	P
perceived benefit	P
perceived drawback	P
perceived drawback	P
perceived drawback	P
perceived drawback	C
perceived benefit	C
Facilitator	HS
perceived drawback	C
barrier	HS
Facilitator	HS
Facilitator	C
barrier	C
barrier	C
barrier	C
Facilitator	HS
Facilitator	HS
Facilitator	HS
Facilitator	HS
Facilitator	HS
Facilitator	HS
perceived drawback	C
Facilitator	C
Facilitator	C
Barrier	C

Barrier	C
barrier	C
perceived benefit	C
Facilitator	T
Facilitator	T
Barrier	T
Facilitator	C
Facilitator	HS
perceived drawback	C
Barrier	T
Barrier	C
Facilitator	T
Facilitator	HS
Barrier	T
Facilitator	C
Facilitator	HS
Barrier	T
Facilitator	HS
perceived drawback	
perceived drawback	
perceived drawback	C
perceived drawback	C
Facilitator	T
perceived benefit	C
Facilitator	T
Barrier	HS
Facilitator	T
Facilitator	T
Facilitator	HS
perceived drawback	C
Facilitator	C
Barrier	T
Facilitator	T
Facilitator	HS
Facilitator	HS
Facilitator	HS
perceived benefit	P

perceived benefit	P
perceived drawback	C
perceived drawback	C
perceived benefit	P
perceived benefit	C
Barrier	P
facilitator	T
Barrier	T
perceived benefit	C
perceived benefit	C
Facilitator	T
perceived benefit	C
Barrier	P
Barrier	P
Barrier	P
Facilitator	T
Facilitator	T
Barrier	T
Facilitator	T
Barrier	T
Barrier	P
perceived benefit	C
Facilitator	T
Facilitator	T
Barrier	P
Facilitator	T
Barrier	T
Facilitator	T
perceived benefit	C
Facilitator	T
Facilitator	HS
perceived benefit	C
perceived benefit	C
perceived drawback	C
Barrier	C
Barrier	P
Barrier	P

Barrier	
barrier	P
Facilitator	P
Barrier	P
Facilitator	C
Barrier	P
barrier	C
Facilitator	C
Facilitator	C
Barrier	HS
Barrier	P
Barrier	P
Facilitator	P
Facilitator	P
Facilitator	P
Facilitator	C
Barrier	P
Barrier	P
Facilitator	C
Barrier	C
Barrier	C
Facilitator	T
Barrier	T
Barrier	T
Barrier	T
Facilitator	T
Facilitator	T
Facilitator	T
Barrier	HS
Facilitator	HS
Facilitator	HS
Facilitator	HS
Barrier	C
perceived benefit	C
perceived benefit	C
perceived benefit	P
perceived benefit	P

perceived benefit	P
perceived benefit	P
perceived benefit	P
perceived benefit	P
perceived benefit	P
perceived drawback	P
perceived drawback	P
perceived benefit	P
perceived benefit	C
perceived benefit	C
perceived drawback	C
perceived drawback	C
Facilitator	T
perceived benefit	C
barrier	T
Facilitator	T
Facilitator	T
Facilitator	C
Facilitator	C
barrier	P
Facilitator	T
barrier	P
barrier	T
barrier	T
Facilitator	T
barrier	HS
barrier	P
barrier	C
barrier	P
Facilitator	T
barrier	T
Facilitator	T
barrier	T
perceived drawback	P
perceived benefit	P
perceived benefit	C
perceived drawback	C

perceived drawback	C
facilitator	T
facilitator	T
facilitator	T
barrier	C
barrier	T
barrier	P
perceived drawback	C
perceived benefit	P
perceived drawback	C
perceived drawback	C
Facilitator	T
Facilitator	T
Barrier	T
facilitator	C
facilitator	P
facilitator	P
Barrier	C
Barrier	C
facilitator	P
perceived benefit	C
barrier	P
barrier	P
barrier	P
barrier	P
barrier	P
barrier	T
barrier	P
barrier	T
barrier	T
barrier	P
barrier	P
facilitator	C
facilitator	C
barrier	C
barrier	HS
perceived drawback	C

barrier	P
barrier	P
barrier	C
perceived benefit	P
Perceived benefit	P
Perceived benefit	P
Perceived benefit	P
perceived benefit	P
Perceived benefit	P
Perceived benefit	P
Perceived benefit	P
Perceived benefit	C
perceived benefit	C
Perceived benefit	C
Perceived benefit	C
perceived benefit	C
Facilitator	T
Facilitator	T
facilitator	T
facilitator	T
Barrier	C
Facilitator	T
Facilitator	T
Facilitator	T
Facilitator	C
Barrier	P
Barrier	C
Facilitator	T
Facilitator	T
Facilitator	T
Barrier	P
Barrier	P
Facilitator	T
Barrier	HS
facilitator	T
Barrier	P
Facilitator	C

facilitator	HS
Facilitator	P
Facilitator	P
Barrier	P
Facilitator	P
facilitator	C
facilitator	T
Barrier	T
barrier	T
facilitator	T
Facilitator	HS
Facilitator	HS
Facilitator	HS
Barrier	P
Barrier	C
perceived benefit	C
perceived benefit	C
perceived benefit	C
perceived benefit	C
perceived benefit	C
perceived benefit	C
perceived benefit	C
perceived benefit	C
facilitator	T
barrier	T
barrier	HS
barrier	HS
barrier	T
barrier	T
facilitator	C
barrier	C
barrier	P
barrier	HS
perceived benefit	P
perceived benefit	P
perceived benefit	P
perceived benefit	P

perceived benefit	P
perceived drawback	P
perceived drawback	P
perceived benefit	C
perceived benefit	C
perceived drawback	C
facilitator	HS
perceived benefit	C
perceived benefit	C
perceived benefit	C
perceived benefit	C
perceived benefit	C
barrier	T
Barrier	T
barrier	P
barrier	P
facilitator	T
barrier	T
facilitator	T
barrier	T
facilitator	HS
barrier	P
facilitator	C
facilitator	C
facilitator	P
facilitator	P
facilitator	HS
facilitator	HS
barrier	HS
facilitator	HS
perceived benefit	C
facilitator	HS
facilitator	HS
perceived benefit	C
facilitator	HS
facilitator	P
Facilitator	C

facilitator	C
barrier	C
barrier	P
barrier	C
facilitator	T
facilitator	HS
barrier	HS
barrier	HS
barrier	C
perceived drawback	C
perceived drawback	C
perceived drawback	C
perceived benefit	P
Facilitator	C
facilitator	T
Perceived benefit	C
perceived benefit	C
perceived benefit	C
Perceived benefit	P
perceived benefit	P
Perceived benefit	C
Perceived benefit	C
Perceived benefit	C
Perceived benefit	C
perceived benefit	C
perceived benefit	C
perceived benefit	C
perceived benefit	C
Perceived benefit	C
Barrier	C
facilitator	T
facilitator	T
Facilitator	T
Barrier	T
Facilitator	T
facilitator	T
Facilitator	T

facilitator	C
facilitator	C
facilitator	T
facilitator	T
Facilitator	T
barrier	P
Barrier	P
facilitator	C
facilitator	C
Facilitator	C
facilitator	C
facilitator	C
Facilitator	C
facilitator	C
Barrier	P
facilitator	C
facilitator	T
facilitator	T
facilitator	T
facilitator	HS
perceived benefit	C
perceived drawback	C
facilitator	P
Barrier	P
facilitator	C
facilitator	T
Facilitator	T
Facilitator	T
facilitator	HS
facilitator	HS
Barrier	C
facilitator	C
perceived benefit	C
Perceived benefit	C
Perceived benefit	C
Perceived benefit	C
perceived benefit	C

perceived drawback	C
Barrier	HS
perceived benefit	C
Facilitator	T
Facilitator	C
Facilitator	T
Facilitator	T
Facilitator	T
Facilitator	P
Facilitator	P
Facilitator	C
Facilitator	T
Barrier	HS
Barrier	HS
Facilitator	T
Facilitator	T
Facilitator	P
facilitator	T
Facilitator	T
Facilitator	T
Barrier	T
Facilitator	HS
Barrier	HS
Facilitator	HS
Facilitator	HS
Facilitator	HS
Barrier	HS
perceived benefit	C
perceived benefit	C
perceived benefit	P
perceived benefit	P
perceived benefit	P
Facilitator	C
Perceived benefit	C
Perceived drawback	C
Perceived drawback	C
Perceived drawback	C

Perceived drawback	C
Facilitator	C
barrier	C
Facilitator	C
Barrier	T
Barrier	T
Barrier	T
Barrier	T
Barrier	T
barrier	C
Facilitator	C
Facilitator	C
Facilitator	C
Facilitator	C
Facilitator	C
Barrier	C
Barrier	C
Barrier	C
Barrier	C
Facilitator	C
Perceived drawback	C
Perceived drawback	C
Perceived drawback	C
Perceived drawback	C
perceived drawback	C
Perceived drawback	C
Perceived drawback	C
Perceived drawback	C
Perceived drawback	C
Perceived benefit	P
Perceived benefit	P
Perceived benefit	P
perceived benefit	P
Perceived benefit	P
Perceived benefit	P
Perceived benefit	P
Perceived drawback	C

perceived drawback	P
Perceived drawback	P
Perceived drawback	C
Perceived drawback	C
Facilitator	C
Facilitator	T
Facilitator	T
Facilitator	T
Facilitator	HS
Facilitator	T
Facilitator	T
Facilitator	C
Facilitator	T
Facilitator	P
perceived drawback	C
perceived drawback	C
Facilitator	P
Facilitator	P
Facilitator	P
Facilitator	P
Facilitator	C
Facilitator	C
Facilitator	T
Facilitator	T
Facilitator	HS
Facilitator	HS
Facilitator	HS
Facilitator	C
perceived drawback	C
perceived drawback	C
perceived drawback	C
facilitator	T
perceived benefit	P
perceived benefit	P
perceived benefit	P
perceived benefit	P
perceived benefit	C

perceived benefit	C
perceived benefit	C
perceived benefit	C
perceived drawback	C
facilitator	T
facilitator	HS
facilitator	T
barrier	C
barrier	T
barrier	T
facilitator	T
facilitator	T
facilitator	T
facilitator	T
facilitator	HS
barrier	HS
facilitator	HS
facilitator	P
barrier	C
facilitator	C
barrier	C
barrier	HS
facilitator	HS
perceived drawback	C
facilitator	C
facilitator	P
facilitator	C
facilitator	P
facilitator	P
facilitator	C
facilitator	T
barrier	T
barrier	T
facilitator	T
facilitator	T
facilitator	HS
facilitator	HS

Facilitator	HS
facilitator	HS
facilitator	HS
facilitator	HS
facilitator	HS
facilitator	HS
facilitator	HS
facilitator	HS
facilitator	HS
barrier	HS
barrier	HS
barrier	HS
facilitator	HS
barrier	C
barrier	C
barrier	C
perceived benefit	C
perceived drawback	C
perceived drawback	C
perceived drawback	C
Perceived benefit	P
Perceived benefit	P
Perceived benefit	P
Perceived benefit	P
Perceived benefit	P
Perceived benefit	P
perceived benefit	P
Perceived benefit	P
Perceived benefit	C
Perceived benefit	C
Perceived benefit	C
Perceived benefit	C
Perceived benefit	C
Facilitator	T
Facilitator	T
Barrier	T
Facilitator	T

Barrier	P
Barrier	P
Barrier	P
Facilitator	T
Barrier	C
Barrier	P
Barrier	C
Barrier	C
Barrier	C
Barrier	C
Facilitator	T
Facilitator	T
Barrier	T
Facilitator	T
Facilitator	T
Facilitator	T
Barrier	T
Barrier	T
facilitator	C
Perceived benefit	C
Perceived benefit	C
Perceived benefit	C
Facilitator	HS
Facilitator	HS
Barrier	P
Barrier	T
Barrier	T
Barrier	T
Barrier	C
Barrier	T
Barrier	T
Barrier	T
Facilitator	C
Barrier	HS
Barrier	HS
Barrier	HS
Barrier	T

Facilitator	HS
Facilitator	T
Barrier	P
Barrier	P
Facilitator	HS
Barrier	HS
Barrier	HS
Facilitator	HS
Facilitator	HS
Barrier	C
Facilitator	C
Facilitator	P
Facilitator	P
Facilitator	C
Barrier	C
Barrier	C
Barrier	C
Barrier	T
Facilitator	HS
Facilitator	HS
Facilitator	HS
Facilitator	HS
Facilitator	HS
Facilitator	HS
Barrier	HS
Barrier	HS
Barrier	HS
Barrier	HS
Barrier	HS
Facilitator	HS
Facilitator	HS
Facilitator	HS
perceived drawback	C
perceived drawback	C
perceived drawback	C
perceived drawback	C
perceived benefit	C

perceived benefit	C
perceived benefit	C
perceived drawback	C
perceived drawback	C
Perceived drawback	C
perceived benefit	C
Facilitator	P
Barrier	P
Facilitator	T
Facilitator	HS
Facilitator	T
Facilitator	T
Facilitator	T
facilitator	C
Facilitator	P
Barrier	HS
Facilitator	HS
Facilitator	HS
barrier	HS
Barrier	T
Facilitator	T
Barrier	HS
Facilitator	P
Facilitator	C
Barrier	C
Facilitator	HS
Facilitator	T
Barrier	C
Barrier	C
Facilitator	C
Facilitator	C
Facilitator	C
Facilitator	C
Facilitator	C
Facilitator	C
Barrier	P
Facilitator	T

Facilitator	T
Facilitator	T
Barrier	HS
Barrier	HS
Barrier	HS
Barrier	HS
Facilitator	HS
Facilitator	HS
Barrier	C
perceived benefit	C
Perceived drawback	P
perceived benefit	C
perceived drawback	C
perceived drawback	C
perceived drawback	C
Facilitator	T
perceived benefit	C
perceived benefit	C
perceived drawback	C
Barrier	T
barrier	T
Facilitator	T
barrier	T
Facilitator	C
Facilitator	T
Facilitator	T
barrier	C
barrier	C
barrier	C
barrier	C
barrier	C
Facilitator	C
perceived drawback	C
Facilitator	P
barrier	C
Facilitator	C

barrier	C
facilitator	C
facilitator	C
facilitator	C
Facilitator	P
barrier	T
Facilitator	T
Facilitator	T
Facilitator	T
barrier	T
barrier	HS
barrier	HS
barrier	HS
barrier	HS
barrier	HS
barrier	C
perceived drawback	C
perceived benefit	C
perceived benefit	C
perceived benefit	C
perceived benefit	C
perceived benefit	C
perceived benefit	P
perceived benefit	P
barrier	P
barrier	P
barrier	T
facilitator	P
barrier	P
barrier	P
barrier	P
barrier	T
barrier	T
facilitator	C
facilitator	HS
barrier	HS
barrier	HS

barrier	T
barrier	T
barrier	T
facilitator	HS
facilitator	HS
barrier	P
barrier	HS
facilitator	P
facilitator	P
facilitator	C
facilitator	C
barrier	HS
facilitator	HS
facilitator	HS
barrier	C
perceived benefit	C
Facilitator	C
Facilitator	C
perceived benefit	C
perceived benefit	C
Facilitator	C
Barrier	C
perceived benefit	C
Facilitator	C
Facilitator	C
Facilitator	T
perceived benefit	C
perceived benefit	P
perceived benefit	P
perceived benefit	C
perceived benefit	C
perceived benefit	C
Perceived benefit	C
barrier	C
barrier	P
barrier	P
barrier	T

barrier	P
barrier	HS
facilitator	T
barrier	P
barrier	C
barrier	C
barrier	C
barrier	C
facilitator	T
facilitator	HS
perceived benefit	P
barrier	HS
Facilitator	C
perceived benefit	C
Perceived benefit	C
Perceived benefit	C
Barrier	P
barrier	T
Barrier	T
facilitator	T
Barrier	T
Barrier	C
Facilitator	C
perceived drawback	C
perceived drawback	C
perceived drawback	C
Barrier	C
Barrier	P
Facilitator	C
Facilitator	C
Facilitator	C
Barrier	C
Facilitator	T
barrier	T
facilitator	T
Facilitator	HS
Barrier	HS
Barrier	HS

facilitator	HS
facilitator	HS
Barrier	C
Barrier	C
facilitator	C
Barrier	C
perceived drawback	C
perceived drawback	C
perceived drawback	C
perceived drawback	C
perceived drawback	C
perceived drawback	C
perceived drawback	C
Perceived benefit	P
Perceived benefit	P
Perceived benefit	P
Perceived benefit	P
Perceived benefit	P
Perceived benefit	P
Perceived benefit	C
Perceived benefit	C
Perceived benefit	C
Perceived benefit	C
Perceived benefit	C
facilitator	C
facilitator	C
Facilitator	T
Barrier	T
Facilitator	T
Facilitator	T
Facilitator	T
Facilitator	T
Facilitator	T
Facilitator	T
Facilitator	T
Barrier	C
Barrier	P

Facilitator	T
Facilitator	T
Facilitator	T
Barrier	P
Barrier	P
Barrier	P
Barrier	T
Facilitator	T
Barrier	T
Barrier	C
Barrier	T
Facilitator	T
Facilitator	T
Facilitator	T
Facilitator	C
Facilitator	HS
Barrier	P
Barrier	P
Barrier	P
Barrier	P
Facilitator	P
Facilitator	C
Barrier	C
Facilitator	T
Facilitator	T
Facilitator	HS
Facilitator	HS
Facilitator	HS
Perceived benefit	C
Perceived benefit	C
Perceived benefit	C
perceived drawback	C
facilitator	C
perceived benefit	C
perceived benefit	C
perceived benefit	C
perceived benefit	C

perceived benefit	C
facilitator	T
barrier	T
facilitator	T
barrier	T
facilitator	C
facilitator	T
barrier	P
Barrier	P
Barrier	P
facilitator	C
facilitator	C
perceived drawback	C
perceived drawback	C
perceived drawback	C
perceived drawback	C
facilitator	C
barrier	P
facilitator	T
facilitator	T
facilitator	HS
perceived drawback	C
perceived benefit	P
perceived benefit	C
perceived benefit	C
perceived benefit	C
perceived benefit	C
perceived benefit	C
barrier	P
Facilitator	P
barrier	P
barrier	P
Barrier	T
Barrier	HS
Barrier	T
Facilitator	C
barrier	C

Facilitator	C
Barrier	P
Barrier	C
Barrier	T
Barrier	C
Barrier	C
Facilitator	C
Barrier	C
Barrier	C
Barrier	C
Barrier	C
perceived benefit	C
Facilitator	T
Barrier	C
perceived benefit	C
perceived benefit	C
perceived benefit	C
perceived benefit	C
Facilitator	C
Barrier	C
Barrier	C
Barrier	P
Facilitator	P
Barrier	T
Barrier	C
Barrier	T
Barrier	C
Barrier	P
Barrier	C
Barrier	C
Barrier	C
Barrier	T
perceived benefit	C
perceived benefit	C
perceived benefit	P
perceived benefit	P
perceived benefit	P

perceived benefit	P
perceived benefit	P
perceived benefit	C
perceived benefit	C
perceived benefit	C
facilitator	T
facilitator	C
facilitator	C
barrier	T
facilitator	T
facilitator	P
Barrier	HS
barrier	C
barrier	C
barrier	HS
facilitator	HS
barrier	HS
barrier	HS
barrier	T
barrier	P
facilitator	P
barrier	C
barrier	HS
barrier	HS
barrier	HS
barrier	HS
barrier	HS
facilitator	C
facilitator	HS
facilitator	C
facilitator	C
facilitator	P
facilitator	C
barrier	T
facilitator	HS
perceived drawback	C
perceived drawback	C

Barrier	T
Barrier	T
perceived drawback	C
Barrier	C
barrier	P
perceived benefit	C
perceived benefit	C
facilitator	T
barrier	T
facilitator	T
facilitator	T
barrier	T
Barrier	HS
facilitator	HS
barrier	C
facilitator	T
facilitator	T
perceived benefit	P
facilitator	T
barrier	T
barrier	T
barrier	T
Facilitator	C
facilitator	P
barrier	P
facilitator	T
facilitator	T
barrier	C
barrier	C
barrier	C
barrier	P
facilitator	P
barrier	P
barrier	C
barrier	T
barrier	T
barrier	T

barrier	T
barrier	HS
barrier	HS
barrier	C
perceived benefit	P
Facilitator	HS
Barrier	C
Facilitator	HS
Facilitator	C
Facilitator	C
Facilitator	HS
perceived drawback	C
perceived benefit	C
perceived drawback	C
perceived benefit	P
perceived drawback	C
perceived benefit	P
Facilitator	T
Facilitator	T
Facilitator	T
Facilitator	T
Facilitator	T
barrier	T
barrier	T
barrier	C
barrier	C
barrier	C
barrier	C
barrier	C
barrier	C
Facilitator	HS
barrier	T
barrier	P
barrier	T
barrier	HS
Facilitator	T
perceived benefit	P

perceived benefit	P
perceived benefit	P
perceived benefit	P
perceived benefit	C
perceived benefit	C
perceived benefit	C
Facilitator	C
Facilitator	C
barrier	P
facilitator	T
barrier	T
Facilitator	HS
Facilitator	HS
Facilitator	C
Facilitator	C
Facilitator	C
Facilitator	C
Facilitator	C
Facilitator	C
Facilitator	HS
Facilitator	P
Facilitator	P
perceived benefit	C
barrier	T
perceived benefit	C
perceived drawback	C
perceived benefit	C
perceived benefit	C
perceived drawback	C
barrier	P
barrier	P
barrier	P
barrier	C
barrier	C
Facilitator	C
barrier	P
barrier	P

barrier	P
barrier	P
barrier	T
Facilitator	HS
barrier	T
barrier	T
Facilitator	HS
barrier	HS
Facilitator	HS
barrier	HS
barrier	HS
barrier	P
Facilitator	C
Facilitator	HS
barrier	P
barrier	P
barrier	C
barrier	C
facilitator	T
barrier	T
Barrier	T
Facilitator	T
Facilitator	T
Facilitator	HS
Barrier	HS
Facilitator	HS
facilitator	HS
facilitator	HS
perceived drawback	C
perceived benefit	C
perceived benefit	C
perceived benefit	P
perceived benefit	P
barrier	T
facilitator	T
facilitator	T
facilitator	T

facilitator	T
barrier	C
Barrier	P
barrier	P
barrier	P
barrier	P
facilitator	T
barrier	T
barrier	T
barrier	T
facilitator	C
facilitator	C
barrier	C
facilitator	P
facilitator	P
perceived drawback	P
perceived benefit	C
perceived benefit	C
perceived benefit	C
perceived benefit	C
barrier	T
barrier	T
barrier	T
Facilitator	T
facilitator	T
barrier	C
perceived benefit	C
facilitator	P
barrier	P
barrier	HS
barrier	HS
barrier	HS
facilitator	HS
barrier	T
barrier	P
barrier	HS
barrier	HS

barrier	HS
barrier	P
barrier	C
barrier	C
perceived benefit	P
perceived benefit	P
perceived benefit	P
perceived benefit	P
perceived benefit	P
perceived benefit	P
perceived benefit	P
perceived benefit	C
perceived benefit	C
perceived benefit	C
Perceived benefit	C
Perceived benefit	P
Perceived benefit	P
Perceived benefit	C
Perceived benefit	C
Perceived benefit	C
Facilitator	P
Facilitator	P
Facilitator	C
Facilitator	C
Facilitator	T
perceived drawback	C
perceived drawback	C
perceived benefit	C
perceived benefit	C
perceived benefit	C
Facilitator	C
Barrier	T
Barrier	T
barrier	T
Barrier	T
Facilitator	HS
Barrier	HS

Facilitator	HS
Barrier	C
Barrier	C
perceived drawback	C
facilitator	HS
barrier	C
barrier	C
barrier	P
barrier	P
barrier	P
facilitator	T
facilitator	T
barrier	P
facilitator	T
barrier	T
barrier	P
facilitator	HS
barrier	T
barrier	HS
barrier	C
barrier	C
barrier	HS
barrier	HS
barrier	P
facilitator	C
facilitator	P
barrier	C
facilitator	T
facilitator	T
barrier	HS
barrier	C
perceived benefit	P
Facilitator	C
perceived benefit	C
perceived benefit	C
perceived benefit	C
perceived benefit	C

perceived benefit	C
Facilitator	C
Facilitator	T
Facilitator	T
barrier	T
Facilitator	T
barrier	T
Facilitator	C
Facilitator	C
Facilitator	C
Facilitator	C
Facilitator	T
Facilitator	T
Facilitator	C
Facilitator	T
Facilitator	C
Facilitator	T
barrier	C
barrier	C
barrier	C
Facilitator	C
Facilitator	C
Barrier	C
barrier	C
Facilitator	C
Facilitator	C
Facilitator	C
Facilitator	C
Facilitator	T
Facilitator	T
Facilitator	T
Facilitator	T
perceived benefit	C
facilitator	T
perceived benefit	C
perceived benefit	C
barrier	T

barrier	T
facilitator	T
facilitator	T
barrier	C
barrier	C
barrier	C
facilitator	C
facilitator	T
facilitator	HS
barrier	C
Facilitator	T
perceived benefit	C
perceived benefit	C
perceived benefit	C
perceived benefit	C
perceived benefit	C
perceived benefit	C
perceived benefit	C
perceived benefit	C
facilitator	C
Facilitator	T
Facilitator	T
Facilitator	T
Facilitator	T
barrier	C
barrier	C
barrier	C
Facilitator	C
Facilitator	C
Facilitator	C
Facilitator	C
perceived benefit	C
barrier	C
Facilitator	T
Facilitator	T
Facilitator	T
Facilitator	HS

Facilitator	HS
perceived benefit	C
perceived benefit	C
perceived benefit	C
perceived benefit	P
Facilitator	HS
perceived benefit	P
Barrier	P
Barrier	P
Barrier	T
Barrier	T
Barrier	T
Facilitator	T
Barrier	T
Facilitator	C
Barrier	P
Facilitator	T
facilitator	C
Facilitator	C
Facilitator	P
Barrier	T
Facilitator	HS
perceived benefit	P
facilitator	C
perceived benefit	C
facilitator	C
perceived benefit	C
Facilitator	HS
perceived benefit	C
perceived drawback	P
perceived drawback	P
Facilitator	C
Facilitator	C
barrier	T
barrier	T
Facilitator	P
Facilitator	C

barrier	C
barrier	C
Barrier	HS
Barrier	T
Barrier	T
Barrier	T
Barrier	T
Barrier	HS
Barrier	HS
Facilitator	HS
perceived benefit	C
Perceived benefit	P
Perceived benefit	P
Facilitator	C
Facilitator	C
Facilitator	C
perceived benefit	C
facilitator	C
perceived benefit	C
perceived benefit	C
perceived benefit	C
perceived drawback	C
perceived benefit	P
perceived benefit	P
perceived benefit	P
perceived benefit	P
perceived benefit	P
perceived benefit	P
perceived benefit	P
perceived benefit	P
perceived benefit	P
perceived drawback	P
perceived drawback	P
perceived drawback	P
perceived drawback	P
perceived benefit	C
perceived benefit	C

perceived benefit	C
perceived benefit	C
perceived drawback	C
perceived drawback	C
perceived benefit	C
perceived benefit	C
perceived benefit	C
facilitator	C
barrier	C
Perceived drawback	C
perceived drawback	C
perceived drawback	C
perceived drawback	C
perceived drawback	C
perceived drawback	C
perceived drawback	C
perceived drawback	C
perceived drawback	C
perceived drawback	C
perceived drawback	C
barrier	P
barrier	P
barrier	C
barrier	P
barrier	P
barrier	T
barrier	T
facilitator	P
barrier	P
barrier	P
barrier	P
barrier	P
barrier	P
barrier	P
barrier	P
facilitator	HS
Facilitator	T

barrier	T
facilitator	T
Facilitator	C
barrier	P
Facilitator	HS
facilitator	HS
facilitator	HS
Facilitator	C
facilitator	C
facilitator	T
Facilitator	T
Facilitator	T
Facilitator	T
Barrier	HS
facilitator	HS
facilitator	HS
facilitator	HS
facilitator	HS
barrier	HS
barrier	HS
Barrier	HS
perceived drawback	C
perceived benefit	P
perceived benefit	C
perceived benefit	C
Perceived benefit	C
Perceived benefit	C
barrier	P
barrier	T
barrier	T
barrier	T
barrier	C
facilitator	T
facilitator	T
facilitator	T
barrier	T
barrier	T

barrier	T
barrier	T
perceived drawback	C
facilitator	C
barrier	T
barrier	T
barrier	T
facilitator	HS
Perceived benefit	C
perceived drawback	C
perceived drawback	C
perceived drawback	C
facilitator	T
Barrier	T
facilitator	T
barrier	HS
barrier	T
Barrier	HS
Barrier	T
perceived benefit	C
perceived benefit	C
perceived benefit	C
perceived benefit	C
perceived benefit	C
perceived benefit	C
perceived benefit	C
perceived benefit	C
facilitator	C
facilitator	C
barrier	P
barrier	P
barrier	C
barrier	P
barrier	P
facilitator	C
barrier	P
facilitator	C

facilitator	HS
facilitator	C
facilitator	C
Facilitator	C
facilitator	P
barrier	C
Facilitator	T
facilitator	HS
barrier	HS
perceived benefit	P
perceived benefit	P
perceived benefit	P
perceived benefit	P
perceived benefit	P
perceived benefit	P
perceived drawback	C
barrier	P
facilitator	P
facilitator	T
facilitator	T
facilitator	T
facilitator	T
facilitator	T
facilitator	T
facilitator	T
facilitator	P
barrier	P
facilitator	T
facilitator	T
facilitator	T
facilitator	T
facilitator	T
barrier	P
facilitator	T
facilitator	T
facilitator	T
facilitator	T

facilitator	T
barrier	T
facilitator	T
facilitator	P
facilitator	C
facilitator	C
facilitator	HS
barrier	P
facilitator	P
facilitator	P
facilitator	P
facilitator	P
facilitator	P
facilitator	P
barrier	P
barrier	P
facilitator	P
facilitator	P
facilitator	P
barrier	P
facilitator	C
facilitator	C
facilitator	T
facilitator	T
facilitator	T
facilitator	T
facilitator	T
facilitator	T
facilitator	T
facilitator	HS
Barrier	C
Facilitator	C
Facilitator	C
Facilitator	C
Facilitator	C
Facilitator	C
Facilitator	C

Facilitator	C
Facilitator	P
Facilitator	C
Facilitator	C
Facilitator	C
Facilitator	C
Facilitator	C
Facilitator	P
Barrier	C
Facilitator	T
perceived benefit	P
barrier	P
Facilitator	T
Facilitator	T
Facilitator	T
Facilitator	T
Facilitator	T
Facilitator	C
Facilitator	C
Facilitator	P
barrier	P
barrier	P
barrier	P
barrier	T
Facilitator	T
barrier	T
barrier	T
barrier	T
Facilitator	T
barrier	P
barrier	C
barrier	C
Facilitator	T
Facilitator	T
Facilitator	T
Facilitator	T
barrier	HS

barrier	HS
perceived drawback	C
Barrier	C
Barrier	C
Barrier	P
Barrier	T
Barrier	T
Barrier	HS
Barrier	P
Barrier	HS
Barrier	HS
Barrier	C
Barrier	C
perceived benefit	C
perceived benefit	C
perceived benefit	P
perceived benefit	P
perceived benefit	P
perceived benefit	P
perceived benefit	P
perceived benefit	P
Barrier	T
facilitator	C
barrier	P
facilitator	C
barrier	T
facilitator	HS
barrier	HS
barrier	HS
barrier	HS
facilitator	P
facilitator	HS
facilitator	T
facilitator	T
facilitator	T
facilitator	HS
facilitator	P

facilitator	P
facilitator	C
facilitator	HS
facilitator	HS
barrier	HS
facilitator	P
facilitator	P
facilitator	C
facilitator	C
facilitator	C
facilitator	P
facilitator	HS
facilitator	HS
facilitator	HS
facilitator	HS
facilitator	HS
perceived benefit	C
perceived drawback	C
perceived drawback	C
perceived drawback	C
facilitator	HS
Barrier	P
Barrier	P
Barrier	P
facilitator	T
facilitator	T
facilitator	C
Barrier	P
facilitator	T
Barrier	C
facilitator	C
facilitator	T
Barrier	C
Barrier	P
Barrier	P
Barrier	P
Barrier	P

facilitator	HS
facilitator	HS
Barrier	HS
facilitator	HS
Barrier	HS
facilitator	C
facilitator	T
facilitator	T
facilitator	T
Barrier	T
facilitator	HS
Barrier	HS
Barrier	P
facilitator	C
Barrier	HS
facilitator	P
facilitator	C
facilitator	C
facilitator	C
Barrier	C
facilitator	HS
facilitator	HS
Facilitator	HS
facilitator	HS
facilitator	HS
Barrier	HS
Barrier	HS
Barrier	HS
barrier	HS
Barrier	HS
facilitator	HS
Barrier	C
perceived drawback	C
perceived drawback	C
facilitator	C
perceived benefit	C
perceived benefit	C

perceived benefit	C
perceived benefit	P
perceived benefit	P
perceived benefit	P
perceived benefit	P
perceived benefit	P
perceived benefit	P
perceived benefit	P
perceived benefit	P
perceived benefit	P
perceived benefit	P
perceived benefit	P
perceived drawback	C
perceived benefit	C
facilitator	T
facilitator	P
barrier	P
barrier	T
barrier	P
facilitator	T
barrier	T
barrier	HS
barrier	HS
perceived benefit	P
perceived benefit	C
perceived benefit	C
perceived benefit	C
perceived benefit	C
perceived benefit	C
perceived benefit	C
perceived benefit	C
perceived benefit	C
perceived benefit	C
perceived benefit	C
perceived benefit	P
perceived benefit	P
perceived benefit	P

perceived benefit	P
Barrier	P
Facilitator	T
Facilitator	T
Facilitator	T
Facilitator	C
Facilitator	C
Barrier	C
barrier	P
Facilitator	HS
Facilitator	C
Facilitator	T
Barrier	C
Barrier	P
Barrier	P
Barrier	P
Barrier	P
Barrier	T
Barrier	T
Barrier	T
Facilitator	HS
Facilitator	HS
Facilitator	HS
barrier	T
Facilitator	HS
Barrier	C
Facilitator	P
Facilitator	T
Facilitator	T
Facilitator	T
Barrier	T
Facilitator	HS
Facilitator	HS
perceived benefit	C
perceived benefit	C
perceived benefit	C
facilitator	P

facilitator	P
facilitator	T
facilitator	P
facilitator	P
facilitator	P
barrier	T
barrier	T
facilitator	P
barrier	P
barrier	P
barrier	P
barrier	C
facilitator	T
facilitator	T
barrier	T
barrier	T
barrier	T
barrier	T
barrier	T
facilitator	P
perceived benefit	C
perceived benefit	C
perceived benefit	C
perceived drawback	C
barrier	P
barrier	P
facilitator	P
facilitator	T
facilitator	C
barrier	C
facilitator	P
barrier	P
barrier	T
barrier	P
facilitator	HS
facilitator	C
barrier	P

facilitator	C
Facilitator	HS
barrier	HS
facilitator	HS
facilitator	C
barrier	C
barrier	P
facilitator	T
facilitator	T
barrier	HS
facilitator	HS
barrier	HS
barrier	HS
barrier	C
perceived benefit	P
perceived drawback	C
perceived benefit	C
Barrier	C
perceived benefit	C
perceived benefit	C
Facilitator	T
Barrier	T
Facilitator	T
Facilitator	T
Barrier	C
Barrier	C
barrier	T
Barrier	T
Facilitator	T
Barrier	T
Barrier	T
Facilitator	T
Facilitator	T
Barrier	T
Facilitator	T
Barrier	C
Facilitator	C

Barrier	T
Barrier	HS
Barrier	T
Barrier	HS
barrier	HS
Barrier	T
Barrier	T
Facilitator	T
Facilitator	T
Facilitator	T
Facilitator	HS
Facilitator	HS
Facilitator	C
Facilitator	C
Facilitator	C
facilitator	C
barrier	C
Barrier	C
barrier	C
barrier	C
Barrier	C
Barrier	C
Barrier	C
Facilitator	C
barrier	C
barrier	C
Barrier	C
Barrier	C
Barrier	C
Barrier	C
Barrier	T
Barrier	T
barrier	T
barrier	T
barrier	T
Facilitator	HS
Barrier	HS

Barrier	C	
Barrier	C	
barrier	C	
perceived drawback	C	
Perceived drawback	P	
perceived drawback	C	
facilitator	P	
perceived benefit	C	
perceived benefit	C	
perceived benefit	C	
perceived benefit	C	
Perceived drawback	C	
perceived benefit	C	
barrier	P	
barrier	P	
barrier	P	
barrier	P	
Facilitator	HS	
barrier	T	
barrier	T	
facilitator	C	
facilitator	P	
barrier	C	
facilitator	P	
barrier	C	
facilitator	T	
perceived benefit	P	
perceived benefit	P	
perceived benefit	P	
perceived benefit	P	
perceived benefit	P	
perceived drawback	C	
Facilitator	T	
perceived benefit	C	
perceived benefit	C	
perceived benefit	P	
perceived benefit	P	

perceived benefit	P
Facilitator	T
Facilitator	C
Facilitator	C
Facilitator	T
Facilitator	C
Facilitator	T
Facilitator	T
Facilitator	C
facilitator	HS
perceived benefit	C
perceived benefit	C
perceived benefit	P
facilitator	T
facilitator	T
facilitator	C
facilitator	C
facilitator	C
facilitator	HS
facilitator	HS
perceived benefit	C
perceived benefit	C
perceived benefit	C
perceived benefit	C
perceived benefit	C
Facilitator	P
Barrier	T
Facilitator	T
facilitator	T
Facilitator	T
Barrier	C
Barrier	C
Barrier	C
Facilitator	P
Barrier	T
Barrier	C
Facilitator	HS

Facilitator	T
barrier	HS
Facilitator	HS
Barrier	C
barrier	HS
Facilitator	HS
barrier	C
barrier	C
Barrier	C
Barrier	C
Barrier	C
Barrier	C
Barrier	C
Facilitator	P
Barrier	C
Facilitator	C
Facilitator	C
barrier	P
facilitator	HS
Facilitator	HS
Facilitator	HS
Facilitator	HS
barrier	HS
Facilitator	HS
Facilitator	HS
Facilitator	HS
Facilitator	HS
Barrier	C
Perceived drawback	C
perceived benefit	C
perceived benefit	C
perceived benefit	C
perceived benefit	C
perceived benefit	C
perceived benefit	C
perceived drawback	C
perceived drawback	C

perceived drawback	C
barrier	T
barrier	T
facilitator	C
barrier	P
facilitator	T
barrier	C
barrier	C
facilitator	P
facilitator	P
facilitator	P
facilitator	HS
barrier	T
barrier	HS
barrier	T
barrier	HS
barrier	T
Facilitator	C
barrier	HS
barrier	HS
barrier	HS
barrier	C
barrier	C
barrier	P
Barrier	C
facilitator	C
facilitator	C
facilitator	C
barrier	C
facilitator	T
Barrier	HS
perceived benefit	C
perceived benefit	C
perceived drawback	C
perceived drawback	C
perceived drawback	C
perceived drawback	C

Facilitator	P
Perceived benefit	C
perceived benefit	C
perceived benefit	C
perceived benefit	C
perceived benefit	C
perceived benefit	C
Perceived benefit	C
perceived benefit	C
Perceived benefit	C
Perceived benefit	C
perceived benefit	P
perceived benefit	P
perceived benefit	P
Perceived benefit	P
Perceived benefit	P
Perceived benefit	P
Perceived benefit	P
Perceived benefit	P
Perceived benefit	P
Perceived benefit	P
Perceived benefit	P
Perceived benefit	P
perceived benefit	P
Perceived benefit	P
Perceived benefit	P
Perceived benefit	P
Perceived benefit	C
Perceived benefit	C
barrier	P
barrier	P
barrier	P
barrier	P
Facilitator	T
Facilitator	T
Facilitator	T
facilitator	T

barrier	C
Facilitator	T
Facilitator	T
Facilitator	P
barrier	C
barrier	P
Facilitator	C
Facilitator	T
Facilitator	T
Facilitator	T
barrier	P
barrier	P
barrier	P
barrier	P
barrier	P
barrier	P
Facilitator	T
Facilitator	T
Facilitator	T
barrier	T
barrier	HS
barrier	C
Facilitator	T
Facilitator	T
Facilitator	T
barrier	T
barrier	T
barrier	T
Barrier	P
Facilitator	T
Facilitator	T
Facilitator	T
Facilitator	T
Facilitator	C
Facilitator	P
Facilitator	C
Barrier	C

Facilitator	C
Facilitator	C
barrier	C
barrier	P
barrier	C
barrier	C
barrier	P
Facilitator	T
Facilitator	T
Facilitator	T
Facilitator	T
Facilitator	T
Facilitator	T
Facilitator	T
Facilitator	HS
Facilitator	HS
barrier	C
Perceived drawback	C
Perceived drawback	C
Perceived drawback	C
facilitator	C
perceived benefit	C
perceived benefit	C
perceived benefit	C
perceived benefit	P
perceived benefit	P
perceived benefit	P
perceived benefit	P
perceived benefit	C
perceived benefit	C
perceived benefit	C
perceived benefit	C
facilitator	C
facilitator	T
facilitator	P
barrier	T
barrier	P

facilitator	C
barrier	C
barrier	C
facilitator	C
facilitator	C
barrier	C
perceived benefit	C
facilitator	C
perceived benefit	C
barrier	P
facilitator	T
barrier	P
barrier	P
barrier	P
barrier	P
barrier	P
barrier	P
facilitator	P
facilitator	P
facilitator	T
barrier	T
perceived benefit	P
perceived benefit	P
perceived benefit	P
Facilitator	C
perceived benefit	C
perceived drawback	C
perceived drawback	P
Barrier	C
Barrier	C
Barrier	C
barrier	C
Facilitator	P
Barrier	P
Barrier	P
Facilitator	T
barrier	T

Facilitator	T
Facilitator	T
Barrier	P
Facilitator	T
Facilitator	T
Barrier	P
barrier	P
Facilitator	T
Barrier	P
barrier	T
barrier	T
Facilitator	HS
Barrier	P
Barrier	C
Facilitator	C
Facilitator	C
Facilitator	HS
Facilitator	HS
Facilitator	C
Facilitator	T
Barrier	T
barrier	T
Facilitator	HS
Facilitator	HS
barrier	C
Barrier	C
perceived benefit	C
perceived drawback	C
perceived benefit	C
facilitator	C
perceived benefit	C
perceived benefit	C
perceived benefit	C
perceived drawback	P
perceived drawback	C
facilitator	T
barrier	T

barrier	T
barrier	C
barrier	C
barrier	C
Facilitator	C
facilitator	C
facilitator	C
barrier	C
Facilitator	T
barrier	P
barrier	C
facilitator	C
barrier	HS
barrier	HS
barrier	HS
barrier	C
barrier	T
perceived benefit	C
perceived benefit	C
perceived benefit	C
perceived benefit	C
perceived benefit	P
perceived drawback	P
Perceived benefit	P
perceived benefit	P
perceived benefit	P
perceived benefit	P
perceived benefit	P
perceived benefit	P
perceived drawback	P
perceived benefit	P
Facilitator	P
barrier	T
Barrier	T
Facilitator	C
Barrier	C
Barrier	C

barrier	T
Facilitator	P
Facilitator	P
Facilitator	P
barrier	P
barrier	T
barrier	T
barrier	P
Facilitator	T
barrier	P
Barrier	P
Barrier	P
barrier	HS
barrier	C
barrier	P
perceived drawback	C
Facilitator	P
barrier	P
Facilitator	P
barrier	P
barrier	P
barrier	T
barrier	T
barrier	T
barrier	T
barrier	T
barrier	T
Barrier	HS
facilitator	HS
barrier	HS
barrier	HS
barrier	HS
facilitator	HS
facilitator	HS
facilitator	HS
facilitator	HS
facilitator	HS

facilitator	HS
Barrier	P
perceived drawback	C
perceived drawback	C
perceived benefit	C
perceived drawback	C
perceived drawback	C
perceived benefit	C
perceived drawback	C
perceived benefit	C
perceived drawback	C
perceived benefit	C
perceived benefit	C
facilitator	C
perceived drawback	C
perceived drawback	C
perceived benefit	P
perceived benefit	P
Perceived benefit	P
barrier	P
facilitator	P
Facilitator	T
facilitator	T
Facilitator	T
Facilitator	T
Facilitator	T
barrier	P
barrier	P
barrier	T
facilitator	HS
Facilitator	P
barrier	P
barrier	HS
barrier	T
facilitator	HS
facilitator	HS
facilitator	HS

barrier	HS
Facilitator	HS
facilitator	HS
barrier	P
barrier	C
barrier	C
Facilitator	C
barrier	C
facilitator	C
barrier	HS
barrier	HS
Facilitator	HS
barrier	C
barrier	P
Facilitator	P
barrier	P
facilitator	C
barrier	C
barrier	P
barrier	P
Facilitator	P
Facilitator	C
facilitator	T
facilitator	T
Barrier	HS
barrier	HS
facilitator	HS
barrier	HS
facilitator	HS
Facilitator	HS
Facilitator	HS
Facilitator	HS
Facilitator	HS
Facilitator	HS
Facilitator	HS
Facilitator	HS
facilitator	HS

perceived drawback	C
perceived drawback	C
perceived benefit	C
perceived benefit	C
Perceived drawback	C
perceived drawback	C
perceived benefit	P
perceived benefit	P
perceived drawback	P
perceived benefit	C
Facilitator	T
facilitator	T
barrier	P
barrier	P
barrier	T
barrier	T
barrier	T
barrier	T
Facilitator	C
facilitator	C
barrier	C
barrier	C
Facilitator	T
barrier	T
barrier	C
barrier	C
barrier	C
perceived drawback	C
perceived benefit	P
perceived benefit	C
perceived benefit	P
perceived benefit	P
perceived benefit	P
perceived benefit	P
perceived benefit	P
perceived benefit	P
perceived benefit	C

perceived benefit	C
perceived benefit	C
facilitator	P
facilitator	T
facilitator	T
facilitator	T
barrier	T
facilitator	T
facilitator	T
facilitator	P
barrier	P
Barrier	P
facilitator	T
facilitator	T
facilitator	T
Facilitator	T
facilitator	P
barrier	P
barrier	P
facilitator	T
facilitator	T
facilitator	C
barrier	T
facilitator	T
barrier	T
barrier	P
barrier	P
Barrier	P
facilitator	C
facilitator	C
facilitator	C
Facilitator	C
barrier	P
barrier	P
facilitator	C
facilitator	HS
facilitator	T

perceived benefit	P
perceived benefit	P
perceived drawback	P
Barrier	C
Barrier	P
facilitator	T
facilitator	T
facilitator	T
facilitator	T
barrier	T
barrier	T
facilitator	T
barrier	C
Barrier	C
facilitator	T
facilitator	T
barrier	T
facilitator	T
barrier	T
barrier	P
Barrier	P
barrier	T
barrier	C
barrier	P
Barrier	C
Barrier	P
facilitator	P
facilitator	HS
facilitator	HS
Barrier	P
Barrier	C
barrier	HS
barrier	P
facilitator	C
facilitator	P
barrier	P
barrier	C
facilitator	T

facilitator	T
barrier	T
facilitator	HS
barrier	HS
barrier	HS
barrier	HS
barrier	HS
barrier	C
barrier	C
perceived benefit	C
perceived benefit	C
perceived benefit	C
perceived drawback	P
perceived drawback	P
barrier	T
barrier	HS
barrier	C
barrier	C
barrier	C
barrier	T
barrier	T
barrier	T
barrier	HS
barrier	HS
barrier	C
barrier	C
perceived drawback	C
perceived drawback	C
facilitator	P
perceived benefit	C
perceived benefit	C
Facilitator	C
barrier	P
facilitator	T
facilitator	T
facilitator	P
facilitator	P

barrier	P
facilitator	T
barrier	P
facilitator	T
facilitator	T
facilitator	C
facilitator	C
facilitator	C
facilitator	C
facilitator	C
facilitator	C
facilitator	C
facilitator	T
facilitator	T
facilitator	T
facilitator	T
facilitator	HS
perceived drawback	C
Facilitator	T
Facilitator	C
Facilitator	T
facilitator	T
perceived benefit	C
facilitator	P
perceived benefit	C
barrier	T
perceived benefit	C
perceived drawback	C
perceived benefit	P
perceived drawback	P
barrier	T
barrier	T
facilitator	T
facilitator	T
facilitator	T
barrier	T
facilitator	T

facilitator	P
barrier	T
barrier	T
barrier	T
barrier	T
barrier	C
barrier	P
barrier	P
barrier	P
barrier	P
barrier	P
facilitator	P
facilitator	C
facilitator	C
barrier	C
barrier	C
barrier	C
barrier	C
Facilitator	C
barrier	C
barrier	T
facilitator	HS
barrier	C
perceived drawback	C
perceived drawback	C
facilitator	HS
perceived benefit	C
perceived benefit	P
barrier	C
barrier	C
barrier	C
barrier	C
barrier	P
barrier	P
barrier	P
barrier	P
barrier	P

barrier	T
facilitator	T
barrier	C
barrier	HS
barrier	T
barrier	T
facilitator	T
barrier	HS
barrier	C
facilitator	C
barrier	C
facilitator	C
facilitator	HS
barrier	HS
barrier	HS
barrier	HS
barrier	HS
barrier	HS
barrier	HS
facilitator	HS
barrier	C
barrier	P
facilitator	C
facilitator	P
facilitator	HS
facilitator	HS
Facilitator	T
perceived benefit	C
perceived benefit	C
perceived drawback	C
perceived drawback	C
perceived benefit	C
perceived benefit	C
perceived benefit	C
perceived benefit	C
perceived drawback	C
perceived drawback	C

perceived drawback	C
perceived drawback	C
Facilitator	C
Facilitator	T
barrier	T
barrier	T
barrier	T
barrier	T
barrier	T
barrier	T
barrier	C
barrier	C
perceived drawback	C
Facilitator	P
barrier	P
facilitator	P
Facilitator	T
Facilitator	T
facilitator	HS
facilitator	HS
facilitator	HS
facilitator	HS
facilitator	HS
barrier	HS
barrier	C
perceived drawback	C
perceived benefit	C
Facilitator	P
perceived benefit	C
perceived drawback	C
perceived drawback	C
perceived benefit	C
perceived benefit	P
perceived benefit	P
barrier	T
facilitator	T
facilitator	T

facilitator	T
facilitator	T
Facilitator	T
facilitator	T
facilitator	T
facilitator	T
facilitator	P
facilitator	P
facilitator	P
barrier	T
barrier	P
barrier	T
facilitator	T
facilitator	T
facilitator	T
barrier	T
barrier	T
facilitator	P
facilitator	P
facilitator	C
facilitator	P
facilitator	P
Barrier	C
facilitator	C
facilitator	T
facilitator	T
facilitator	T
facilitator	T
facilitator	T
facilitator	HS
perceived drawback	C
Barrier	P
Barrier	T
Barrier	T
Barrier	C
Barrier	C
facilitator	HS

barrier	T
Barrier	C
facilitator	C
perceived drawback	C
Barrier	C
facilitator	HS
Facilitator	C
perceived benefit	C
perceived benefit	C
perceived benefit	C
perceived drawback	C
Facilitator	HS
Facilitator	T
Facilitator	HS
Facilitator	HS
Barrier	P
Facilitator	C
Facilitator	HS
Barrier	C
Facilitator	C
Facilitator	C
Facilitator	HS
Barrier	C
perceived benefit	C
perceived benefit	C
perceived benefit	P
perceived benefit	P
perceived drawback	C
perceived drawback	C
perceived drawback	C
perceived drawback	C
facilitator	T
facilitator	T
facilitator	T
facilitator	T
barrier	T
facilitator	C

facilitator	C
facilitator	C
facilitator	C
facilitator	C
facilitator	C
barrier	C
facilitator	T
facilitator	T
facilitator	T
facilitator	T
facilitator	T
facilitator	T
facilitator	T
facilitator	T
barrier	T

Country income classification
lower-middle
lower-middle
lower-middle
lower-middle
lower-middle
lower-middle
lower-middle
lower-middle
lower-middle
lower-middle
lower-middle
lower-middle
lower-middle
lower-middle
lower-middle
lower-middle
lower-middle
lower-middle
high
high
high
high
high
high
high
high
high
high
high
high
high
high
high
high
high
high

high
high
high
high
high
high
high
high
high
high
high
high
lower-middle
lower-middle
lower-middle
lower-middle
lower-middle
lower-middle
lower-middle
lower-middle
lower-middle
lower-middle
lower-middle
lower-middle
lower-middle
lower-middle
lower-middle
lower-middle
lower-middle
lower-middle
lower-middle
lower-middle
lower-middle
lower-middle
lower-middle
lower-middle
lower-middle

lower-middle
lower-middle
lower-middle
lower-middle
high
high
high
high
high
high
high
high
high
high
high
high
high
high
high
high
high
high
high
high
high
high
high
high
high
high
high
high
high
high
high
high
high

high
high
high
high
high
high
high
high
high
high
high
high
high
high
high
high
high
high
high
high
high
high
high
high
high
high
high
high
high
high
high
high
high
high
high
high
high

high
high
high
high
high
high
high
high
high
high
high
high
high
high
high
high
high
high
high
high
high
high
high
high
high
high
high
high
high
high
high
high
high
high
high
high
high

high
high
high
high
high
high
high
high
high
high
high
high
high
lower-middle
lower-middle
lower-middle
lower-middle
lower-middle
lower-middle
lower-middle
high
high
high
high
high
high
high
high
high
high
high
high
high
high
high
high
high

high
high
high
high
high
high
high
high
high
high
high
high
high
high
high
high
high
high
high
high
high
high
high
high
high
high
high
high
high
high
high
high
high
high
high
high
high

high
high
high
high
high
high
high
high
high
high
high
high
high
high
high
high
high
high
high
high
high
high
high
high
high
high
high
high
high
high
high
high
high
high
high
high
high

high
high
high
high
high
high
lower-middle
lower-middle
lower-middle
lower-middle
lower-middle
lower-middle
lower-middle
lower-middle
lower-middle
lower-middle
lower-middle
lower-middle
lower-middle
lower-middle
lower-middle
lower-middle
lower-middle
lower-middle
lower-middle
high
high
high
high
high
high
high
high
high
high
high
high

high
high
high
high
high
high
high
high
high
high
high
high
high
high
high
high
high
high
high
high
high
high
high
high
high
high
high
high
high
high
high
high
high
high
high
high
high

high
high
high
high
high
high
high
high
high
high
high
high
high
high
high
high
high
high
high
high
high
high
high
high
high
high
high
high
high
high
high
high
high
high
high
high
high

high
high
high
high
high
high
high
high
high
high
high
high
high
high
high
high
high
high
high
high
high
high
high
high
high
high
high
high
high
high
high
high
high
high
high
high
high

high
high
high
high
high
high
high
high
high
high
high
high
high
high
high
high
high
high
high
high
high
high
high
high
high
high
high
high
high
high
high
high
high
high
upper-middle
upper-middle
upper-middle

upper-middle
upper-middle
upper-middle
upper-middle
upper-middle
upper-middle
upper-middle
upper-middle
upper-middle
upper-middle
upper-middle
upper-middle
upper-middle
upper-middle
upper-middle
upper-middle
upper-middle
upper-middle
upper-middle
upper-middle
upper-middle
upper-middle
upper-middle
upper-middle
high
high
high
high
high
high
high
high
high
high
high
high
high

high
high
high
high
high
high
high
high
high
high
high
high
high
high
high
high
high
high
high
high
high
high
high
high
high
high
high
high
high
high
high
high
high
high
high
high
high

high
high
high
high
high
high
high
high
high
high
high
high
high
high
high
high
high
high
high
high
high
high
high
high
high
high
high
high
high
high
high
high
high
high
high
high
high

high
high
high
high
high
high
high
high
high
high
high
high
high
high
high
high
high
high
high
high
high
high
high
high
high
high
high
high
high
high
high
high
high
high
high
high
high

high
high
high
high
high
high
high
high
high
high
high
high
high
high
high
high
high
high
high
high
high
high
high
high
high
high
high
high
high
high
high
high
high
high
high
high
high

high
high
high
high
high
high
high
high
high
high
high
high
high
high
high
high
high
high
high
high
high
high
high
high
high
high
high
high
high
high
high
high
high
high
high
high
high

high
high
high
high
high
high
high
high
high
high
high
high
high
high
high
high
high
high
high
high
high
high
high
high
high
high
high
high
high
high
high
high
high
high
high
high
high

high
high
high
high
high
high
high
high
high
high
high
high
high
high
high
high
high
high
high
high
high
high
high
high
high
high
high
high
high
high
high
high
high
high
high
high
high

high
high
high
high
high
high
high
high
high
high
high
high
high
high
high
high
high
high
high
high
high
high
high
high
high
high
high
high
high
high
high
high
high
high
high
high
high

high
high
high
high
high
high
high
high
high
high
high
high
high
high
high
high
high
high
high
high
high
high
high
high
high
high
high
high
high
high
high
high
high
high
high
high
high

high
high
high
high
high
high
high
high
high
high
high
high
high
high
high
high
high
high
high
high
high
high
high
high
high
high
high
high
high
high
high
high
high
high
high
high
high

high
high
high
high
high
high
high
high
high
high
high
high
high
high
high
high
high
high
high
high
high
high
high
high
high
high
high
high
high
high
high
high
high
high
high
high
high

high
high
high
high
high
high
high
high
high
high
high
high
high
high
high
high
high
high
high
high
high
high
high
high
high
high
high
high
high
high
high
high
high
high
high
high
high

high
high
high
high
high
high
high
high
high
high
high
high
high
high
high
high
high
high
high
high
high
high
high
high
high
high
high
high
high
high
high
high
high
high
high
high
high

high
high
high
high
high
high
high
high
high
high
high
high
high
high
high
high
high
high
high
high
high
high
high
high
high
high
high
high
high
high
high
high
high
high
high
high
high

high
high
high
high
high
high
high
high
high
high
high
high
high
high
high
high
high
high
high
high
high
high
high
high
high
high
high
high
high
high
high
high
high
high
high
high
high

high
high
high
high
high
high
high
high
high
high
high
high
high
high
high
high
high
high
high
high
high
high
high
high
high
high
high
high
high
high
high
high
high
high
high
high
high

high
high
high
high
high
high
high
high
high
high
high
high
high
high
high
high
high
high
high
high
high
high
high
high
high
high
high
high
high
high
high
high
high
high
high
high
high

high
high
high
high
high
high
high
high
high
high
high
high
high
high
high
high
high
high
high
high
high
high
high
high
high
high
high
high
high
high
high
high
high
high
high
high
high

high
high
high
high
high
high
high
high
high
high
high
high
high
high
high
high
high
high
high
high
high
high
high
high
high
high
high
high
high
high
high
high
high
high
high
high
high

high
high
high
high
high
high
high
high
high
high
high
high
high
high
high
high
high
high
high
high
high
high
high
high
high
high
high
high
high
high
high
high
high
high
high
high
high

high
high
high
high
high
high
high
high
high
high
high
high
high
high
high
high
high
high
high
high
high
high
high
high
high
high
high
high
high
high
high
high
high
high
high
high
high

high
high
high
high
high
high
high
high
high
high
high
high
high
high
high
high
high
high
high
high
high
high
high
high
high
high
high
high
high
high
high
high
high
high
high
high
high

high
high
high
high
high
high
high
high
high
high
high
high
high
high
high
high
high
high
high
high
high
high
high
high
high
high
high
high
high
high
high
high
high
high
high
high
high

high
high
high
high
high
high
high
high
high
high
high
high
high
high
high
high
high
high
high
high
high
high
high
high
high
high
high
high
high
high
high
high
high
high
high
high
high

high
high
high
high
high
high
high
high
high
high
high
high
high
high
high
high
high
high
high
high
high
high
high
high
high
high
high
high
high
high
high
high
high
high
high
high
high

high
high
high
high
high
high
high
high
high
high
high
high
high
high
high
high
high
high
high
high
high
high
high
high
high
high
high
high
high
high
high
high
high
high
high
high

high
high
high
high
high
high
high
high
high
high
high
high
high
high
high
high
high
high
high
high
high
high
high
high
high
high
high
high
high
high
high
high
high
high
high
high
high

high
high
high
high
high
high
high
high
high
high
high
high
high
high
high
high
lower-middle
lower-middle
lower-middle
lower-middle
lower-middle
lower-middle
lower-middle
lower-middle
lower-middle
lower-middle
lower-middle
lower-middle
high
high
high
high
high
high
high
high
high

high
high
high
high
high
high
high
high
high
high
high
high
high
high
high
high
high
high
high
high
high
high
high
high
high
high
high
high
high
high
high
high
high
high
high
high
high
high

high
high
high
high
high
high
high
high
high
high
high
high
high
high
high
high
high
high
high
high
high
high
high
high
high
high
high
high
high
high
high
high
high
high
high
high
high

high
high
high
high
high
high
high
high
high
high
high
high
high
high
high
high
high
high
high
high
high
high
high
high
high
high
high
high
high
high
high
high
high
high
high
high
high

high
high
high
high
high
high
high
high
high
high
high
high
high
high
high
high
high
high
high
high
high
high
high
high
high
high
high
high
high
high
high
high
high
high
high
high
high

high
high
high
high
high
high
high
high
high
high
high
high
high
high
high
high
high
high
high
high
high
high
high
high
high
high
high
high
high
high
high
high
high
high
high
high
high

high
high
high
high
high
high
high
high
high
high
high
high
high
high
high
high
high
high
high
high
high
high
high
high
high
high
high
high
high
high
high
high
high
high
high
high
high

high
high
high
high
upper-middle
upper-middle
upper-middle
upper-middle
upper-middle
upper-middle
upper-middle
upper-middle
upper-middle
upper-middle
upper-middle
upper-middle
upper-middle
upper-middle
high
high
high
high
high
high
high
high
high
high
high
high
high
high
high
high
high
high
high

high
high
high
high
high
high
high
high
high
high
high
high
high
high
high
high
high
high
high
high
high
high
high
high
high
high
high
high
high
high
high
high
high
high
high
high
high

high
high
high
high
high
high
high
high
high
high
high
high
high
high
high
high
high
high
high
high
high
high
high
high
high
high
high
high
high
high
high
high
high
high
high
high
high

high
high
high
high
high
high
high
high
high
high
high
high
high
high
high
high
high
high
high
high
high
high
high
high
high
high
high
high
high
upper-middle
upper-middle
upper-middle
high
high
high
high
high

high
high
high
high
high
high
high
high
high
high
high
high
high
high
high
high
lower-middle
lower-middle
lower-middle
lower-middle
lower-middle
lower-middle
lower-middle
lower-middle
lower-middle
lower-middle
lower-middle
lower-middle
lower-middle
lower-middle
lower-middle
lower-middle
lower-middle
lower-middle
lower-middle
lower-middle
lower-middle

lower-middle
lower-middle
lower-middle
lower-middle
high
high
high
high
high
high
high
high
high
high
high
high
high
high
high
high
high
high
high
high
high
high
high
high
high
high
high
high
high
high
high
high
high

high
high
high
high
high
high
high
high
high
high
high
high
high
high
high
high
high
high
high
high
high
high
high
high
high
high
high
high
high
high
high
high
high
high
high
high
high

high
high
high
high
high
high
high
high
high
high
high
high
high
high
high
high
high
high
high
high
high
high
high
high
high
high
high
high
high
high
high
high
high
high
high
high
high

high
high
high
high
high
high
high
high
high
high
high
high
high
high
high
high
high
high
high
high
high
high
high
high
high
high
high
high
high
high
high
high
high
high
high
high
high

high
high
high
high
high
high
high
high
high
high
high
high
high
high
high
high
high
high
high
high
high
high
high
high
high
high
high
high
high
high
high
high
high
high
high
high
high

high
high
high
high
high
high
high
high
high
high
high
high
high
high
high
high
high
high
high
high
high
high
high
high
high
high
high
high
high
high
high
high
high
high
high
high
high

high
high
high
high
high
high
high
high
high
high
high
high
high
high
high
high
high
high
high
high
high
high
high
high
high
high
high
high
high
high
high
high
high
high
high
high
high

high
high
high
high
high
high
high
high
high
high
high
high
high
high
high
high
high
high
high
high
high
high
high
high
high
high
high
high
high
high
high
high
high
high
high
high
high

high
high
high
high
high
high
high
high
high
high
high
high
lower-middle
lower-middle
lower-middle
lower-middle
lower-middle
lower-middle
lower-middle
high
high
high
high
high
high
high
high
high
high
high
high
high
high
high
high
high
high

high
high
high
high
high
high
high
high
high
high
high
high
high
high
high
high
high
high
high
high
high
high
high
high
high
high
high
high
high
high
high
high
high
high
high
high
high

high
high
high
high
high
high
high
high
high
high
high
high
high
high
high
high
high
high
high
high
high
high
high
high
high
high
high
high
high
high
upper-middle
upper-middle
upper-middle
upper-middle
upper-middle
upper-middle
upper-middle

upper-middle
upper-middle
upper-middle
upper-middle
upper-middle
upper-middle
upper-middle
upper-middle
upper-middle
high
high
high
high
high
high
high
high
high
high
high
high
high
high
high
high
high
high
high
high
high
high
high
high
high
high
high
high

high
high
high
high
high
high
high
high
high
high
high
high
high
high
high
high
high
high
high
high
high
high
high
high
high
high
high
high
high
high
high
high
high
high
high
high
high

high
high
high
high
high
high
high
high
high
high
high
high
high
high
high
high
high
high
high
high
high
high
high
high
high
high
high
high
high
high
high
high
high
high
high
high
high

high
high
high
high
high
high
high
high
high
high
high
high
high
high
high
high
high
high
high
high
high
high
high
high
high
high
high
high
high
high
high
high
high
high
high
high
high

high
high
high
high
high
high
high
high
high
high
high
high
high
high
high
high
high
high
high
high
high
high
high
lower-middle
lower-middle
lower-middle
lower-middle
lower-middle
lower-middle
lower-middle
lower-middle
lower-middle
lower-middle
lower-middle
lower-middle
lower-middle
lower-middle

lower-middle
lower-middle
lower-middle
lower-middle
lower-middle
lower-middle
lower-middle
lower-middle
lower-middle
lower-middle
high
high
high
high
high
high
high
high
high
high
high
high
high
high
high
high
high
high
high
high
high
high
high
high
high
high
high

high
upper-middle
upper-middle
upper-middle
upper-middle
upper-middle
upper-middle
upper-middle
upper-middle
upper-middle
upper-middle
upper-middle
upper-middle
upper-middle
upper-middle
upper-middle
upper-middle
upper-middle
upper-middle
high
high
high
high
high
high
high
high
high
high
high
high
high
high
high
high
high
high

high
high
high
high
high
high
high
high
high
high
high
high
high
high
high
high
high
high
high
high
high
high
high
high
high
high
high
high
high
high
high
high
high
high
high
high
high

high
high
high
high
high
high
high
high
high
high
high
high
high
high
high
high
high
high
high
high
high
high
high
high
high
high
high
high
high
high
high
high
high
high
high
high
high

high
high
high
high
high
high
high
high
high
high
high
high
high
high
high
high
high
high
high
high
high
high
high
high
high
high
high
high
high
high
high
high
high
high
high
high
high

high
high
high
high
high
high
high
high
high
high
high
high
high
high
high
high
high
high
high
high
lower-middle
lower-middle
lower-middle
lower-middle
lower-middle
lower-middle
lower-middle
lower-middle
lower-middle
lower-middle
lower-middle
lower-middle
lower-middle
lower-middle
lower-middle
lower-middle
lower-middle

lower-middle
lower-middle
lower-middle
lower-middle
lower-middle
lower-middle
lower-middle
lower-middle
lower-middle
lower-middle
lower-middle
lower-middle
lower-middle
lower-middle
lower-middle
lower-middle
lower-middle
lower-middle
lower-middle
lower-middle
lower-middle
lower-middle
lower-middle
lower-middle
lower-middle
lower-middle
lower-middle
lower-middle
lower-middle
high
high
high
high
high
high
high
high

high
high
high
high
high
high
high
high
high
high
high
high
high
high
high
high
high
high
high
high
high
high
high
high
high
high
high
high
high
high
high
high
high
high
high
high
high

high
high
high
high
high
high
high
high
high
high
high
high
high
high
high
high
high
high
high
high
high
high
high
high
high
high
high
high
high
high
high
high
high
high
high
high

high
high
high
high
high
high
high
high
high
high
high
high
high
high
high
high
high
high
high
high
high
high
high
high
high
high
high
high
high
high
high
high
high
high
high
high
high

high
high
high
high
high
high
high
high
high
high
high
high
high
high
high
high
high
high
high
high
high
high
high
high
high
high
high
high
high
high
high
high
high
high
high
high
high

high
high
high
high
high
high
high
lower-middle
lower-middle
lower-middle
lower-middle
lower-middle
lower-middle
lower-middle
lower-middle
lower-middle
lower-middle
lower-middle
lower-middle
lower-middle
lower-middle
lower-middle
lower-middle
lower-middle
lower-middle
lower-middle
lower-middle
lower-middle
lower-middle
lower-middle
lower-middle
lower-middle
lower-middle
lower-middle
lower-middle
lower-middle
lower-middle

lower-middle
lower-middle
lower-middle
lower-middle
lower-middle
lower-middle
lower-middle
lower-middle
lower-middle
lower-middle
lower-middle
lower-middle
lower-middle
lower-middle
lower-middle
lower-middle
lower-middle
lower-middle
lower-middle
lower-middle
lower-middle
lower-middle
lower-middle
lower-middle
lower-middle
lower-middle
lower-middle
lower-middle
lower-middle
high
high
high
high
high
high
high
high

high
high
high
high
high
high
high
high
high
high
high
high
high
high
high
high
high
high
high
high
high
high
high
high
high
high
high
high
high
high
high
high
high
high
high
high
high

high
high
high
high
high
high
high
high
high
high
high
high
high
high
high
high
high
high
high
high
high
high
high
high
high
high
high
high
high
high
high
high
high
high
high
high
high

high
high
high
high
high
high
high
high
high
high
high
high
high
high
high
high
high
high
high
high
high
high
high
high
high
high
high
high
high
high
high
high
high
high
high
high
high

high
high
high
high
high
high
high
high
high
high
high
high
high
high
high
high
high
high
high
high
high
high
high
high
high
high
high
high
high
high
high
high
high
high
high
high
high

high
high
high
high
high
high
high
high
high
high
high
high
high
high
high
high
high
high
high
high
high
high
high
high
high
high
high
high
high
high
high
high
high
high
high
high
high

high
high
high
high
high
high
high
high
high
high
high
high
high
high
high
high
high
high
high
high
high
high
high
high
high
high
high
high
high
high
high
high
high
high
high
high
high

high
high
high
high
high
high
high
high
high
high
high
high
high
high
high
high
high
high
high
high
high
high
high
high
high
high
high
high
high
high
high
high
high
high
high
high
high
high

high
high
high
high
high
high
high
high
high
high
high
high
high
high
high
high
high
high
high
high
high
high
high
high
high
high
high
high
high
high
high
high
high
high
high
high
high

high
high
high
high
high
high
high
high
high
high
high
high
high
high
high
high
high
high
high
high
high
high
high
high
high
high
high
high
high
high
high
high
high
high
high
high
high

high
high
high
high
high
high
high
high
high
high
high
high
high
high
high
high
high
high
high
high
high
high
high
high
high
high
high
high
high
high
high
high
high
high
high
high
high

high
high
high
high
high
high
high
high
high
high
high
high
high
high
high
high
high
high
high
high
high
high
high
high
high
high
high
high
high
high
high
high
high
high
high
high
high

high
high
high
high
high
high
high
high
high
high
high
high
high
high
high
high
high
high
high
high
high
high
high
high
high
high
high
high
high
high
high
high
high
high
high
high
high

high
high
high
high
high
high
high
high
high
high
high
high
high
high
high
high
high
high
high
high
high
high
high
high
high
high
high
high
high
high
high
high
high
high
high
high
high

high
high
high
high
high
high
upper-middle
upper-middle
upper-middle
upper-middle
upper-middle
upper-middle
upper-middle
upper-middle
upper-middle
upper-middle
upper-middle
upper-middle
upper-middle
upper-middle
upper-middle
upper-middle
upper-middle
high
high
high
high
high
high
high
high
high
high
high
high
high
high

high
high
high
high
high
high
high
high
high
high
high
high
high
high
high

Supplemental file 3.

Table S3.1: Characteristics of 125 included primary studies.
Table S3.2: Distribution of the number of studies and healthcare providers across countries.

The reference list of all 125 included primary studies is given at the end of this supplemental file.

Abbreviations
ACS, acute coronary syndrome
AF, atrial fibrillation
AI, artificial intelligence
AMA, American Medical Association
CKD, chronic kidney disease
CV, cardiovascular
CVD, cardiovascular disease
CAD, coronary artery disease
DHI, digital health intervention
EHR, electronic health records
HCP, healthcare provider
HF, heart failure
HFrEF, heart failure with reduced ejection fraction
ICD, implantable cardioverter-defibrillator
IoHT, internet of healthcare things
IT, information technology
MI, myocardial infarction
NYHA, New York Heart Association
RN, registered nurse
SMS, short message service
TAVI, transcatheter aortic valve implantation
WHO, World Health Organization

Table S3.1. Characteristics of 125 included primary studies.

Study	Reference and country	Clinical population	Clinical setting	Type of prevention	WHO 2.0 classification of digital interventions	AMA classification of digital health tools	Aim of DHI	Total HCP sample size (n)	n (%) of male HCPs	HCP background (n)	Method of data collection	Analysis
1	Adler 2020 [1] (Ghana)	Patients with hypertension	Outpatient	Primary	Targeted communication to persons	Patient engagement	Community-based education on CVD risk factors and healthy lifestyle, treatment adherence, and refill reminders through SMS messaging linked to a cloud-based health records system	17	N/A	Nurses (7), physician (1), physician's assistant (1), pharmacist (1), and policy makers (7)	Semi-structured interviews	Thematic content analysis
2	Alaboud 2022 [2] (United States)	Patients with CAD	Outpatient	Secondary	Telemedicine	Remote monitoring and management for improved care and patient engagement	Remote monitoring to support clinical and patient self-management of CAD	7	N/A	Physicians (4), registered nurse (RN) (1), and clinical researchers (2)	Semi-structured interviews	Thematic analysis
3	Alarabyat 2023 [3] (Jordan)	Patients with CVD	Outpatient	Secondary	Telemedicine	Tele visits/virtual visits and remote monitoring and management for improved care	Teleconsultations and remote monitoring for better management of acute and chronic CVD	24	18 (75)	Cardiologists (7), internists (3), and nurses (14)	Semi-structured interviews	Thematic analysis
4	Alessa 2021 [4] (Saudi Arabia)	Patients with hypertension	Outpatient	Primary	Personal health tracking	Patient engagement	Apps to support self-management of hypertension	12	4 (33)	Physicians (12)	Interviews	Framework analysis
5	Al Hemyari [5] 2023 (United States)	General patient population	Outpatient, rural community	Primary and secondary	Telemedicine	Tele visits/virtual visits	Teleconsultations to deliver care, including cardiovascular care	58	35 (60)	Physicians (58)	Survey with Likert scale questi ons	Quantitative descriptive analysis
6	Andersson 2021 [6] (Sweden)	Patients with hypertension	Outpatient	Primary	Telemedicine	Remote monitoring and management for improved care and patient engagement	Interactive web-based system that allows remote monitoring to support self-management of hypertension	15	5 (33)	Physicians (6) and nurses (9)	Focus group	Thematic analysis

7	Aquino 2022 [7] (Canada)	Patients at high risk for preeclampsia	Outpatient	Primary	Telemedicine	Remote monitoring and management for improved care	Telemonitoring programme to support self-care and clinical management of patients at high risk for preeclampsia	5	1 (20)	Physicians (4) and nurse practitioner (1)	Semi-structured interviews	Thematic analysis
8	Artanian 2021 [8] (Canada)	Patients with HF	Outpatient	Secondary	Telemedicine and prescription and medication management	Remote monitoring and management for improved care	Telemonitoring programme for patients with HF to facilitate remote medication titration	5	1 (20)	Cardiologist (1) and nurse (1)	Focus group	Conventional content analysis
9	Auton 2023 [9] (United Kingdom)	Patients with HF with reduced ejection fraction (HFrEF)	Outpatient, nurse-led setting	Secondary	Telemedicine and targeted communication to persons	Remote monitoring and management for improved care and patient engagement	Remote monitoring for nurse-led management of HFrEF, better adherence to guideline-directed medical therapy, facilitated communication with nurses and clinical improvement	6	N/A	Nurses (6)	Semi-structured focus group	Thematic analysis
10	Aw 2020 [10] (Kenya)	General patient population	Outpatient, rural community	Primary	Healthcare provider decision support	Clinical decision support	Algorithmic risk stratification for CVD screening based on SMS text input	1	N/A	Community health worker (1)	Interview	Informal feedback
11	Baratta 2022 [11] (United States)	Patients with hypertension	Outpatient	Primary	Telemedicine	Remote monitoring and management for improved care	Remote blood pressure monitoring to improve management of hypertension	15 (only 10 reported their demos)	3 (30)	Primary care physicians (4), specialist medical doctors (4), pharmacist (1), and nurse (1)	Semi-structured interviews	Inductive and deductive thematic analysis
12	Bazzano 2024 [12] (United States)	Patients with diabetes	Outpatient, low socio-economic setting	Primary	Telemedicine	Tele visits/virtual visits	Teleconsultations to deliver services for diabetes to ensure continuity of care	6	2 (33)	Primary care physicians, endocrinologists, and advanced practice nurses (n value is not specified)	Semi-structured interviews	Reflexive thematic analysis
13	Bergstedt 2024 [13] (United States)	Hospitalized patients	Inpatient	Primary	Healthcare provider decision support	Clinical decision support	Electronic health records (EHR)-embedded tool to promote adherence to American Heart Association practice standards for initiation cardiac telemetry monitoring and duration	16	N/A	Physicians (16)	Semi-structured interviews	Inductive and deductive analysis (using CFIR framework)
14	Bernardi 2020 [14] (United Kingdom)	Patients with HF	Outpatient	Secondary	Telemedicine	Tele-visits/virtual visits and remote monitoring and	Teleconsultations and remote monitoring for community -based	11	7 (64)	Primary care physicians (6), ENT physicians (2),	Semi-structured interviews and observations of	Inductive coding process

						management for improved care	management of patients with HF			public health practitioner (1), social care worker (1), business consultant for telehealth provider (1)	organizational meetings around delivery of telehealth	
15	Bhandari 2021 [15] (Nepal)	Patients with hypertension	Outpatient	Primary	Targeted communication to persons	Patient engagement	Text messaging strategy to optimize self-management of hypertension	11 HCPs and 4 key informants	N/A	Medical officers (2), physicians (4), nurse (1), health assistants/ auxiliary nurses (4), policy/governmental agents (3), and researcher (1)	Semi-structured interviews	Thematic analysis
16	Boriani 2020 [16] (482 HCPs from Europe [77.4%], Asia/Oceania [13.7%], North or South America [8.3%] and 3 [0.6%] in other regions)	Patients at risk for AF	Outpatient	Primary	Healthcare provider decision support	Remote monitoring and management for improved care	AF screening through wearables, devices and apps	588 (only 475 reported their profession)	N/A	Electrophysiologists (176), general cardiologists (84), neurologists/stroke physicians (19), general practitioners (114), internal medicine physicians/ geriatricians (14), nurses/allied health professionals (54), others (14)	Survey with close-ended questions	Quantitative descriptive analysis
17	Bouchard 2024 [17] (Canada)	Patients with HF	Outpatient	Secondary	Telemedicine	Remote monitoring and management for improved care	AI social robots equipped with telemonitoring to assist in the care of patients with HF	22	5 (23)	Cardiologists (7), nurses (4), mental health specialists (3), cardiology residents (2), health services management workers (2), physiotherapists (2), and cardiac rehabilitation physicians (2)	Individual structured interviews	Thematic analysis
18	Breil 2022 [18] (Germany)	Patients with hypertension	Outpatient	Primary	Personal health tracking	Patient engagement	Apps to support self-management of hypertension	46	18 (39)	Physicians (46)	Observational cross-sectional	Qualitative descriptive and

												simple/multiple hierarchical regression
19	Brouns 2024 [19] (The Netherlands)	Stroke patients	Outpatient and inpatient	Secondary	Telemedicine	Remote monitoring and management for improved care and patient engagement	eRehabilitation intervention to support stroke patients and healthcare professionals during inpatient and after discharge rehabilitation	62	N/A	Occupational therapists (17), physical therapists (20), rehabilitation physicians (7), psychologists (4), speech therapists (5), social workers (3), and others (6)	Field notes and in-depth individual interviews	According to the implementation model of Grol
20	Brown 2020 [20] (United States)	Patients with obesity	Outpatient, primary care, rural	Primary	Telemedicine	Tele visits/virtual visits	Behavioural programme with teleconsultations for weight loss and management	22	N/A	Physicians (11), nurses/midwives (6), administrators (5)	Survey with open-ended questions	Qualitative descriptive analysis
21	Brown-Johnson 2021 [21] (United States)	General patient population	Outpatient, primary care	Primary	Telemedicine and	Remote monitoring and management for improved care	Integrated precision health, including remote monitoring of blood pressure, weight, glucose, and activity to provide personalized care	11	N/A	Primary care providers (9) and staff employed for programme intake and orientation (2)	Semi-structured interviews	Consensus qualitative analysis
22	Buis 2020 [22] (United States)	Patients with hypertension	Outpatient, pharmacist-led setting	Primary	Telemedicine	Remote monitoring and management for improved care	Remote blood pressure monitoring and medication adherence tracking to support pharmacist-led hypertension management	2	N/A	Pharmacist (1) and primary care physician (1)	Semi-structured interviews	Thematic analysis
23	Burunkaya 2024 [23] (Türkiye)	Patients with CVD	Hospital N.S. (hospital)	Secondary	N/A General Digital Health	N/A General Digital Health	General digital technology, including wearables, cardiac implantable electronic device telemonitoring, health apps, teleconsultations, and information management to support delivery of cardiovascular care	308	223 (72)	Cardiologists (308)	Survey with close-ended questions	Quantitative descriptive and chi-square tests
24	Cao 2023 [24] (United States)	Patients with CVD eligible for statin therapy	Outpatient	Secondary	Prescription and medication management	Remote monitoring and management for improved care	App to gather patient feedback on muscle symptoms to help diagnose statin intolerance and avoid unnecessary statin discontinuation	5	N/A	Pharmacists (5)	Semi-structured interviews	Thematic analysis

25	Cartledge 2022 [25] (Australia)	Cardiac patients	Outpatient	Secondary	Telemedicine	Tele visits/virtual visits	Teleconsultations to conduct cardiac rehabilitation following a cardiac event or diagnosis	30 (only 17 reported their demos)	3 (18)	RNs (13), exercise physiologists (3), and physiotherapist (1)	Focus group	Inductive thematic analysis
26	Cher 2020 [26] (Singapore)	Patients with AF	Outpatient	Primary	Targeted communication to persons	Patient engagement	Education tool to improve knowledge and self-management of AF	12	N/A	Physicians (4), nurses (4), and pharmacists (4)	Semi-structured interviews	Thematic analysis
27	Chew 2023 [27] (Singapore)	Patients with hypertension	Outpatient, primary care	Primary	Telemedicine	Remote monitoring and management for improved care	Remote blood pressure monitoring for better management of hypertension	8	1 (13)	Family physicians (2), care managers (3), senior nurse clinician (1), and care coordinators (2)	Individual interviews	Inductive thematic analysis
28	Cornell 2023 [28] (Australia)	General patient population	Outpatient, primary care	Primary	Healthcare provider decision support	Clinical decision support	EHR-integrated clinician decision support tool to calculate risk of CVD and to discuss prevention options	12	N/A	General practitioners and nurses (n value is not specified)	Field notes after each practice interaction	Qualitative descriptive
29	Coylewright 2020 [29] (United States)	General patient population	Outpatient	Primary	Personal health tracking	Consumer access to clinical data and patient engagement	EHR-integrated patient decision aids to help patients better visualize personalized risk estimates for CVD risk	109	N/A	Physicians (109)	Survey with open-ended and Likert scale questions	Quantitative descriptive analysis and thematic analysis
30	Danielsbacka 2023 [30] (Sweden)	Patients with CAD	Outpatient	Secondary	Targeted communication to persons	Patient engagement	Digital patient educational programme as part of cardiac rehabilitation after acute CAD	12	2 (16)	Nurses (5) and physiotherapists (7)	Semi-structured interviews	Inductive content analysis
31	Demers 2021 [31] (United States)	Postpartum patients	Outpatient	Primary	Telemedicine	Remote monitoring and management for improved care	Text-based blood pressure monitoring to follow American College of Obstetrics and Gynecology recommendations for postpartum surveillance	2	0 (0)	Physicians (2)	Semi-structured interviews	Thematic analysis
32	Ding 2020 [32] (77 countries, with the most respondents from the United States)	Patients at risk for AF	Outpatient	Primary	Healthcare provider decision support	Point of care/workflow enhancement	Digital devices to screen AF and to initiate earlier use of oral anticoagulant for stroke prevention	1601	N/A	Cardiac electro-physiologists (698), cardiologists (152), electrophysiology fellows (99), cardiology fellows (105), other physicians (50), advanced practice practitioners (186),	Survey with close-ended questions	Quantitative descriptive analysis and univariate logistic regression

										nurses (122), and others (189)		
33	Dullabh 2023 [33] (United States)	Postpartum patients with hypertension	Outpatient	Primary	Telemedicine	Remote monitoring and management for improved care	EHR-integrated app for remote monitoring of postpartum women experiencing hypertensive disorders of pregnancy	4	N/A	Physicians (4)	Semi-structured interviews	Thematic analysis
34	Einhorn 2023 [34] (United States)	Patients with hypertension	Outpatient	Primary	Telemedicine	Remote monitoring and management for improved care	Remote blood pressure monitoring to support management of hypertension and to achieve control of blood pressure	17	N/A	Physicians (11) and nurses/medical assistants (6)	Semi-structured interviews	Inductive thematic analysis
35	Ferguson 2020 [35] (Australia)	General elderly patient population	Outpatient	Primary and secondary	Telemedicine	Remote monitoring and management for improved care	Wearable technology for monitoring heartrate and rhythm to improve the quality of care for older adults and the detection and management of CVD	50	28 (56)	Cardiologists (5), geriatricians (4), allied health professionals (10), junior and resident medical officers and students (15), and nurses (16)	Focus groups	Thematic content analysis
36	Flores-Sandoval 2024 [36] (Canada)	Elderly cardiac patients	Outpatient	Secondary	Telemedicine	Tele visits/virtual visits	Teleconsultations to deliver cardiac rehabilitation to elderly patients	6	2 (33)	Administrative workers, community health workers, health educators, RNs (n value is not specified)	Semi-structured interviews	Deductive analysis
37	Frith 2021 [37] (United Kingdom)	Patients with HF	Outpatient	Secondary	Telemedicine	Remote monitoring and management for improved care and patient engagement	Telemetry system to monitor, provide additional support and increase activation for patients in a remote cardiac rehabilitation programme	3	N/A	Cardiac rehabilitation exercise professionals (3)	Interviews	Thematic analysis
38	Gago 2024 [38] (United States)	Patients with hypertension	Outpatient, primary care	Primary	Telemedicine and targeted communication to persons	Patient engagement and remote monitoring and management for improved care	Virtual high-risk clinic managed by RNs consisting of health coaching to improve medication adherence and promote lifestyle changes and remote blood pressure monitoring for continuous care	67	N/A	N/A	Semi - structured interviews	Deductive thematic analysis

39	Giordan 2022 [39] (Australia)	Patients with HF	Outpatient, primary care, low socio-economic setting	Secondary	Telemedicine, targeted communication to persons, and personal access health tracking	Patient engagement, remote monitoring and management for improved care, and consumer access to clinical data	Patient-facing app allowing remote monitoring of vital signs, communication with provider, visualization of health data to support self-management of HF	6	1 (17)	General practitioners (2), clinical pharmacist (1), dietitian (1), exercise physiologist (1), and counsellor (1)	Semi-structured interviews	Inductive thematic analysis
40	Gomis-Pastor 2020 [40] (Spain)	Heart transplant patients	Heart transplant outpatient clinic	Secondary	Targeted communication to persons and prescription and medication management	Patient engagement and improved care and consumer access to clinical data	Mobile tool to improve medication safety and effectiveness and patient-provider interactions and to help providers deliver comprehensive clinical care for heart transplant recipients	26	9 (35)	Physicians (6), nurses (3), pharmacists (5), psychologists (2), technology analysts (2), key representatives of local health authorities (3), representatives of regional health authorities (2), and experts in mHealth (3)	Survey with open-ended questions	Descriptive qualitative analysis
41	Gondi 2021 [41] (United States)	Patients with hypertension	Outpatient, primary care	Primary	Telemedicine	Remote monitoring and management for improved care	Remote blood pressure monitoring to improve hypertension management	17	2 (12)	Physicians (17)	Survey with open-ended questions	Qualitative analysis according to theoretical domains framework
42	Gordon 2022 [42] (Canada)	Patients with multiple and complex chronic conditions, including HF, hypertension, diabetes	Outpatient, primary care, nurse-led setting	Primary and secondary	Telemedicine	Remote monitoring and management for improved care	Mobile telemonitoring platform to support nurse-led services for patients with multiple and complex chronic conditions, including CVD	7	0 (0)	Frontline providers (4) and management administrators (3)	Likert-scale questionnaires and semi-structured interviews	Quantitative descriptive and deductive thematic analysis
43	Gregory 2023 [43] (United States)	Patients with cancer	Outpatient	Primary	Prescription and medication management	Remote monitoring and management for improved care	Symptom-tracking tool for early recognition of cardiotoxicity in patients with cancer after cancer therapy initiation	11	N/A	General cardiologists (2), oncologists (2), hematologists (3), cardio-oncologists	Semi-structured interviews	Thematic analysis

										(3), and radiation oncologist (1)		
44	Grey 2023 [44] (United Kingdom)	Patients with cardiovascular or rheumatic diseases	Outpatient	Secondary	Telemedicine	Tele visits/virtual visits	Remote consultations to deliver care for people with chronic conditions in cardiology and rheumatology	12	7 (58)	Physicians (7) and nurses (5) in cardiology (6) and rheumatology 6)	Semi-structured interviews	Thematic analysis
45	Gupta 2023 [45] (United States)	Patients with hypertension	Outpatient, primary care	Primary	Telemedicine, targeted communication to persons and prescription and medication management	Remote monitoring and management for improved care, patient engagement, and tele visits/ virtual visits	Virtual collaborative care clinic including tele visits to prescribe medication, remote blood pressure monitoring and frequent communication with provider to support self-management of hypertension	11	N/A	Physicians (6) and pharmacists (5)	Semi-structured interviews	Combination of inductive coding and template analysis
46	Hammersley 2020 [46] (United Kingdom)	People with hypertension	Outpatient, primary care	Primary	Telemedicine	Remote monitoring and management for improved care and remote monitoring for efficiency	EHR-integrated remote blood pressure monitoring system to improve blood pressure control	8	N/A	General practitioners, practice nurses, and healthcare assistants (n value is not specified)	Semi-structured interviews	Thematic analysis
47	Harris 2022 [47] (United Kingdom)	Patients with stable angina	Inpatient	Secondary	On demand communication with persons	Patient engagement	Patient decision aid to support patients with stable angina in deciding between continuing cardiac medicines or opting for elective percutaneous coronary intervention	9	5 (56)	Cardiologists (4), cardiology specialist nurses (4), and interventional radiology matron (1)	Semi-structured interviews	Deductive thematic analysis
48	Harvey 2022 [48] (United States [n=40], United Kingdom [n=3], Australia [n=1], Egypt [n=1])	Patients with some form of implantable cardiac device (ICD)	Outpatient	Secondary	Healthcare provider decision support and telemedicine	Remote monitoring and management for improved care	Remote monitoring clinic for ICDs for arrythmia screening	45	N/A	RNs (19), nurse practitioners (14), physician's assistants (2), technicians (5) and others (5)	Likert-scale questionnaire and survey	Quantitative descriptive and thematic analysis
49	Hassan 2023 [49] (United Kingdom)	General patient population	Outpatient, primary dental care practice	Primary	Healthcare provider decision support	Point of care/workflow enhancement	Electronic device to screen AF in primary dental care	11	6 (54)	Managers (3), dentists, dental therapists, dental	Semi-structured interviews	Thematic framework analysis

										nurses (n value is not specified)		
50	Hauschildt 2023 [50] (United States)	Patients with risk factors for CVD	Outpatient, primary care	Primary	Healthcare provider decision support	Clinical decision support	Clinical decision support tool for CVD risk management	44	N/A	N/A	Survey with open-ended questions	Thematic analysis
51	Hellem 2024 [51] (United States)	Patients with hypertension	Outpatient	Primary	Telemedicine	Remote monitoring and management for improved care	Remote blood pressure monitoring to inform hypertension treatment and enhance BP control	10	N/A	HCPs (4) and administrative workers (6) (n value is not specified)	Semi-structured interviews	Content analysis
52	Honein-AbouHaidar 2020 [52] (Lebanon)	Patients with diabetes or hypertension	Outpatient	Primary	Personal health tracking and targeted communication to persons	Consumer access to clinical data and patient engagement	Electronic patient portals that enable patients to access their clinical summaries and communicate directly with their providers to increase engagement in self-managing their condition	17	1 (6)	N/A	Survey with close-ended questions and Likert-scale-type questions	Quantitative descriptive analysis based on the Technology Acceptance Model
53	Houle 2020 [53] (Canada)	Patients with CAD	Outpatient	Secondary	Telemedicine	Remote monitoring and management for improved care and patient engagement	Combined socio-cognitive and pedometer-based intervention to support cardiac rehabilitation following acute CAD	16 HCPs and 13 managers	3 (19) for HCPs<br><br>2 (15) for managers	Nurses (6), nurse practitioners (2), kinesiologists (6), physicians (2), cardiac and/or respiratory services managers (8), chronic disease program managers (4), assistant directors of nursing (1)	Focus groups	Thematic analysis
54	Indraratna 2022 [54] (Australia)	Patients with acute coronary syndrome (ACS) and heart failure	Outpatient	Secondary	Telemedicine	Remote monitoring and management for improved care and patient engagement	Telemonitoring app to improve clinical and self-management of patients of ACS and heart failure	10	NA	Teleclinical care team members (4), cardiologists (3), and general practitioners (3)	Semi-structured interviews	According to the Framework Method for analysis of qualitative data
55	Islam 2022 [55] (Australia)	Patients with HF	Outpatient	Secondary	Telemedicine and personal health tracking	Remote monitoring and management for improved care and consumer access to clinical	A smart home system to allow users to access health data, while clinicians can monitor health status and communicate to improve self-management and	9	2 (22)	Nurses (4), cardiologists (3), general practitioner (1), and pharmacist (1)	Semi-structured interviews	Inductive thematic analysis

						data and patient engagement	clinical care in people with HF					
56	Jongsma 2021 [56] (The Netherlands)	Patients at high risk of hypertensive disorders of pregnancy (HPD)	Outpatient	Primary	Telemedicine	Remote monitoring and management for improved care	Remote monitoring of patients at high risk of HPD for rapid intervention, medication management, and hospital admission prevention	14	1 (7)	Nurses (6), residents (2), obstetricians (6)	Semi-structured interviews	Deductive and inductive thematic analysis
57	Kalinina 2020 [57] (Russian Federation)	Patients with chronic disease, in particular arterial hypertension	Outpatient	Primary	Telemedicine	Remote monitoring and management for improved care	Telemedicine technologies in dispensary observation of patients with chronic disease, in particular with arterial hypertension	93	14 (15)	Primary care physicians (93)	Survey with close-ended questions	Descriptive analysis
58	Kalinina 2021 [58] (Russian Federation)	Patients with chronic disease, in particular arterial hypertension	Clinics in two regional area (outpatient)	Primary	Telemedicine	Remote monitoring and management for improved care	Telemedicine technologies in dispensary observation of patients with chronic disease, in particular with arterial hypertension	284	50 (18)	Primary health care physicians (282)	Survey with close-ended questions	Descriptive analysis
59	Kauw 2020 [59] (The Netherlands)	Cardiac patients	Outpatient	Secondary	Telemedicine	Remote monitoring and management for improved care	Mobile health allowing remote monitoring for rapid remote diagnosis and improved care for cardiac patients	13	7 (13)	Cardiologists (3), cardiac nurses (2), professors in medicine (3), hospital manager (1), outpatient clinic manager (1), hospital board member (1), director of outpatient clinic (1), director of national IT healthcare institute (1)	Semi-structured interviews	Inductive thematic analysis
60	Kerr 2020 [60] (Ireland)	Patients with HF	Outpatient	Secondary	Telemedicine	Tele visits/virtual visits	Disease management programmes for delivering community HF care through virtual consults or structured telephonic assessments	N/A	N/A	N/A	Observational cross-sectional	Quantitative descriptive analysis

61	Khonsari 2020 [61] (Iran)	Patients with coronary heart disease	Outpatient	Secondary	Targeted communication to persons	Patient engagement	Text messaging technology to improve ACS patients' adherence in medication taking	23	4 (17)	Nurses (23)	Focus group	Thematic analysis
62	Klaiman 2023 [62] (United States)	Patients with HF	Outpatient	Secondary	Telemedicine and prescription and medication management	Remote monitoring and management for improved care and patient engagement	EHR-integrated remote monitoring to track weight and diuretic adherence and to promote treatment adherence for patients with HF	16	N/A	Physicians (9), nurse practitioners (2), nurses (5)	Semi-structured interviews	Thematic analysis
63	Kleiner 2022 [63] (Hungary)	General patient population	Outpatient	Primary	Personal health tracking	Patient engagement	A software to identify risks of inactive lifestyle, weight problems, diabetes mellitus or cardiovascular event, and help in the personal health planning and management of the risks	75	N/A	Pharmacists (75)	Surveys with close-ended questions	Quantitative descriptive analysis
64	Kobe 2022 [64] (United States)	Patients with clinic-refractory type 2 diabetes	Outpatient, rural setting	Primary	Telemedicine	Patient engagement and remote monitoring and management for improved care	Telemonitoring to facilitate delivery of comprehensive care and improve self-management of clinic-refractory type 2 diabetes	15	N/A	Veterans' Health Administration staff and administrators (15)	Semi-structured interviews	Thematic analysis and the matrix method
65	Kocanda 2021 [65] (Australia)	Patients with CVD or at risk for CVD	Outpatient, rural setting	Primary and secondary	Telemedicine	Tele visits/virtual visits	Teleconsultations to enhance services for cardiovascular care	10	1 (10)	Physicians (2), nurses (3), allied health professionals (4), and hospital administrator (1)	Semi-structured interviews	Thematic analysis
66	Kołtowski 2021 [66] (Poland)	Patients with CVD	Outpatient	Secondary	Telemedicine	Tele visits/virtual visits	Teleconsultations for delivery of care for patients with CVD	8	N/A	Cardiologists (5) and cardiology residents (3)	Individual interviews	Qualitative descriptive
67	Ladegaard 2022 [67] (Denmark)	Frail elderly patients with AF	Outpatient, primary care	Primary	Referral coordination and healthcare provider communication	Point of care/ workflow enhancement	Compact heart sensor to facilitate referrals from general practitioner to cardiologist and to provide remote support by cardiologists for general practitioners	2	N/A	General practitioner (1) and nurse (1)	Semi-structured interviews	Manifest qualitative content analysis
68	Leonard 2023 [68] (United States)	Patients with HF	Outpatient	Secondary	Telemedicine	Tele visits/virtual visits	Teleconsultations for management of HF	17	N/A	Physician, clinical pharmacist, nurse (n value is not specified)	Semi-structured interviews	Content analysis

69	Lino 2023 [69] (Brazil)	Patients with hypertension or diabetes	Outpatient, primary care	Primary	Telemedicine	Remote monitoring and management for improved care	Telemonitoring for the follow-up of patients with hypertension or diabetes	65	9 (14)	Nurses (65)	Survey with close-ended questions	Exploratory Analysis
70	Lukey 2023 [70] (Canada)	Patients with HF	Outpatient	Secondary	Targeted communication to persons	Patient engagement	Website focused on patient education to support self-management of HF	8	N/A	Nurses (8)	Focus group and individual interviews	Thematic analysis
71	Lyles 2023 [71] (Kenya)	Patients with hypertension or diabetes	Outpatient, primary care, refugee camp setting	Primary	Scheduling and activity planning for healthcare providers	Point of care/ workflow enhancement	Clinician-controlled app consisting of abbreviated medical records to improve the coverage and quality of care for hypertension or diabetes	6	N/A	N/A	Individual interviews	Thematic analysis
72	Mahmoud 2023 [72] (Nigeria)	Patients with hypertensive disorders of pregnancy (HDP)	Outpatient, tertiary care	Primary	Telemedicine	Remote monitoring and management for improved care	Home blood pressure monitoring program in the management of hypertensive disorders of pregnancy	22	N/A	Physicians (11), nurses/midwives (6), and administrators (5)	Semi-structured interviews and focus groups	Qualitative analysis with a combined inductive and deductive process according to Consolidated Framework for Implementation Research
73	Maria 2022 [73] (Portugal)	Patients with CVD	Outpatient, transition from primary care to secondary care	Secondary	Telemedicine and referral coordination	Tele visits/virtual visits and point of care/ workflow enhancement	Teleconsultations involving the general practitioner, patient, caregiver, and cardiologist as alternatives to in-person referrals to cardiology	11	3 (27)	General practitioners (7) and cardiologists (4)	Semi-structured interviews	Conventional content analysis
74	Marzolini 2021 [74] (Canada)	Cardiac patients	Outpatient	Secondary	Telemedicine	Tele visits/virtual visits	Virtual group-based or single sessions or web-based platforms that provide resources for cardiac rehabilitation	114	N/A	n value not specified	Survey with close and open-ended questions	Qualitative and quantitative descriptive
75	Matthiesen 2021 [75] (Denmark)	Patients with an implantable cardioverter defibrillator (ICD)	Outpatient	Secondary	Healthcare provider decision support	Clinical decision support	Machine learning-based tool for predict ventricular tachycardia/ventricular fibrillation to support clinical decision-making in the remote monitoring of patients with an ICD	7	6 (86)	Cardiologists (7)	Semi-structured interviews	Inductive qualitative approach based on grounded theory

76	McCarthy 2024 [76] (United States)	General patient population	Outpatient, ambulatory preventive cardiology setting	Primary	Healthcare provider decision support	Clinical decision support	Three-question Physical Activity Vital Sign embedded in EHR to alert providers to patients reporting low physical activity and prompt providers to counsel patients as needed	4	N/A	Cardiologists (3) and nurse practitioner (1)	Semi-structured interviews	Inductive content analysis
77	McGillion 2020 [77] (Canada [n=15] and United Kingdom [n=11])	Patients post cardiac or major vascular surgery	Inpatient and outpatient	Secondary	Telemedicine	Remote monitoring and management for improved care	1- In-hospital remote monitoring to identify patients with early signs of clinical deterioration to facilitate prompt intervention<br><br>2- At-home remote monitoring to detect early signs of postoperative complications	26	4 (15)	Nurses (26)	Semi-structured interviews	Inductive thematic content analysis
78	Meador 2024 [78] (United States)	Patients with high blood pressure	Outpatient	Primary	Telemedicine	Remote monitoring and management for improved care	Remote blood pressure monitoring to improve blood pressure control and to confirm a new diagnosis of hypertension before initiating treatment	65	N/A	Physicians (36), nurse practitioners (19), physician assistants, pharmacists, and executive directors (10)	Surveys with close-ended questions, Likert scale and open-ended questions	Thematic analysis and quantitative descriptive analysis
79	Mehta 2020 [79] (United States)	Patients with HF	Outpatient	Secondary	Telemedicine	Remote monitoring and management for improved care	Wearable fitness tracker to evaluate NYHA class and prognosis and to inform clinical decision-making	6	N/A	Cardiologists (6)	Survey with close-ended questions	Quantitative descriptive analysis
80	Morton 2021 [80] (United Kingdom)	Patients with uncontrolled hypertension	Outpatient, primary care	Primary	Telemedicine	Remote monitoring and management for improved care	Remote blood pressure to lower blood pressure in patients with uncontrolled hypertension	27	9 (33)	General practitioners (13), healthcare assistants (2), general practice manager (1), and nurses (11)	Semi-structured interviews	Quantitative descriptive statistics and reflexive thematic analysis
81	Muehlensiepen 2024 [81] (Germany)	Patients with CVD or at risk for CVD	Outpatient	Primary and secondary	Telemedicine	Tele visits/virtual visits and remote monitoring and management for improved care	Telecardiology consultations and remote monitoring to provide adequate and personalized cardiology care	97	54 (56)	Physicians (97) (cardiologists, internists, and general practitioners) (n value is not specified)	Survey with close-ended questions	Multidimensional scaling and k-means clustering

82	Nau 2021 [82] (Australia)	Patients with hypertension	Outpatient, primary care	Primary	Targeted communication to persons	Patient engagement	Mobile intervention to deliver lifestyle advice concerning diet and physical activity and to encourage medication adherence to support self-management of hypertension	7	N/A	General Practitioners (7)	Semi-structured interviews	Thematic analysis
83	Nayyar 2022 [83] (Canada)	Patients with ambulatory care sensitive conditions, including hypertension, AF, HF, diabetes	Outpatient, specialty clinic	Primary and secondary	Telemedicine	Tele visits/virtual visits	Virtual visits for improved management of ambulatory care sensitive conditions	34 (14 focus group participants and 20 survey participants)	Focus group: 5 (36)<br><br>Survey: 0 (0)	Focus group: cardiologists (3), endocrinologists (5), respirology physicians (2), general medicine physicians (2), and administrative staff (2)<br><br>Survey: allied health professionals (8), physicians (7), nurse practitioners (3), and nurses (2) in divisions of: cardiology (6), endocrinology (13), general medicine (1)	Survey with close and open-ended questions and a focus group	Interpretive description approach and quantitative descriptive analysis
84	Nemis-White 2021 [84] (Canada)	Patients with AF	Outpatient, primary care	Primary	Healthcare provider decision support	Clinical decision support	Clinical decision support software to deliver individualized guideline-based AF patient care	N/A	N/A	N/A	Feedback obtained from randomized controlled trial	Qualitative descriptive
85	Otieno 2023 [85] (Kenya)	Patients with hypertension and type 2 diabetes	Outpatient and inpatient	Primary	Scheduling and activity planning for healthcare providers	Point of care/ workflow enhancement	National health management information system to provide integrated and connected outpatient and inpatient care services for hypertension and type 2 diabetes	44	27 (61)	Health managers (11), healthcare workers (24), national policymakers (5), key persons from non-governmental organisations (4), physicians, clinical officers, nurses, pharmacists and laboratory technologists (n	Focus groups and individual interviews	Deductive and inductive thematic analysis

										value is not specified)		
86	Palozzi 2020 [86] (Italy)	Patients with chronic cardiac disease	Outpatient (Hospital discharge)	Secondary	Telemedicine	Remote monitoring and management for improved care	General IoHT (Internet of healthcare things) technologies for the remote management of cardiac chronicity	5	N/A	Physicians (5)	Semi-structured interviews	Qualitative content analysis
87	Pelly 2023 [87] (Australia)	Patients with myocardial infarction (MI)	Outpatient	Secondary	Targeted communication to persons	Patient engagement	Digital health to support self-management for patients with a history of MI and to prevent subsequent MI events	16	N/A	General physician, cardiac nurses, physiotherapists, aged care emergency attendants, rehabilitation consultants, cardiac rehabilitation specialists (n value is not specified)	Focus groups	Qualitative descriptive
88	Piskorz 2022 [88] (Latin-American countries)	Patients with hypertension	Outpatient	Primary	Telemedicine	Tele visits/virtual visits and remote monitoring and management for improved care	Remote blood pressure monitoring and telemedicine to improve clinical management of patients with hypertension	1753	1084 (62)	Cardiologists (714), internists (245), general practitioners (197), and others (523)	Survey with close-ended questions	Quantitative descriptive analysis and bivariate logistic regression
89	Portz 2020 [89] (United States)	Patients with HF	Outpatient	Secondary	Telemedicine	Remote monitoring and management for improved care	mHealth to pair patient/ caregiver-generated data with clinical intervention to improve clinical management of patients with HF	20	4 (20)	Advanced practice nurses (7), art/music therapists (2), chaplains/spiritual care (2), physicians (4), RN (3), and social workers (2)	Semi-structured interviews	Phenomenological thematic analysis

90	Queiroz 2024 [90] (Portugal)	Patients with CVD and at risk for CVD	Hospital and university	Primary and secondary	N/A General digital health	N/A General digital health	General digital health to help promote cardiovascular health and treat cardiovascular disease	117	55 (47)	Medical specialists (74), cardiopulmonary technicians/clinical physiology technicians (28), medical residents (9), nurses (5), and researchers (1)	Survey with close-ended questions	Quantitative descriptive analysis
91	Rakers 2024 [91] (The Netherlands)	Patients with cardiovascular risk factors	Outpatient, primary care	Primary	Telemedicine	Remote monitoring and management for improved care	Remote patient management to assist in achieving treatment targets for cardiovascular risk factors and improving lifestyle	24	5 (26) for HCPs only<br><br>11 (46) in total	General practitioners (9) and practical nurses (10) and other stakeholders (5)	Semi-structured interviews and focus groups	Thematic analysis
92	Riboli-Sasco 2024 [92] (United Kingdom)	High-risk patients with hypertension	Outpatient, primary care	Primary	Telemedicine	Remote monitoring and management for improved care	Remote blood pressure monitoring to titrate antihypertensive medication, better manage hypertension, and shift care from general practitioners to a multidisciplinary team	20	9 (45)	General practitioners (6), clinical pharmacists (3), practice nurse managers/leads and nursing associates (3), program/practice managers (8)	Semi-structured interviews and focus groups	Thematic analysis
93	Rodrigues 2021 [93] (The Netherlands)	Patients with CVD	Outpatient	Secondary	Targeted communication to persons	Patient engagement	eHealth tools to support lifestyle changes for patients with CVD	16	6 (38)	Physicians (4), nurses (7), physician assistant (1), physiotherapists (2), psychologist (1), and life coach (1)	Semi-structured interviews	Content analysis
94	Saifan 2022 [94] (Jordan)	Patients with CVD	Outpatient	Secondary	Telemedicine	Tele visits/virtual visits and remote monitoring and management for improved care	Remote monitoring and virtual consultations for management of critical and long-reaching cardiovascular health issues	12	9 (75)	Cardiologists (4), internist (1), and nurses (7)	Semi-structured interviews	Thematic analysis
95	Sanderson 2021 [95] (Canada)	Patients with CVD	Outpatient, secondary care, cardiology	Secondary	Telemedicine	Tele visits/virtual visits	Virtual consultations to deliver cardiac care to patients with CVD	26	N/A	Staff physicians (13), physician trainees (8), nurse practitioners (2), allied health professionals (2), and practical nurse	Survey with open-ended questions	Quantitative descriptive analysis and content analysis

										(1), all in the field of cardiology		
96	Santana 2020 [96] (Canada)	Patients with CVD	Outpatient	Secondary	Targeted communication to persons	Patient engagement	General eHealth to facilitate communication of between cardiac patients and providers	7	4 (57)	Physicians and nurses with specialties in CV care, endocrinology, nephrology, and internal medicine (n value is not specified)	Workshops that used participatory health research	Thematic analysis using a deductive process
97	Santos 2023 [97] (Brazil)	Cardiac patients	Outpatient, low-resource setting	Secondary	Targeted communication to persons	Patient engagement	Virtual educational programme to support behaviour changes for cardiac patients	8	N/A	Physiotherapists (8)	Survey with open-ended questions and individual interviews	Qualitative interpretative descriptive
98	Savickas 2020 [98] (United Kingdom)	General patient population	Outpatient, primary care, pharmacist-led setting	Primary	Healthcare provider decision support	Remote monitoring for efficiency	Novel ECG devices for AF screening	13	2 (15)	Clinical pharmacists (4) and general practice staff (9)	Focus group	Deductive Theoretical Domains Framework approach
99	Schepart 2023 [99] (United States)	Patients with CVD	N.S. (hospital)	Secondary	N/A<br>General (AI) digital health	N/A<br>General (AI) digital health	Various AI-enabled technologies to allow the earlier detection of disease, risk-stratifying patients for targeted interventions, and identifying gaps and facilitating improvements in quality of cardiovascular care	140 (20 interview participants and 120 survey participants)	N/A	Interview: cardiologists (12) and health information technology administrators (8)<br><br>Survey: cardiologists (90) and IT administrators (30)	Survey and focus groups/ semi-structured interview	Grounded theory approach and quantitative descriptive analysis
100	Schertzer 2024 [100] (Canada)	Patients with cardiac or respiratory conditions	Outpatient	Secondary	Telemedicine	Tele visits/virtual visits	Teleconsultations to conduct cardiac and pulmonary rehabilitation	7	0 (0)	Physiotherapists (7)		Conventional content analysis

101	Schott 2021 [101] (United States)	Patients with AF	Outpatient and inpatient	Primary	Healthcare provider decision support	Clinical decision support	EHR-integrated decision aid to help with decision making around stroke prevention in AF	9	4 (44)	Attending cardiologists (2), cardiology fellows (2), advance practice providers (2), and primary care attendings (3)	Semi-structured interviews	Inductive thematic analysis
102	Sideris 2024 [102] (United States)	Patients with HF	Outpatient	Secondary	Telemedicine and healthcare provider decision support	Remote monitoring and management for improved care and clinical decision support	AI-powered continuous wearable monitoring to predict heart failure decompensation	14	N/A	Physicians, nurse practitioners, RN, and pharmacists (n value is not specified)	Semi-structured interviews	Thematic analysis
103	Singh 2022 [103] (India)	Patients with CVD	Outpatient	Secondary	Healthcare provider decision support and targeted communication to persons	Clinical decision support and patient engagement	Strategy which combines EHR-decision support system and text messaging to offer patients lifestyle advice to improve cardiovascular care quality	56	41 (73)	Physicians (38), non-physician health workers (14) and health administrator or policy maker (4)	Semi-structured interviews	Iterative approach based on the framework method for qualitative research and Consolidated Framework for Implementation Research domains
104	Singhal 2023 [104] (United Kingdom)	Patients with HF	Outpatient, secondary care, cardiology	Secondary	Telemedicine	Tele visits/virtual visits	Teleconsultations for improved management of heart failure	8	4 (50)	Cardiologists (4), specialist nurses (3), and rotational training-grade doctor (1)	Semi-structured interviews	Thematic analysis
105	Sivakumar 2022 [105] (Canada)	Patients with HF	Outpatient	Secondary	Targeted communication to persons	Patient engagement	Mobile health apps to support patients with HF treatment adherence and self-management	21	4 (19)	Cardiologists (8), nurses (6), and nurse practitioners (7)	Semi-structured interviews	Content analysis
106	Sobreiro 2020 [106] (Portugal)	Patients with CAD	N.S. (hospital)	Secondary	Targeted communication to persons	Patient engagement	Information and communication technologies to help populations obtain accurate information about CVD, enabling access to preventive services and improved self-care	19	10 (58)	Nurses (11), physiotherapists (2), physicians (3), and cardiac physiologists (3)	Focus group	Content textual analysis

107	Steinman 2020 [107] (Cambodia)	Patients with diabetes and hypertension	Outpatient, hard-to-reach population, peer educator program	Primary	1- Targeted communication to persons<br><br>2- Person-centred health records	1- Patient engagement<br><br>2- Point of care/ workflow enhancement	1- Mobile voice messages to provide education on disease management, support and reminders for medication adherence, monitoring, and provider consultations<br><br>2- Tablets for peer educators to facilitate longitudinal tracking and access of patient health data and to support community-clinical linkages	6	N/A	Peer educators (6)	Interviews	Deductive thematic analysis
108	Steinman 2020 [108] (Cambodia)	Patients with diabetes and hypertension	Outpatient	Primary	Telemedicine and targeted communication to persons	Patient engagement and remote monitoring and management for improved care	App to monitor remotely blood glucose levels, weight, exercise, and symptoms and to deliver educational content to improve self-management of diabetes and hypertension	11	6 (55)	Peer educators (11)	Interviews	Deductive thematic analysis
109	Suen 2024 [109] (Australia)	Patients with type 2 diabetes or CVD patients	Outpatient and inpatient, primary care	Primary and secondary	Healthcare provider decision support	Clinical decision support	Clinical decision support tool to recommend SGLT2 inhibitor prescribing for patients with type 2 diabetes to reduce CVD and chronic kidney disease (CKD) risk	16	5 (31)	General practitioners (8), practice nurses (6), practice manager and practice nurse (1), and clinical health assistant (1)	Semi-structured interviews	Thematic analysis
110	Teo 2023 [110] (Singapore)	Patients with hypertension	Outpatient, primary care	Primary	Telemedicine	Tele visits/virtual visits and remote monitoring and management for improved care	Remote blood pressure monitoring combined with teleconsultations to titrate medication if necessary and improve blood pressure control	8	1 (13)	Family physicians (2), nurse clinician (1), care managers (3), and care coordinators (2)	Semi-structured interviews	Inductive analysis
111	Thomas 2023 [111] (Australia)	Patients in cardiac and pulmonary rehabilitation	Outpatient	Secondary	Telemedicine	Tele visits/virtual visits and remote monitoring and management for improved care	Remote monitoring with teleconsultations to provide long-term self-management strategies for patients in cardiac and pulmonary rehabilitation	27	8 (30)	Clinical nurses (14), physiotherapists (11), exercise physiologists (2)	Semi-structured interviews and focus groups	Hybrid inductive/deductive framework analysis

112	Thorup 2022 [112] (Denmark)	Patients post Transcatheter Aortic Valve Implantation (TAVI)	Outpatient	Secondary	Targeted communication to persons and personal health tracking	Patient engagement	Program with supervised home-based video training, web sessions with a cardiac nurse, an informative website, and an activity tracker to support cardiac rehabilitation after TAVI	4	2 (50)	Nurses (2) and physiotherapists (2)	Semi-structured interviews	Inductive content analysis
113	Tighe 2022 [113] (Australia)	Patients with CVD	Outpatient	Secondary	On demand communication with persons	Patient engagement	Unifying platform that hosts a digitally supported palette of evidence-based health interventions and includes a guidance tool to assist patients in selecting interventions for self-management of CVD	8	2 (25)	NA	Semi-structured interviews and a focus group	Inductive thematic analysis
114	Toft 2024 [114] (Denmark)	Elderly patients post cardiac surgery	Outpatient	Secondary	Telemedicine	Remote monitoring and management for improved care	Mobile app to support remote exercise-based cardiac rehabilitation of elderly patients early after cardiac surgery	2	0 (0)	Physiotherapists (2)	Semi-structured interviews	Thematic analysis
115	Tsukada 2021 [115] (Japan)	Elderly Patients with HF	Outpatient and inpatient	Secondary	Referral coordination	Point of care/ workflow enhancement	Information and communications technology for sharing patient information between acute care hospitals and general practitioners in regional healthcare networks	392	352 (87)	General practitioners (392)	Survey with close-ended questions	Quantitative descriptive analysis
116	Van Emmenis 2022 [116] (United Kingdom)	Patients with hypertension	Outpatient, primary care	Primary	Targeted communication to persons	Patient engagement	Text messaging or mobile app intervention to support hypertension medication adherence	11	1 (9)	General practitioner (1), healthcare assistants (2), practice nurses (6), and practice pharmacists (2)	Semi-structured interviews	Analysis informed by the constant comparative approach and the perceptions and practicalities approach
117	Vandeker-ckhove 2020 [117] (Belgium)	Patients with CVD	Outpatient, secondary care	Secondary	Telemedicine	Tele visits/virtual visits	Teleconsultations in the outpatient clinic with the patient, nurse, and cardiologist to replace in-person cardiology consultations	4	N/A	Lead hospital IT manager (1), research nurse (1), the treating cardiologist (1), and the head of the	Interviews	Qualitative descriptive

										Department of Cardiology (1)		
118	Varnfield 2021 [118] (Australia)	Patients with gestational diabetes	Outpatient	Primary	Telemedicine and targeted communication to persons	Patient engagement and remote monitoring and management for improved care	App for remote monitoring of blood glucose level, weight, exercise, and symptoms and delivery of motivational prompts and educational media for patients with gestational diabetes	N/A	N/A	Clinicians and diabetes educators (n value per type of HCPs not specified)	Survey with open-ended questions	Qualitative descriptive
119	Vugs 2023 [119] (The Netherlands)	Patients with HF	Outpatient	Secondary	Telemedicine	Remote monitoring and management for improved care	Telemonitoring to manage deterioration of HF	8	N/A	Cardiologist (1), heart failure nurses (5), general practitioners (2)	Semi-structured interviews	Reflexive thematic analysis
120	Waldman 2022 [120] (United States)	Patients with CVD	Outpatient	Secondary	Telemedicine	Tele visits/virtual visits	Teleconsultations to deliver cardiovascular care	342	255 (75)	Cardiologists (315), advanced practice nurses (12), physician assistants (4), nurses (4), practice administrators (3), pharmacist (1), and other (2)	Qualitative survey with multiple-choice and open-ended questions	Qualitative descriptive analysis and $\chi 2$**,** Mann-Whitney and one-way analyses
121	Wali 2021 [121] (Canada)	Patients with HF	Outpatient	Secondary	Telemedicine	Remote monitoring and management for improved care	Mobile telemonitoring program to provide remote clinical support for patients with HF	13	N/A	Clinicians including cardiologists and nurses (9) and operational staff (4)	Semi-structured interviews	Inductive thematic analysis
122	Wali 2023 [122] (Canada)	Pediatric patients with CVD	Outpatient, pediatric setting	Secondary	Telemedicine and targeted communication to persons	Patient engagement and remote monitoring and management for improved care	Digital therapeutics for remote monitoring of weight, blood and symptoms to automatically generate individualized self-care instructions to adolescents with CVD and simultaneously alert	8	N/A	Cardiologists (4), nurse practitioners (3) and cardiology fellow (1)	Semi-structured interviews	Inductive thematic analysis

							clinicians of changes to health status				
123	Watson 2024 [123] (Canada)	Patients with diabetes, kidney, and cardiovascular chronic conditions	Outpatient	Primary and secondary	Targeted communication to persons	Patient engagement	Tool to offer sick-day medication guidance and to improve self-management of diabetes, kidney, and cardiovascular conditions	28	10 (36)	Pharmacists (13), primary care physician (12), nurse practitioners (3)	Focus groups and individual interviews	Inductive analysis
124	Yao 2023 [124] (China)	General patient population	Outpatient, primary care	Primary	Healthcare provider decision support	Remote monitoring and management for improved care	Wearable electrocardiograph device to screen patients for AF	1,004	378 (38)	Clinicians from primary health care facilities (1,004)	Survey with close-ended questions	Bivariate correlation analysis and SEM analysis
125	Zolezzi 2023 [125] (Qatar)	General patient population	Outpatient, primary care	Primary	Healthcare provider decision support	Clinician decision support	Mobile tool to assess CVD risk to guide CVD prevention and management strategies	10	6 (60)	Community pharmacists (10)	Semi-structured interviews	Deductive content analysis

Table S3.2. Distribution of the number of studies and healthcare providers across countries. N.A., not applicable due to missing data. N.A. in the column 'number of studies' refers to that country having been included in a study involving multiple countries. In terms of 'number of healthcare providers', the studies Nemis-White (2021) for Canada, Varnfield (2021) for Australia, and Kerr (2020) for Ireland did not provide sample sizes and were treated as missing data.

Country	Number of studies	Number of healthcare providers
Australia	13	201
Belgium	1	4
Brazil	2	73
Cambodia	2	17
Canada	17	340
China	1	1004
Denmark	4	15
Egypt	N.A.	1
Germany	2	143
Ghana	1	17
Hungary	1	75
India	1	56
Iran	1	23
Ireland	1	N.A.
Italy	1	5
Japan	1	392
Jordan	2	36
Kenya	3	51
Lebanon	1	17
Nepal	1	15

Nigeria	1	22
Poland	1	8
Portugal	3	147
Qatar	1	10
Russian Federation	2	377
Saudi Arabia	1	12
Singapore	3	28
Spain	1	26
Sweden	2	27
The Netherlands	6	137
Turkey	1	308
United Kingdom	12	139
United States	30	1082
Studies across multiple countries	5	4,013
**Total**	**125**	**8,821**

References

1. Adler, A. J. *et al.* Barriers and facilitators to the implementation of a community-based hypertension improvement project in Ghana: a qualitative study of ComHIP. *BMC Health Services Research* **20**, 67 (2020).
2. Alaboud, K. *et al.* Clinicians' Perspectives in Using Patient-Generated Health Data to Improve Ischemic Heart Disease Management. *AMIA Jt Summits Transl Sci Proc* **2022**, 112–119 (2022).
3. Alarabyat, I. A. *et al.* Perceived barriers to effective use of telehealth in managing the care of patients with cardiovascular diseases: a qualitative study exploring healthcare professionals' views in Jordan. *BMC Health Services Research* **23**, 452 (2023).
4. Alessa, T., Hawley, M. & Witte, L. de. Identification of the Most Suitable App to Support the Self-Management of Hypertension: Systematic Selection Approach and Qualitative Study. *JMIR mHealth and uHealth* **9**, e29207 (2021).
5. Al Hemyari, B., Coffey, N., Inman, Z. W. & Singh, A. Healthcare Providers' Experience Using Telehealth During the Onset of the COVID-19 Pandemic in a Predominantly Rural Patient Base: An Online Survey. *Cureus* **15**, e36260 (2023).
6. Andersson, U., Bengtsson, U., Ranerup, A., Midlöv, P. & Kjellgren, K. Patients and Professionals as Partners in Hypertension Care: Qualitative Substudy of a Randomized Controlled Trial Using an Interactive Web-Based System Via Mobile Phone. *Journal of Medical Internet Research* **23**, e26143 (2021).
7. Aquino, M. *et al.* Patients' and Providers' Perspectives on and Needs of Telemonitoring to Support Clinical Management and Self-care of People at High Risk for Preeclampsia: Qualitative Study. *JMIR Human Factors* **9**, e32545 (2022).
8. Artanian, V., Ware, P., Rac, V. E., Ross, H. J. & Seto, E. Experiences and Perceptions of Patients and Providers Participating in Remote Titration of Heart Failure Medication Facilitated by Telemonitoring: Qualitative Study. *JMIR Cardio* **5**, e28259 (2021).
9. Auton, A. *et al.* Smartphone-Based Remote Monitoring for Chronic Heart Failure: Mixed Methods Analysis of User Experience From Patient and Nurse Perspectives. *JMIR Nursing* **6**, e44630 (2023).

10. Aw, M. *et al.* Critical appraisal of a mHealth-assisted community-based cardiovascular disease risk screening program in rural Kenya: an operational research study. *Pathogens and Global Health* (2020).

11. Baratta, J. *et al.* Patient and Health Professional Perceptions of Telemonitoring for Hypertension Management: Qualitative Study. *JMIR Formative Research* **6**, e32874 (2022).

12. Bazzano, A. N., Patel, T., Nauman, E., Cernigliaro, D. & Shi, L. Optimizing Telehealth for Diabetes Management in the Deep South of the United States: Qualitative Study of Barriers and Facilitators on the Patient and Clinician Journey. *Journal of Medical Internet Research* **26**, e43583 (2024).

13. Bergstedt, A. *et al.* Evaluation of a Clinical Decision Support Tool to Guide Adoption of the American Heart Association Telemetry Monitoring Practice Standards. *J Am Heart Assoc* **13**, e031523 (2024).

14. Bernardi, R. & Exworthy, M. Clinical managers' identity at the crossroad of multiple institutional logics in it innovation: The case study of a health care organization in England. *Information Systems Journal* **30**, 566–595 (2020).

15. Bhandari, B. *et al.* Acceptability of a mHealth strategy for hypertension management in a low-income and middle-income country setting: a formative qualitative study among patients and healthcare providers. (2021) doi:10.1136/bmjopen-2021-052986.

16. Boriani, G. *et al.* Consumer-led screening for atrial fibrillation using consumer-facing wearables, devices and apps: A survey of health care professionals by AF-SCREEN international collaboration. *Eur J Intern Med* **82**, 97–104 (2020).

17. Bouchard, K. *et al.* Cardiology professionals' views of social robots in augmenting heart failure patient care. *European Heart Journal - Digital Health* **5**, 69–76 (2024).

18. Breil, B., Salewski, C. & Apolinário-Hagen, J. Comparing the Acceptance of Mobile Hypertension Apps for Disease Management Among Patients Versus Clinical Use Among Physicians: Cross-sectional Survey. *JMIR Cardio* **6**, e31617 (2022).

19. Brouns, B. *et al.* What works and why in the implementation of eRehabilitation after stroke – a process evaluation. *Disability and Rehabilitation: Assistive Technology* **19**, 345–359 (2024).

20. Brown, J. D. *et al.* Description, utilisation and results from a telehealth primary care weight management intervention for adults with obesity in South Carolina. *J Telemed Telecare* **26**, 28–35 (2020).

21. Brown-Johnson, C. G. *et al.* Implementation outcomes of Humanwide: integrated precision health in team-based family practice primary care. *BMC Fam Pract* **22**, 28 (2021).

22. Buis, L. R. *et al.* Understanding the Feasibility, Acceptability, and Efficacy of a Clinical Pharmacist-led Mobile Approach (BPTrack) to Hypertension Management: Mixed Methods Pilot Study. *Journal of Medical Internet Research* **22**, e19882 (2020).

23. Koçyigit Burunkaya, D. *et al.* Perspectives on the Use of Digital Health Technologies in Cardiology Among Specialists from an ESC Member Country: Results from a Survey. *Turk Kardiyol Dern Ars* **52**, 44–51 (2024).

24. Cao, W., Li, L., Mathur, P., Thompson, J. & Milks, M. W. A mobile health application for patients eligible for statin therapy: app development and qualitative feedback on design and usability. *BMC Med Inform Decis Mak* **23**, 128 (2023).

25. Cartledge, S. *et al.* Telehealth is here to stay but not without challenges: a consultation of cardiac rehabilitation clinicians during COVID-19 in Victoria, Australia. *Eur J Cardiovasc Nurs* **21**, 548–558 (2022).

26. Cher, B. P. *et al.* Understanding the Attitudes of Clinicians and Patients Toward a Self-Management eHealth Tool for Atrial Fibrillation: Qualitative Study. *JMIR Hum Factors* **7**, e15492 (2020).

27. Chew, E. *et al.* Trust and Uncertainty in the Implementation of a Pilot Remote Blood Pressure Monitoring Program in Primary Care: Qualitative Study of Patient and Health Care Professional Views. *JMIR Hum Factors* **10**, e36072 (2023).

28. Cornell, S. *et al.* Implementing patient decision aids into general practice clinical decision support systems: Feasibility study in cardiovascular disease prevention. *PEC Innov* **2**, 100140 (2023).

29. Coylewright, M. *et al.* Pragmatic Study of Clinician Use of a Personalized Patient Decision Aid Integrated into the Electronic Health Record: An 8-Year Experience. *Telemed J E Health* **26**, 597–602 (2020).

30. Danielsbacka, J., Feldthusen, C. & Bäck, M. Healthcare professionals' perceptions of using a digital patient educational programme as part of cardiac rehabilitation in patients with coronary artery disease - a qualitative study. *BMC Health Serv Res* **23**, 1017 (2023).

31. Demers, S. & Wagner, J. M. Nurses' and Physicians' Perspectives on Text-Based Postpartum Blood Pressure Monitoring. *The Journal for Nurse Practitioners* **17**, 348–353 (2021).

32. Ding, E. Y. *et al.* Survey of current perspectives on consumer-available digital health devices for detecting atrial fibrillation. *Cardiovasc Digit Health J* **1**, 21–29 (2020).

33. Dullabh, P. *et al.* Implementation and evaluation of an electronic health record-integrated app for postpartum monitoring of hypertensive disorders of pregnancy using patient-contributed data collection. *JAMIA Open* **6**, ooad098 (2023).

34. Einhorn, J. *et al.* Automated Messaging Program to Facilitate Systematic Home Blood Pressure Monitoring: Qualitative Analysis of Provider Interviews. *JMIR Cardio* **7**, e51316 (2023).

35. Ferguson, C. *et al.* Clinician Perspectives on the Design and Application of Wearable Cardiac Technologies for Older Adults: Qualitative Study. *JMIR Aging* **3**, e17299 (2020).

36. Flores-Sandoval, C. *et al.* Virtual care during COVID-19: The perspectives of older adults and their healthcare providers in a cardiac rehabilitation setting. *Can J Aging* **43**, 491–498 (2024).

37. Frith, G. *et al.* Changes in patient activation following cardiac rehabilitation using the Active+me digital healthcare platform during the COVID-19 pandemic: a cohort evaluation. *BMC Health Services Research* **21**, 1363 (2021).

38. Gago, C. *et al.* 'Hypertension is such a difficult disease to manage': federally qualified health center staff- and leadership-perceived readiness to implement a technology-facilitated team-based hypertension model. *Implement Sci Commun* **5**, 49 (2024).

39. Bezerra Giordan, L., Ronto, R., Chau, J., Chow, C. & Laranjo, L. Use of Mobile Apps in Heart Failure Self-management: Qualitative Study Exploring the Patient and Primary Care Clinician Perspective. *JMIR Cardio* **6**, e33992 (2022).

40. Gomis-Pastor, M. *et al.* Interdisciplinary Mobile Health Model to Improve Clinical Care After Heart Transplantation: Implementation Strategy Study. *JMIR Cardio* **4**, e19065 (2020).

41. Gondi, S. *et al.* Physician perceived barriers and facilitators for self-measured blood pressure monitoring- a qualitative study. *PLoS One* **16**, e0255578 (2021).

42. Gordon, K. *et al.* Normalizing Telemonitoring in Nurse-Led Care Models for Complex Chronic Patient Populations: Case Study. *JMIR Nurs* **5**, e36346 (2022).

43. Gregory, M. E. *et al.* Exploring the Incorporation of a Novel Cardiotoxicity Mobile Health App Into Care of Patients With Cancer: Qualitative Study of Patient and Provider Perspectives. *JMIR Cancer* **9**, e46481 (2023).

44. Grey, E. *et al.* Patient-clinician dynamics in remote consultations: a qualitative study of cardiology and rheumatology outpatient clinics in the UK. *BMJ Open* **13**, e070923 (2023).

45. Gupta, A. *et al.* Implementing a home-based virtual hypertension programme-a pilot feasibility study. *Fam Pract* **40**, 414–422 (2023).

46. Hammersley, V. *et al.* Telemonitoring at scale for hypertension in primary care: An implementation study. *PLOS Medicine* **17**, e1003124 (2020).

47. Harris, E. *et al.* Development and user-testing of a digital patient decision aid to facilitate shared decision-making for people with stable angina. *BMC Med Inform Decis Mak* **22**, 143 (2022).

48. Harvey, M. & Seiler, A. Challenges in managing a remote monitoring device clinic. *Heart Rhythm O2* **3**, 3–7 (2022).

49. Obaid Hassan, A. *et al.* Screening of atrial fibrillation in dental practices: a qualitative feasibility study. *Expert Rev Cardiovasc Ther* **21**, 643–649 (2023).

50. Hauschildt, J. *et al.* Adoption of shared decision-making and clinical decision support for reducing cardiovascular disease risk in community health centers. *JAMIA Open* **6**, ooad012 (2023).

51. Hellem, A. *et al.* Determinants of Bluetooth-Enabled Self-Measured Blood Pressure Monitoring in Federally Qualified Health Centers. *J Prim Care Community Health* **15**, 21501319241229921 (2024).

52. Honein-AbouHaidar, G. N., Antoun, J., Badr, K., Hlais, S. & Nazaretian, H. Users' acceptance of electronic patient portals in Lebanon. *BMC Med Inform Decis Mak* **20**, 31 (2020).

53. Houle, J. *et al.* Acceptability of a computer-tailored and pedometer-based socio-cognitive intervention in a secondary coronary heart disease prevention program: A qualitative study. *Digit Health* **6**, 2055207619899840 (2020).

54. Indraratna, P. *et al.* Process Evaluation of a Randomised Controlled Trial for TeleClinical Care, a Smartphone-App Based Model of Care. *Front Med (Lausanne)* **8**, 780882 (2021).

55. Islam, S. M. S. *et al.* Healthcare providers' perspectives on using smart home systems to improve self-management and care in people with heart failure: A qualitative study. *Int J Med Inform* **167**, 104837 (2022).

56. Jongsma, K. R., Bekker, M. N., Haitjema, S. & Bredenoord, A. L. How digital health affects the patient-physician relationship: An empirical-ethics study into the perspectives and experiences in obstetric care. *Pregnancy Hypertens* **25**, 81–86 (2021).

57. Kalinina, A. M. *et al.* The attitude of primary care physicians to the use of telemedicine technologies in dispensary observation of patients with chronic diseases (medical and sociological research). *Profil. med.* **23**, 8 (2020).

58. Kalinina, A. M. *et al.* On the topic of demand and usage of the telemedicine model «doctor-patient» for the prevention and control of chronic diseases: the view of a primary health care doctor. *Profil. med.* **24**, 28 (2021).

59. Kauw, D. *et al.* Mobile health in cardiac patients: an overview on experiences and challenges of stakeholders involved in daily use and development. *BMJ Innovations* **6**, (2020).

60. Kerr, B. *et al.* Changing to remote management of a community heart failure population during COVID-19 - Clinician and patient perspectives'. *Int J Cardiol Heart Vasc* **31**, 100665 (2020).

61. Khonsari, S., Chandler, C., Parker, R. & Holloway, A. Increasing cardiovascular medication adherence: A medical research council complex mhealth intervention mixed-methods feasibility study to inform global practice. *J Adv Nurs* **76**, 2670–2684 (2020).

62. Klaiman, T. *et al.* Qualitative analysis of a remote monitoring intervention for managing heart failure. *BMC Cardiovasc Disord* **23**, 440 (2023).

63. Kleiner, D. *et al.* Implementation of a personal health planning program and health promotion in the community pharmacies. *Heliyon* **8**, (2022).

64. Kobe, E. A. *et al.* Implementation of an Intensive Telehealth Intervention for Rural Patients with Clinic-Refractory Diabetes. *J Gen Intern Med* **37**, 3080–3088 (2022).

65. Kocanda, L. *et al.* Informing telehealth service delivery for cardiovascular disease management: exploring the perceptions of rural health professionals. *Aust. Health Review* **45**, 241–246 (2021).

66. Kołtowski, Ł. *et al.* Cardiological teleconsultation in the coronavirus disease 2019 era: patient's and physician's perspective. *Kardiol Pol* **79**, 76–78 (2021).

67. Ladegaard, C. T. *et al.* Reaching Frail Elderly Patients to Optimize Diagnosis and Management of Atrial Fibrillation (REAFEL): A Feasibility Study of a Cross-Sectoral Shared-Care Model. *Int J Environ Res Public Health* **19**, 7383 (2022).

68. Leonard, C. *et al.* Informing Use of Telehealth for Managing Chronic Conditions: Mixed-Methods Evaluation of Telehealth Use to Manage Heart Failure During COVID-19. *J Am Heart Assoc* **12**, e027362 (2023).

69. Lino, I. G. T. *et al.* Nurses' willingness to resort to Telemonitoring in users with chronic conditions: A cross-sectional study. *Online Brazilian Journal of Nursing* **22**, (2023).

70. Lukey, A., Mackay, M., Hasan, K. & Rush, K. L. Clinical Perspectives on the Development of a Gamified Heart Failure Patient Education Web Site. *Comput Inform Nurs* **41**, 615–620 (2023).

71. Lyles, E. *et al.* Adoption of Electronic Medical Records for Chronic Disease Care in Kenyan Refugee Camps: Quantitative and Qualitative Prospective Evaluation. *JMIR Mhealth Uhealth* **11**, e43878 (2023).

72. Mahmoud, Z. *et al.* Facilitators and barriers to optimal home blood pressure management in patients with hypertensive disorders of pregnancy in a tertiary care facility in Abuja, Nigeria: a qualitative research study. *BMC Health Serv Res* **23**, 954 (2023).

73. Maria, A. R. J., Serra, H. & Heleno, B. Teleconsultations and their implications for health care: A qualitative study on patients' and physicians' perceptions. *Int J Med Inform* **162**, 104751 (2022).

74. Marzolini, S., Ghisi, G. L. de M., Hébert, A.-A., Ahden, S. & Oh, P. Cardiac Rehabilitation in Canada During COVID-19. *CJC Open* **3**, 152–158 (2021).

75. Matthiesen, S. *et al.* Clinician Preimplementation Perspectives of a Decision-Support Tool for the Prediction of Cardiac Arrhythmia Based on Machine Learning: Near-Live Feasibility and Qualitative Study. *JMIR Hum Factors* **8**, e26964 (2021).

76. McCarthy, M. M. *et al.* Implementing a Clinical Decision Support Tool to Improve Physical Activity. *Nurs Res* **73**, 216–223 (2024).

77. McGillion, M. *et al.* Postoperative Remote Automated Monitoring and Virtual Hospital-to-Home Care System Following Cardiac and Major Vascular Surgery: User Testing Study. *Journal of Medical Internet Research* **22**, e15548 (2020).

78. Meador, M. *et al.* Self-Measured Blood Pressure Monitoring During the COVID-19 Pandemic: Perspectives From Community Health Center Clinicians. *J Healthc Qual* **46**, 109–118 (2024).

79. Mehta, S., Mehta, N., Tang, W. H. & Young, J. Cardiologists' Perception of Wearable Device Data in Patients with Heart Failure. *J Gen Intern Med* **35**, 940–941 (2020).

80. Morton, K. *et al.* Implementing a digital intervention for managing uncontrolled hypertension in Primary Care: a mixed methods process evaluation. *Implement Sci* **16**, 57 (2021).

81. Muehlensiepen, F. *et al.* Acceptance of Telemedicine by Specialists and General Practitioners in Cardiology Care: Cross-Sectional Survey Study. *JMIR Form Res* **8**, e49526 (2024).

82. Nau, T., Owen, A., Mazza, D. & Smith, B. J. Engaging primary care providers in a mobile health strategy to support lifestyle change and blood pressure management. *Digit Health* **7**, 20552076211066746 (2021).

83. Nayyar, D. *et al.* Quality of virtual care for ambulatory care sensitive conditions: Patient and provider experiences. *Int J Med Inform* **165**, 104812 (2022).

84. Nemis-White, J. M. *et al.* Lessons learned from Integrated Management Program Advancing Community Treatment of Atrial Fibrillation (IMPACT-AF): a pragmatic clinical trial of computerized decision support in primary care. *Trials* **22**, 531 (2021).

85. Otieno, P. *et al.* Perceived health system facilitators and barriers to integrated management of hypertension and type 2 diabetes in Kenya: a qualitative study. *BMJ Open* **13**, e074274 (2023).

86. Palozzi, G., Binci, D. & Schettini, I. Digital Transformation in the Healthcare Sector: Empirical Evidences of IoHT Benefits and Limits on Chronic Disease Management. in *Mobility Internet of Things 2018* (eds. Cagáňová, D. & Horňáková, N.) 433–449 (Springer International Publishing, Cham, 2020). doi:10.1007/978-3-030-30911-4_31.

87. Pelly, M. L., Fatehi, F., Liew, D. & Verdejo-Garcia, A. Digital Health Secondary Prevention Using Co-Design Procedures: Focus Group Study With Health Care Providers and Patients With Myocardial Infarction. *JMIR Cardio* **7**, e49892 (2023).

88. Piskorz, D. *et al.* Blood pressure telemonitoring and telemedicine for hypertension management-positions, expectations and feasibility of Latin-American practitioners. SURVEY carried out by several cardiology and hypertension societies of the Americas. *Blood Press* **31**, 236–244 (2022).

89. Portz, J. D. *et al.* 'I Like the Idea of It…But Probably Wouldn't Use It' - Health Care Provider Perspectives on Heart Failure mHealth: Qualitative Study. *JMIR Cardio* **4**, e18101 (2020).

90. Queiroz, C. *et al.* Digital health and cardiovascular healthcare professionals in Portugal: Current status, expectations and barriers to implementation. *Rev Port Cardiol* **43**, 459–467 (2024).
91. Rakers, M. *et al.* Tailoring remote patient management in cardiovascular risk management for healthcare professionals using panel management: a qualitative study. *BMC Prim Care* **25**, 122 (2024).
92. Riboli-Sasco, E. *et al.* Investigating barriers & facilitators for the successful implementation of the BP@home initiative in London: Primary care perspectives. *PLoS One* **19**, e0298898 (2024).
93. Rodrigues, T. R. C. *et al.* Facilitators of and Barriers to Lifestyle Support and eHealth Solutions: Interview Study Among Health Care Professionals Working in Cardiac Care. *Journal of Medical Internet Research* **23**, e25646 (2021).
94. Saifan, A. R., Alarabyat, I. A., Alrimawi, I. & Al-Nsair, N. Utilizing telehealth intervention to support patients with cardiovascular diseases in Jordan: A qualitative study. *Appl Nurs Res* **68**, 151641 (2022).
95. Sanderson, K. E., Spithoff, K. D., Corovic, M., Langdon, K. M. & Schwalm, J.-D. An Evaluation of Cardiology Virtual Care During the COVID-19 Pandemic. *CJC Open* **3**, 1294–1299 (2021).
96. Santana, M.-J. *et al.* Patients, clinicians and researchers working together to improve cardiovascular health: a qualitative study of barriers and priorities for patient-oriented research. *BMJ Open* **10**, e031187 (2020).
97. Santos, R. Z. D. *et al.* Feasibility of a Virtual Educational Programme for Behaviour Change in Cardiac Patients from a Low-Resource Setting. *Int J Environ Res Public Health* **20**, 5934 (2023).
98. Savickas, V. *et al.* Pharmacists detecting atrial fibrillation in general practice: a qualitative focus group study. *BJGP Open* **4**, bjgpopen20X101042 (2020).
99. Schepart, A. *et al.* Artificial intelligence-enabled tools in cardiovascular medicine: A survey of current use, perceptions, and challenges. *Cardiovasc Digit Health J* **4**, 101–110 (2023).
100. Schertzer, K. *et al.* Physiotherapists' Adoption and Perceptions of Tele-Rehabilitation for Cardiorespiratory Care in Response to COVID-19. *Physiotherapy Canada* **76**, 211–217 (2024).
101. Schott, S. L. *et al.* Personalized, Electronic Health Record-Integrated Decision Aid for Stroke Prevention in Atrial Fibrillation: A Small Cluster Randomized Trial and Qualitative Analysis of Efficacy and Acceptability. *Circ Cardiovasc Qual Outcomes* **14**, e007329 (2021).

102. Sideris, K. *et al.* Artificial intelligence predictive analytics in heart failure: results of the pilot phase of a pragmatic randomized clinical trial. *J Am Med Inform Assoc* **31**, 919–928 (2024).

103. Singh, K. *et al.* Collaborative Quality Improvement Strategy in Secondary Prevention of Cardiovascular Disease in India: Findings from a Multi-Stakeholder, Qualitative Study using Consolidated Framework for Implementation Research (CFIR). *Glob Heart* **17**, 72 (2022).

104. Singhal, A., Riley, J. P. & Cowie, M. R. Benefits and challenges of telemedicine for heart failure consultations: a qualitative study. *BMC Health Serv Res* **23**, 847 (2023).

105. Sivakumar, B. *et al.* Evaluating Health Care Provider Perspectives on the Use of Mobile Apps to Support Patients With Heart Failure Management: Qualitative Descriptive Study. *JMIR Cardio* **6**, e40546 (2022).

106. Sobreiro, P. & Oliveira, A. The Importance of ICT and Wearable Devices in Monitoring the Health Status of Coronary Patients. in *Human Systems Engineering and Design II* (eds. Ahram, T., Karwowski, W., Pickl, S. & Taiar, R.) 705–711 (Springer International Publishing, Cham, 2020). doi:10.1007/978-3-030-27928-8_107.

107. Steinman, L. *et al.* Can mHealth and eHealth improve management of diabetes and hypertension in a hard-to-reach population? -lessons learned from a process evaluation of digital health to support a peer educator model in Cambodia using the RE-AIM framework. *Mhealth* **6**, 40 (2020).

108. Steinman, L. *et al.* Facilitators and Barriers to Chronic Disease Self-Management and Mobile Health Interventions for People Living With Diabetes and Hypertension in Cambodia: Qualitative Study. *JMIR mHealth and uHealth* **8**, e13536 (2020).

109. Suen, M., Manski-Nankervis, J.-A., McBride, C., Lumsden, N. & Hunter, B. Implementing a Sodium-Glucose Cotransporter 2 Inhibitor Module With a Software Tool (Future Health Today): Qualitative Study. *JMIR Form Res* **8**, e50737 (2024).

110. Teo, S. H. *et al.* Implementation and use of technology-enabled blood pressure monitoring and teleconsultation in Singapore's primary care: a qualitative evaluation using the socio-technical systems approach. *BMC Prim Care* **24**, 71 (2023).

111. Thomas, E. E., Chambers, R., Phillips, S., Rawstorn, J. C. & Cartledge, S. Sustaining telehealth among cardiac and pulmonary rehabilitation services: a qualitative framework study. *Eur J Cardiovasc Nurs* **22**, 795–803 (2023).

112. Thorup, C. B. *et al.* Perspectives on Participation in a Feasibility Study on Exercise-Based Cardiac Telerehabilitation After Transcatheter Aortic Valve Implantation: Qualitative Interview Study Among Patients and Health Professionals. *JMIR Form Res* **6**, e35365 (2022).

113. Tighe, S. A., Ball, K., Kayser, L., Kensing, F. & Maddison, R. Qualitative study of the views of people living with cardiovascular disease, and healthcare professionals, towards the use of a digital platform to support cardiovascular disease self-management. *BMJ Open* **12**, e056768 (2022).

114. Toft, B. S. *et al.* Feasibility of early digital health rehabilitation after cardiac surgery in the elderly: a qualitative study. *BMC Health Serv Res* **24**, 113 (2024).

115. Tsukada, Y. T. *et al.* Status of Medical Care and Management Requirements of Elderly Patients With Heart Failure in a Comprehensive Community Health System - Survey of General Practitioners' Views. *Circ Rep* **3**, 77–85 (2021).

116. Van Emmenis, M. *et al.* Patient and practitioner views on a combined face-to-face and digital intervention to support medication adherence in hypertension: a qualitative study within primary care. *BMJ Open* **12**, e053183 (2022).

117. Vandekerckhove, P., Vandekerckhove, Y., Tavernier, R., De Jaegher, K. & de Mul, M. Leveraging User Experience to Improve Video Consultations in a Cardiology Practice During the COVID-19 Pandemic: Initial Insights. *J Med Internet Res* **22**, e19771 (2020).

118. Varnfield, M. *et al.* M♡THer, an mHealth System to Support Women with Gestational Diabetes Mellitus: Feasibility and Acceptability Study. *Diabetes Technol Ther* **23**, 358–366 (2021).

119. Vugs, S., Scholte, N. T. B., Markopoulos, P. & Ronner, E. Clinician Attitudes Towards Telemonitoring for Heart Failure Care: Opportunities for Design Research. in *Extended Abstracts of the 2023 CHI Conference on Human Factors in Computing Systems* 1–8 (Association for Computing Machinery, New York, NY, USA, 2023). doi:10.1145/3544549.3585723.

120. Waldman, C. E. *et al.* COVID-19 telehealth preparedness: a cross-sectional assessment of cardiology practices in the USA. *Per Med* **19**, 411–422 (2022).

121. Wali, S. *et al.* Expanding Telemonitoring in a Virtual World: A Case Study of the Expansion of a Heart Failure Telemonitoring Program During the COVID-19 Pandemic. *Journal of Medical Internet Research* **23**, e26165 (2021).

122. Wali, S. *et al.* Exploring the use of a digital therapeutic intervention to support the pediatric cardiac care journey: Qualitative study on clinician perspectives. *PLOS Digit Health* **2**, e0000371 (2023).

123. Watson, K. E. *et al.* Managing Medications During 'Sick Days' in Patients With Diabetes, Kidney, and Cardiovascular Conditions: A Theory-informed Approach to Intervention Design and Implementation. *Can J Diabetes* **48**, 259-268.e4 (2024).

124. Yao, Y. *et al.* Factors affecting wearable ECG device adoption by general practitioners for atrial fibrillation screening: cross-sectional study. *Front Public Health* **11**, 1128127 (2023).

125. Zolezzi, M. *et al.* Translating and piloting a cardiovascular risk assessment and management online tool using mobile technology. *Saudi Pharm J* **31**, 492–498 (2023).

Supplemental file 4. Quality appraisal of included studies.

Appraisal questions for qualitative studies

1. Is the research question/objective sufficiently described?
2. Is the research study design evident and appropriate?
3. Is the context for the study clear?
4. Is there a connection to a theoretical framework/wider body of knowledge?
5. Was a sampling strategy described, relevant and justified?
6. Were the data collection methods clearly described and systematic?
7. Was the data analysis clearly described and systematic?
8. Was a verification procedure(s) used to establish credibility?
9. Were the conclusions supported by the results?
10. Was there any reflexivity of the account performed?

First Author	Year	Article DOI	Appraisal question										Score
			1	2	3	4	5	6	7	8	9	10	
Alessa	2021	10.2196/29207	Yes	Yes	Yes	Yes	Partial	Yes	Yes	Yes	Yes	No	0.85
Adler	2020	10.1186/s12913-019-4774-x	Yes	Yes	Yes	No	Yes	Partial	Partial	Yes	Yes	No	0.7
Alaboud	2022	PMID 35854732	Yes	Yes	Partial	No	Partial	Partial	Partial	No	Yes	No	0.5
Alarabyat	2023	10.1186/s12913-023-09368-w.	Yes	Yes	Yes	Partial	Yes	Yes	Yes	Yes	Yes	Partial	0.9
Andersson	2021	10.2196/26143	Yes	Yes	Yes	Yes	Yes	Yes	Yes	Yes	Yes	No	0.9
Aquino	2022	10.2196/32545	Yes	Yes	Yes	Yes	Yes	Yes	Yes	Yes	Yes	Partial	0.95
Artanian	2021	10.2196/28259	Yes	Yes	Yes	Yes	Yes	Yes	Yes	Yes	Yes	No	0.9
Auton	2023	10.2196/44630	Yes	Yes	Yes	Yes	No	Yes	Yes	No	Yes	Yes	0.8
Aw	2020	10.1080/20477724.2020.1816286	Yes	Yes	Yes	No	Partial	No	No	No	Yes	No	0.45
Baratta	2022	10.2196/32874	Yes	Yes	Yes	Partial	Partial	Yes	Partial	Yes	Yes	No	0.75
Bazzano	2024	10.2196/43583	Yes	Yes	Yes	Yes	Partial	Yes	Yes	No	Yes	Yes	0.85
Bergstedt	2024	10.1161/JAHA.123.031523	Yes	Yes	Yes	Yes	Yes	Yes	Yes	Yes	Yes	Partial	0.95

Bernardi	2020	10.1111/isj.12267	Yes	Yes	Yes	Yes	No	Yes	Yes	No	Yes	Yes	0.8
Bhandari	2021	10.1136/bmjopen-2021-052986	Yes	Yes	Yes	Yes	Yes	Yes	Yes	Yes	Yes	Partial	0.95
Bouchard	2024	10.1093/ehjdh/ztad067	Yes	Yes	Yes	Yes	Yes	Yes	Yes	No	Yes	Yes	0.9
Brouns	2024	10.1080/17483107.2022.2088867	Yes	Yes	Yes	Yes	No	Yes	Yes	No	Yes	Partial	0.75
Brown	2020	10.1177/1357633X18789562	Yes	Yes	Yes	Yes	Partial	Partial	No	No	Partial	No	0.55
Brown-Johnson	2021	10.1186/s12875-021-01373-4	Yes	Yes	Yes	Yes	Yes	Yes	Yes	Yes	Yes	Yes	1
Buis	2020	10.2196/19882	Yes	Yes	Yes	Partial	Yes	Partial	Partial	No	Yes	No	0.65
Cao	2023	10.1186/s12911-023-02221-4	Yes	Yes	Yes	Yes	No	Yes	Yes	No	Yes	Partial	0.75
Cartledge	2022	10.1093/eurjcn/zvab118	Yes	Yes	Yes	Yes	Partial	Yes	Yes	Yes	Yes	Yes	0.95
Cher	2020	10.2196/15492	Yes	Yes	Yes	Yes	Yes	Yes	Yes	No	Yes	No	0.8
Chew	2023	10.2196/36072	Yes	Yes	Yes	Partial	Partial	Yes	Yes	No	Yes	Partial	0.75
Cornell	2023	10.1016/j.pecinn.2023.100140	Yes	Yes	Yes	No	Partial	Yes	Partial	No	Partial	Partial	0.6
Coylewright	2020	10.1089/tmj.2019.0112	Partial	Yes	Yes	No	Yes	No	Partial	No	Yes	No	0.5
Danielsbacka	2023	10.1186/s12913-023-09997-1	Yes	Yes	Yes	Yes	Yes	Yes	Yes	Yes	Yes	Partial	0.95
Demers	2021	10.1016/j.nurpra.2020.09.004	Yes	Partial	Partial	No	Partial	Partial	Partial	No	Yes	No	0.45
Dullabh	2023	10.1093/jamiaopen/ooad098.	Partial	Yes	Yes	Yes	No	Yes	Yes	No	Partial	Partial	0.65
Einhorn	2023	10.2196/51316	Yes	Yes	Yes	Yes	Partial	Yes	Yes	No	Yes	Partial	0.8
Ferguson	2020	10.2196/17299	Yes	Yes	Yes	No	Partial	Yes	Partial	Yes	Yes	No	0.7
Flores-Sandoval	2024	10.1017/S0714980824000102	Yes	Yes	Yes	Yes	Partial	Yes	Yes	No	Yes	Partial	0.8
Frith	2021	https://doi.org/10.1186/s12913-021-07363-7	Yes	Yes	Yes	Partial	Partial	Yes	Yes	Yes	Yes	No	0.8
Gago	2024	10.1186/s43058-024-00587-8	Yes	Yes	Yes	Yes	Partial	Yes	Yes	No	Yes	Partial	0.8
Giordan	2022	10.2196/33992	Yes	Yes	Yes	Yes	Partial	Yes	Yes	Partial	Yes	Partial	0.85
Gomis-Pastor	2020	10.2196/19065	Yes	Yes	Yes	No	Partial	Partial	Partial	No	Yes	No	0.55
Gondi	2021	10.1371/journal.pone.0255578	Yes	Yes	Yes	Yes	Yes	Yes	Yes	Yes	Yes	Partial	0.95

Gordon	2022	10.2196/36346	Yes	Yes	Yes	Yes	Yes	Yes	Yes	Yes	Yes	Partial	0.95
Gregory	2023	10.2196/46481	Yes	Yes	Yes	Yes	Partial	Yes	Yes	No	Yes	Partial	0.8
Grey	2023	10.1136/bmjopen-2022-070923	Yes	Yes	Yes	No	Partial	Yes	Yes	No	Yes	Yes	0.75
Gupta	2023	10.1093/fampra/cmac084.	Yes	Yes	Yes	No	No	Yes	Yes	No	Yes	No	0.6
Hammersley	2020	10.1371/journal.pmed.1003124	Yes	Yes	Yes	Partial	Partial	Yes	Partial	Partial	Yes	No	0.7
Harris	2022	10.1186/s12911-022-01882-x	Yes	Yes	Yes	Yes	Yes	Yes	Yes	Yes	Yes	Partial	0.95
Harvey	2022	10.1016/j.hroo.2021.12.002	Yes	Yes	Partial	Partial	Partial	Yes	Partial	No	Partial	No	0.55
Hassan	2023	10.1080/14779072.2023.2254679.	Yes	Yes	Yes	Yes	Yes	Yes	Yes	Yes	Yes	No	0.9
Hauschildt	2023	10.1093/jamiaopen/ooad012	Yes	Yes	Yes	Yes	Partial	Yes	Yes	No	Yes	No	0.75
Hellem	2024	10.1101/2023.08.17.23294249	Yes	Yes	Yes	Yes	Partial	Yes	Yes	No	Partial	Partial	0.75
Houle	2020	10.1177/2055207619899840	Yes	Yes	Yes	Yes	Yes	Yes	Yes	No	Yes	No	0.8
Indraratna	2022	10.3389/fmed.2021.780882	Yes	Yes	Yes	Yes	Yes	Yes	Partial	No	Yes	No	0.75
Islam	2022	10.1016/j.ijmedinf.2022.104837.	Yes	Yes	Partial	Partial	Partial	Yes	Yes	Yes	Yes	No	0.75
Jongsma	2021	10.1016/j.preghy.2021.05.017	Yes	Partial	Yes	No	Partial	Partial	Yes	Partial	Yes	No	0.6
Kauw	2020	10.1136/bmjinnov-2019-000418	Yes	Yes	Partial	No	Partial	Yes	Yes	No	Yes	No	0.6
Khonsari	2020	10.1111/jan.14465	Yes	Yes	Yes	Yes	Partial	Yes	Yes	Yes	Yes	Partial	0.9
Klaiman	2023	10.1186/s12872-023-03456-9	Yes	Yes	Yes	Partial	Yes	Yes	Yes	Yes	Yes	No	0.85
Kobe	2022	10.1007/s11606-021-07281-8	Partial	Yes	Yes	Yes	Partial	Partial	Yes	Yes	Partial	No	0.7
Kocanda	2021	doi.org/10.1071/AH19231	Yes	Yes	Yes	Yes	Yes	Yes	Yes	Yes	Yes	No	0.9
Kołtowski	2021	10.33963/KP.15737	Partial	Partial	Yes	No	Partial	Partial	No	No	Yes	No	0.4
Ladegaard	2022	10.3390/ijerph19127383	Yes	Yes	Yes	Yes	Partial	No	Yes	Yes	Partial	No	0.7
Leonard	2023	10.1161/JAHA.122.027362	Yes	Yes	Yes	Partial	Yes	Yes	Yes	Yes	Yes	No	0.85
Lukey	2023	10.1097/CIN.0000000000000983	Yes	Partial	Partial	Partial	Partial	Yes	Yes	Yes	Yes	No	0.7
Lyles	2023	10.2196/43878	Yes	Yes	Yes	Yes	Yes	Partial	Partial	No	Yes	Yes	0.8
Mahmoud	2023	10.1186/s12913-023-09976-6	Yes	Yes	Yes	Yes	Yes	No	Yes	No	Yes	No	0.7
Maria	2022	10.1016/j.ijmedinf.2022.104751	Yes	Yes	Yes	No	Partial	No	Yes	Yes	Yes	No	0.65
Marzolini	2021	10.1016/j.cjco.2020.09.021	Yes	Yes	Yes	Partial	Partial	Yes	No	No	Yes	No	0.6
Matthiesen	2021	10.2196/26964	Yes	Yes	Yes	Partial	Partial	Yes	Yes	Yes	Yes	No	0.8

McCarthy	2024	10.1097/NNR.0000000000000714	Yes	Yes	Yes	Yes	No	Partial	Partial	No	Yes	No	0.6
McGillion	2020	10.2196/15548	Yes	Yes	Yes	Yes	Yes	Yes	Yes	Partial	Yes	Partial	0.9
Meador	2024	10.1097/JHQ.0000000000000417	Yes	Yes	Yes	No	No	Yes	Yes	No	Yes	Partial	0.65
Mehta	2020	10.1007/s11606-019-05390-z	Yes	Yes	Partial	No	Yes	Partial	Yes	No	Yes	No	0.6
Morton	2021	10.1186/s13012-021-01123-1	Yes	Yes	Yes	Yes	Partial	Yes	Yes	Yes	Yes	Partial	0.9
Nau	2021	10.1177/20552076211066746	Yes	Yes	Yes	Partial	Partial	Yes	Partial	No	Yes	No	0.65
Nayyar	2022	10.1016/j.ijmedinf.2022.104812	Yes	Yes	Yes	Yes	Partial	No	Yes	Yes	Yes	No	0.75
Nemis-White	2021	10.1186/s13063-021-05488-y	Yes	Yes	Yes	No	No	Partial	No	No	Yes	No	0.45
Otieno	2023	10.1136/bmjopen-2023-074274	Yes	Yes	Yes	Yes	Yes	Yes	Yes	Partial	Yes	No	0.85
Palozzi	2020	10.1007/978-3-030-30911-4_31	Yes	Yes	Yes	Yes	Partial	Partial	Partial	No	Yes	No	0.65
Pelly	2023	10.2196/49892	Yes	Yes	Yes	Yes	Partial	Yes	Yes	Partial	Yes	Partial	0.85
Portz	2020	10.2196/18101	Yes	Yes	Yes	Yes	Partial	Partial	Yes	Yes	Yes	Partial	0.85
Rakers	2024	10.1186/s12875-024-02355-y	Yes	Yes	Yes	Yes	Yes	Yes	Yes	No	Yes	Partial	0.85
Riboli-Sasco	2024	10.1371/journal.pone.0298898	Yes	Yes	Yes	Yes	Partial	Yes	Yes	No	Yes	Partial	0.8
Rodrigues	2021	10.2196/25646	Yes	Yes	Yes	Partial	Partial	Yes	Yes	Yes	Yes	No	0.8
Saifan	2022	10.1016/j.apnr.2022.151641	Yes	Yes	Yes	Partial	Partial	Yes	Yes	Yes	Yes	No	0.8
Sanderson	2021	10.1016/j.cjco.2021.05.015	Yes	Yes	Yes	Yes	Partial	Partial	Partial	No	Yes	No	0.65
Santana	2020	10.1136/bmjopen-2019-031187	Yes	Yes	Yes	Yes	Yes	Yes	Yes	Yes	Yes	No	0.9
Santos	2023	10.3390/ijerph20115934	Yes	Yes	Yes	Partial	Yes	Partial	Partial	No	Yes	No	0.65
Savickas	2020	10.3399/bjgpopen20X101042	Yes	Yes	Yes	Yes	Yes	No	Yes	Yes	Yes	Partial	0.85
Schepart	2023	10.1016/j.cvdhj.2023.04.003.	Yes	Yes	Partial	Partial	Partial	Yes	Yes	Yes	Yes	Partial	0.8
Schertzer	2024	10.3138/ptc-2021-0135	Yes	Yes	Yes	Partial	Partial	Yes	Yes	Partial	Yes	No	0.75
Schott	2021	10.1161/CIRCOUTCOMES.120.007329	Yes	Yes	Yes	Partial	Partial	Partial	Yes	Yes	Yes	No	0.75
Sideris	2024	10.1093/jamia/ocae017	Yes	Yes	Yes	Yes	No	Yes	Yes	No	Yes	Partial	0.75
Singh	2022	10.5334/gh.1161	Yes	Yes	Yes	Yes	Yes	Yes	Yes	Yes	Yes	No	0.9
Singhal	2023	10.1186/s12913-023-09872-z.	Yes	Yes	Yes	Partial	Yes	Yes	Yes	Partial	Yes	No	0.8
Sivakumar	2022	10.2196/40546	Yes	Yes	Yes	Yes	Yes	Yes	Yes	Yes	Partial	Partial	0.9

Sobreiro	2020	10.1007/978-3-030-27928-8_107	Yes	Yes	Partial	No	No	Partial	Partial	No	Yes	No	0.45
Steinman	2020	10.21037/mhealth-19-249	Yes	Yes	Yes	Yes	Yes	Yes	Yes	Yes	Yes	No	0.9
Steinman	2020	10.2196/13536	Yes	Yes	Yes	Yes	Yes	No	Yes	Partial	Yes	No	0.75
Suen	2024	10.2196/50737	Yes	Yes	Yes	Yes	No	Yes	Yes	No	Yes	Partial	0.75
Teo	2023	10.1186/s12875-023-02014-8	Yes	Yes	Yes	Yes	Yes	Yes	Yes	Partial	Yes	No	0.85
Thomas	2023	10.1093/eurjcn/zvac111	Yes	Yes	Yes	Yes	Yes	Yes	Yes	No	Yes	No	0.8
Thorup	2022	10.2196/35365	Yes	Yes	Yes	Partial	Yes	No	Yes	Partial	Yes	No	0.7
Tighe	2022	10.1136/bmjopen-2021-056768	Yes	Yes	Yes	Yes	Partial	Yes	Yes	Partial	Yes	No	0.8
Toft	2024	10.1186/s12913-024-10601-3	Yes	Yes	Yes	Yes	Yes	Yes	Yes	No	Yes	Yes	0.9
Tsukada	2021	10.1253/circrep.CR-20-0132	Yes	Yes	Yes	Partial	Partial	Yes	Yes	Partial	Yes	No	0.75
Van Emmenis	2022	10.1136/bmjopen-2021-053183	Yes	Yes	Yes	Yes	Partial	Yes	Yes	Yes	Yes	No	0.85
Vandekerckhove	2020	10.2196/19771	Partial	Partial	Yes	Yes	Partial	No	No	No	Yes	No	0.45
Varnfield	2021	10.1089/dia.2020.0509	Yes	Yes	Yes	No	No	No	No	No	Yes	No	0.4
Vugs	2023	10.1145/3544549.3585723	Yes	Yes	Yes	No	Partial	Partial	Yes	Partial	Yes	No	0.65
Waldman	2022	10.2217/pme-2021-0179	Yes	Yes	Yes	No	Yes	Partial	Partial	No	Yes	No	0.6
Wali	2021	10.2196/26165	Yes	Yes	Yes	Yes	Yes	Yes	Yes	Yes	Yes	Partial	0.95
Wali	2023	10.1371/journal.pdig.0000371	Yes	Yes	Yes	Yes	Yes	Partial	Yes	No	Yes	No	0.75
Watson	2024	10.1016/j.jcjd.2024.02.003	Yes	Yes	Yes	Yes	Yes	Yes	Yes	No	Yes	Partial	0.85
Zolezzi	2023	10.1016/j.jsps.2023.02.001	Yes	Yes	Yes	Yes	Yes	Yes	Yes	Yes	Yes	No	0.9

Appraisal questions for quantitative studies

1. Is the research question/objective sufficiently described?
2. Is the research study design evident and appropriate?
3. Is the method of subject/comparison group selection or source of information/input variables described and appropriate?
4. Are the subject (and comparison group, if applicable) characteristics sufficiently described?
5. If interventional and random allocation was possible, was it described?
6. If interventional and blinding of investigators was possible, was it reported?
7. If interventional and blinding of subjects was possible, was it reported?
8. Outcome and (if applicable) exposure measure(s) well defined and robust to measurement / misclassifi cation bias? Means of assessment reported?
9. Sample size appropriate?
10. Analytic methods described/justified and appropriate?
11. Some estimate of variance is reported for the main results?
12. Controlled for confounding?
13. Results reported in sufficient detail?
14. Conclusions supported by the results?

First Author	Year	Article DOI	Appraisal question														Score
			1	2	3	4	5	6	7	8	9	10	11	12	13	14	
Al Hemyari	2023	10.7759/cureus.36260	Yes	Yes	Partial	Yes	N/A	N/A	N/A	Yes	Yes	Yes	Yes	N/A	Yes	Yes	0.95
Boriani	2020	10.1016/j.ejim.2020.09.005	Yes	Yes	Yes	Yes	N/A	N/A	N/A	Partial	Yes	Yes	N/A	N/A	Yes	Yes	0.94
Breil	2022	10.2196/31617	Yes	Yes	Yes	Yes	N/A	N/A	N/A	Yes	Partial	Yes	Yes	Yes	Yes	Yes	0.95
Burunkaya	2024	10.5543/tkda.2023.13660	Yes	Yes	Yes	Yes	N/A	N/A	N/A	No	Yes	Yes	N/A	N/A	Yes	Yes	0.89
Coylewright	2020	10.1089/tmj.2019.0112	Partial	Yes	Yes	Partial	N/A	N/A	N/A	No	Partial	Yes	N/A	N/A	Partial	Yes	0.67
Ding	2020	10.1016/j.cvdhj.2020.06.002	Yes	Yes	Yes	Yes	N/A	N/A	No	Yes	Yes	Yes	Yes	N/A	Yes	Yes	0.91
Gordon	2022	10.2196/36346	Partial	Yes	Partial	Partial	N/A	N/A	N/A	Partial	N/A	Yes	No	N/A	Partial	Partial	0.56
Harvey	2022	10.1016/j.hroo.2021.12.002	Yes	Yes	Yes	Yes	N/A	N/A	N/A	Yes	Partial	Yes	N/A	N/A	Yes	Yes	0.94
Honein-AbouHaidar	2020	10.1186/s12911-020-1047-x	Yes	Yes	Yes	Yes	N/A	N/A	N/A	Yes	Partial	Yes	N/A	N/A	Yes	Yes	0.94
Kalinina 2020	2020	10.17116/profmed202023062 8	Yes	Yes	Yes	Partial	N/A	N/A	N/A	Partial	Partial	Yes	N/A	Partial	Yes	Yes	0.80
Kalinina 2021	2021	10.17116/profmed202124061 28	Yes	Yes	Partial	Yes	N/A	N/A	N/A	Partial	Partial	Yes	N/A	N/A	Yes	Yes	0.83

Kerr	2020	10.1016/j.ijcha.2020.100665	Yes	Partial	Yes	No	N/A	N/A	N/A	No	No	No	N/A	N/A	Partial	Yes	0.44
Kleiner	2022	10.1016/j.heliyon.2022.e09900	Partial	Yes	Yes	No	N/A	N/A	N/A	Yes	Partial	Partial	N/A	N/A	No	Partial	0.56
Lino	2023	10.17665/1676-4285.20236648	Yes	Yes	Partial	Yes	N/A	N/A	N/A	No	Partial	Partial	Yes	N/A	Partial	Yes	0.70
Meador	2024	10.1097/JHQ.0000000000000417	Yes	No	No	Partial	N/A	N/A	N/A	Yes	N/A	Yes	No	N/A	Yes	Yes	0.61
Morton	2021	10.1186/s13012-021-01123-1	Yes	Yes	Yes	Partial	N/A	N/A	N/A	Yes	Yes	Yes	Yes	N/A	Yes	Yes	0.95
Muehlensiepen	2024	10.2196/49526	Yes	Yes	Partial	Yes	N/A	N/A	N/A	Yes	N/A	Yes	No	N/A	Yes	Yes	0.83
Nayyar	2022	10.2196/35599	Yes	Yes	Partial	Yes	N/A	N/A	N/A	No	No	Yes	N/A	N/A	Yes	Yes	0.72
Piskorz	2022	10.1080/08037051.2022.2123781	Yes	Yes	Yes	Yes	N/A	N/A	N/A	Yes	Yes	Yes	Yes	Yes	Partial	Yes	0.95
Queiroz	2024	10.1016/j.repc.2023.10.014	Yes	Yes	No	Yes	N/A	N/A	N/A	Yes	N/A	Yes	No	N/A	Yes	Yes	0.78
Sanderson	2021	10.1016/j.cjco.2021.05.015	Yes	Partial	Partial	No	N/A	N/A	N/A	Yes	Yes	Partial	No	No	Partial	Partial	0.50
Schepart	2023	10.1016/j.cvdhj.2023.04.003	Yes	Yes	Yes	Partial	N/A	N/A	N/A	Partial	N/A	Yes	No	N/A	Yes	Yes	0.78
Waldman	2022	10.2217/pme-2021-0179	Yes	Yes	Partial	Yes	N/A	N/A	N/A	Partial	N/A	Yes	No	N/A	Partial	Yes	0.72
Yao	2023	10.3389/fpubh.2023.1128127	Partial	Yes	Yes	Yes	N/A	N/A	N/A	Partial	Yes	Yes	Yes	N/A	Yes	Yes	0.90

Supplemental file 5. Factors influencing digital health intervention (DHI) uptake by health care providers (HCPs) in relation to four domains: HCPs, patients, technology and the health system.

Numbers represent the number of times that the factor was cited, either as a facilitator (green) or as a barrier (red). Studies from high income countries are represented in dark green and red, and studies from middle income countries in light green and red.

Figure S5.1. Factors relating to HCPs

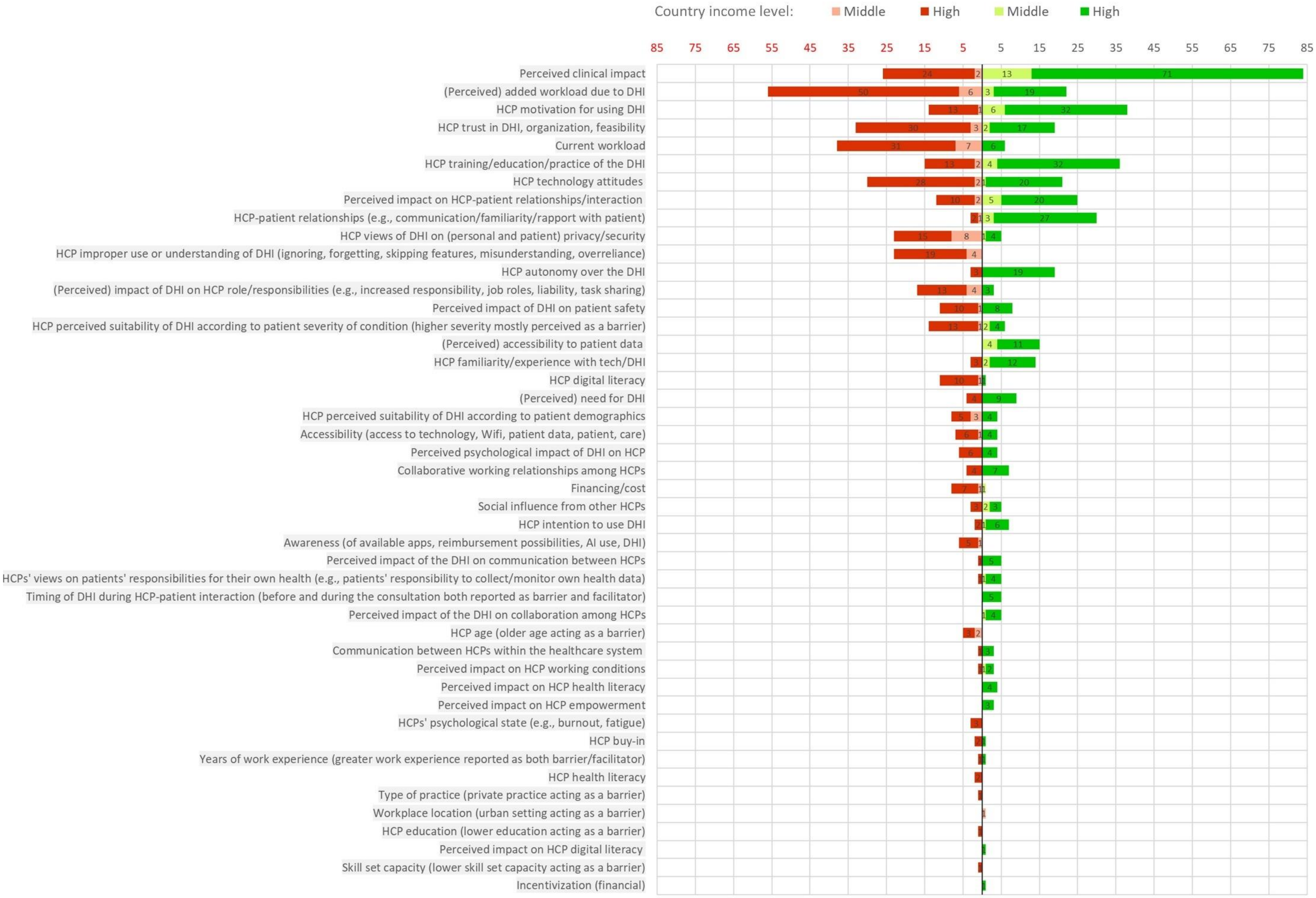

Figure S5.2. Factors relating to patients

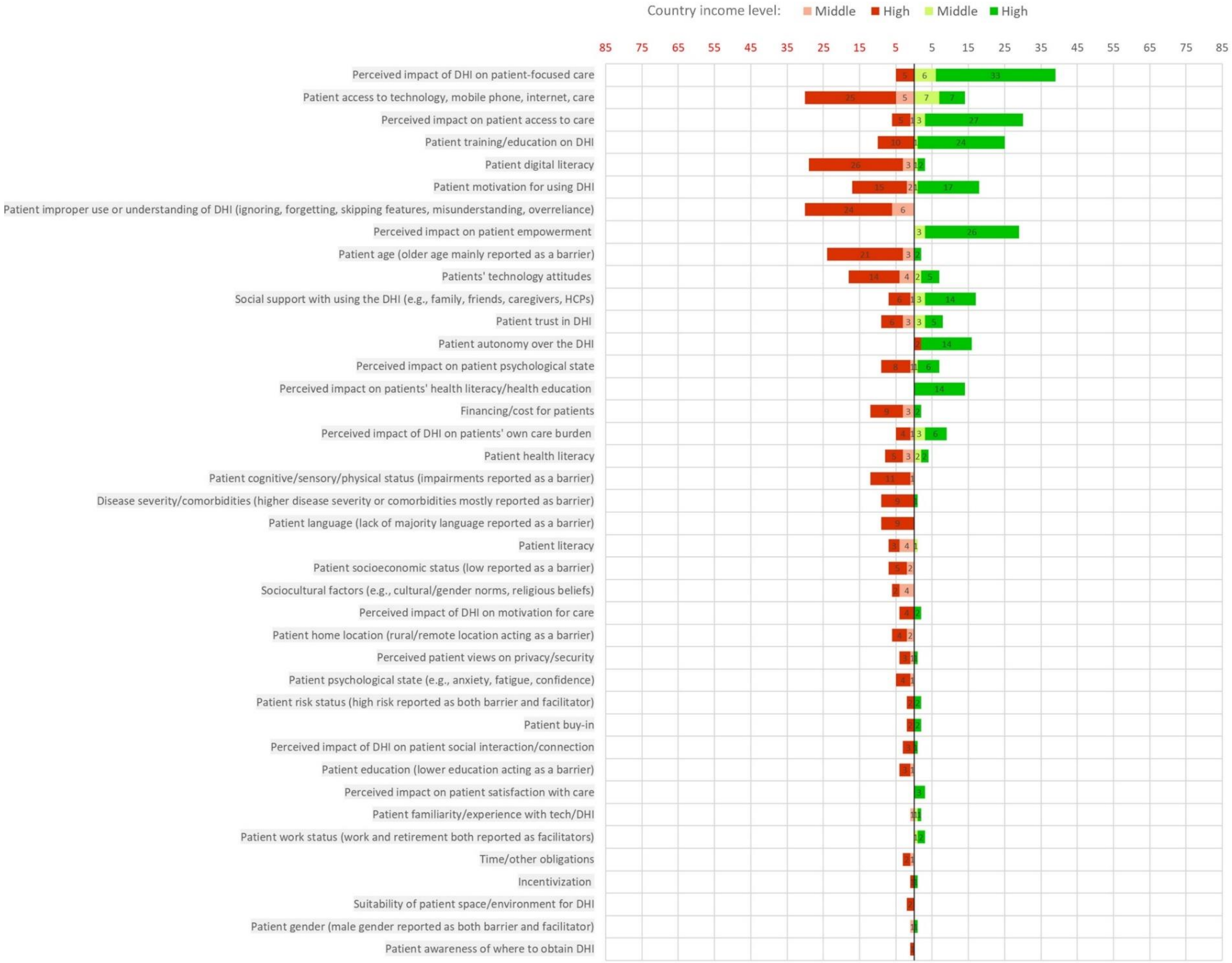

Figure S5.3. Factors relating to technology

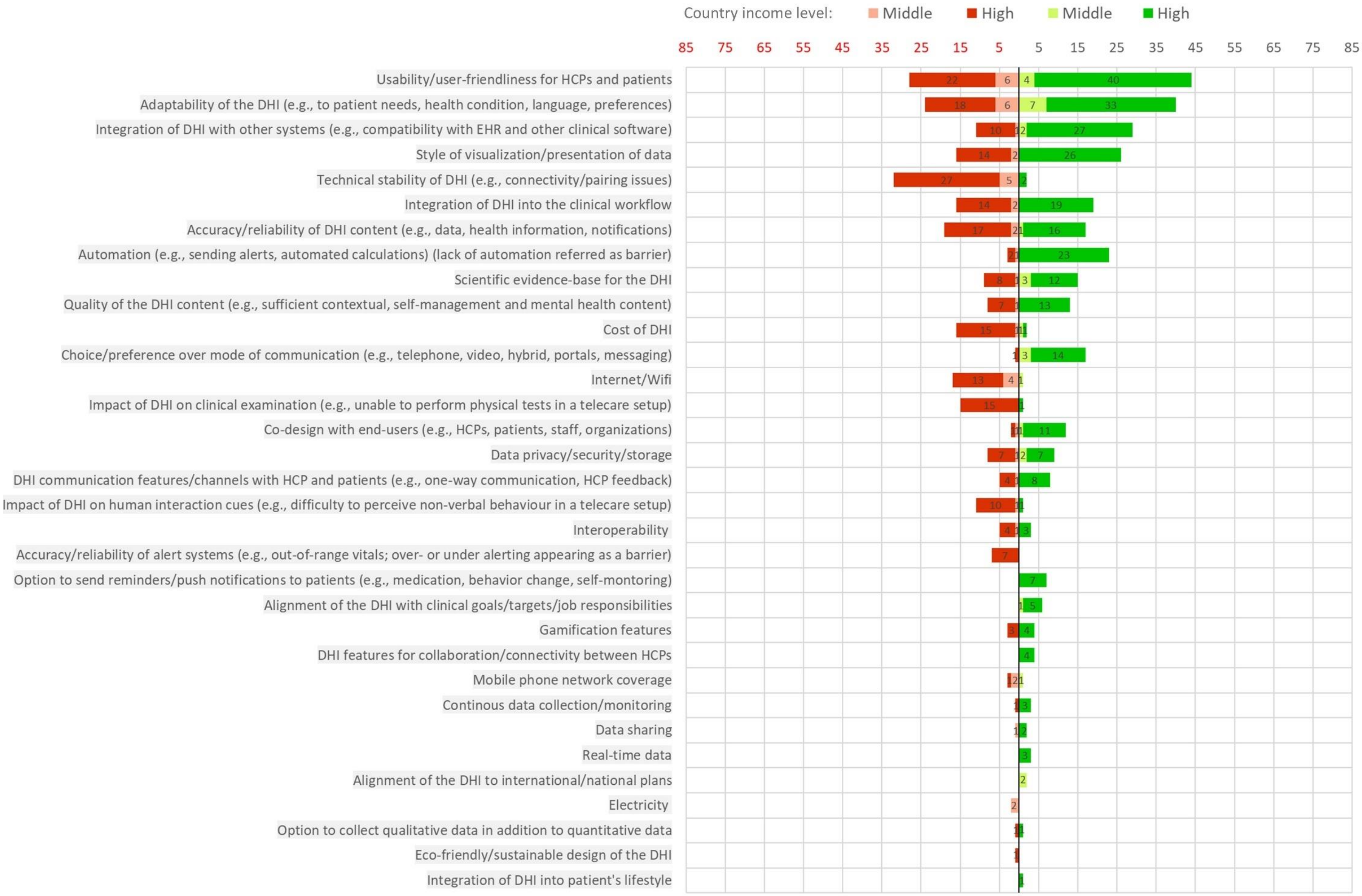

Figure S5.4. Factors relating to the health system

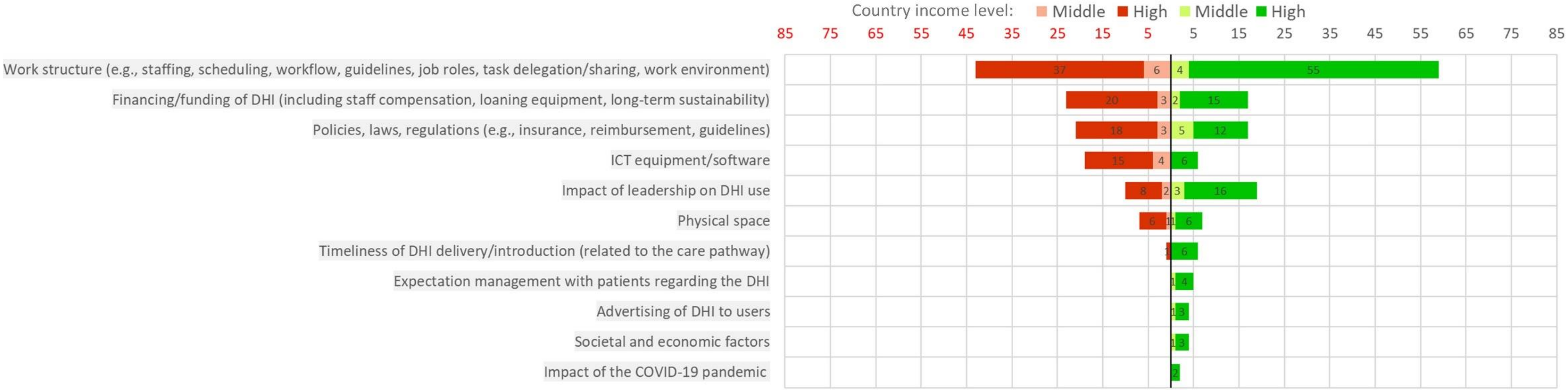

Supplement: Supplemental Files. — Supplemental file S1 to S5. [file gh-21-1-1561-s1.pdf]
